# Genomic Tracking of Market‐Derived Bull Shark Fins Back to Source Population of Origin

**DOI:** 10.1111/mec.70546

**Published:** 2026-09-16

**Authors:** Floriaan Devloo‐Delva, Pierre Feutry, Stanley Kwok Ho Shea, Huarong Zhang, Laura García Barcia, Diego Cardeñosa, Demian D. Chapman

**Affiliations:** ^1^ Australian National Fish Collection, NCMI CSIRO Hobart Tasmania Australia; ^2^ Environment CSIRO Hobart Tasmania Australia; ^3^ Bloom Association, Central Hong Kong S.A.R China; ^4^ The ADM Capital Foundation Ltd Hong Kong S.A.R China; ^5^ Kadoorie Farm and Botanic Garden Hong Kong, S.A.R China; ^6^ Department of Biological Sciences Florida International University North Miami Florida USA; ^7^ Lilly Research Laboratories Eli Lilly and Company Indianapolis Indiana USA; ^8^ Sharks and Rays Conservation Research Program Mote Marine Laboratory & Aquarium Sarasota Florida USA

**Keywords:** *Carcharhinus leucas*, DArTcap, DNA forensics, genetic sex, provenance assignment

## Abstract

International trade of shark fins remains difficult to monitor because products are rarely labelled to species and are often highly processed, resulting in severely degraded DNA. For several shark species listed under Appendix II of the Convention on International Trade in Endangered Species of Wild Fauna and Flora (CITES), this limits external verification of source populations supplying global trade hubs. Here, we assess whether nuclear genomic approaches can be applied to market‐derived bull shark ( *Carcharhinus leucas* ) fins to determine their population of origin. We analysed dried fin trimmings collected from retail vendors in Hong Kong SAR, one of the world's largest dried shark fin trade hubs, using a targeted DArTcap single nucleotide polymorphism (SNP) panel, originally developed for population genomic studies of this species. Despite substantial DNA degradation, genomic libraries were successfully obtained for most samples, yielding sufficient SNP data to perform robust provenance and sex assignment. Using a Bayesian mixed‐stock analysis, most fin samples were assigned to the Indo–West Pacific (71.4%), with smaller contributions from the western Atlantic (22.6%) and eastern Pacific (3.0%). Genetic sex assignment revealed twice as many males as females, although results indicated a conservative bias towards male assignment due to the limited number of X‐linked markers available in degraded samples. Our results demonstrate that genome‐wide targeted approaches can be effectively applied to highly processed shark fin products to infer population sources and sex composition. This study provides proof‐of‐concept for integrating genomics into shark trade monitoring, highlighting its potential to improve traceability, support CITES implementation and inform conservation and fisheries management, particularly for species with well‐resolved population structure.

## 1 Introduction

Shark‐derived commodities such as meat and dried fins are frequently traded across international borders, with supply chains of numerous fishing nations terminating in a smaller number of international trade hubs and consumer nations (Dent and Clarke 2015). While trade flows are reasonably well understood at the generic ‘shark’ level (Dent and Clarke 2015), species‐specific supply chains are often opaque because shark products are rarely labelled to species. Within the last decade, over 100 shark and ray species have been listed on Appendix II of the Convention on International Trade of Endangered Species (CITES), which permits international trade if it is compliant with the laws of the fishing nation, sustainable and documented (Booth 2026). Documentation of trade through CITES could better resolve species‐specific supply chains if compliance is high (Booth 2026). Genetic approaches (single or bi‐locus mitochondrial DNA sequencing) have recently been used to infer international trade flows by revealing source population contributions to trade hub or market samples for a few shark species (Cardeñosa et al. 2021; Chapman et al. 2009). Comparing genetic mixed‐stock analysis of a market sample of fins to CITES import records for the same market has often revealed discrepancies that can only be explained by undocumented and thus illegal trade (Cardeñosa et al. 2025; Fields et al. 2025). In rare cases, mitochondrial single nucleotide polymorphisms (SNPs) can be used to assign individual fins to a population of origin (Cardeñosa et al. 2026), but in most single‐locus studies too few diagnostic SNPs exist to allow for this and mixed‐stock analysis on a market sample is required (Fields et al. 2025). More comprehensive product tracking efforts might be facilitated by nuclear genomic approaches in which a larger number of loci are surveyed at once and each individual product in a sample can be reliably assigned provenance (Andrews et al. 2016; Green et al. 2019; Wenne 2023). Nuclear SNP panels have increasingly been applied in wildlife and food forensics, enabling reliable genetic inference from processed and traded animal products, including sex identification using sex‐linked markers (Arenas et al. 2017; Devloo‐Delva et al. 2024; Ogden 2011).

**FIGURE 1:**
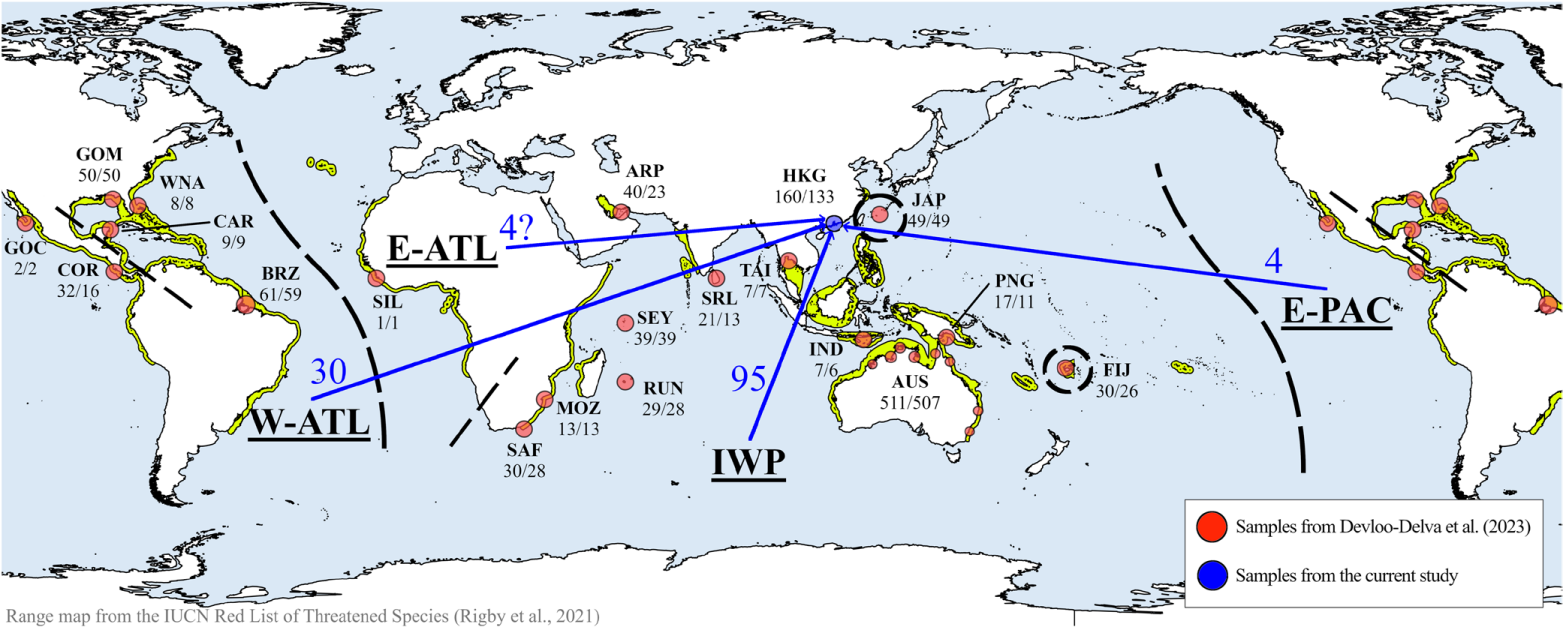
Map indicating the *Carcharhinus leucas* reference sample locations with red circles and the known species range distribution in yellow (modified from Devloo‐Delva et al. 2023). Region‐of‐origin assignment of Hong Kong fin trimming, according to *rubias*, are indicated in blue. The sample sizes are indicated with the number of samples before/after data filtering. Putative barriers for connectivity are indicated by dashed lines. ARP, Arabian Peninsula; AUS, Australia; BRZ, Brazil; CAR, Caribbean Sea; COR, Costa Rica; E‐ATL, eastern Atlantic Ocean; E‐PAC, eastern Pacific Ocean; FIJ, Fiji; GOC, Gulf of California; GOM, Gulf of Mexico; HKG, Hong Kong; IND, Indonesia; IWP, Indo‐West Pacific region; JAP, Japan; MOZ, Mozambique; PNG, Papua New Guinea; RUN, Réunion Island; SAF, South Africa; SEY, Seychelles; SIL, Sierra Leone; SRL, Sri Lanka; TAI, Thailand; W‐ATL, western Atlantic Ocean and WNA, Western North Atlantic.

The bull shark ( *Carcharhinus leucas* ) is a large‐bodied, circumtropical shark species that is listed as globally ‘Vulnerable’ by the International Union for the Conservation of Nature (IUCN) and was listed on CITES Appendix II in 2022 (Rigby et al. 2021). The species is fished in many parts of the world that do not adequately manage shark fisheries for sustainability (Rigby et al. 2021), and bull shark depletion might be especially problematic because it is an apex predator species that could disproportionately influence ecosystem structure and function (Dedman et al. 2024). The international dried shark fin trade is a major driver of bull shark fishing, and their fins are sold in one of the world's largest fin trade hubs (Hong Kong Special Administrative Region of the People's Republic of China, hereafter ‘Hong Kong’ or HKG) under a specific high‐value trade name (‘Sha qing’). Bull sharks made up at least 1.8%–2.6% of the Hong Kong fin trade from October 1999 to March 2001 (Clarke, Magnussen, et al. 2006), which was used to estimate global mortality of a few hundred thousand to ~1.5 million individuals per year (Clarke, McAllister, et al. 2006). More recent fin market surveys from 2014–2021 confirm this species remains among the most common species in the market (Cardeñosa et al. 2024, 2022). Labelling of Sha qing fins takes place after the fin is imported, and there is limited information on the global supply chains for bull shark fins. Bull sharks are coastal and philopatric, with unique mitochondrial control region sequences characterising populations occupying continental shelves separated by wide oceanic expanses and cold water. Recent genomic studies have further refined knowledge of the species population structure, detecting more localised structure, using nuclear SNPs (Devloo‐Delva et al. 2023; Glaus et al. 2020; Postaire et al. 2024), suggesting that genomic analyses could clarify source areas supplying bull shark fins to trade hubs like Hong Kong. Specifically, Devloo‐Delva et al. (2023) determined that 250–500 informative SNPs were sufficient to achieve a near 100% provenance assignment accuracy at ocean basin and island population level (i.e., eastern Pacific, E‐PAC; western Atlantic, W‐ATL; eastern Atlantic, E‐ATL; Indo‐West Pacific, IWP; Japan, JAP and Fiji; FIJ; see Figure 1). Only E‐ATL (Sierra Leone), which had one sample, could not be validated sufficiently.

Dried fins are often processed (skinned and bleached) quickly after being imported, which substantially degrades genomic DNA. Although this has been overcome with mini‐DNA barcoding to identify species and piece‐meal reconstruction of one or two loci for DNA zip coding, it may pose challenges for some genomic workflows, particularly reduced‐representation approaches that rely on restriction enzyme digestion and recovery of longer fragments. However, specialised short‐read sequencing methods can often recover genomic information from degraded material (Domingues et al. 2021; Hahn et al. 2022). The goal of this study was to determine whether genome‐wide targeted approaches can be used to assess the source populations supplying a global trade hub with dried fins, using market‐derived fin samples that are known to contain highly degraded DNA. We used an existing global population genomic dataset for bull sharks in which 1849 nuclear SNPs define six differentiated populations as a baseline of potential population sources (Devloo‐Delva et al. 2023). Dried fin trimmings, which are small pieces of dried tissue (muscle, skin, cartilage) excised from processed fins, and collected in Hong Kong, were used as a market sample. Our objectives were to (1) determine if standard genomic workflows and analyses yielded robust population genomic data from a market sample composed of a typical processed fin product available in southeast Asia and (2) obtain initial insights into the main fishing areas that supply bull shark fins to the world's largest dried shark fin hub.

## 2 Material and Methods

### 2.1 Sample Collection

Bull shark fin trimmings were collected from random retail vendors in Hong Kong between 2014 and 2018 (*n* = 160; Figure 1). Species identity was previously established by Cardeñosa et al. (2024); Cardeñosa et al. (2020) using a multiplex mini‐barcode PCR assay to amplify and sequence two small amplicons (150–200 bp of the mitochondrial cytochrome oxidase I gene; Cardeñosa et al. 2017).

### 2.2 DNA Extraction and Genotyping

Prior to tissue digestion, each fin clip was rinsed with milli‐Q water to remove any potential cross‐contaminating dust or flakes. DNA was extracted using the Qiagen Blood and Tissue kit following the standard protocol (Qiagen Inc., Valencia, California, USA). DNA quality and quantity were assessed on a 1% agarose gel, stained with SYBR safe (Invitrogen, USA), and with the NanoDrop ND‐8000 spectrophotometer (ThermoFisher Scientific, Waltham, Massachusetts, USA).

DNA was sent to Diversity Arrays Technologies (DArT; Canberra, Australia) for DArTcap library preparation and sequencing, as described by Feutry et al. (2020). DArTcap involves adding a hybridisation‐based enrichment step before the DArTseq libraries are sequenced. The hybridisation step uses custom synthesised biotinylated RNA MYbaits (Arbor Bioscience) designed based on the chosen DArTseq markers. Specifically, 3410 baits, including 210 putatively located on sex chromosomes, were designed by Devloo‐Delva et al. (2023). The DArTcap enriched libraries were sequenced on a HiSeq 2500 (Illumina, San Diego, California, USA).

### 2.3 Data Quality Filtering

The resulting sequencing data was pooled with data from Devloo‐Delva et al. (2023) to identify common SNPs. Sequence clustering was conducted using DArT's proprietary DArT‐Soft14 analytical pipeline and allele counts were loaded into the R package *radiator* v1.4.0 for SNP calling and data quality control (Gosselin et al. 2020). For SNP loci, the following parameters were assessed: (i) Reproducibility: proportion of consistent genotype calls, estimated using technical replicates (13 HKG samples were prepared and sequenced twice); (ii) Minor allele count (MAC): frequency of the less common allele; (iii) Coverage: average read depth per locus across all individuals; (iv) Call rate: proportion of individuals without missing data at a locus; (v) SNP position: location of the SNP within the sequenced fragment; (vi) Short linkage disequilibrium: for multiple SNPs on the same fragment, only the SNP with the highest MAC (most informative) was retained; (vii) Hardy–Weinberg equilibrium (HWE): evaluated within each sampling location. For individual samples, filtering included: (i) Missingness: proportion of missing genotype data per individual; (ii) Heterozygosity: proportion of loci with two different alleles; (iii) Duplicate: pairwise measure of genetic distance between individuals, with samples exhibiting genetic distance < 0.007 considered technical replicates or duplicate individuals. Individual‐level filtering parameters were relaxed to retain as many fin trade samples as possible given the lower DNA qualities, whereas SNP filtering was more stringent. Thresholds applied for each parameter during filtering are detailed in Table 1.

**TABLE 1:** Data quality filtering with the *radiator* package.

Filtering steps	Filtering parameters	Initial (ind/pop/loci/SNPs)	Removed (ind/pop/loci/SNPs)
Filter DArT reproducibility	0.98	1137/35/7458/8364	0/0/1354/1651
Filter monomorphic markers	NA	1137/35/6104/6713	0/0/0/0
Filter markers in common	NA	1137/35/6104/6713	0/0/1556/1762
Filter individuals based on missingness	0.8	1137/35/4548/4951	8/0/0/0
Filter individuals based on heterozygosity	[0–0.09]	1129/35/4548/4951	23/0/0/0
Filter individuals based on total coverage	[2000 – 200,000]	1106/35 / 4548/4951	0/0/0/0
Filter monomorphic markers	NA	1106/35/4548/4951	0/0/458/538
Filter Minor Allele Count	2	1106/35/4090/4413	0/0/258/296
Filter coverage min/max	[6–62]	1106/35/3832/4117	0/0/1235/1308
Filter genotyping	0.1	1106/35/2597/2809	0/0/223/257
Filter SNPs position on the read	all	1106/35/2374/2552	0/0/0/0
Filter number SNPs per locus	1	1106/35/2374/2552	0/0/165/343
detect mixed genomes (heterozygosity)	0 1	1106/35/2209/2209	0/0/0/0
detect duplicate genomes (replicates)	0.007	1106/35/2209/2209	71/0/0/0
Filter monomorphic markers	NA	1035/35/2209/2209	0/0/0/0
Filter HWE	4 locations; *p* < 0.05	1035/35/2209/2209	0/0/67/67
Final data		1035/35/2142/2142	

*Note:* The number of individuals (ind), sampling locations (pop), unique RADtags of 70 bp length (loci) and SNP markers (SNPs) before and after each filtering step are presented.

### 2.4 Provenance and Sex Assignment

We assessed the provenance assignment to the six previously identified populations: eastern Pacific (E‐PAC), western Atlantic (W‐ATL), eastern Atlantic (E‐ATL), Indo‐West Pacific (IWP), Japan (JAP) and Fiji (FIJ). First, a discriminant analysis of principal components (DAPC; Jombart et al. 2010) was performed in *adegenet* v2.1.11 to discriminate the individuals from each genetic group and samples of unknown origin (i.e., Hong Kong fin trade samples) were assigned using ‘predict.dapc’ function. The number of PCs was selected based on ‘xvalDapc’ cross‐validation (see Supporting Information). DAPC is particularly advantageous for analysing complex datasets characterised by overlapping or closely related genetic groups, as it maximises between‐group variance while minimising within‐group variance, thereby enhancing the resolution of group discrimination. Yet, since the clustering algorithm can be biased by unequal sample sizes (Foster et al. 2018; Puechmaille 2016), the reference IWP population was subsampled to 120 individuals. Secondly, a mixed‐stock analysis in *rubias* v0.3.4 was performed to assign the fin trade samples to their putative population of origin (Moran and Anderson 2019). *Rubias* is a Bayesian hierarchical genetic stock assignment approach that accounts for population structure among baseline populations using a mixture set of unknown origin and a reference set of known origin (Anderson et al. 2008). Posterior density curves and 95% credible intervals for mixture proportions were created from Markov Chain Monte Carlo (MCMC) output or parametric bootstrapping with 10,000 iterations. DAPC and *rubias* were used as complementary assignment approaches. DAPC provided individual‐based assignment and visualisation of genetic clustering, whereas *rubias* provided a Bayesian stock‐assignment framework and estimates of mixture proportions. Concordance between methods was used as an additional measure of assignment robustness.

A principal components analysis (PCA) was performed on the full data with *adegenet* to visualise the placement of the HKG samples in respect to reference samples. Unlike DAPC, which maximises discrimination among predefined groups, PCA is an unconstrained ordination that was used here primarily to visualise overall genetic structure and the behaviour of samples with varying levels of missing data.

Using the 14 X‐linked SNP markers identified in Devloo‐Delva et al. (2023), the putative genetic sex was assigned using a K‐means clustering algorithm implemented in *dartR.sexlinked* v1.0.5 (Mijangos et al. 2022; Robledo‐Ruiz et al. 2023). Sex identification from X‐linked markers relies on homozygous genotypes for all X‐linked markers in males while expecting several heterozygous genotypes in females (Devloo‐Delva et al. 2024). To minimise misassignment arising from genotyping error, individuals with only one or two heterozygous markers (7%–14%) were classified as males. Nevertheless, this method may still lead to females being misassigned as males if insufficient markers are available. Therefore, to quantify the uncertainty associated with market‐level sex ratio estimates, we calculated the rate of male‐to‐female and female‐to‐male misassignment based on individuals with known phenotypic sex (i.e., presence or absence of claspers). Here, we assume that the visual sex assignment is correct.

## 3 Results

### 3.1 DNA Extraction, Genotyping and Quality Filtering

Because fins undergo intensive processing, DNA was degraded in most samples (< 600 bp; Supporting Information), and 22 samples failed library construction. All 151 libraries (including 13 technical replicates) yielded sequencing data with an average of 105,078 reads per sample and an average read depth of 9.2 for 8364 SNPs.

**FIGURE 2:**
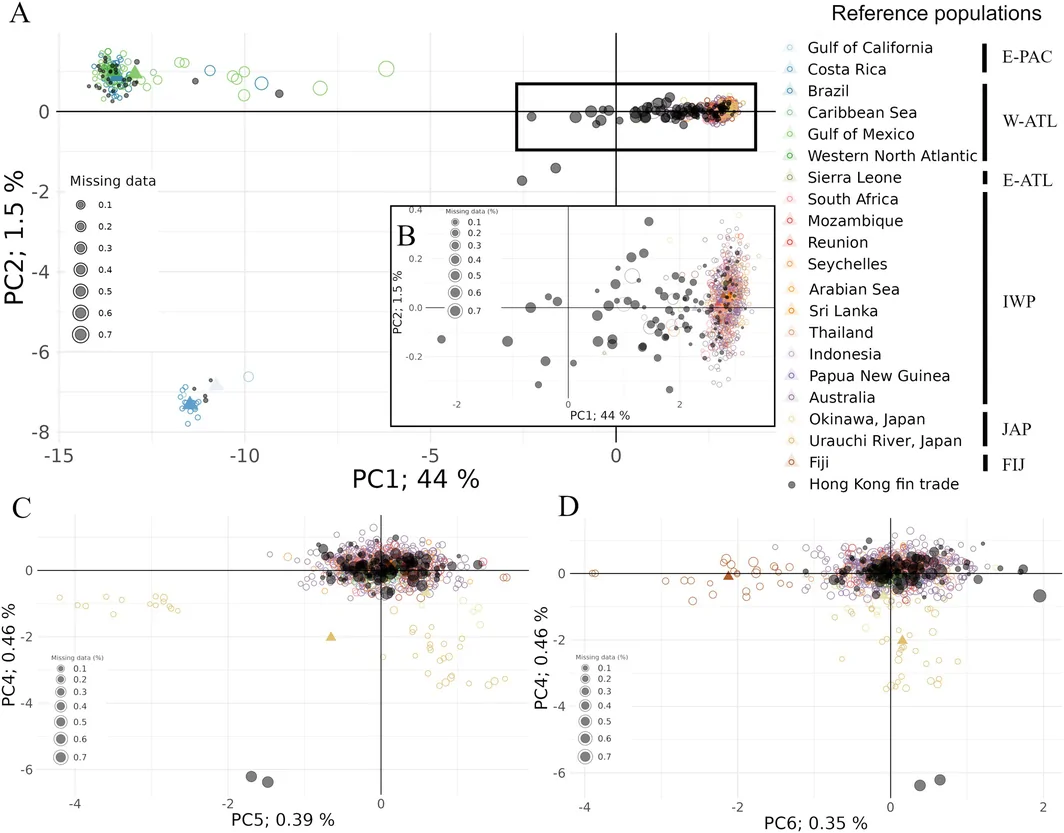
Principal component analysis (PCA) for the global *Carcharhinus leucas* reference data and Hong Kong fin samples of unknown origin. The data of 2142 SNPs represents 895 sharks from reference populations (including 59 technical replicates), and 140 samples from Hong Kong fin trade (including seven replicates). Each point represents an individual shark; the point size reflects the proportion of missing data per individual; triangles indicate the mean PCA score per sampling location; and colours represent the sampling country or oceanographic location: E‐ATL, eastern Atlantic Ocean; E‐PAC, eastern Pacific Ocean; IWP, Indo‐West Pacific region and W‐ATL, western Atlantic Ocean. (A) PCA scatterplot with PC1 on the x‐axis and PC2 on the y‐axis. (B) Inset of panel A, with a focus on IWP. (C) PCA scatterplot with PC4 on the x‐axis and PC5 on the y‐axis. (D) PCA scatterplot with PC4 on the x‐axis and PC6 on the y‐axis. PC3 is not shown because it was highly similar to PC2 and primarily reflected variation associated with missing data.

After relaxed quality filtering, we maintained 2142 SNPs for 140 HKG samples, containing seven technical replicates. Eleven HKG samples (including six replicates) were removed during individual‐level filtering based on high missing data and heterozygosity levels and obvious duplicate detection. Given the reduced quality filtering parameters, many HKG samples still had high missing data (*n* = 42 with > 20% missing data) and high heterozygosity (*n* = 5 with > 10% heterozygous genotypes). We see that these samples have an intermediate placement along PC1 in the PCA (Figure 2B). Nevertheless, the overall genetic structure was consistent with previous studies. PC1 explained 44% of the total genetic variance and primarily differentiated the IWP from the W‐ATL and E‐PAC populations, whereas PC2 separated E‐PAC from the remaining regions (Figure 2A). At finer spatial scales, PC4 and PC6 distinguished the Japan and Fiji populations, respectively, although each axis explained less than 0.45% of the total variance (Figure 2C,D).

### 3.2 Provenance and Sex Assignment

Posterior group assignment (K = 6; PCs = 95) was used to identify the region of origin with the DAPC analysis. All samples were assigned to one of three regions: E‐PAC, W‐ATL, or IWP (Table 2). Similar results were found for *rubias* assignment with MCMC and parametric bootstrapping methods, but four samples that DAPC assigned to IWP were reported as originating from E‐ATL. It is worth noting that 40 HKG samples that were assigned to IWP had an intermediate placement in PCA space between IWP and E‐ATL (see Figure 2B). The *rubias* z‐scores, which provide a measure of how well individuals fit the reference baseline populations, were approximately normally distributed. Lower values were significantly correlated with missing data (r = −0.41; *p* < 0.001). Assignment accuracy during self‐assignment of the reference dataset was high for both DAPC and *rubias* (100%; see Supporting Information), with lower assignment confidence generally observed among individuals with elevated levels of missing data. Overall, most of the samples were assigned to the IWP (*n* = 101–105, including six replicates) and W‐ATL (*n* = 31, including one replicate) populations. All technical replicates were assigned the same reference population.

**TABLE 2:** Provenance assignment under different algorithms.

reference group	DAPC	Rubias – MCMC	Rubias – parametric bootstrap
eastern Pacific (E‐PAC)	4 (4)	4 (4)	4 (4)
western Atlantic (W‐ATL)	31 (30)	31 (30)	31 (30)
eastern Atlantic (E‐ATL)	0	4 (4)	4 (4)
Indo‐West Pacific (IWP)	105 (99)	101 (95)	101 (95)
Japan (JAP)	0	0	0
Fiji (FIJ)	0	0	0

*Note:* The number in brackets represents the number of unique individuals (technical replicates removed).

Genetic sex identification based on individuals of known sex (*n* = 853) showed that female‐to‐male misassignment (i.e., *P* (*phenotypic F | genetic M*)) occurred in 19.7% of cases and male‐to‐female misassignment (i.e., *P* (*phenotypic M | genetic F*)) in 10.5%. Because sex assignment relies on only 14 X‐linked markers, it could be performed before quality filtering on a larger subset of samples (*n* = 138 unique individuals) than provenance assignment, which required the full filtered SNP panel (*n* = 133 unique individuals). The number of available X‐linked loci varied among individuals because of missing data associated with DNA degradation. On average, 12.5 X‐linked markers were genotyped per individual, but some only had 3 markers. When applying this method to the HKG samples, we genetically assigned 43 individuals as female and 95 as male. Based on the observed misassignment rates in the validation dataset, the true number of females and males was estimated to lie between 38.5–61.7 and 76.3–99.5 individuals, respectively. All technical replicates were assigned the same sex.

## 4 Discussion

This study provides the first demonstration that genomic data can be used to assign shark tissues of unknown origin to their source population, addressing a long‐standing gap in the monitoring of shark trade products (Domingues et al. 2021). Despite the highly degraded nature of the material, the standard DArTcap genomic workflow yielded sufficient data for most samples, with failures limited to a subset of libraries that could not be constructed (*n* = 22). The key outcome of this study is not simply that genomic data could be recovered from degraded fins, but that these data were sufficient to generate biologically meaningful inferences regarding source populations and sex composition in a globally traded shark species. Overall, SNP data enabled assignment of both provenance and genetic sex for most Hong Kong fin trimming samples (*n* = 133 and *n* = 138 unique sharks, respectively). Since fin trimmings are simply small pieces excised from whole fins to make them more visually appealing after they have been fully processed, it is reasonable to conclude that the typical dried shark fins found in Asian markets will be amenable to this type of genomic analysis across multiple species with similar processing histories.

Provenance assignment accuracy was nonetheless influenced by the substantial proportion of missing data observed in some samples, a pattern clearly visible in the PCA (Figure 2B). This loss of information is attributed to the severe processing of fins prior to trade, including bleaching and drying. However, given the strong global population structure of bull sharks, high levels of missing data did not compromise population‐level assignments in this species (Manel et al. 2002). Caution is warranted when applying similar genomic approaches to species with weaker genetic differentiation (e.g., Nikolic et al. 2023), where missing data may have a disproportionate effect on assignment confidence. Additionally, while global sampling of reference populations was achieved by Devloo‐Delva et al. (2023), caution is advised for interpreting samples with high missing data that match unsampled (e.g., putative island populations) or under‐sampled (E‐ATL) regions. Notably, all four samples assigned to the eastern Atlantic by *rubias* exhibited > 47% missing data and poor model fit (low z‐scores), suggesting these assignments are driven by data loss rather than true E‐ATL provenance. Overall, confidence in the provenance assignments is supported by the strong concordance among both assignment analyses (DAPC and *rubias*) and visual inspection of PCA clustering, as well as the complete agreement observed among technical replicates. Although samples with high missingness tended to occupy intermediate positions in PCA space and showed lower *rubias* z‐scores, indicating reduced assignment confidence, their population‐level assignments were generally consistent across analytical approaches. Together, these results suggest that the major source regions identified here are robust to both assignment methodology and variation in DNA quality.

Genomic analysis provided an additional advantage over single‐locus approaches by enabling genetic sex identification using X‐linked SNP markers. The method exhibited a conservative bias towards male assignment because reliable female identification requires heterozygosity at multiple X‐linked loci. Confidence in sex assignment varied among samples and was positively related to the number of genotyped X‐linked markers, which itself was affected by DNA degradation. At the market level, the sex‐ratio estimate revealed twice as many males (2.21:1 M:F). When the underlying sex ratio approaches parity (1:1), even relatively low misassignment rates can have a disproportionate effect on the inferred sex composition. Therefore, the sex ratios reported here should be interpreted as broad indicators of market composition rather than precise demographic estimates.

Compared with single‐locus mtDNA‐based approaches, nuclear SNPs offer several advantages for provenance analysis, including biparental inheritance, independence among loci and reduced sensitivity to homoplasy and stochastic drift (Attard et al. 2022; Ballard and Whitlock 2004; Estoup et al. 2002; Hassanin et al. 1998). Previous mtDNA‐based studies of the shark fin trade usually did not assign provenance to each product. Instead, population contributions were estimated for the whole sample with mixed‐stock analysis, often with broad confidence intervals (Fields et al. 2020). In contrast, the approach used here assigned provenance to each fin product with explicit, quantitative measures of data quality, such as missingness and heterozygosity, which are critical when working with degraded market samples. Robust provenance assignment of individual products will provide more accurate estimates of population contribution to the market. Conversely, because sharks typically exhibit strong female philopatry and male‐biased dispersal (Chapman et al. 2015; Phillips et al. 2021), single locus mtDNA may reveal finer‐scale structure than nuclear SNPs in some contexts and thus higher geographic precision. However, when DNA is highly degraded, mtDNA analyses require very short amplicons. This can exacerbate homoplasy, particularly in the mitochondrial control region, which is often used for provenance assignment. Taken together, our results highlight targeted SNP panels as a robust and complementary tool for species identification, provenance assignment and sex determination in the monitoring of shark trade (Domingues et al. 2021).

We found that most bull shark fins sampled originated from the IWP (71.4%) and W‐ATL (22.6%). Although our market sampling does not overlap with the period when this species was listed on CITES Appendix II, a comparison with the CITES Trade Database for 2024 (i.e., the first full year that the species was listed), reveals broad concordance (https://trade.cites.org/). The primary exporters of bull shark fins to Hong Kong were from the IWP (Sri Lanka, Indonesia), followed by Mexico (which would be expected to supply fins from the W‐ATL and E‐PAC). This coincides with the top exporting countries of shark fins (Rodenbiker et al. 2023; Shea and To 2017). Future studies will be able to directly compare genomic assignments from market samples with CITES trade records to detect discrepancies that are indicative of undocumented and thus illegal trade at points of aggregation within global supply chains (e.g., Tinsman et al. 2023).

This study provides proof‐of‐concept that genomic approaches can deliver actionable information on the global supply chains underpinning the trade of sharks, even for highly processed products like dried and bleached fins. Most samples yielded sufficient data for robust inference despite substantial DNA degradation, demonstrating the feasibility of applying genomic traceability tools to real‐world market products. Realising the full potential of this approach will require expanded global population‐genomic baselines for CITES‐listed species, with comprehensive geographic coverage to minimise ascertainment bias and maximise assignment accuracy (e.g., Feutry et al. 2020; Nikolic et al. 2023; Wagner et al. 2024). Such baselines would increase the spatial precision of inferences about the supply chains involved and enable routine, independent verification of declared trade origins. Beyond provenance, genomics can also reveal biologically meaningful patterns in trade, including skewed sex ratios (here 2.21 males per female), which may raise additional conservation concerns for species with sex‐biased vulnerability or nursery area exploitation (Cardeñosa et al. 2024; Phillips et al. 2021). Future efforts should focus not only on expanding genomic reference resources but also on translating these datasets into cost‐effective monitoring tools, such as informative SNP panels or targeted SNP‐chip assays that retain assignment power while reducing analytical costs and turnaround times. Currently, one‐third of species traded in the Hong Kong shark fin market are threatened with extinction and have limited fisheries management (Fields et al. 2018). Integrating genomic monitoring into regulatory frameworks therefore offers a powerful, scalable pathway to improve traceability, support CITES implementation, detect undocumented trade and better align shark fisheries exploitation with conservation and management objectives.

## Author Contributions

Floriaan Devloo‐Delva, Pierre Feutry and Demian D. Chapman conceived and designed the study; Stanley Kwok Ho Shea, Huarong Zhang, Laura García Barcia, Diego Cardeñosa and Demian D. Chapman collected and aggregated samples; Floriaan Devloo‐Delva conducted lab work; Floriaan Devloo‐Delva analysed the data; Floriaan Devloo‐Delva, Pierre Feutry and Demian D. Chapman secured funding; Floriaan Devloo‐Delva and Demian D. Chapman drafted the manuscript and all coauthors (Pierre Feutry, Stanley Kwok Ho Shea, Huarong Zhang, Laura García Barcia and Diego Cardeñosa) revised and improved the manuscript.

## Funding

This work was supported by Sea World Research and Rescue Foundation, 27.

## Ethics Statement

Fin trimmings used in this study were acquired from various retail vendors in Hong Kong. As these samples originated from processed products and no live animals were handled or sampled as part of this study, no animal ethics approval or sampling permits were required. All samples from reference populations were originally collected for other purposes, including fisheries monitoring and biodiversity surveys, and were re‐used to avoid additional invasive sampling. Sample collection was conducted under relevant animal ethics approvals, research permits, fisheries permits and institutional guidelines in the respective jurisdictions (described in Devloo‐Delva et al. 2023), including Australia, Papua New Guinea, the United States, Japan, Fiji, Brazil, Costa Rica, Sri Lanka, Réunion, Seychelles, South Africa and countries across the Western North Atlantic, Gulf of California, Arabian Peninsula, Africa and Southeast Asia. Where applicable, samples were obtained from fisheries landings, markets, bycatch, or shark control programs and therefore did not require specific animal ethics approval. Collection in Papua New Guinea was undertaken with the consent of local communities and supervision from provincial fisheries authorities. All import, export and transport of shark tissues complied with relevant national regulations and permit requirements. Samples were collected, shipped and received prior to the inclusion of requiem sharks (*Carcharhinidae*) on CITES Appendix II (November 2023).

## Conflicts of Interest

The authors declare no conflicts of interest.

## Supporting information


**Data S1:** An annotated R Markdown document containing the complete analytical workflow for this study, including data processing, quality control, SNP filtering, population genetic analyses and provenance assignment procedures, together with all associated results, figures and summary tables.

## Data Availability

Sample metadata, an Rmarkdown document which includes all performed analyses discussed in this study, and raw DArTcap and filtered genotype data will be available via the CSIRO Data Access Portal: https://doi.org/10.25919/4bca‐xc12 (Devloo‐Delva et al. 2026). The data for the reference populations are already available on DataDryad: https://doi.org/10.5061/dryad.4qrfj6qdg.
