# Supplementary material for: Genomic Tracking of Market‐Derived Bull Shark Fins Back to Source Population of Origin
Source: Mol Ecol. 2026 Sep 16;35(18):e70546. doi: 10.1111/mec.70546 (PMC13583026; doi:10.1111/mec.70546)
Supplement: Supplementary file 1 [file MEC-35-e70546-s001.pdf]

Supplemental Information for:

Genomic tracking of market-derived bull shark fins back to source population of origin

Floriaan Devloo-Delva*, Pierre Feutry, Stanley Kwok Ho Shea, Huarong Zhang, Laura Garcia-Barcia, Diego Cardeñosa, and Demian Chapman

* *Author produced this Rmarkdown*

May 2026

Contents

Background **2**

Sampling map and metadata **2**

Visual sex ID . . . 2

Total lengths . . . 3

Aproximate Length-at-age . . . 3

Catch date . . . 5

Age . . . 5

Cohort . . . 7

DNA quality gels **8**

Load packages **11**

DArTcap - All data **14**

Set strata . . . 14

Identify sex-linked markers . . . 14

Sex assignment . . . 15

Filter data . . . 18

Save data . . . 18

Load data . . . 19

FST . . . 21

PCA . . . 23

With HKG . . . 23

Without HKG . . . 37

Provenance assignment . . . 44

DAPC assignment . . . 44

Rubias assignment . . . 76

Compare DAPC to rubias . . . 88

Reference **90**

Citations for packages **90**

Session info **90**

Background

This Rmarkdown document described the data-filtering and analysis steps for assessing provenance of the Bull Shark (*Carcharhinus leucas*) from Hong Kong fin market samples.

1. Hundred-eighty-eight sharks, with 32 technical replicates, were sequenced and genotyped according to the DArTseq protocol (see Feutry et al. 2017) to yield 250,950 single nucleotide polymorphisms (SNPs).
   - The data with only Bull Sharks was used to select approximately 3400 SNPs to design targeted capture probes for DArTcap genotyping (see Feutry et al. 2020).
2. We analysed the resulting DArTcap SNPs for 940 individuals and an additional 74 technical replicates.
   - The data with only Bull Sharks was analysed for provenance assignment
   - 133 unique shark samples from the Hong Kong fin trade market were assigned to the most likely population of origin.

Sampling map and metadata

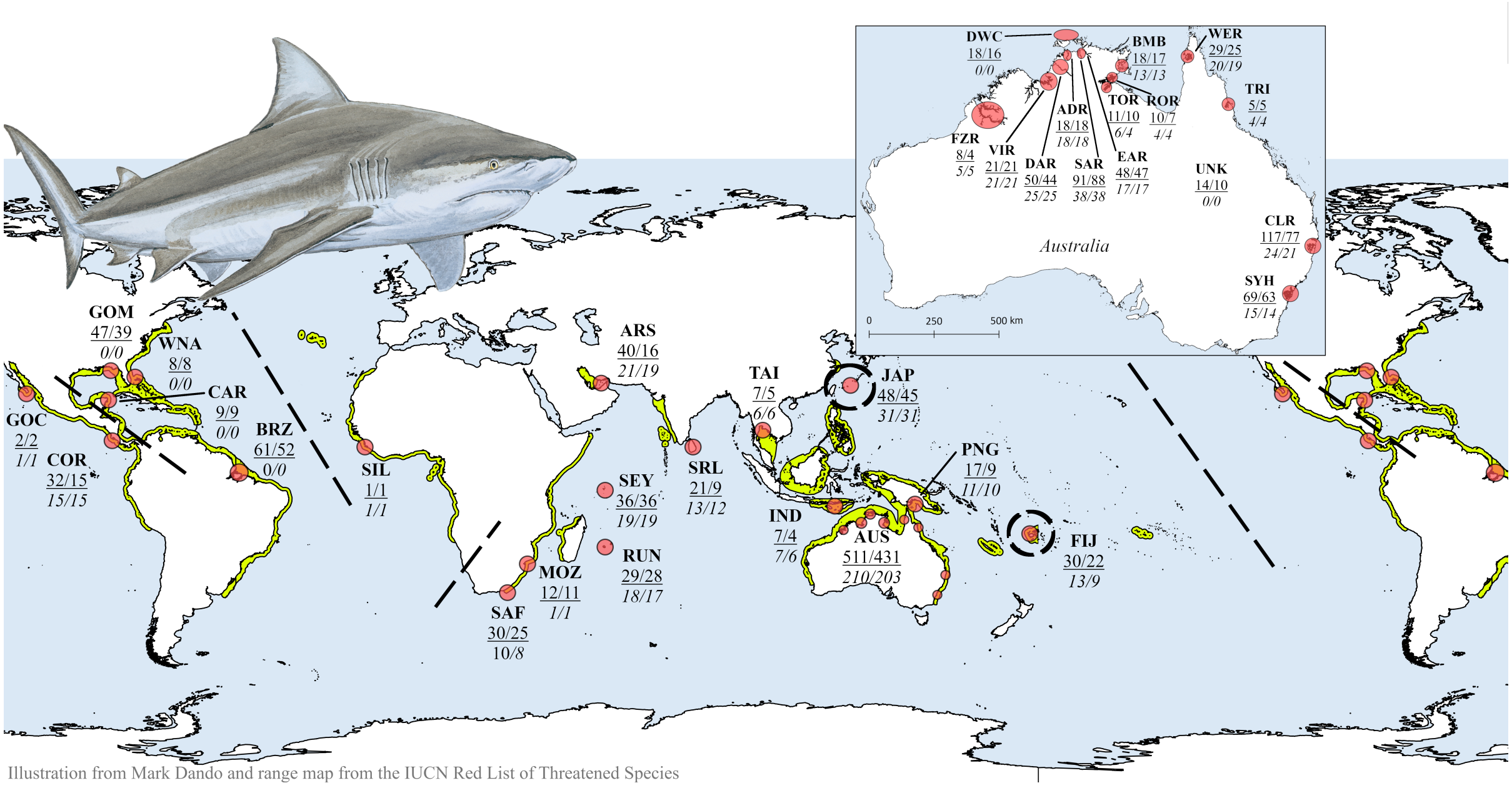

Illustration from Mark Dando and range map from the IUCN Red List of Threatened Species

Visual sex ID

```r
metafile <- "1.BullShark_AllSamples_metadata.csv"
meta <- readr::read_csv(metafile) %>%
  dplyr::filter(SpeciesID_genetic == "Carcharhinus leucas")
pop.levels <- c("Gulf of California", "Costa Rica" , "Caribbean Sea", "Gulf of Mexico",
                "Western North Atlantic", "Brazil", "Sierra Leone", "South Africa",
                "Mozambique", "Reunion" , "Seychelles", "Arabian Sea", "Sri Lanka",
                "Thailand", "Indonesia", "Papua New Guinea" , "Australia", "Japan",
                "Fiji")
meta$Site <- factor(meta$Site, levels = pop.levels)
meta$Date_sampled <- as.Date(meta$Date_sampled, "%d/%m/%Y")
```

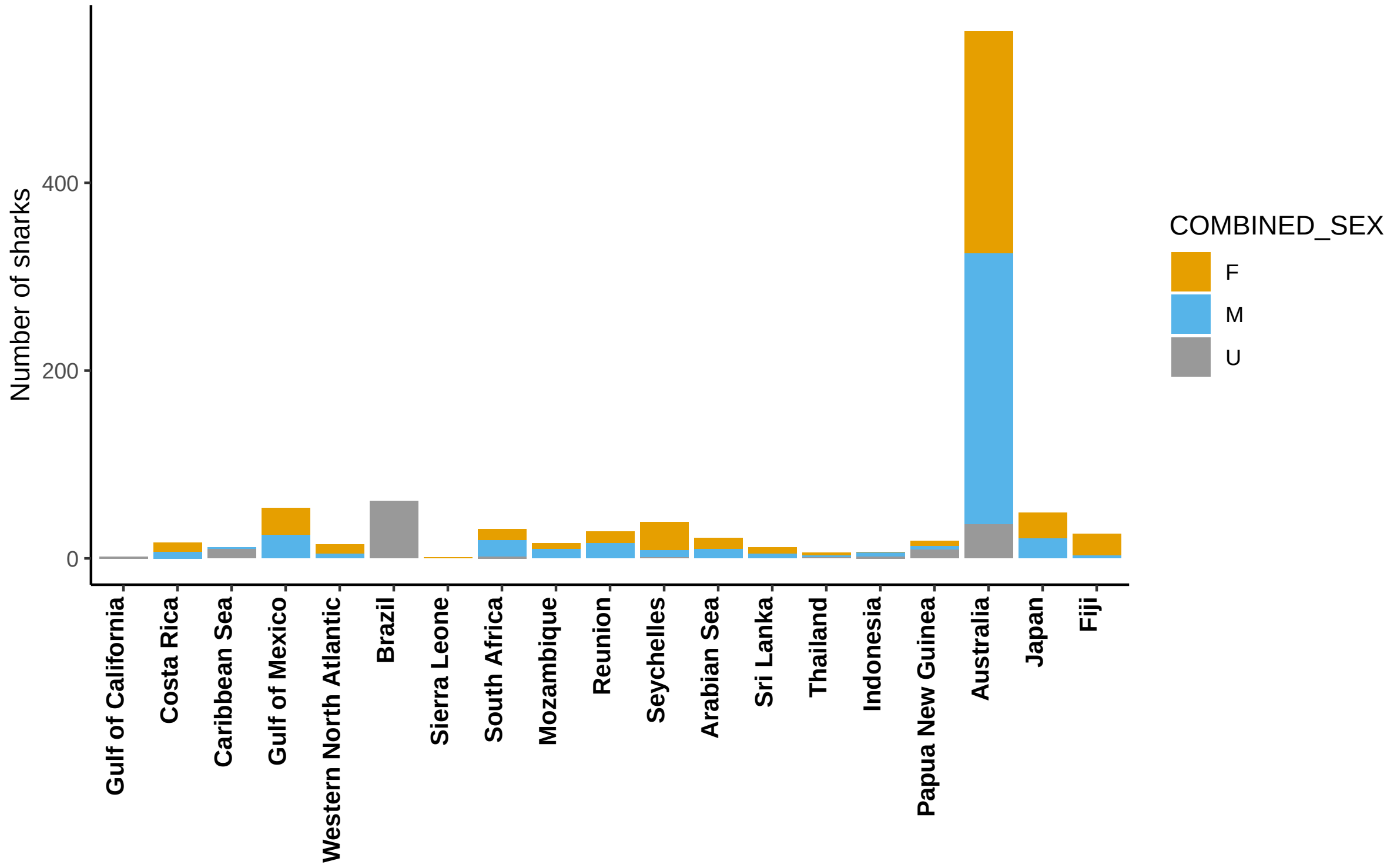

Total lengths

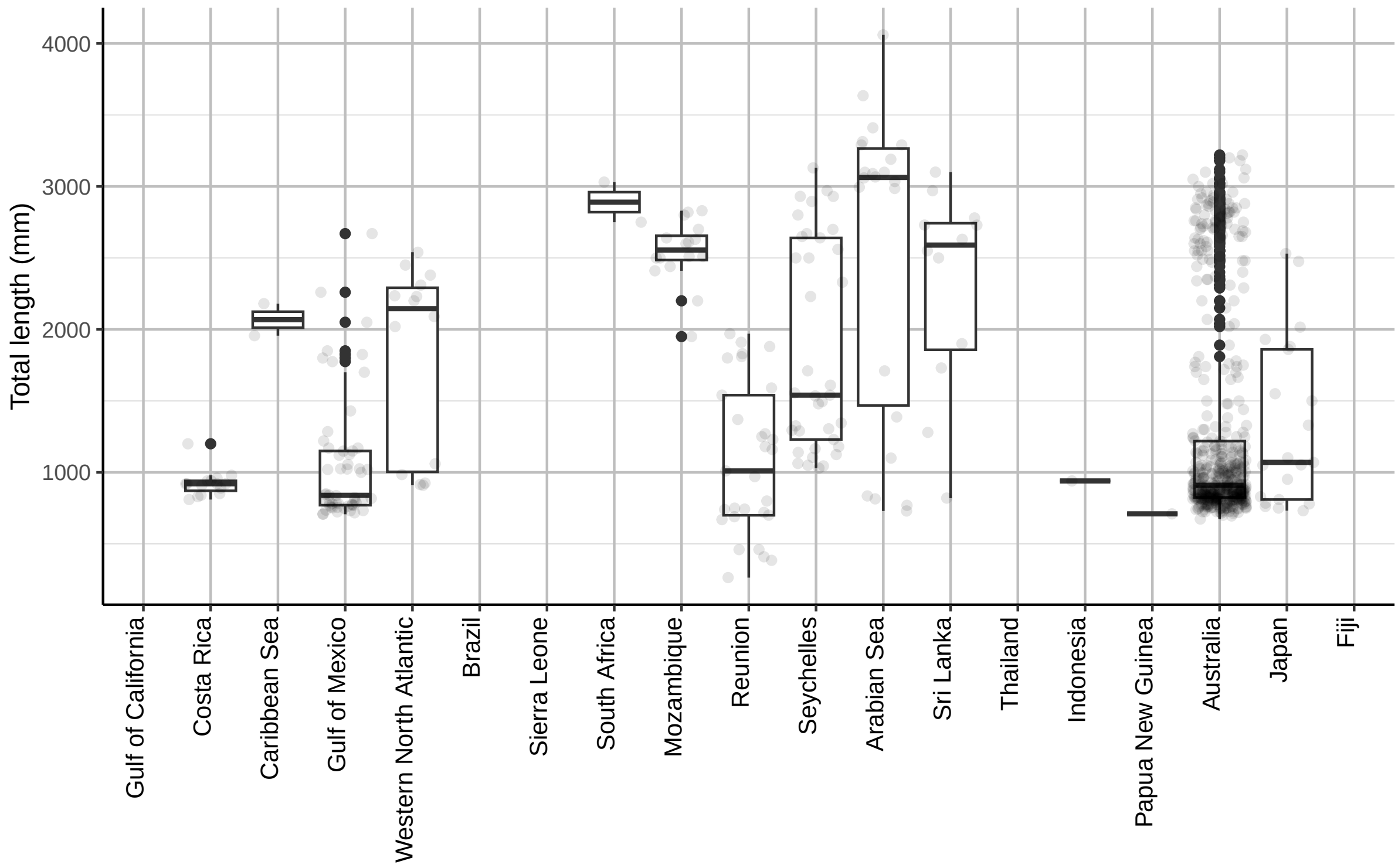

Aproximate Length-at-age

Based on length-at-age function from Tillet et al. (2011).

"Model-averaged growth parameters for these length-at-age data are theoretical asymptotic length (LN) (s.e.) = 3119 (9.803)mm and a growth coefficient (k)¼0.158 year-1 respectively. Age at maturity was

9.5 years and like pig-eye sharks, bull sharks are long-lived with a predicted longevity of .27 years (Table 2). Juvenile growth rate was 186 mm/year for the first five years and then slowed to 45 mm/year post-maturity."

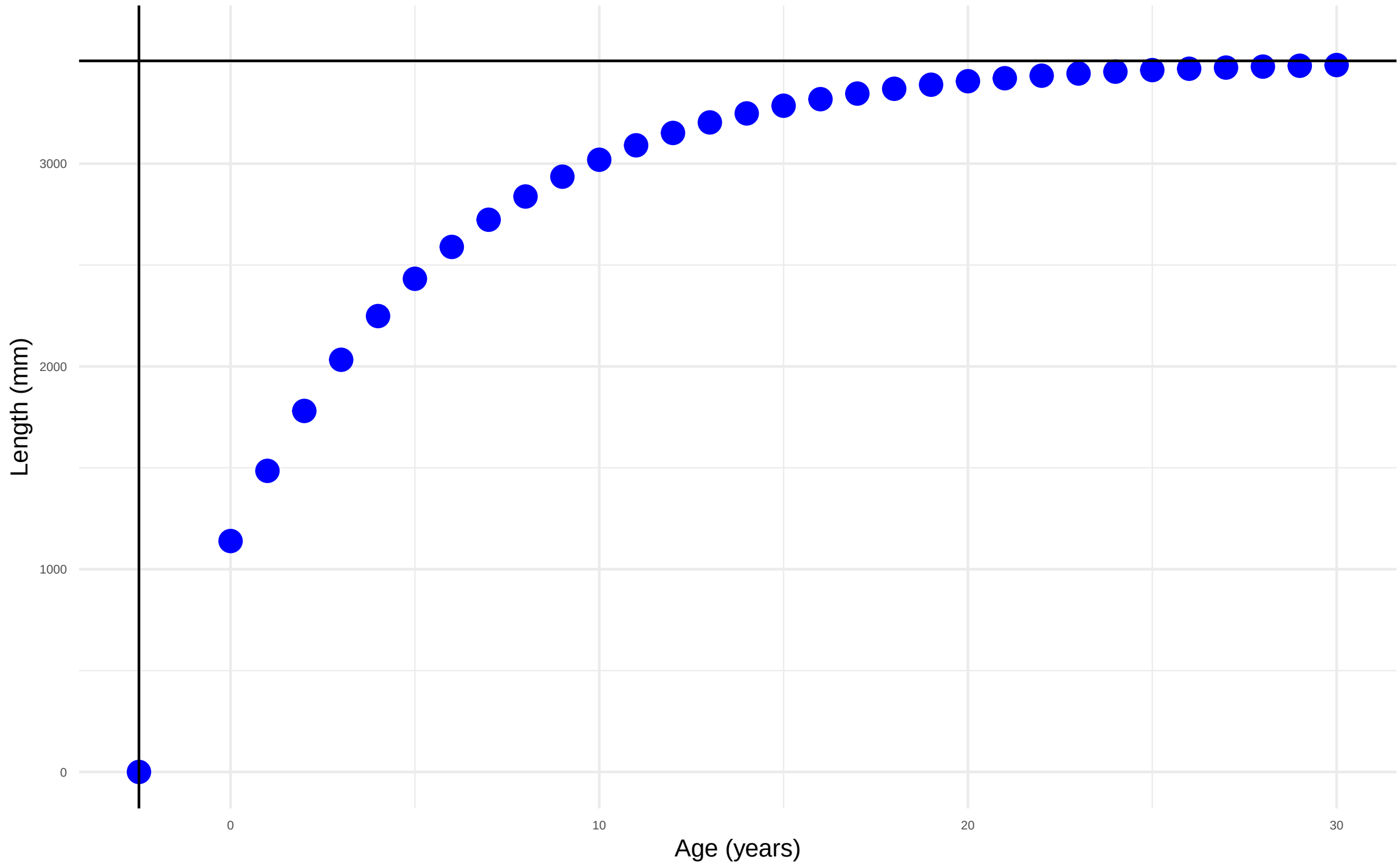

Catch date

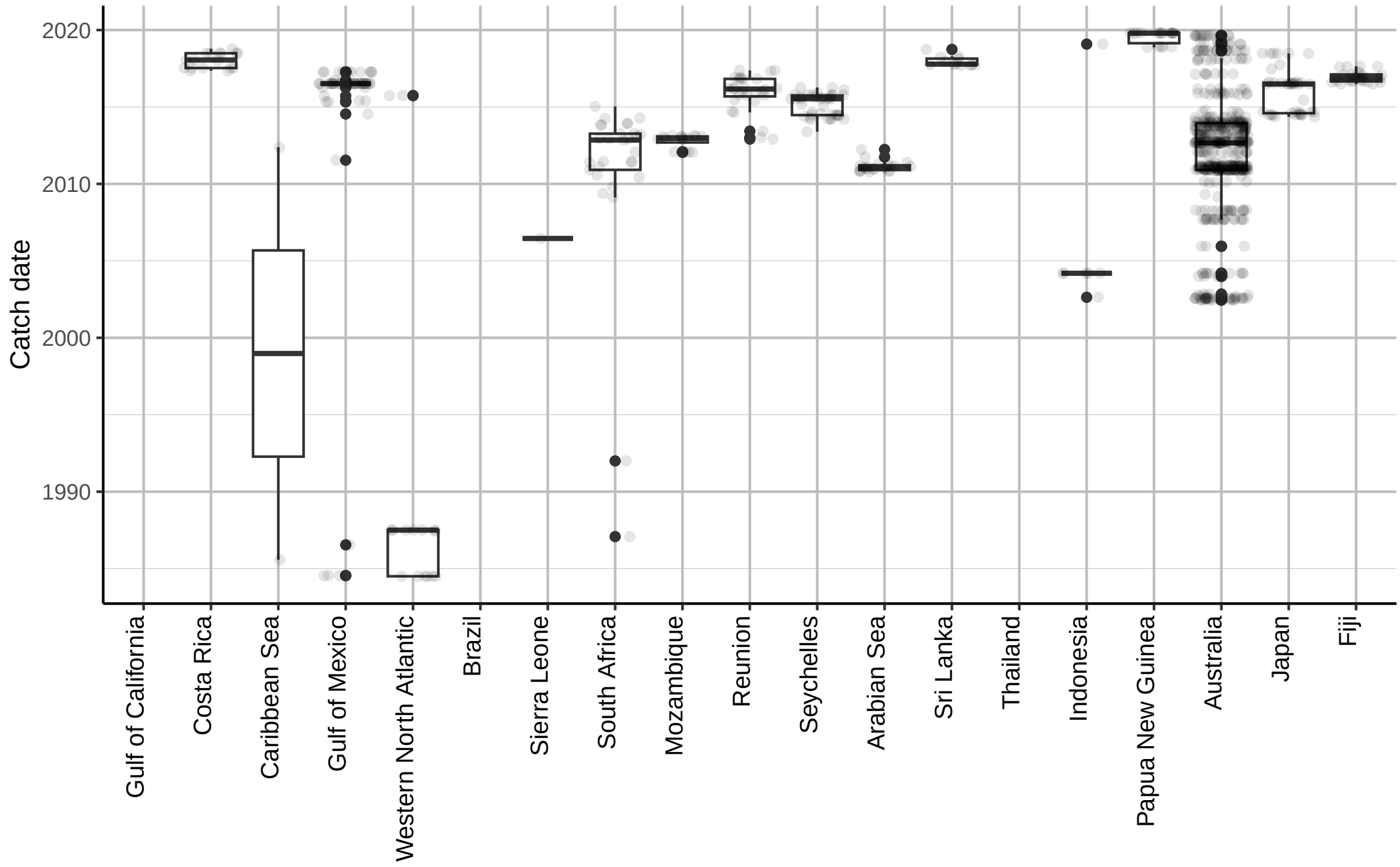

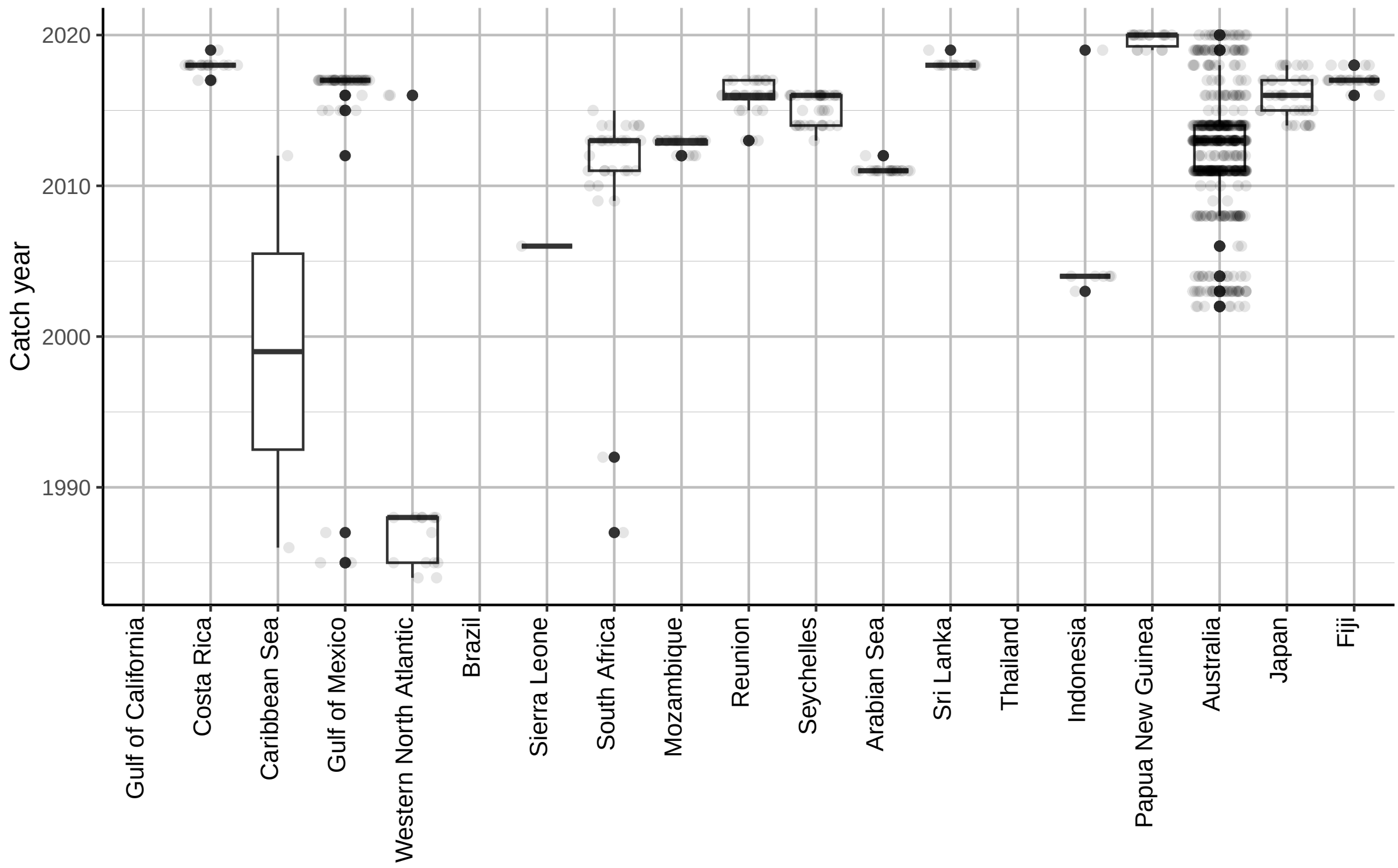

Age

```
## [1] 4060
```

```
## [1] 2
```

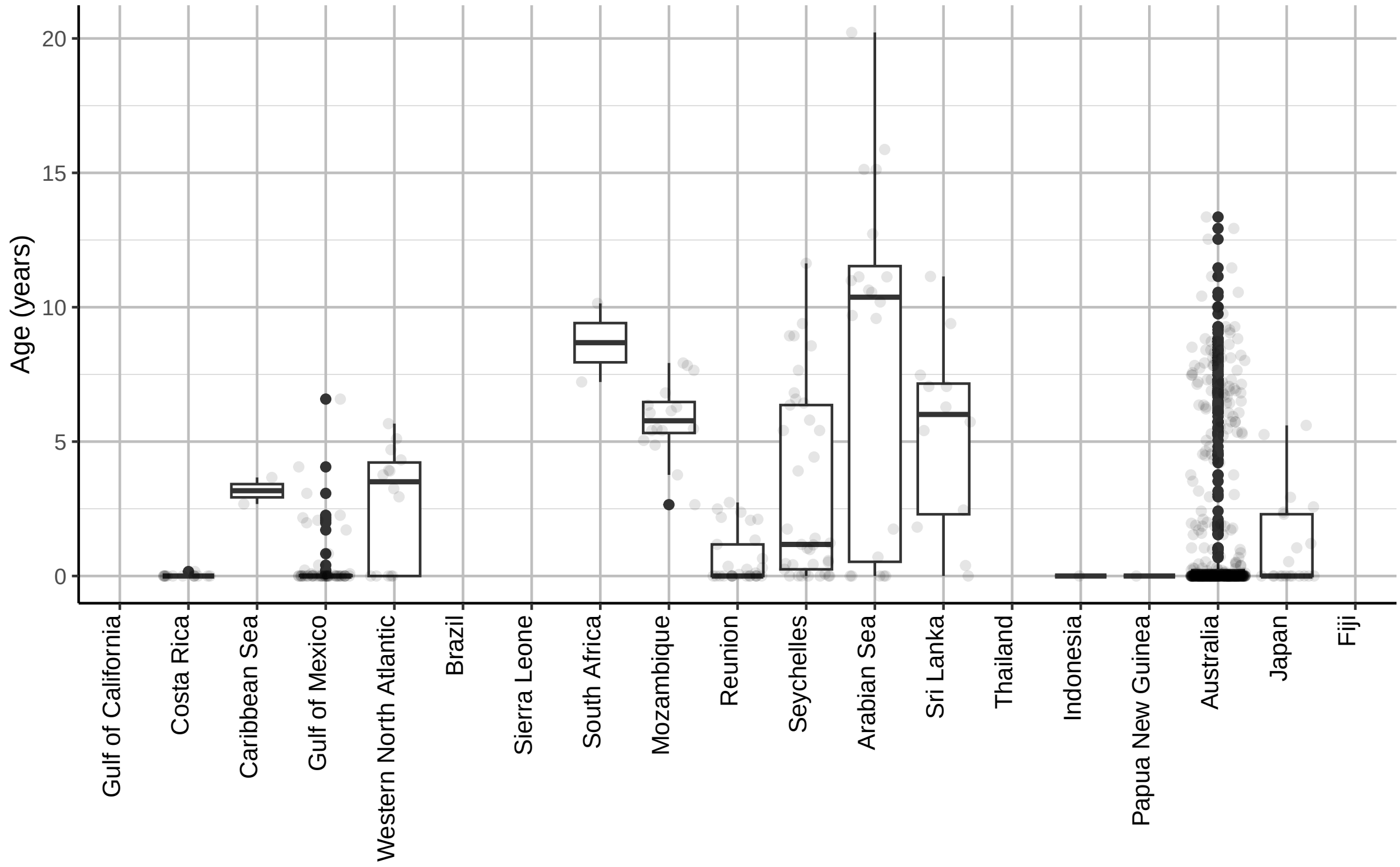

Age (years)
20
15
10
5
0
Gulf of California
Costa Rica
Caribbean Sea
Gulf of Mexico
Western North Atlantic
Brazil
Sierra Leone
South Africa
Mozambique
Reunion
Seychelles
Arabian Sea
Sri Lanka
Thailand
Indonesia
Papua New Guinea
Australia
Japan
Fiji

Cohort

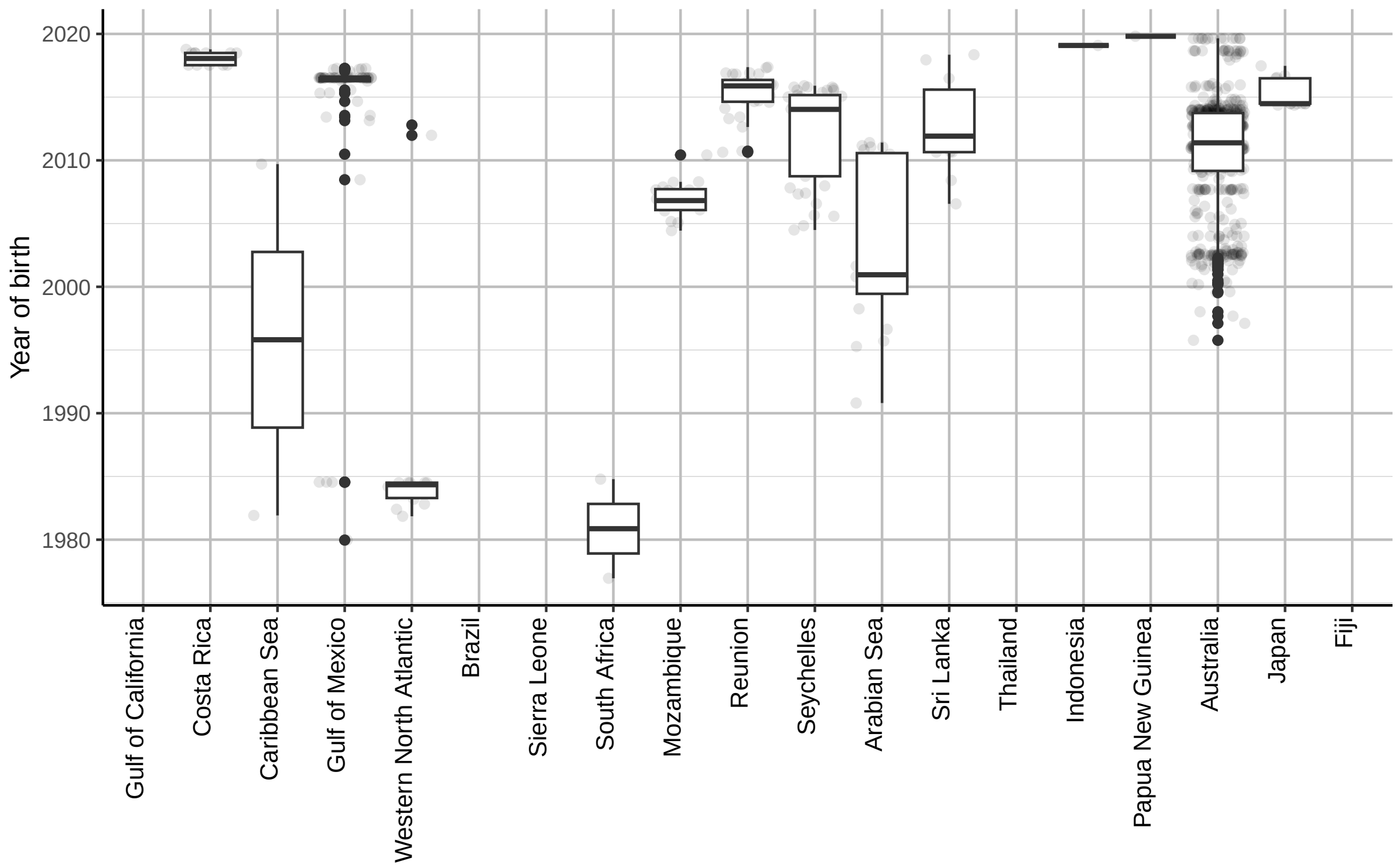

2020
2010
2000
1990
1980
Year of birth
Gulf of California
Costa Rica
Caribbean Sea
Gulf of Mexico
Western North Atlantic
Brazil
Sierra Leone
South Africa
Mozambique
Reunion
Seychelles
Arabian Sea
Sri Lanka
Thailand
Indonesia
Papua New Guinea
Australia
Japan
Fiji

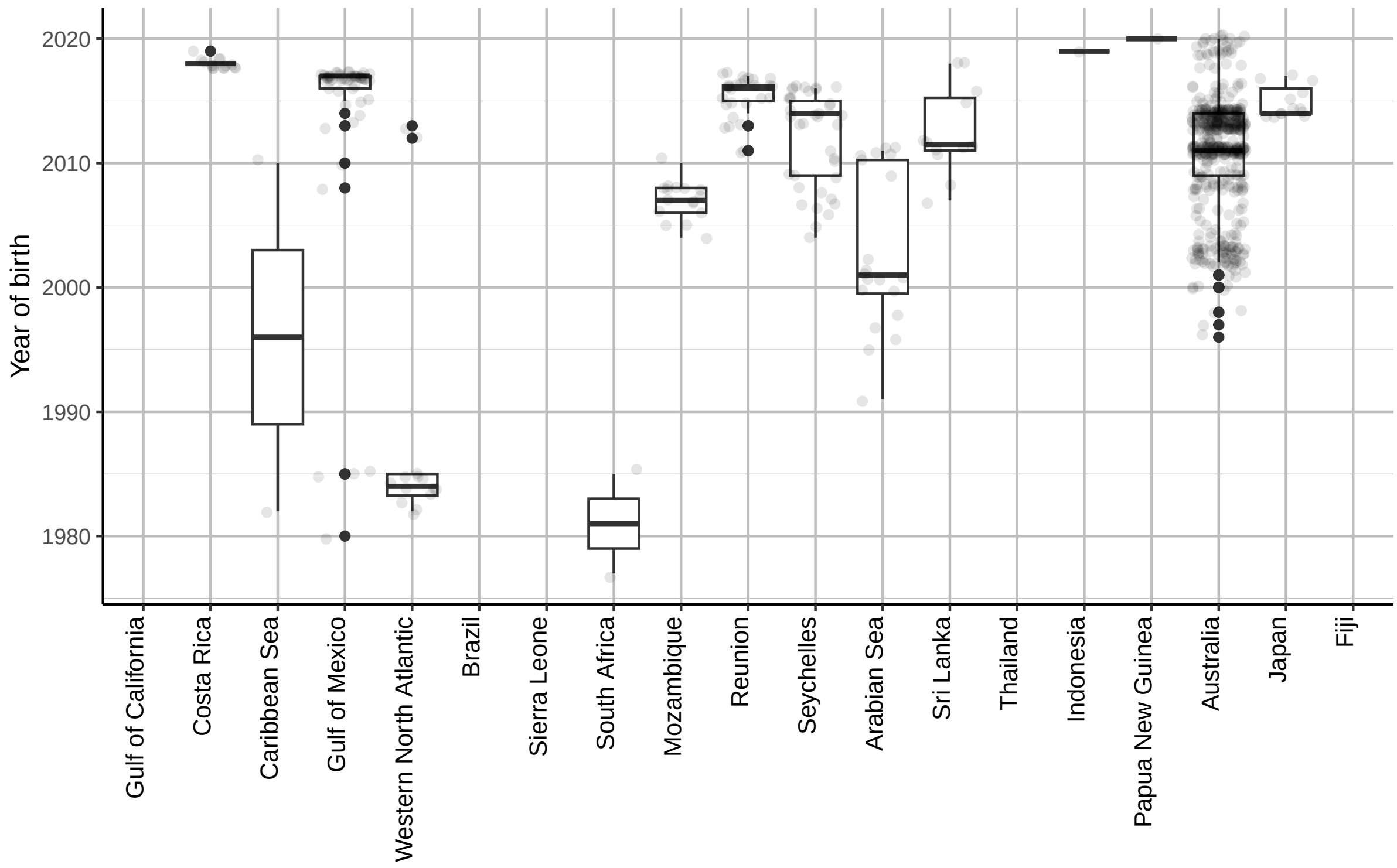

2020
2010
2000
1990
1980
Year of birth
Gulf of California
Costa Rica
Caribbean Sea
Gulf of Mexico
Western North Atlantic
Brazil
Sierra Leone
South Africa
Mozambique
Reunion
Seychelles
Arabian Sea
Sri Lanka
Thailand
Indonesia
Papua New Guinea
Australia
Japan
Fiji

DNA quality gels

DNA quality was checked on a 1 % agarose gel, stained with SYBR safe (Invitrogen, USA). The ladder was NEB TriDye™ 1 kb DNA Ladder, where to top ban represents 10Kb.

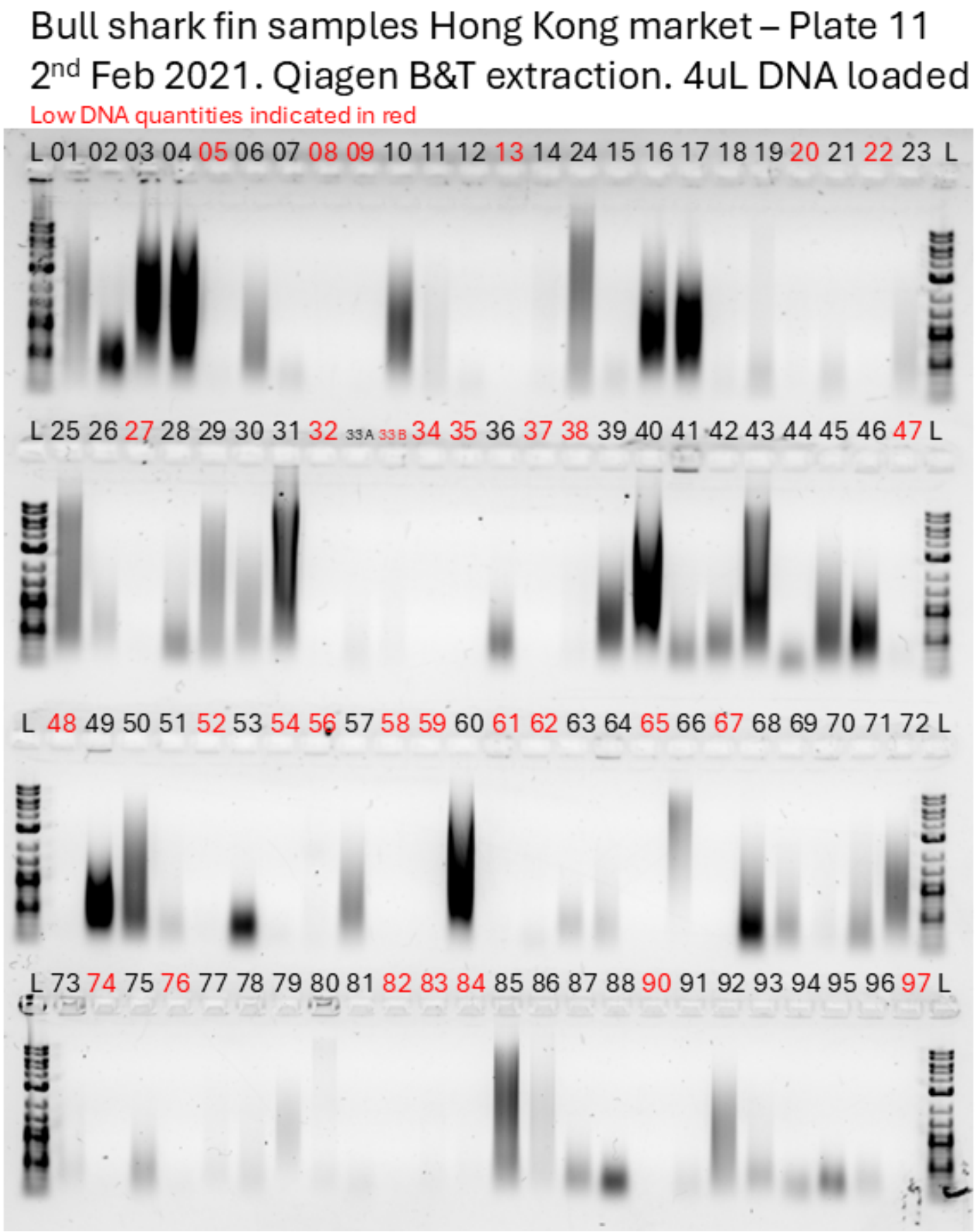

Bull shark fin samples Hong Kong market – Plate 12
2nd Feb 2021. Qiagen B&T extraction. 4uL DNA loaded
Low DNA quantities indicated in red

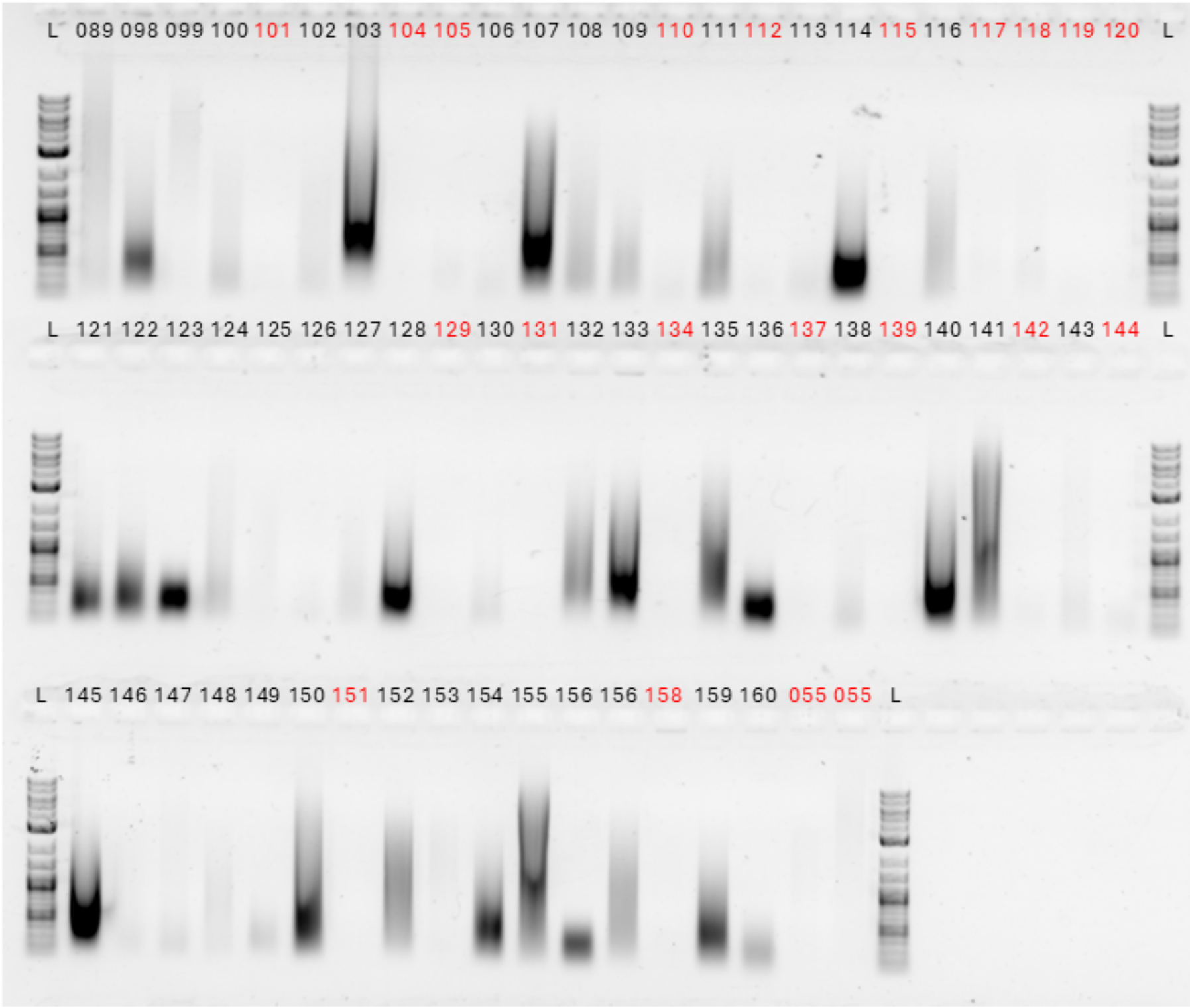

Bull shark fin samples Hong Kong market – Plate 11+12
6th Feb 2021. Dry elution volume from 100 -> 40 uL.
4uL DNA loaded
Low DNA quantities indicated in red

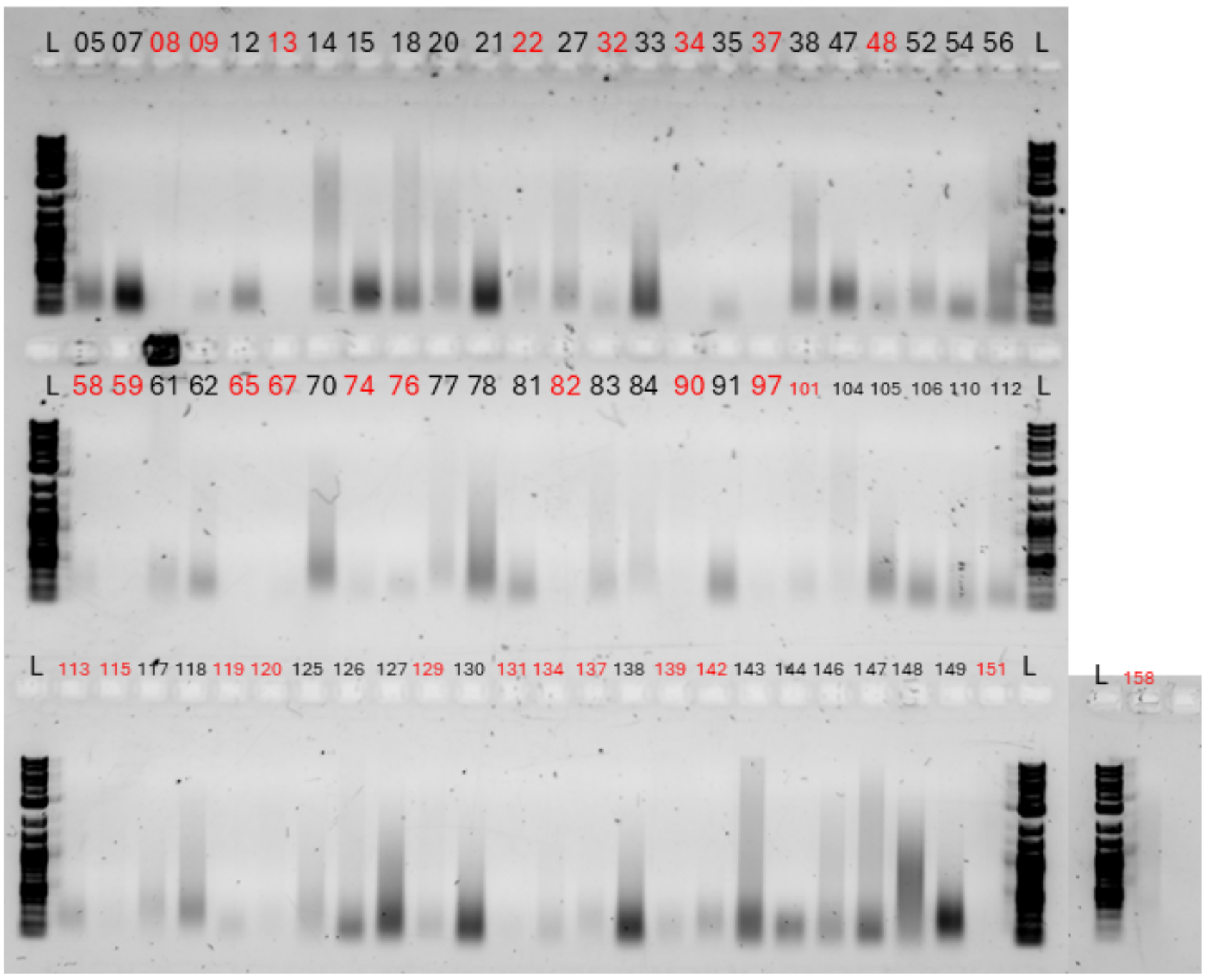

Bull shark fin samples Hong Kong market – Plate 11+12
7th Feb 2021. Re-extracted samples.
4uL DNA loaded

Low DNA quantities indicated in red

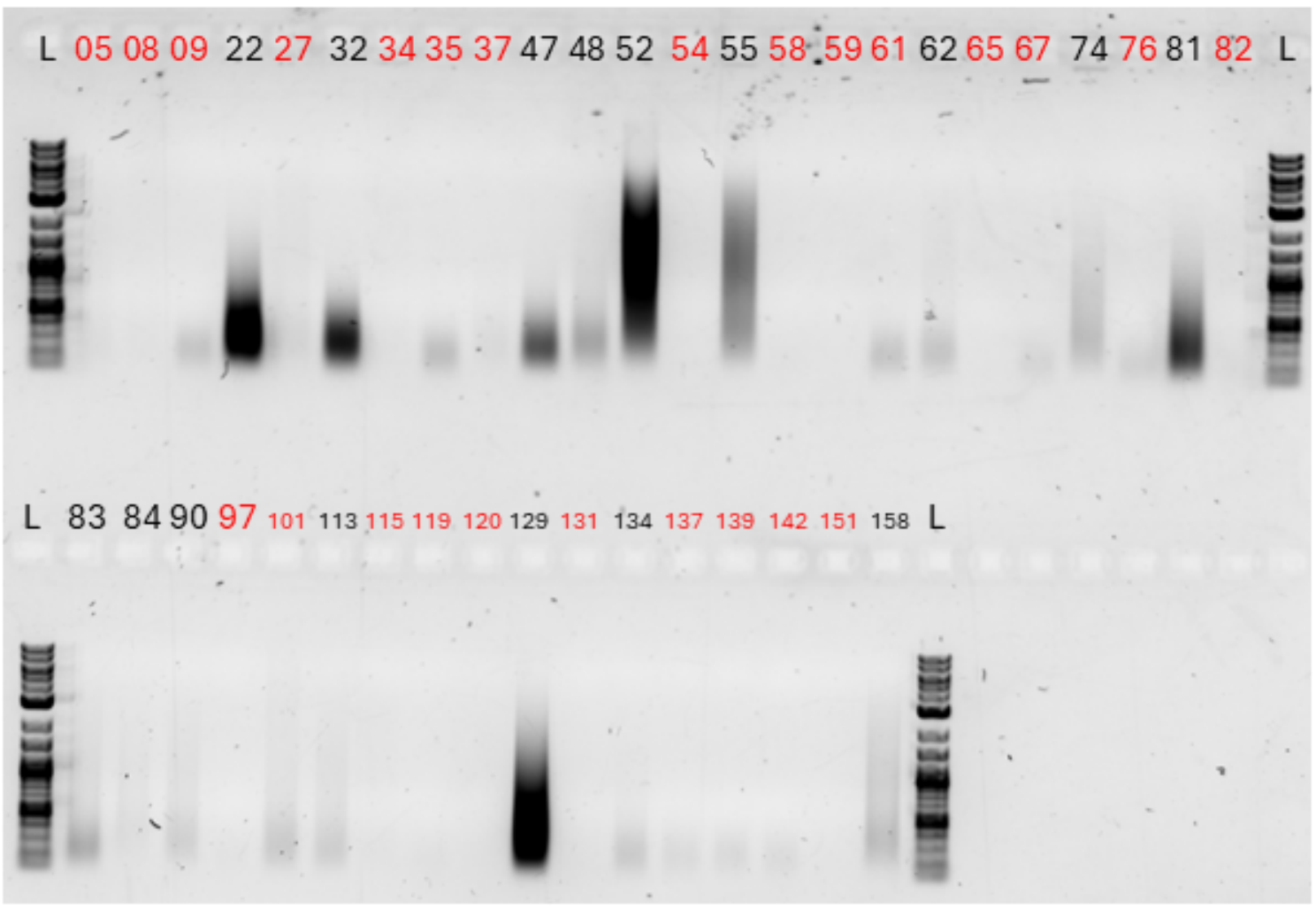

Load packages

```
library(radiator)
library(ggplot2)
library(dplyr)
library(tidyverse)
library(ade4)
library(adegenet)
library(ape)
library(apex)
library(assignPOP)
library(dartRverse)
library(fossil)
library(haplotypes)
library(data.table)
library(ggplot2)
library(grid)
library(gridExtra)
library(gtools)
library(kableExtra)
library(knitr)
library(marmap)
library(magrittr)
library(mvbutils)
library(pegas)
library(rubias)
library(strataG)
library(vegan)
source("Filtering funtions.R")


#set colours

colours.prior <- adegenet::funky(10)

colours.4 <- adegenet::funky(4)
names(colours.4) <- c("Brazil","Caribbean Sea","Gulf of Mexico",
                      "Western North Atlantic")

colours.6 <- adegenet::funky(6)
names(colours.6) <- c("E-PAC","W-ATL","E-ATL","IWP", "Japan","Fiji")

colours.8 <- adegenet::funky(8)
names(colours.8) <- c("E-PAC","E-ATL", "W-IO",  "N-IO", "E-IO",  "W-PAC",
                      "Japan", "Fiji")

colours.10 <- adegenet::funky(10)
names(colours.10) <- c("South Africa", "Mozambique", "Reunion" , "Seychelles",
                       "Arabian Sea", "Sri Lanka", "Thailand", "Indonesia",
                       "Papua New Guinea" , "Australia")

colours.14 <- adegenet::funky(14)
names(colours.14) <- c("Gulf of California","Costa Rica", "Sierra Leone", "South
                       Africa","Reunion","Seychelles","Arabian Sea","Sri Lanka",
                       "Thailand","Indonesia",
                       "Papua New Guinea","Australia","Japan","Fiji")
```

```r
colours.18 <- adegenet::funky(18)
names(colours.18) <- c("Costa Rica","Brazil", "Caribbean Sea", "Gulf of Mexico",
                       "Western North Atlantic",  "Sierra Leone", "South Africa",
                       "Mozambique", "Reunion","Seychelles","Arabian Sea","Sri Lanka",
                       "Thailand","Indonesia","Papua New Guinea","Australia","Japan",
                       "Fiji")

colours.20 <- adegenet::funky(20)
names(colours.20) <- c("Gulf of California","Costa Rica","Brazil", "Caribbean Sea",
                       "Gulf of Mexico", "Western North Atlantic",  "Sierra Leone",
                       "South Africa","Mozambique", "Reunion","Seychelles","Arabian Sea",
                       "Sri Lanka","Thailand","Indonesia","Papua New Guinea","Australia",
                       "Okinawa, Japan", "Urauchi River, Japan","Fiji")


colours.13 <- adegenet::funky(13)
names(colours.13) <- c("Fitzroy River","Victoria River","Daly River","Adelaide River",
                       "South Alligator River","East Alligator River","Blue mud Bay",
                       "Roper River","Towns River", "Wenlock River","Trinity inlet",
                       "Clarence River","Sydney Harbour")


colours.26 <- adegenet::funky(26)
names(colours.26) <- c("South Africa", "Mozambique", "Reunion" , "Seychelles",
                       "Arabian Sea", "Sri Lanka", "Thailand", "Indonesia",
                       "Papua New Guinea" , "Fitzroy River", "Daly River",
                       "Adelaide River","Darwin Coastal",  "South Alligator River",
                       "East Alligator River","Blue mud Bay", "Roper River","Towns River",
                       "Unknown","Wenlock River","Trinity inlet","Clarence River",
                       "Sydney Harbour","Okinawa", "Urauchi River", "Fiji")

colours.27 <- adegenet::funky(27)
names(colours.27) <- c("Gulf of California", "Costa Rica", "Sierra Leone",
                       "South Africa", "Reunion", "Seychelles", "Arabian Sea",
                       "Sri Lanka", "Thailand", "Indonesia", "Papua New Guinea",
                       "Fitzroy River", "Victoria River", "Daly River",
                       "Adelaide River", "South Alligator River",
                       "East Alligator River", "Blue mud Bay", "Roper River",
                       "Towns River", "Wenlock River", "Trinity inlet",
                       "Clarence River", "Sydney Harbour", "Okinawa",
                       "Urauchi River", "Fiji")

colours.29 <- adegenet::funky(29)
names(colours.29) <- c("Brazil", "Caribbean Sea", "Gulf of Mexico",
                       "Western North Atlantic", "Sierra Leone", "South Africa",
                       "Mozambique", "Reunion" , "Seychelles", "Arabian Sea",
                       "Sri Lanka", "Thailand", "Indonesia", "Papua New Guinea",
                       "Fitzroy River", "Daly River", "Adelaide River",
                       "South Alligator River", "East Alligator River",
                       "Blue mud Bay", "Roper River", "Towns River",
                       "Wenlock River", "Trinity inlet", "Clarence River",
                       "Sydney Harbour", "Okinawa", "Urauchi River", "Fiji")

colours.32 <- adegenet::funky(32)
names(colours.32) <- c("Costa Rica" , "Brazil", "Caribbean Sea",
                       "Gulf of Mexico", "Western North Atlantic",
                       "Sierra Leone", "South Africa", "Mozambique",
```

```
                        "Reunion" , "Seychelles", "Arabian Sea", "Sri Lanka",
                        "Thailand", "Indonesia", "Papua New Guinea",
                        "Fitzroy River", "Daly River", "Adelaide River",
                        "Darwin Coastal", "South Alligator River",
                        "East Alligator River", "Blue mud Bay", "Roper River",
                        "Towns River", "Unknown", "Wenlock River",
                        "Trinity inlet", "Clarence River", "Sydney Harbour",
                        "Okinawa", "Urauchi River", "Fiji")

colours.20B <- c(adegenet::funky(20), "black")
names(colours.20B) <- c("Gulf of California", "Costa Rica", "Brazil",
                         "Caribbean Sea", "Gulf of Mexico",
                         "Western North Atlantic", "Sierra Leone",
                         "South Africa", "Mozambique", "Reunion", "Seychelles",
                         "Arabian Sea", "Sri Lanka", "Thailand", "Indonesia",
                         "Papua New Guinea", "Australia", "Okinawa, Japan",
                         "Urauchi River, Japan", "Fiji", "Hong Kong fin trade")

colours.21 <- adegenet::funky(21)
names(colours.21) <- c("Gulf of California", "Costa Rica", "Brazil",
                        "Caribbean Sea", "Gulf of Mexico",
                        "Western North Atlantic", "Sierra Leone", "South Africa",
                        "Mozambique", "Reunion", "Seychelles", "Arabian Sea",
                        "Sri Lanka", "Thailand", "Indonesia", "Papua New Guinea",
                        "Australia", "Okinawa, Japan", "Urauchi River, Japan",
                        "Fiji", "Hong Kong fin trade")

colours.35 <- adegenet::funky(35)
names(colours.35) <- c("Gulf of California", "Costa Rica" , "Brazil",
                        "Caribbean Sea", "Gulf of Mexico",
                        "Western North Atlantic", "Sierra Leone", "South Africa",
                        "Mozambique", "Reunion" , "Seychelles", "Arabian Sea",
                        "Sri Lanka", "Thailand", "Indonesia", "Papua New Guinea",
                        "Fitzroy River", "Victoria River", "Daly River",
                        "Adelaide River", "Darwin Coastal",
                        "South Alligator River", "East Alligator River",
                        "Blue mud Bay", "Roper River", "Towns River", "Unknown",
                        "Wenlock River", "Trinity inlet", "Clarence River",
                        "Sydney Harbour", "Okinawa", "Urauchi River", "Fiji",
                        "Hong Kong fin trade")
```

DArTcap - All data

Set strata

twenty-three samples (22 HKG samples) failed library construction.

```
metafile <- "1.BullShark_AllSamples.csv"
data.file.snp <- "Bull_shark_HK_DArTcap_ALL.csv"
targetid <- readr::read_tsv("Bull_shark_HK_DArTcap_ALL_targetid.tsv")
targetid2 <- radiator::extract_dart_target_id(data.file.snp)

strata <- readr::read_csv(metafile) %>%
  dplyr::left_join(targetid, by = "INDIVIDUALS") %>%
  dplyr::filter(!is.na(TARGET_ID)) %>%
  dplyr::mutate(STRATA = Site2)
strata$INDIVIDUALS <- with(strata, make.unique(as.character(INDIVIDUALS)))
# readr::write_tsv(strata, file = "Bull_shark_HK_DArTcap_strata.tsv")
```

Identify sex-linked markers

```
data <- "Bull_shark_HK_DArTcap_ALL.csv"
silicodata <- "Bull_shark_HK_DArTcap_ALL_SILICO.csv"
strata <- radiator::read_strata("Bull_shark_HK_DArTcap_strata.tsv",
                                keep.two = FALSE)$strata
strata %<>% dplyr::rename(POP2 = "STRATA") %>%
  dplyr::rename(STRATA = "COMBINED_SEX")

bull_shark <- radiator::sexy_markers(
  data = data,
  silicodata = silicodata,
  strata = strata,
  filters = FALSE,
  interactive.filter = TRUE)
save(bull_shark, file = "Sex_results_high_filter.Rdata")
```

```
load("Sex_results_high_filter.Rdata")
knitr::kable(table(bull_shark$sexy.summary$MARKER_TYPE,
                   bull_shark$sexy.summary$METHOD)) %>%
  kableExtra::kable_styling(full_width = FALSE,font_size = 9)
```

	Coverage_method-SILICO	Coverage_method-SNP	heterozygosity_method-SNP
Homogametic_sex-marker	122	75	11

```
data <- "Bull_shark_HK_DArTcap_ALL_LowFilter.csv"
silicodata <- "Bull_shark_HK_DArTcap_ALL_LowFilter_SILICO.csv"
strata <- radiator::read_strata("Bull_shark_HK_DArTcap_strata.tsv",
                                keep.two = FALSE)$strata
strata %<>% dplyr::rename(POP2 = "STRATA") %>%
  dplyr::rename(STRATA = "COMBINED_SEX")

bull_shark <- radiator::sexy_markers(
  data = data,
  silicodata = silicodata,
```

```r
  strata = strata,
  filters = FALSE,
  interactive.filter = TRUE)
save(bull_shark, file = "Sex_results_low_filter.Rdata")
```

```r
load("Sex_results_low_filter.Rdata")
knitr::kable(table(bull_shark$sexy.summary$MARKER_TYPE,
                   bull_shark$sexy.summary$METHOD)) %>%
  kableExtra::kable_styling(full_width = FALSE,font_size = 9)
```

	Coverage_method-SILICO	Coverage_method-SNP	heterozygosity_method-SNP
Homogametic_sex-marker	177	136	12

Sex assignment

```r
data <- "Bull_shark_HK_DArTcap_ALL_LowFilter.csv"
strata <- "Bull_shark_HK_DArTcap_strata.tsv"
strata <- readr::read_tsv(strata)
gds <- radiator::read_dart(data = data,strata = strata, parallel.core = 17)
convo <- radiator::genomic_converter(gds,strata = strata, parallel.core = 17,
                                     output = c("genlight"))

gl <- convo$genlight
strata$id <- strata$INDIVIDUALS
strata$pop <- strata$Sex
strata <- strata[order(match(strata$id, gl$ind.names)),]
all(gl$ind.names == strata$id)
gl@other$ind.metrics <-  as.data.frame(strata)

# save(gl, file = "bull_shark_genlight_raw_data.Rdata")
```

```r
print(load("bull_shark_genlight_raw_data.Rdata"))
```

```
## [1] "gl"
```

```r
gl@other$ind.metrics <- as.data.frame(gl@other$ind.metrics)
gl@other$ind.metrics$Sex[gl@other$ind.metrics$Sex == "U"] <- NA
gl@other$ind.metrics$sex <- gl@other$ind.metrics$Sex
slm <- dartR.sexlinked::gl.keep.sexlinked(gl, system = "xy")
```

```
## Starting ::
##  Starting dartR.sexlinked
##  Starting gl.keep.sexlinked
##   Processing genlight object with SNP data
##   Multiple columns matched 'sex' (case-insensitive): Sex, sex. Using the first: 'Sex'.

## Completed: ::
##  Completed: dartR.sexlinked
##  Completed: gl.keep.sexlinked
```

```r
# wrongly identified marker:
slm$x.linked <- slm$x.linked[, locNames(slm$x.linked) != "CHROM_1__100063799__5"]

sex.id <- dartR.sexlinked::gl.infer.sex(slm, system = "xy")
# save(slm, sex.id, file = "bull_shark_genetic_sex_ID.Rdata")
```

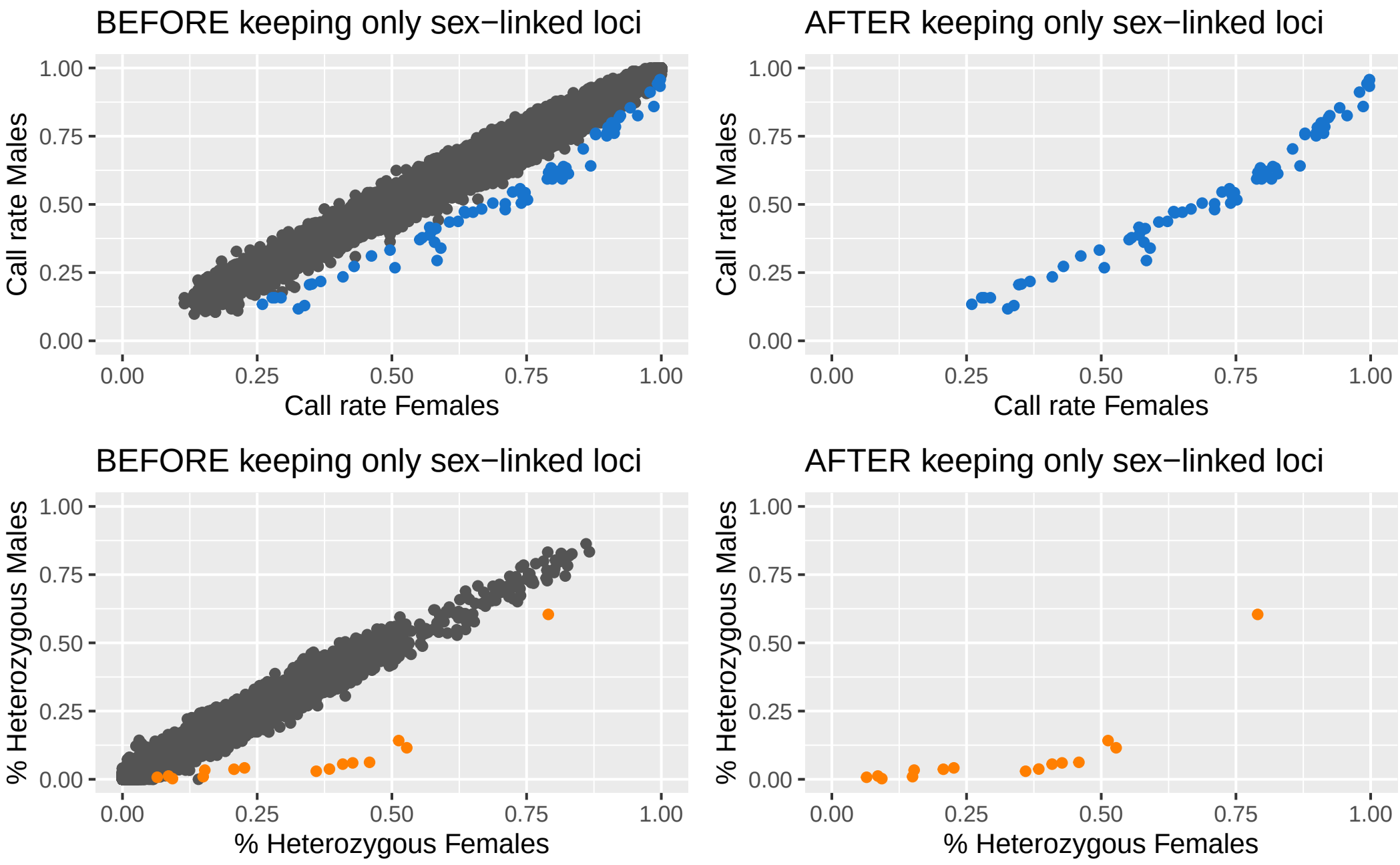


```r
print(load( "bull_shark_genetic_sex_ID.Rdata"))
```

```
## [1] "slm"     "sex.id"
```

```r
knitr::kable(table(gl@other$ind.metrics$sex, sex.id$x.linked.sex,
                   useNA = "always"))
```

	F	M	NA
F	342	93	0
M	40	378	0
NA	81	200	3

```r
#         genetic sex
# Visual
# sex
# |   |    F|    M| NA|
# |:--|---:|---:|--:|
# |F  | 342|   93|   0|
# |M  |  40| 378|   0|
# |NA |  81| 200|   3|

## Mis-assigned
# Genetic sex identification based on individuals of known sex (n=853)
# P(phenotypic M | genetic F) = 40/(342+40) = 10.5%
# P(phenotypic F | genetic M) = 93/(378+93 ) =19.7%
```

```r
adegenet::pop(gl) <- factor(gl@other$ind.metrics$STRATA)
HKG.sex.df <- sex.id[gl@pop == "Hong Kong fin trade",]
HKG.sex <- sex.id$x.linked.sex[gl@pop == "Hong Kong fin trade"]
knitr::kable(table(HKG.sex))
```

HKG.sex	Freq
F	49
M	102

```r
# |HKG.sex | Freq|
# |:-------|----:|
# |F       |   49|
# |M       |  102|

# minimum number of females(excludes females that could be misassigned)
# 49 - (49*0.105) = 43.855
# maximum number of females (includes males that could be misassigned)
# 49 + (102*0.197) = 69.094
# minimum number of males(excludes males that could be misassigned)
# 102 - (102*0.197) = 81.906
# maximum number of males (includes females that could be misassigned)
# 102 + (49*0.105) = 107.145

 #  F (10.5% error)  M (19.7% error)
 # 49[43.9-69.1]    102 [81.9-107.2]



## without technical replicates
# minimum number of females(excludes females that could be misassigned)
# 43 - (43*0.105) = 38.485
# maximum number of females (includes males that could be misassigned)
# 43 + (95*0.197) = 61.715
# minimum number of males(excludes males that could be misassigned)
# 95 - (95*0.197) = 76.285
# maximum number of males (includes females that could be misassigned)
# 95 + (43*0.105) = 99.515

 #  F (10.5% error)   M (19.7% error)
 # 43 [38.5-61.7]    95 [76.3-99.5]
# readr::write_csv(HKG.sex.df, file = "2.bull_shark_hong_kong_sex_ID.csv")
```

Filter data

1. Marker Reproducibility: 0.98
2. Individual missingness: 0.8
3. Individual Het - low: 0
4. Individual Het - high: 0.09
5. Individual total coverage - low: 2,000
6. Individual total coverage - high: 200,000
7. Marker MAC - low: 2
8. Marker coverage - low: 6
9. Marker coverage - high:62
10. Marker maximum missing proportion: 0.1
11. Marker snp position on locus: n
12. Max 1 SNPs per locus
13. 1 SNP per locus according to mac
14. no LD filter
15. Individual Het - low: n
16. Individual Het - high: n
17. Duplicated individuals: 0.007
18. Filter on HWE: 4 pop, p < 0.05

```
dartcap.data <- "Bull_shark_HK_DArTcap_ALL.csv"
strata <- "Bull_shark_HK_DArTcap_strata.tsv"
BSsnp <- radiator::filter_rad(dartcap.data, strata,
                              parallel.core = 1)
BS.dartcap.HK.gds <- BSsnp$gds
BS.dartcap.HK.tidy <- BSsnp$output$tidy.data
print(BS.dartcap.HK.gds, expand=TRUE, all=TRUE, attribute=TRUE,
      attribute.trim=FALSE)
save(BS.dartcap.HK.tidy, file = "Bull_shark_HK_DArTcap_ALL.Rdata")
```

FILTERS	VALUES	BEFORE	AFTER
Filter DArT reproducibility	0.98	1137 / 35 / 1 / 7458 / 8364	1137 / 35 / 1 / 6104 / 6713
Filter monomorphic markers	NA	1137 / 35 / 1 / 6104 / 6713	1137 / 35 / 1 / 6104 / 6713
Filter markers in common	NA	1137 / 35 / 1 / 6104 / 6713	1137 / 35 / 1 / 4548 / 4951
Filter individuals based on missingness	0.8	1137 / 35 / 1 / 4548 / 4951	1129 / 35 / 1 / 4548 / 4951
Filter individuals based on heterozygosity	0; 0.09	1129 / 35 / 1 / 4548 / 4951	1106 / 35 / 1 / 4548 / 4951
Filter individuals based on total coverage	2,000; 200,000	1106 / 35 / 1 / 4548 / 4951	1106 / 35 / 1 / 4548 / 4951
Filter monomorphic markers	NA	1106 / 35 / 1 / 4548 / 4951	1106 / 35 / 1 / 4090 / 4413
Filter MAC	2	1106 / 35 / 1 / 4090 / 4413	1106 / 35 / 1 / 3832 / 4117
Filter coverage min / max	6; 62	1106 / 35 / 1 / 3832 / 4117	1106 / 35 / 1 / 2597 / 2809
Filter genotyping	0.1	1106 / 35 / 1 / 2597 / 2809	1106 / 35 / 1 / 2374 / 2552
Filter SNPs position on the read	all	1106 / 35 / 1 / 2374 / 2552	1106 / 35 / 1 / 2374 / 2552
Filter markers snp number	1	1106 / 35 / 1 / 2374 / 2552	1106 / 35 / 1 / 2209 / 2209
Filter short ld	not filtering	1106 / 35 / 1 / 2209 / 2209	1106 / 35 / 1 / 2209 / 2209
detect mixed genomes	0 1	1106 / 35 / 1 / 2209 / 2209	1106 / 35 / 1 / 2209 / 2209
detect duplicate genomes	0.007	1106 / 35 / 1 / 2209 / 2209	1035 / 35 / 1 / 2209 / 2209
Filter monomorphic markers	NA	1035 / 35 / 1 / 2209 / 2209	1035 / 35 / 1 / 2209 / 2209
Filter HWE	4; 0.05	1035 / 35 / 1 / 2209 / 2209	1035 / 35 / 1 / 2142 / 2142

Save data

```
BSconvert <- radiator::genomic_converter(BS.dartcap.HK.tidy,
                                         output = c("genlight", "stockr", "pcadapt",
                                                    "gtypes", "rubias"))
BS.dartcap.HK.gl <- BSconvert$genlight
BS.dartcap.HK.gt <- BSconvert$gtypes
```

```r
BS.dartcap.HK.pcadapt <- BSconvert$pcadapt
BS.dartcap.HK.stockr <- BSconvert$stockr
BS.dartcap.HK.rubias <- BSconvert$rubias


markers.meta <- radiator::extract_markers_metadata(BS.dartcap.HK.gds, whitelist = TRUE)
individuals.meta <- radiator::extract_individuals_metadata(BS.dartcap.HK.gds,
                                                           whitelist = TRUE)
BS.dartcap.HK.gl$other$ind.metrics <- individuals.meta
BS.dartcap.HK.gl$other$loc.metrics <- markers.meta

pop.levels <- c("Gulf of California", "Costa Rica" , "Brazil","Caribbean Sea","Gulf of Mexico",
                "Western North Atlantic", "Sierra Leone", "South Africa", "Mozambique",
                "Reunion" , "Seychelles", "Arabian Sea", "Sri Lanka", "Thailand",
                "Indonesia","Papua New Guinea","Fitzroy River", "Victoria River",
                "Daly River","Adelaide River", "Darwin Coastal","South Alligator River",
                "East Alligator River","Blue mud Bay", "Roper River","Towns River",
                "Wenlock River","Trinity inlet","Clarence River", "Sydney Harbour",
                "Unknown","Okinawa","Urauchi River", "Fiji", "Hong Kong fin trade")
BS.dartcap.HK.gl$pop <- factor(BS.dartcap.HK.gl$other$ind.metrics$Site2,
                               levels = pop.levels)

save(BS.dartcap.HK.tidy, BS.dartcap.HK.gl, BS.dartcap.HK.gt, BS.dartcap.HK.pcadapt,
     BS.dartcap.HK.stockr, BS.dartcap.HK.rubias,BS.dartcap.HK.rubias2, markers.meta,
     individuals.meta,
     file = "Bull_shark_HK_DArTcap_ALL_objects.Rdata")
```

Load data

```r
load("Bull_shark_HK_DArTcap_ALL_objects.Rdata")
pop.levels <- c("Gulf of California", "Costa Rica" , "Brazil","Caribbean Sea",
                "Gulf of Mexico",  "Western North Atlantic", "Sierra Leone",
                "South Africa", "Mozambique", "Reunion" , "Seychelles", "Arabian Sea",
                "Sri Lanka", "Thailand", "Indonesia", "Papua New Guinea" , "Australia",
                "Okinawa, Japan","Urauchi River, Japan",
                "Fiji", "Hong Kong fin trade")

sites <- BS.dartcap.HK.gl$other$ind.metrics$Site
sites[BS.dartcap.HK.gl$other$ind.metrics$Site2 == "Okinawa"] <- "Okinawa, Japan"
sites[BS.dartcap.HK.gl$other$ind.metrics$Site2 == "Urauchi River"] <- "Urauchi River, Japan"

BS.dartcap.HK.gl$other$ind.metrics$Site4 <- factor(sites, levels = pop.levels)
BS.dartcap.HK.gl$pop <- factor(BS.dartcap.HK.gl$other$ind.metrics$Site4,
                               levels = pop.levels)


pop.levels2 <- c("E-PAC","W-ATL", "E-ATL", "IWP", "Japan", "Fiji", "Hong Kong fin trade")
Region2 <- BS.dartcap.HK.gl$other$ind.metrics$Region
Region2[Region2 %in% c("E-IO","E-IO/W-PAC","N-IO","W-IO","W-PAC")] <- "IWP"
BS.dartcap.HK.gl$other$ind.metrics$Region2 <- factor(Region2,levels = pop.levels2)


BS.dartcap.HK.gl <- BS.dartcap.HK.gl[order(BS.dartcap.HK.gl$pop,
                                           BS.dartcap.HK.gl$ind.names)]

adegenet::nInd(BS.dartcap.HK.gl) # 1035
```

```
## [1] 1035
```

```r
adegenet::nLoc(BS.dartcap.HK.gl) # 2142
```

```
## [1] 2142
```

```r
knitr::kable(table(BS.dartcap.HK.gl$pop),
             caption = "Summary: Number of sharks by location")
```

Table 6: Summary: Number of sharks by location

Var1	Freq
Gulf of California	2
Costa Rica	16
Brazil	59
Caribbean Sea	9
Gulf of Mexico	50
Western North Atlantic	8
Sierra Leone	1
South Africa	28
Mozambique	13
Reunion	28
Seychelles	39
Arabian Sea	23
Sri Lanka	13
Thailand	7
Indonesia	6
Papua New Guinea	11
Australia	507
Okinawa, Japan	10
Urauchi River, Japan	39
Fiji	26
Hong Kong fin trade	140

```r
sum(grepl(pattern = "\\.1", x = BS.dartcap.HK.gl$ind.names)) #66
```

```
## [1] 66
```

```r
sum(duplicated(BS.dartcap.HK.gl$other$ind.metrics$`Genetic code`
               [BS.dartcap.HK.gl$other$ind.metrics$`Genetic code` != ""])) #25
```

```
## [1] 25
```

```r
ind.call.rate <- 1 - rowSums(is.na(as.matrix(BS.dartcap.HK.gl)))/nLoc(BS.dartcap.HK.gl)
ind.missing <-  rowSums(is.na(as.matrix(BS.dartcap.HK.gl)))/nLoc(BS.dartcap.HK.gl)
BS.dartcap.HK.gl@other$ind.metrics$CallRate <- ind.call.rate
BS.dartcap.HK.gl@other$ind.metrics$missingness <- ind.missing
```

FST

```
BS.dartcap.HK.gt.sub <- BS.dartcap.HK.gt
BS.dartcap.HK.gt.sub@schemes <- BS.dartcap.HK.gl@other$ind.metrics
pop.levels4 <- c("Gulf_of_California", "Costa_Rica", "Brazil","Caribbean_Sea",
                 "Gulf_of_Mexico", "Western_North_Atlantic", "Sierra_Leone",
                 "South_Africa", "Mozambique", "Reunion", "Seychelles",
                 "Arabian_Sea", "Sri_Lanka", "Thailand", "Indonesia", "Papua_New_Guinea" ,
                 "Fitzroy_River", "Victoria_River", "Daly_River", "Adelaide_River",
                 "Darwin_Coastal","South_Alligator_River", "East_Alligator_River",
                 "Blue_mud_Bay", "Roper_River","Towns_River", "Wenlock_River",
                 "Trinity_inlet","Clarence_River", "Sydney_Harbour", "Unknown",
                 "Okinawa","Urauchi_River", "Fiji", "Hong_Kong_fin_trade")
BS.dartcap.HK.gt.sub@data$stratum <- factor(BS.dartcap.HK.gt.sub@data$stratum,
                                            levels = pop.levels4)
BS.dartcap.HK.gt.sub <- BS.dartcap.HK.gt.sub[,,pop.levels4[pop.levels4 != "Hong_Kong_fin_trade"],
                                             drop = TRUE]
BS.dartcap.HK.gt.sub@data <- BS.dartcap.HK.gt.sub@data[order(
  BS.dartcap.HK.gt.sub@data$stratum, BS.dartcap.HK.gt.sub@data$id),]
BS.dartcap.HK.gt.sub@data$stratum <- as.character(BS.dartcap.HK.gt.sub@data$stratum)
```

```
pwFST <- strataG::pairwiseTest(BS.dartcap.HK.gt.sub, nrep = 1000,
                               by.locus = FALSE, hap.locus = 1,
                               quietly = FALSE, stat = "Fst", max.cores = 3)
save(pwFST, file = "DArTcap_HK_FST_2.Rdata")
```

```
# load("DArTcap_HK_FST.Rdata")
load("DArTcap_HK_FST_2.Rdata")

pop.levels5 <- c("Gulf_of_California", "Costa_Rica", "Brazil","Caribbean_Sea",
                 "Gulf_of_Mexico", "Western_North_Atlantic", "Sierra_Leone",
                 "South_Africa", "Mozambique", "Reunion", "Seychelles", "Arabian_Sea",
                 "Sri_Lanka", "Thailand", "Indonesia", "Papua_New_Guinea" ,
                 "Fitzroy_River", "Victoria_River", "Daly_River", "Adelaide_River",
                 "Darwin_Coastal","South_Alligator_River", "East_Alligator_River","Blue_mud_Bay",
                 "Roper_River","Towns_River", "Wenlock_River","Trinity_inlet",
                 "Clarence_River", "Sydney_Harbour", "Unknown", "Okinawa",
                 "Urauchi_River", "Fiji")

pwres.Fst <- strataG::pairwiseMatrix(pwFST, stat = "Fst", locus = "All")

sumres.Fst <- pairwiseSummary(pwFST, locus = "All")
sumres.Fst$strata.1 <- factor(sumres.Fst$strata.1, levels = pop.levels5)
sumres.Fst$strata.2 <- factor(sumres.Fst$strata.2, levels = pop.levels5)
sumres.Fst <- sumres.Fst[order(sumres.Fst$strata.1, sumres.Fst$strata.2),]
# readr::write_csv(sumres.Fst, file = "DArTcap_HK_FST_summary.csv")


m2 <- sumres.Fst[order(match(sumres.Fst$strata.1,pop.levels5)),c(2,3,20)]
Gdf <- igraph::graph_from_data_frame(m2, directed = FALSE)
m2.1 <- igraph::as_adjacency_matrix(Gdf, names = TRUE, sparse = FALSE,
                                    attr = "Fst", type = "lower")
m2 <- sumres.Fst[order(match(sumres.Fst$strata.1,pop.levels5)),c(2,3,21)]
Gdf <- igraph::graph_from_data_frame(m2, directed = FALSE)
m2.2 <- igraph::as_adjacency_matrix(Gdf, names = TRUE, sparse = FALSE,
                                    attr = "Fst_p.val", type = "lower")
m2$Fst.p.adj <- p.adjust(m2$Fst_p.val, "bonferroni")
```

```r
Gdf <- igraph::graph_from_data_frame(m2, directed = FALSE)
m2.3 <- igraph::as_adjacency_matrix(Gdf, names = TRUE, sparse = FALSE,
                                    attr = "Fst.p.adj", type = "lower")

write.csv(m2.1, file = "DArTcap_HK_FST_Sites2.csv")
write.csv(m2.2, file = "DArTcap_HK_FST_Pval_Sites2.csv")
write.csv(m2.3, file = "DArTcap_HK_FST_Pval_bonferroni_Sites2.csv")


FST.table <- m2.1
colnames(FST.table) <- 1:34
rownames(FST.table) <- paste0(1:34,"_", rownames(FST.table))
knitr::kable(round(FST.table, digits = 1), padding = -1L) %>%
  kableExtra::kable_styling(full_width = FALSE,font_size = 6, latex_options = "scale_down")
```

	1	2	3	4	5	6	7	8	9	10	11	12	13	14	15	16	17	18	19	2
1_Gulf_of_California	0.0	0.0	0.0	0.0	0.0	0.0	0.0	0.0	0.0	0.0	0.0	0.0	0.0	0.0	0.0	0.0	0.0	0.0	0.0	0.
2_Costa_Rica	NA	0.0	0.0	0.0	0.0	0.0	0.0	0.0	0.0	0.0	0.0	0.0	0.0	0.0	0.0	0.0	0.0	0.0	0.0	0.
3_Brazil	0.2	0.2	0.0	0.0	0.0	0.0	0.0	0.0	0.0	0.0	0.0	0.0	0.0	0.0	0.0	0.0	0.0	0.0	0.0	0.
4_Caribbean_Sea	0.2	0.2	0.0	0.0	0.0	0.0	0.0	0.0	0.0	0.0	0.0	0.0	0.0	0.0	0.0	0.0	0.0	0.0	0.0	0.
5_Gulf_of_Mexico	0.2	0.2	0.0	0.0	0.0	0.0	0.0	0.0	0.0	0.0	0.0	0.0	0.0	0.0	0.0	0.0	0.0	0.0	0.0	0.
6_Sierra_Leone	NA	NA	0.5	0.5	0.5	0.0	0.0	0.0	0.0	0.0	0.0	0.0	0.0	0.0	0.0	0.0	0.0	0.0	0.0	0.
7_South_Africa	0.5	0.5	0.5	0.5	0.5	0.1	0.0	0.0	0.0	0.0	0.0	0.0	0.0	0.0	0.0	0.0	0.0	0.0	0.0	0.
8_Mozambique	0.5	0.5	0.5	0.5	0.5	0.1	0.0	0.0	0.0	0.0	0.0	0.0	0.0	0.0	0.0	0.0	0.0	0.0	0.0	0.
9_Reunion	0.5	0.5	0.5	0.5	0.5	0.1	0.0	0.0	0.0	0.0	0.0	0.0	0.0	0.0	0.0	0.0	0.0	0.0	0.0	0.
10_Seychelles	0.5	0.5	0.5	0.5	0.5	0.1	0.0	0.0	0.0	0.0	0.0	0.0	0.0	0.0	0.0	0.0	0.0	0.0	0.0	0.
11_Arabian_Sea	0.5	0.5	0.5	0.5	0.5	0.1	0.0	0.0	0.0	0.0	0.0	0.0	0.0	0.0	0.0	0.0	0.0	0.0	0.0	0.
12_Sri_Lanka	0.5	0.5	0.5	0.5	0.5	0.1	0.0	0.0	0.0	0.0	0.0	0.0	0.0	0.0	0.0	0.0	0.0	0.0	0.0	0.
13_Thailand	0.5	0.5	0.5	0.5	0.5	0.1	0.0	0.0	0.0	0.0	0.0	0.0	0.0	0.0	0.0	0.0	0.0	0.0	0.0	0.
14_Indonesia	0.5	0.5	0.5	0.5	0.5	0.1	0.0	0.0	0.0	0.0	0.0	0.0	0.0	0.0	0.0	0.0	0.0	0.0	0.0	0.
15_Papua_New_Guinea	0.5	0.5	0.5	0.5	0.5	0.1	0.0	0.0	0.0	0.0	0.0	0.0	0.0	0.0	0.0	0.0	0.0	0.0	0.0	0.
16_Fitzroy_River	NA	0.5	0.5	0.5	0.5	NA	0.0	0.0	0.0	0.0	0.0	0.0	0.0	0.0	0.0	0.0	0.0	0.0	0.0	0.
17_Victoria_River	0.5	0.5	0.5	0.5	0.5	0.1	0.0	0.0	0.0	0.0	0.0	0.0	0.0	0.0	0.0	0.0	0.0	0.0	0.0	0.
18_Daly_River	0.5	0.5	0.5	0.5	0.5	0.1	0.0	0.0	0.0	0.0	0.0	0.0	0.0	0.0	0.0	0.0	0.0	0.0	0.0	0.
19_Adelaide_River	0.5	0.5	0.5	0.5	0.5	0.1	0.0	0.0	0.0	0.0	0.0	0.0	0.0	0.0	0.0	0.0	0.0	0.0	0.0	0.
20_Darwin_Coastal	0.5	0.5	0.5	0.5	0.5	0.1	0.0	0.0	0.0	0.0	0.0	0.0	0.0	0.0	0.0	0.0	0.0	0.0	0.0	0.
21_South_Alligator_River	0.5	0.5	0.5	0.5	0.5	0.1	0.0	0.0	0.0	0.0	0.0	0.0	0.0	0.0	0.0	0.0	0.0	0.0	0.0	0.
22_East_Alligator_River	0.5	0.5	0.5	0.5	0.5	0.1	0.0	0.0	0.0	0.0	0.0	0.0	0.0	0.0	0.0	0.0	0.0	0.0	0.0	0.
23_Blue_mud_Bay	0.5	0.5	0.5	0.5	0.5	0.1	0.0	0.0	0.0	0.0	0.0	0.0	0.0	0.0	0.0	0.0	0.0	0.0	0.0	0.
24_Roper_River	0.5	0.5	0.5	0.5	0.5	0.1	0.0	0.0	0.0	0.0	0.0	0.0	0.0	0.0	0.0	0.0	0.0	0.0	0.0	0.
25_Towns_River	0.5	0.5	0.5	0.5	0.5	0.1	0.0	0.0	0.0	0.0	0.0	0.0	0.0	0.0	0.0	0.0	0.0	0.0	0.0	0.
26_Wenlock_River	0.5	0.5	0.5	0.5	0.5	0.1	0.0	0.0	0.0	0.0	0.0	0.0	0.0	0.0	0.0	0.0	0.0	0.0	0.0	0.
27_Trinity_inlet	0.5	0.5	0.5	0.5	0.5	0.1	0.0	0.0	0.0	0.0	0.0	0.0	0.0	0.0	0.0	0.0	0.0	0.0	0.0	0.
28_Clarence_River	0.5	0.5	0.5	0.5	0.5	0.1	0.0	0.0	0.0	0.0	0.0	0.0	0.0	0.0	0.0	0.0	0.0	0.0	0.0	0.
29_Sydney_Harbour	0.5	0.5	0.5	0.5	0.5	0.1	0.0	0.0	0.0	0.0	0.0	0.0	0.0	0.0	0.0	0.0	0.0	0.0	0.0	0.
30_Unknown	0.5	0.5	0.5	0.5	0.5	0.1	0.0	0.0	0.0	0.0	0.0	0.0	0.0	0.0	0.0	0.0	0.0	0.0	0.0	0.
31_Okinawa	0.5	0.5	0.5	0.5	0.5	0.1	0.0	0.0	0.0	0.0	0.0	0.0	0.0	0.0	0.0	0.0	0.0	0.0	0.0	0.
32_Urauchi_River	0.5	0.5	0.5	0.5	0.5	0.1	0.0	0.0	0.0	0.0	0.0	0.0	0.0	0.0	0.0	0.0	0.0	0.0	0.0	0.
33_Fiji	0.5	0.5	0.5	0.5	0.5	0.1	0.0	0.0	0.0	0.0	0.0	0.0	0.0	0.0	0.0	0.0	0.0	0.0	0.0	0.
34_Western_North_Atlanti	|0.	|0.	|0.	|0.	|0.	|0.	|0.	|0.	|0.	|0.	|0.	|0.	|0.	|0.	|0.	|0.	|0.	|0.	|0.	|(

PCA

With HKG

```
pca1 <- adegenet::glPca(BS.dartcap.HK.gl, nf = 6, parallel = TRUE,
                        n.cores = parallel::detectCores() - 1)
save(pca1, file = "DArTcap_HK_PCA_2.Rdata")
```

```
load("DArTcap_HK_PCA_2.Rdata")

var_frac <- pca1$eig/sum(pca1$eig)
print(paste0("Total variance explained: ",signif(sum(var_frac[1:6]) * 100, 3), "%"))
```

```
## [1] "Total variance explained: 47.4%"
```

```
barplot(var_frac[1:20]*100, main = "variance explained",
        col = heat.colors(length(var_frac[1:20])))

pca1$scores <- pca1$scores[order(match(rownames(pca1$scores),
                                       BS.dartcap.HK.gl$ind.names)),]
########## PC1-2 ###################
pop <- BS.dartcap.HK.gl$pop
data <- data.frame(ind = rownames(pca1$scores), pop = pop, pca1$scores )
data <- merge(data, aggregate(cbind(mean.x = PC1, mean.y = PC2) ~
                                pop, data, mean), by = "pop")

CallRate <- BS.dartcap.HK.gl@other$ind.metrics$CallRate
CallRate <- CallRate[order(match(names(CallRate), data$ind))]
data$CallRate <- CallRate

missingness <- BS.dartcap.HK.gl@other$ind.metrics$missingness
missingness <- missingness[order(match(names(missingness), data$ind))]
data$missingness <- missingness

data1 <- data[!data$pop %in% "Hong Kong fin trade",]
data2 <- data[data$pop %in% "Hong Kong fin trade",]

pca.plot <- ggplot2::ggplot(data1,
                            ggplot2::aes(x = PC1, y = PC2, color = pop,
                                         size = missingness)) +
  ggplot2::geom_point(alpha = 0.9, shape = 1,
                      # size = 4,
                      ) +
  ggplot2::geom_point(ggplot2::aes(x = mean.x,
                                   y = mean.y, color = pop),
                      size = 4,
                      shape = 17,
                      alpha = 0.15) +
  ggplot2::geom_point(data = data2,
                      ggplot2::aes(x = PC1, y = PC2, color = pop,
                                   # size = CallRate
                                   size = missingness
                                   ),
                      alpha = 0.5, shape = 20) +
  ggplot2::scale_colour_manual(values = colours.20B) +
  ggplot2::scale_size(name = "Missing data (%)",
```

```r
                      breaks = c(0.1, 0.2,0.3,0.4,0.5,0.6, 0.7,0.8,0.9,1),
                      # limits = c(0,1),
                      range = c(1,6)) +
  # ggplot2::scale_fill_manual(values = colours.20B) +
  ggplot2::guides(col = guide_legend(ncol = 2)) +
  ggplot2::geom_hline(yintercept = 0) +
  ggplot2::geom_vline(xintercept = 0) +
  ggplot2::labs(
    subtitle = paste0("Total variance explained: ",
                      signif(sum(var_frac[1:2]) * 100, 3), "%"),
    y = paste0("PC2; ", signif(var_frac[2] * 100, digits = 2), " %"),
    x = paste0("PC1; ", signif(var_frac[1] * 100, digits = 2), " %"),
    title = "Bull shark data coloured according to sampling location",
    caption = "") +
  ggplot2::theme_minimal() +
  ggplot2::theme(
    axis.text = ggplot2::element_text(size = 15),
    axis.title.x = ggplot2::element_text(size = 20),
    axis.title.y = ggplot2::element_text(size = 20),
    legend.text = ggplot2::element_text(size = 7)
  )

print(pca.plot)
ggplot2::ggsave(pca.plot, bg = "white",
                filename =
                  # "DArTcap_HK_PCA1-2_2142SNPs.png",
                  "2.DArTcap_HK_PCA1-2_2142SNPs.png",
                width = 30, height = 15, units = "cm")



#### Zoom IWP ###
data.sub <- data[(data$PC1 > -5 & data$PC2 > -1),]

data1 <- data.sub[!data.sub$pop %in% "Hong Kong fin trade",]
data2 <- data.sub[data.sub$pop %in% "Hong Kong fin trade",]

pca.plot <- ggplot2::ggplot(data1,
                            ggplot2::aes(x = PC1, y = PC2, color = pop,
                                         size = missingness)) +
  ggplot2::geom_point(alpha = 0.9, shape = 1,
                      # size = 4,
                      ) +
  ggplot2::geom_point(ggplot2::aes(x = mean.x,
                                   y = mean.y, color = pop),
                      size = 4,
                      shape = 17,
                      alpha = 0.15) +
  ggplot2::geom_point(data = data2,
                      ggplot2::aes(x = PC1, y = PC2, color = pop,
                                   # size = CallRate
                                   size = missingness
                                   ),
                      alpha = 0.5, shape = 20) +
  ggplot2::scale_colour_manual(values = colours.20B) +
  ggplot2::scale_size(name = "Missing data (%)",
                      breaks = c(0.1, 0.2,0.3,0.4,0.5,0.6, 0.7,0.8,0.9,1),
```

```r
                      # limits = c(0,1),
                      range = c(1,6)) +
                      # range = c(2,12)) +
  # ggplot2::scale_fill_manual(values = colours.20B) +
  ggplot2::guides(col = guide_legend(ncol = 2)) +
  ggplot2::geom_hline(yintercept = 0) +
  ggplot2::geom_vline(xintercept = 0) +
  ggplot2::labs(
    subtitle = paste0("Total variance explained: ",
                      signif(sum(var_frac[1:2]) * 100, 3), "%"),
    y = paste0("PC2; ", signif(var_frac[2] * 100, digits = 2), " %"),
    x = paste0("PC1; ", signif(var_frac[1] * 100, digits = 2), " %"),
    title = "Bull shark (IWP zoom), coloured according to sampling location",
    caption = "") +
  ggplot2::theme_minimal() +
  ggplot2::theme(
    axis.text = ggplot2::element_text(size = 15),
    axis.title.x = ggplot2::element_text(size = 20),
    axis.title.y = ggplot2::element_text(size = 20),
    legend.text = ggplot2::element_text(size = 7)
  )

print(pca.plot)
ggplot2::ggsave(pca.plot, bg = "white",
                filename =
                "2.DArTcap_HK_PCA1-2_2142SNPs_zoomIWP.png",
                # "2.DArTcap_HK_PCA1-2_2142SNPs_zoomIWP_NEW.png",
                width = 30, height = 15, units = "cm")


########## PC1-3 ####################
pop <- BS.dartcap.HK.gl$pop
data <- data.frame(ind = rownames(pca1$scores), pop = pop, pca1$scores )
data <- merge(data, aggregate(cbind(mean.x = PC1, mean.y = PC3) ~
                                pop, data, mean), by = "pop")
CallRate <- BS.dartcap.HK.gl@other$ind.metrics$CallRate
CallRate <- CallRate[order(match(names(CallRate), data$ind))]
data$CallRate <- CallRate

missingness <- BS.dartcap.HK.gl@other$ind.metrics$missingness
missingness <- missingness[order(match(names(missingness), data$ind))]
data$missingness <- missingness

data1 <- data[!data$pop %in% "Hong Kong fin trade",]
data2 <- data[data$pop %in% "Hong Kong fin trade",]


pca.plot <- ggplot2::ggplot(data1,
                            ggplot2::aes(x = PC1, y = PC3, color = pop,
                                         size = missingness)) +
  ggplot2::geom_point(alpha = 0.9, shape = 1,
                      # size = 4,
                      ) +
  ggplot2::geom_point(ggplot2::aes(x = mean.x,
                                   y = mean.y, color = pop),
                      size = 4,
                      shape = 17,
```

```
                    alpha = 0.15) +
  ggplot2::geom_point(data = data2,
                    ggplot2::aes(x = PC1, y = PC3, color = pop,
                                 # size = CallRate
                                 size = missingness
                                 ),
                    alpha = 0.5, shape = 20) +
  ggplot2::scale_colour_manual(values = colours.20B) +
  ggplot2::scale_size(name = "Missing data (%)",
                    breaks = c(0.1, 0.2,0.3,0.4,0.5,0.6, 0.7,0.8,0.9,1),
                    # limits = c(0,1),
                    range = c(1,6)) +
                    # range = c(2,10)) +
  # ggplot2::scale_fill_manual(values = colours.20B) +
  ggplot2::guides(col = guide_legend(ncol = 2)) +
  ggplot2::geom_hline(yintercept = 0) +
  ggplot2::geom_vline(xintercept = 0) +
  ggplot2::labs(
    subtitle = paste0("Total variance explained: ",
                    signif(sum(var_frac[c(1,3)]) * 100, 3), "%"),
    y = paste0("PC3; ", signif(var_frac[3] * 100, digits = 2), " %"),
    x = paste0("PC1; ", signif(var_frac[1] * 100, digits = 2), " %"),
    title = "Bull shark data coloured according to sampling location",
    caption = "") +
  ggplot2::theme_minimal() +
  ggplot2::theme(
    axis.text = ggplot2::element_text(size = 15),
    axis.title.x = ggplot2::element_text(size = 20),
    axis.title.y = ggplot2::element_text(size = 20),
    legend.text = ggplot2::element_text(size = 7)
  )

print(pca.plot)
ggplot2::ggsave(pca.plot, bg = "white",
                filename =
                "2.DArTcap_HK_PCA1-3_2142SNPs.png",
                # "2.DArTcap_HK_PCA1-3_2142SNPs.png_NEW",
                width = 30, height = 15, units = "cm")



#### Zoom IWP ###
data.sub <- data[(data$PC1 > -5 & data$PC3 > -2.5),]

data1 <- data.sub[!data.sub$pop %in% "Hong Kong fin trade",]
data2 <- data.sub[data.sub$pop %in% "Hong Kong fin trade",]

pca.plot <- ggplot2::ggplot(data1,
                          ggplot2::aes(x = PC1, y = PC3, color =
                                         pop,size = missingness)) +
  ggplot2::geom_point(alpha = 0.9, shape = 1,
                    # size = 4,
                    ) +
  ggplot2::geom_point(ggplot2::aes(x = mean.x,
                                 y = mean.y, color = pop),
                    size = 4,
                    shape = 17,
```

```
                    alpha = 0.15) +
  ggplot2::geom_point(data = data2,
                    ggplot2::aes(x = PC1, y = PC3, color = pop,
                                 # size = CallRate
                                 size = missingness
                                 ),
                    alpha = 0.5, shape = 20) +
  ggplot2::scale_colour_manual(values = colours.20B) +
  ggplot2::scale_size(name = "Missing data (%)",
                    breaks = c(0.1, 0.2,0.3,0.4,0.5,0.6, 0.7,0.8,0.9,1),
                    # limits = c(0,1),
                    range = c(1,6)) +
  # ggplot2::scale_fill_manual(values = colours.20B) +
  ggplot2::guides(col = guide_legend(ncol = 2)) +
  ggplot2::geom_hline(yintercept = 0) +
  ggplot2::geom_vline(xintercept = 0) +
  ggplot2::labs(
    subtitle = paste0("Total variance explained: ",
                    signif(sum(var_frac[c(1,3)]) * 100, 3), "%"),
    y = paste0("PC3; ", signif(var_frac[3] * 100, digits = 2), " %"),
    x = paste0("PC1; ", signif(var_frac[1] * 100, digits = 2), " %"),
    title = "Bull shark (IWP zoom), coloured according to sampling location",
    caption = "") +
  ggplot2::theme_minimal() +
  ggplot2::theme(
    axis.text = ggplot2::element_text(size = 15),
    axis.title.x = ggplot2::element_text(size = 20),
    axis.title.y = ggplot2::element_text(size = 20),
    legend.text = ggplot2::element_text(size = 7)
  )

print(pca.plot)
ggplot2::ggsave(pca.plot, bg = "white",
                filename =
                "2.DArTcap_HK_PCA1-3_2142SNPs_zoomIWP.png",
                width = 30, height = 15, units = "cm")




########## PC3-4 ###################
pop <- BS.dartcap.HK.gl$pop
data <- data.frame(ind = rownames(pca1$scores), pop = pop, pca1$scores )
data <- merge(data, aggregate(cbind(mean.x = PC3, mean.y = PC4) ~
                                pop, data, mean), by = "pop")

CallRate <- BS.dartcap.HK.gl@other$ind.metrics$CallRate
CallRate <- CallRate[order(match(names(CallRate), data$ind))]
data$CallRate <- CallRate

missingness <- BS.dartcap.HK.gl@other$ind.metrics$missingness
missingness <- missingness[order(match(names(missingness), data$ind))]
data$missingness <- missingness

data1 <- data[!data$pop %in% "Hong Kong fin trade",]
data2 <- data[data$pop %in% "Hong Kong fin trade",]
```

```r
pca.plot <- ggplot2::ggplot(data1,
                            ggplot2::aes(x = PC3, y = PC4, color = pop,
                                         size = missingness)) +
  ggplot2::geom_point(alpha = 0.9, shape = 1,
                      # size = 4,
                      ) +
  ggplot2::geom_point(ggplot2::aes(x = mean.x,
                                   y = mean.y, color = pop),
                      size = 4,
                      shape = 17,
                      alpha = 0.15) +
  ggplot2::geom_point(data = data2,
                      ggplot2::aes(x = PC3, y = PC4, color = pop,
                                   # size = CallRate
                                   size = missingness
                                   ),
                      alpha = 0.5, shape = 20) +
  ggplot2::scale_colour_manual(values = colours.20B) +
  ggplot2::scale_size(name = "Missing data (%)",
                      breaks = c(0.1, 0.2,0.3,0.4,0.5,0.6, 0.7,0.8,0.9,1),
                      # limits = c(0,1),
                      range = c(1,6)) +
                      # range = c(2,10)) +
  # ggplot2::scale_fill_manual(values = colours.20B) +
  ggplot2::guides(col = guide_legend(ncol = 2)) +
  ggplot2::geom_hline(yintercept = 0) +
  ggplot2::geom_vline(xintercept = 0) +
  ggplot2::labs(
    subtitle = paste0("Total variance explained: ",
                      signif(sum(var_frac[c(3,4)]) * 100, 3), "%"),
    y = paste0("PC4; ", signif(var_frac[4] * 100, digits = 2), " %"),
    x = paste0("PC3; ", signif(var_frac[3] * 100, digits = 2), " %"),
    title = "Bull shark data coloured according to sampling location",
    caption = "") +
  ggplot2::theme_minimal() +
  ggplot2::theme(
    axis.text = ggplot2::element_text(size = 15),
    axis.title.x = ggplot2::element_text(size = 20),
    axis.title.y = ggplot2::element_text(size = 20),
    legend.text = ggplot2::element_text(size = 7)
  )

print(pca.plot)
ggplot2::ggsave(pca.plot, bg = "white",
                filename =
                "2.DArTcap_HK_PCA3-4_2142SNPs.png",
                width = 30, height = 15, units = "cm")




#### Zoom IWP ###
data.sub <- data[(data$PC3 > -4 & data$PC4 < 4),]

data1 <- data.sub[!data.sub$pop %in% "Hong Kong fin trade",]
data2 <- data.sub[data.sub$pop %in% "Hong Kong fin trade",]
```

```r
pca.plot <- ggplot2::ggplot(data1,
                            ggplot2::aes(x = PC3, y = PC4, color = pop,
                                         size = missingness)) +
  ggplot2::geom_point(alpha = 0.9, shape = 1,
                      # size = 4,
                      ) +
  ggplot2::geom_point(ggplot2::aes(x = mean.x,
                                   y = mean.y, color = pop),
                      size = 4,
                      shape = 17,
                      alpha = 0.15) +
  ggplot2::geom_point(data = data2,
                      ggplot2::aes(x = PC3, y = PC4, color = pop,
                                   # size = CallRate
                                   size = missingness
                                   ),
                      alpha = 0.5, shape = 20) +
  ggplot2::scale_colour_manual(values = colours.20B) +
  ggplot2::scale_size(name = "Missing data (%)",
                      breaks = c(0.1, 0.2,0.3,0.4,0.5,0.6, 0.7,0.8,0.9,1),
                      # limits = c(0,1),
                      range = c(1,6)) +
  # ggplot2::scale_fill_manual(values = colours.20B) +
  ggplot2::guides(col = guide_legend(ncol = 2)) +
  ggplot2::geom_hline(yintercept = 0) +
  ggplot2::geom_vline(xintercept = 0) +
  ggplot2::labs(
    subtitle = paste0("Total variance explained: ",
                      signif(sum(var_frac[c(3,4)]) * 100, 3), "%"),
    y = paste0("PC4; ", signif(var_frac[4] * 100, digits = 2), " %"),
    x = paste0("PC3; ", signif(var_frac[3] * 100, digits = 2), " %"),
    title = "Bull shark (IWP zoom), coloured according to sampling location",
    caption = "") +
  ggplot2::theme_minimal() +
  ggplot2::theme(
    axis.text = ggplot2::element_text(size = 15),
    axis.title.x = ggplot2::element_text(size = 20),
    axis.title.y = ggplot2::element_text(size = 20),
    legend.text = ggplot2::element_text(size = 7)
  )

print(pca.plot)
ggplot2::ggsave(pca.plot, bg = "white",
                filename =
                  # "DArTcap_HK_PCA3-4_2142SNPs_zoomIWP.png",
                "2.DArTcap_HK_PCA3-4_2142SNPs_zoomIWP.png",
                width = 30, height = 15, units = "cm")




########## PC4-5 ####################
pop <- BS.dartcap.HK.gl$pop
data <- data.frame(ind = rownames(pca1$scores), pop = pop, pca1$scores )
data <- merge(data, aggregate(cbind(mean.x = PC5, mean.y = PC4) ~
                                pop, data, mean), by = "pop")
```

```
CallRate <- BS.dartcap.HK.gl@other$ind.metrics$CallRate
CallRate <- CallRate[order(match(names(CallRate), data$ind))]
data$CallRate <- CallRate

missingness <- BS.dartcap.HK.gl@other$ind.metrics$missingness
missingness <- missingness[order(match(names(missingness), data$ind))]
data$missingness <- missingness

data1 <- data[!data$pop %in% "Hong Kong fin trade",]
data2 <- data[data$pop %in% "Hong Kong fin trade",]


pca.plot <- ggplot2::ggplot(data1,
                            ggplot2::aes(x = PC5, y = PC4, color = pop,
                                         size = missingness)) +
  ggplot2::geom_point(alpha = 0.9, shape = 1,
                      # size = 4,
                      ) +
  ggplot2::geom_point(ggplot2::aes(x = mean.x,
                                   y = mean.y, color = pop),
                      size = 4,
                      shape = 17,
                      alpha = 0.15) +
  ggplot2::geom_point(data = data2,
                      ggplot2::aes(x = PC5, y = PC4, color = pop,
                                   # size = CallRate
                                   size = missingness
                                   ),
                      alpha = 0.5, shape = 20) +
  ggplot2::scale_colour_manual(values = colours.20B) +
  ggplot2::scale_size(name = "Missing data (%)",
                      breaks = c(0.1, 0.2,0.3,0.4,0.5,0.6, 0.7,0.8,0.9,1),
                      # limits = c(0,1),
                      range = c(1,6)) +
                      # range = c(2,10)) +
  # ggplot2::scale_fill_manual(values = colours.20B) +
  ggplot2::guides(col = guide_legend(ncol = 2)) +
  ggplot2::geom_hline(yintercept = 0) +
  ggplot2::geom_vline(xintercept = 0) +
  ggplot2::labs(
    subtitle = paste0("Total variance explained: ",
                      signif(sum(var_frac[c(5,4)]) * 100, 3), "%"),
    y = paste0("PC4; ", signif(var_frac[4] * 100, digits = 2), " %"),
    x = paste0("PC5; ", signif(var_frac[5] * 100, digits = 2), " %"),
    title = "Bull shark coloured according to sampling location",
    caption = "") +
  ggplot2::theme_minimal() +
  ggplot2::theme(
    axis.text = ggplot2::element_text(size = 15),
    axis.title.x = ggplot2::element_text(size = 20),
    axis.title.y = ggplot2::element_text(size = 20),
    legend.text = ggplot2::element_text(size = 7)
  )

print(pca.plot)
ggplot2::ggsave(pca.plot, bg = "white",
                filename =
```

```
                  "2.DArTcap_HK_PCA4-5_2142SNPs.png",
                  # "2.DArTcap_HK_PCA4-5_2142SNPs_NEW.png",
                width = 30, height = 15, units = "cm")



########## PC4-6 ####################
pop <- BS.dartcap.HK.gl$pop
data <- data.frame(ind = rownames(pca1$scores), pop = pop, pca1$scores )
data <- merge(data, aggregate(cbind(mean.x = PC6, mean.y = PC4) ~
                                pop, data, mean), by = "pop")

CallRate <- BS.dartcap.HK.gl@other$ind.metrics$CallRate
CallRate <- CallRate[order(match(names(CallRate), data$ind))]
data$CallRate <- CallRate

missingness <- BS.dartcap.HK.gl@other$ind.metrics$missingness
missingness <- missingness[order(match(names(missingness), data$ind))]
data$missingness <- missingness

data1 <- data[!data$pop %in% "Hong Kong fin trade",]
data2 <- data[data$pop %in% "Hong Kong fin trade",]


pca.plot <- ggplot2::ggplot(data1,
                            ggplot2::aes(x = PC6, y = PC4, color = pop,
                                         size = missingness)) +
  ggplot2::geom_point(alpha = 0.9, shape = 1,
                      # size = 4,
                      ) +
  ggplot2::geom_point(ggplot2::aes(x = mean.x,
                                   y = mean.y, color = pop),
                      size = 4,
                      shape = 17,
                      alpha = 0.15) +
  ggplot2::geom_point(data = data2,
                      ggplot2::aes(x = PC6, y = PC4, color = pop,
                                   # size = CallRate
                                   size = missingness
                                   ),
                      alpha = 0.5, shape = 20) +
  ggplot2::scale_colour_manual(values = colours.20B) +
  ggplot2::scale_size(name = "Missing data (%)",
                      breaks = c(0.1, 0.2,0.3,0.4,0.5,0.6, 0.7,0.8,0.9,1),
                      # limits = c(0,1),
                      range = c(1,6)) +
                      # range = c(2,10)) +
  # ggplot2::scale_fill_manual(values = colours.20B) +
  ggplot2::guides(col = guide_legend(ncol = 2)) +
  ggplot2::geom_hline(yintercept = 0) +
  ggplot2::geom_vline(xintercept = 0) +
  ggplot2::labs(
    subtitle = paste0("Total variance explained: ",
                      signif(sum(var_frac[c(6,4)]) * 100, 3), "%"),
    y = paste0("PC4; ", signif(var_frac[4] * 100, digits = 2), " %"),
    x = paste0("PC6; ", signif(var_frac[6] * 100, digits = 2), " %"),
    title = "Bull shark coloured according to sampling location",
    caption = "") +
```

```r
  ggplot2::theme_minimal() +
  ggplot2::theme(
    axis.text = ggplot2::element_text(size = 15),
    axis.title.x = ggplot2::element_text(size = 20),
    axis.title.y = ggplot2::element_text(size = 20),
    legend.text = ggplot2::element_text(size = 7)
  )

print(pca.plot)
ggplot2::ggsave(pca.plot, bg = "white",
                filename =
                  "2.DArTcap_HK_PCA4-6_2142SNPs.png",
                  # "2.DArTcap_HK_PCA4-6_2142SNPs_NEW.png",
                width = 30, height = 15, units = "cm")




########## Only HK samples ###################
pca.HK <- pca1$scores[grepl("HK", rownames(pca1$scores)),]

pop <- BS.dartcap.HK.gl$pop
data <- data.frame(pca.HK, pop = "HK")
data <- merge(data, aggregate(cbind(mean.x = PC1, mean.y = PC2) ~
                                pop, data, mean), by = "pop")

pca.plot <- ggplot2::ggplot(data, ggplot2::aes(x = PC1, y = PC2)) +
  ggplot2::geom_point(size = 4, alpha = 0.85) +
  ggplot2::geom_point(ggplot2::aes(x = mean.x,
                                   y = mean.y),
                      size = 5,
                      shape = 17,
                      alpha = 0.15) +
  # ggplot2::scale_colour_manual(values = colours.20B) +
  # ggplot2::scale_fill_manual(values = colours.20B) +
  ggplot2::geom_hline(yintercept = 0) +
  ggplot2::geom_vline(xintercept = 0) +
  ggplot2::labs(
    subtitle = paste0("Total variance explained: ",
                      signif(sum(var_frac[c(1,2)]) * 100, 3), "%"),
    y = paste0("PC1; ", signif(var_frac[1] * 100, digits = 2), " %"),
    x = paste0("PC2; ", signif(var_frac[2] * 100, digits = 2), " %"),
    title = "Bull shark data coloured according to sampling location",
    caption = "") +
  ggplot2::theme_minimal() +
  ggplot2::theme(
    axis.text = ggplot2::element_text(size = 15),
    axis.title.x = ggplot2::element_text(size = 20),
    axis.title.y = ggplot2::element_text(size = 20),
    legend.text = ggplot2::element_text(size = 7)
  )

print(pca.plot)
ggplot2::ggsave(pca.plot,
                filename = "DArTcap_HK_PCA3-5_2142SNPs.png",
                width = 30, height = 15, units = "cm")
```

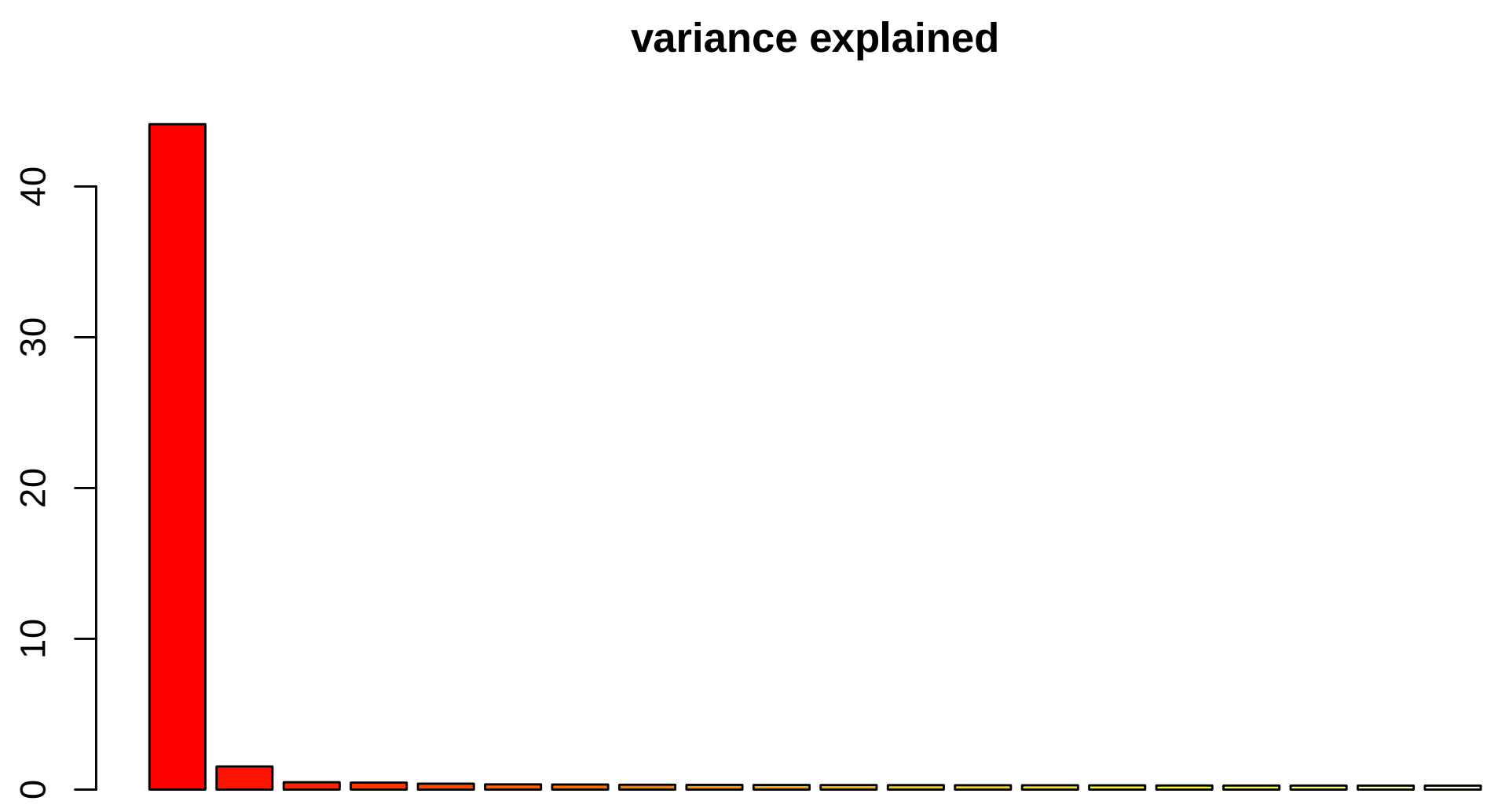
variance explained
0
10
20
30
40

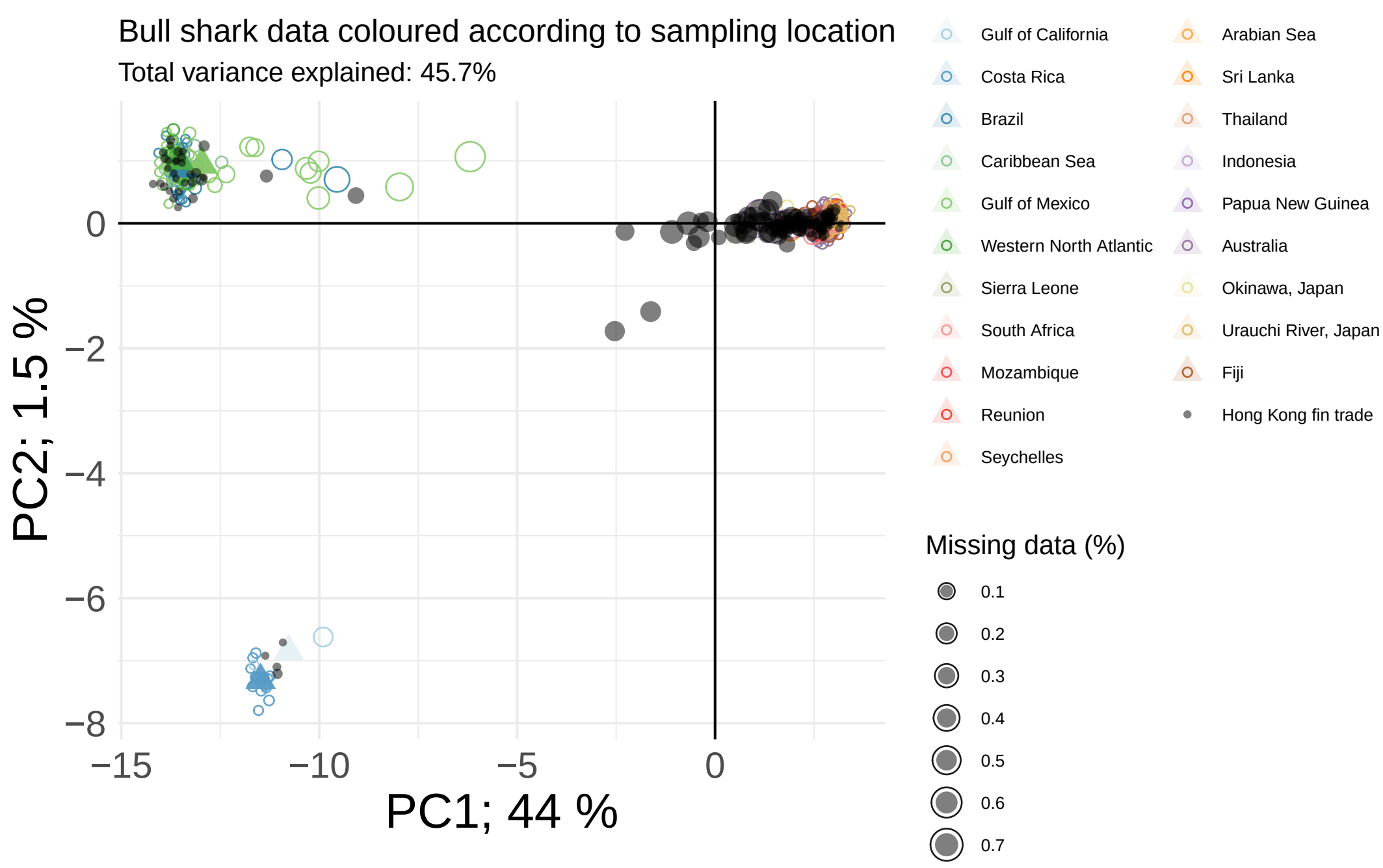
Bull shark data coloured according to sampling location
Total variance explained: 45.7%
PC2; 1.5 %
PC1; 44 %
0
−2
−4
−6
−8
−15
−10
−5
0
Gulf of California
Costa Rica
Brazil
Caribbean Sea
Gulf of Mexico
Western North Atlantic
Sierra Leone
South Africa
Mozambique
Reunion
Seychelles
Arabian Sea
Sri Lanka
Thailand
Indonesia
Papua New Guinea
Australia
Okinawa, Japan
Urauchi River, Japan
Fiji
Hong Kong fin trade
Missing data (%)
0.1
0.2
0.3
0.4
0.5
0.6
0.7

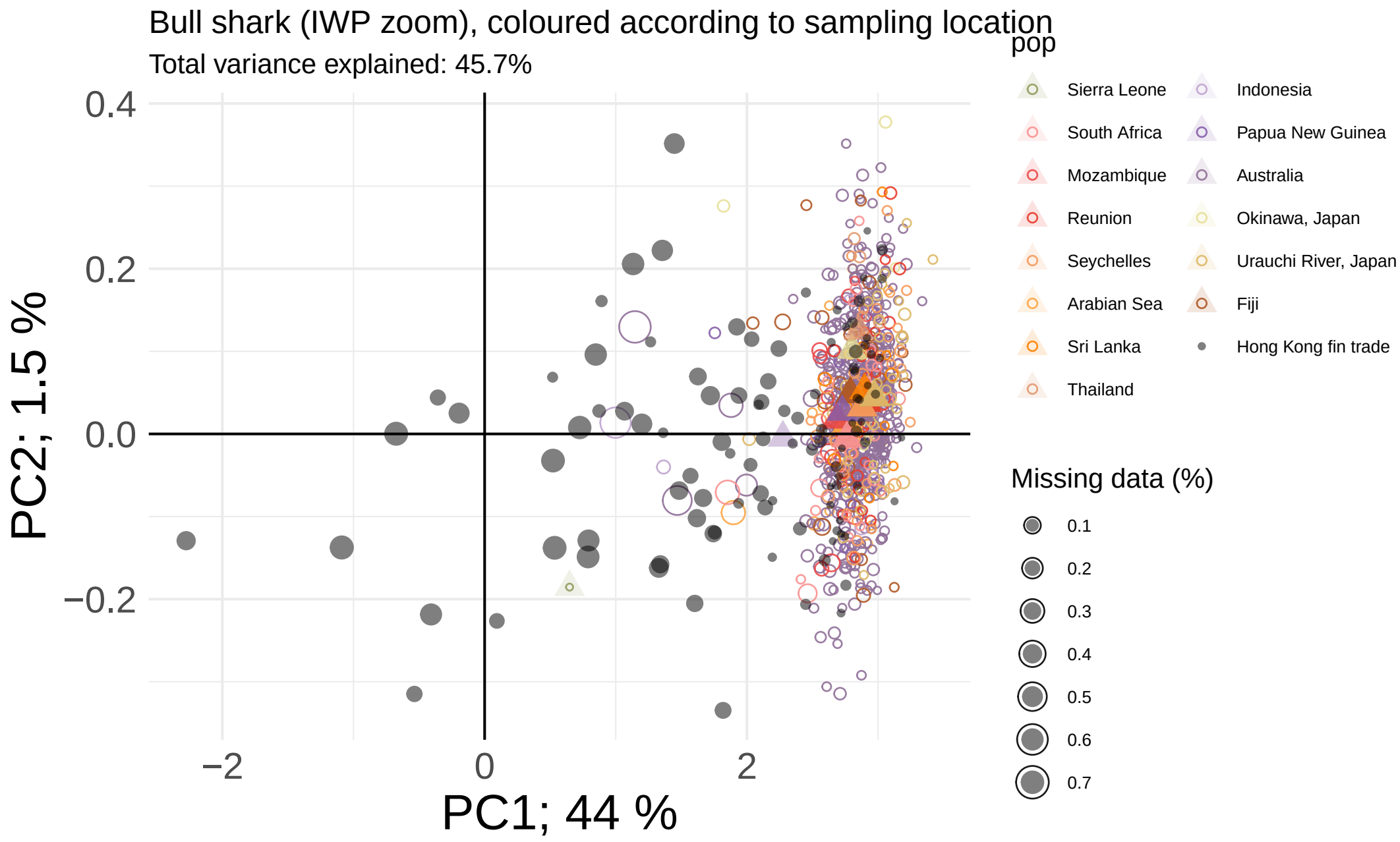
Bull shark (IWP zoom), coloured according to sampling location
Total variance explained: 45.7%
PC2; 1.5 %
PC1; 44 %
0.4
0.2
0.0
−0.2
−2
0
2
pop
Sierra Leone
South Africa
Mozambique
Reunion
Seychelles
Arabian Sea
Sri Lanka
Thailand
Indonesia
Papua New Guinea
Australia
Okinawa, Japan
Urauchi River, Japan
Fiji
Hong Kong fin trade
Missing data (%)
0.1
0.2
0.3
0.4
0.5
0.6
0.7

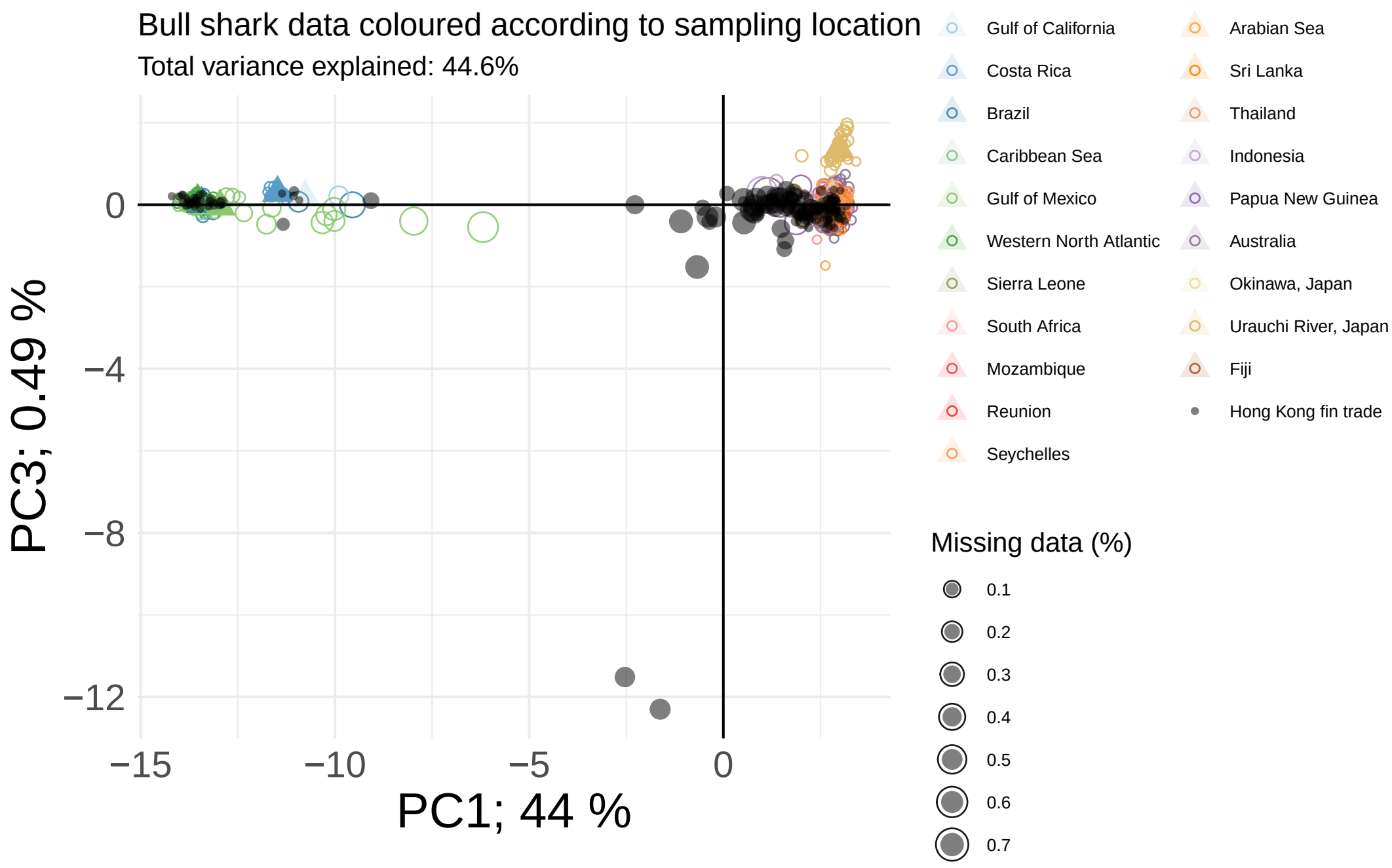
Bull shark data coloured according to sampling location
Total variance explained: 44.6%
PC3; 0.49 %
PC1; 44 %
0
−4
−8
−12
−15
−10
−5
0
Gulf of California
Costa Rica
Brazil
Caribbean Sea
Gulf of Mexico
Western North Atlantic
Sierra Leone
South Africa
Mozambique
Reunion
Seychelles
Arabian Sea
Sri Lanka
Thailand
Indonesia
Papua New Guinea
Australia
Okinawa, Japan
Urauchi River, Japan
Fiji
Hong Kong fin trade
Missing data (%)
0.1
0.2
0.3
0.4
0.5
0.6
0.7

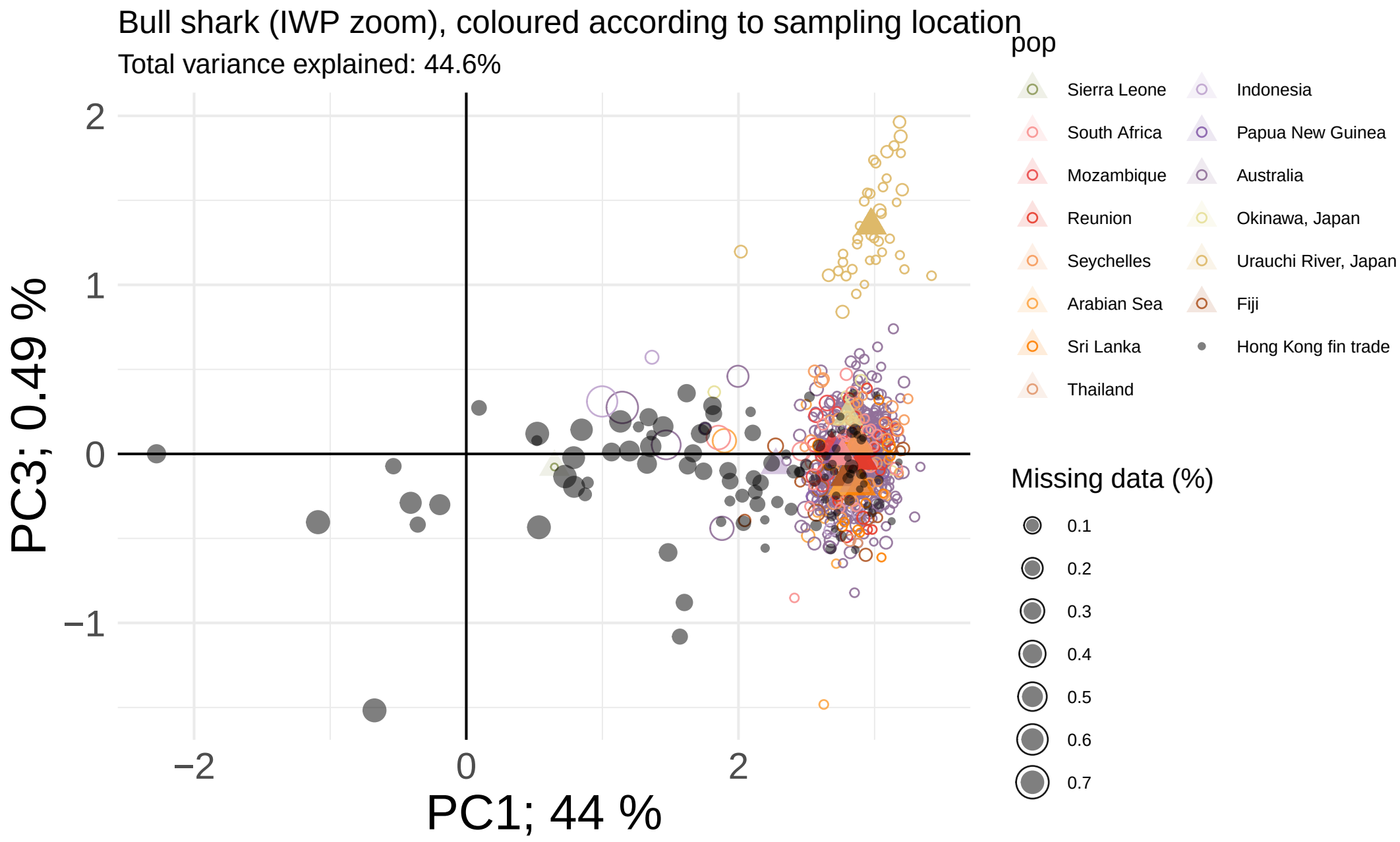

Bull shark (IWP zoom), coloured according to sampling location
Total variance explained: 44.6%
PC3; 0.49 %
PC1; 44 %
pop
Sierra Leone
South Africa
Mozambique
Reunion
Seychelles
Arabian Sea
Sri Lanka
Thailand
Indonesia
Papua New Guinea
Australia
Okinawa, Japan
Urauchi River, Japan
Fiji
Hong Kong fin trade
Missing data (%)
0.1
0.2
0.3
0.4
0.5
0.6
0.7

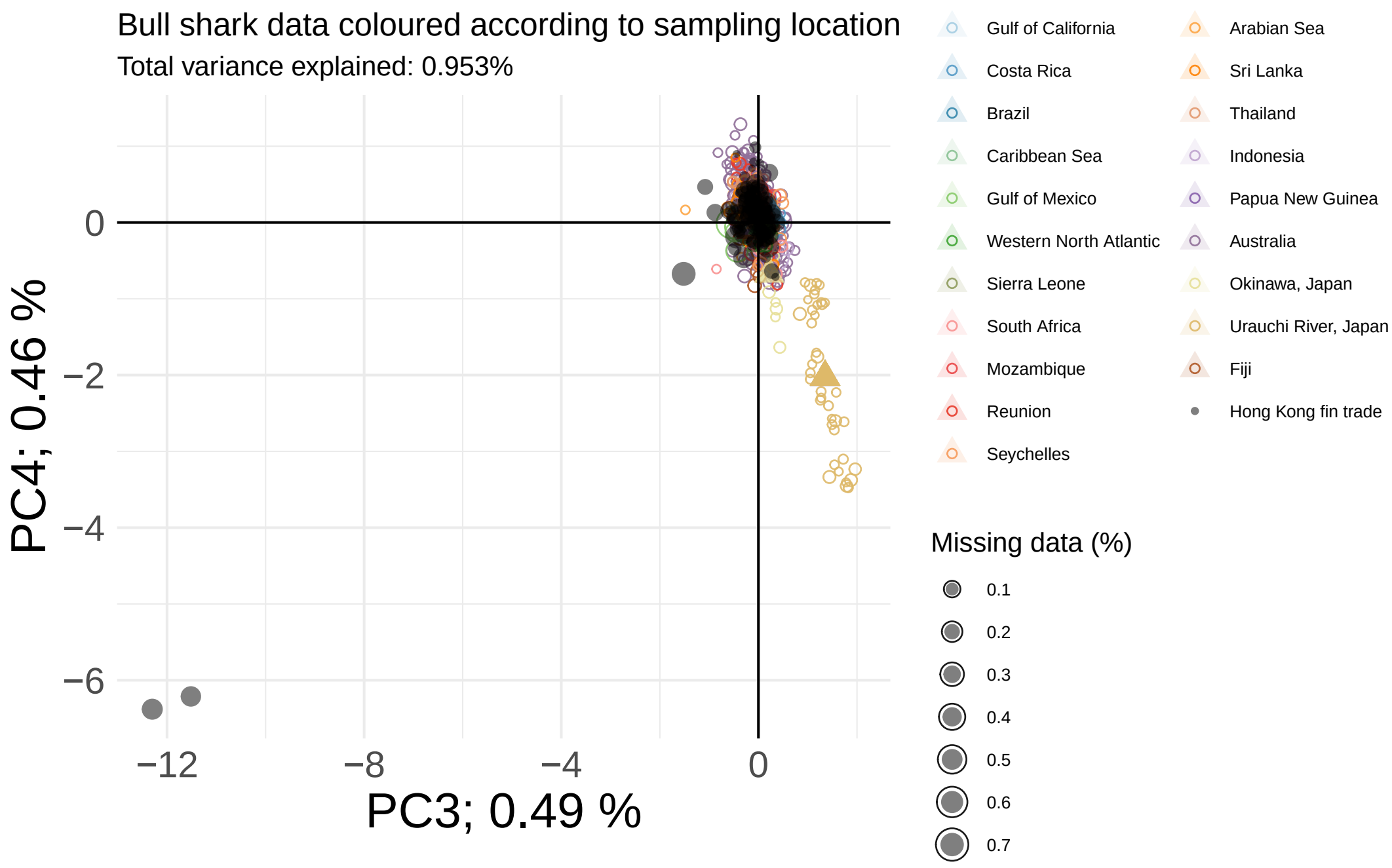

Bull shark data coloured according to sampling location
Total variance explained: 0.953%
PC4; 0.46 %
PC3; 0.49 %
Gulf of California
Costa Rica
Brazil
Caribbean Sea
Gulf of Mexico
Western North Atlantic
Sierra Leone
South Africa
Mozambique
Reunion
Seychelles
Arabian Sea
Sri Lanka
Thailand
Indonesia
Papua New Guinea
Australia
Okinawa, Japan
Urauchi River, Japan
Fiji
Hong Kong fin trade
Missing data (%)
0.1
0.2
0.3
0.4
0.5
0.6
0.7

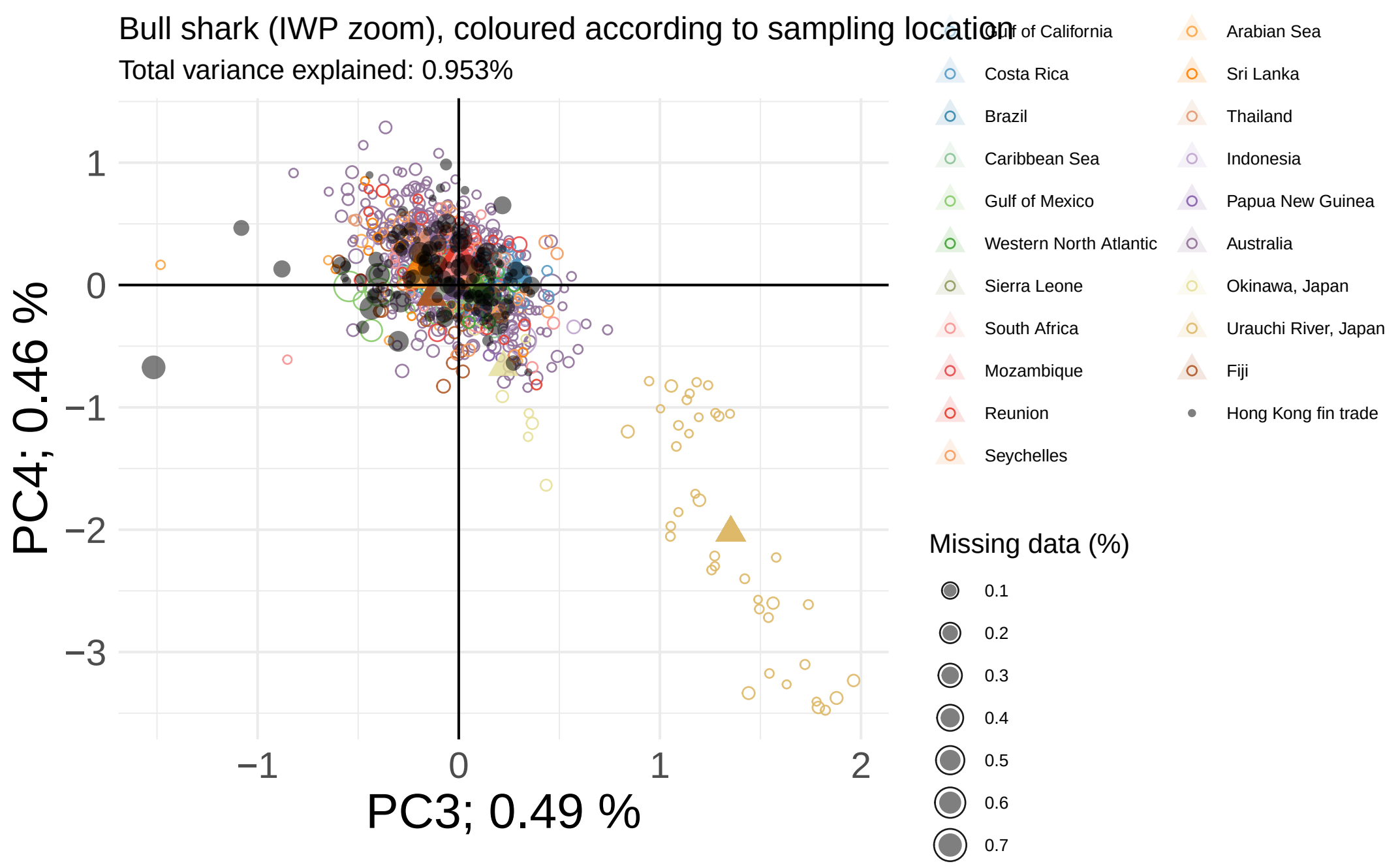
Bull shark (IWP zoom), coloured according to sampling location
Total variance explained: 0.953%
PC4; 0.46 %
PC3; 0.49 %
Gulf of California
Costa Rica
Brazil
Caribbean Sea
Gulf of Mexico
Western North Atlantic
Sierra Leone
South Africa
Mozambique
Reunion
Seychelles
Arabian Sea
Sri Lanka
Thailand
Indonesia
Papua New Guinea
Australia
Okinawa, Japan
Urauchi River, Japan
Fiji
Hong Kong fin trade
Missing data (%)
0.1
0.2
0.3
0.4
0.5
0.6
0.7

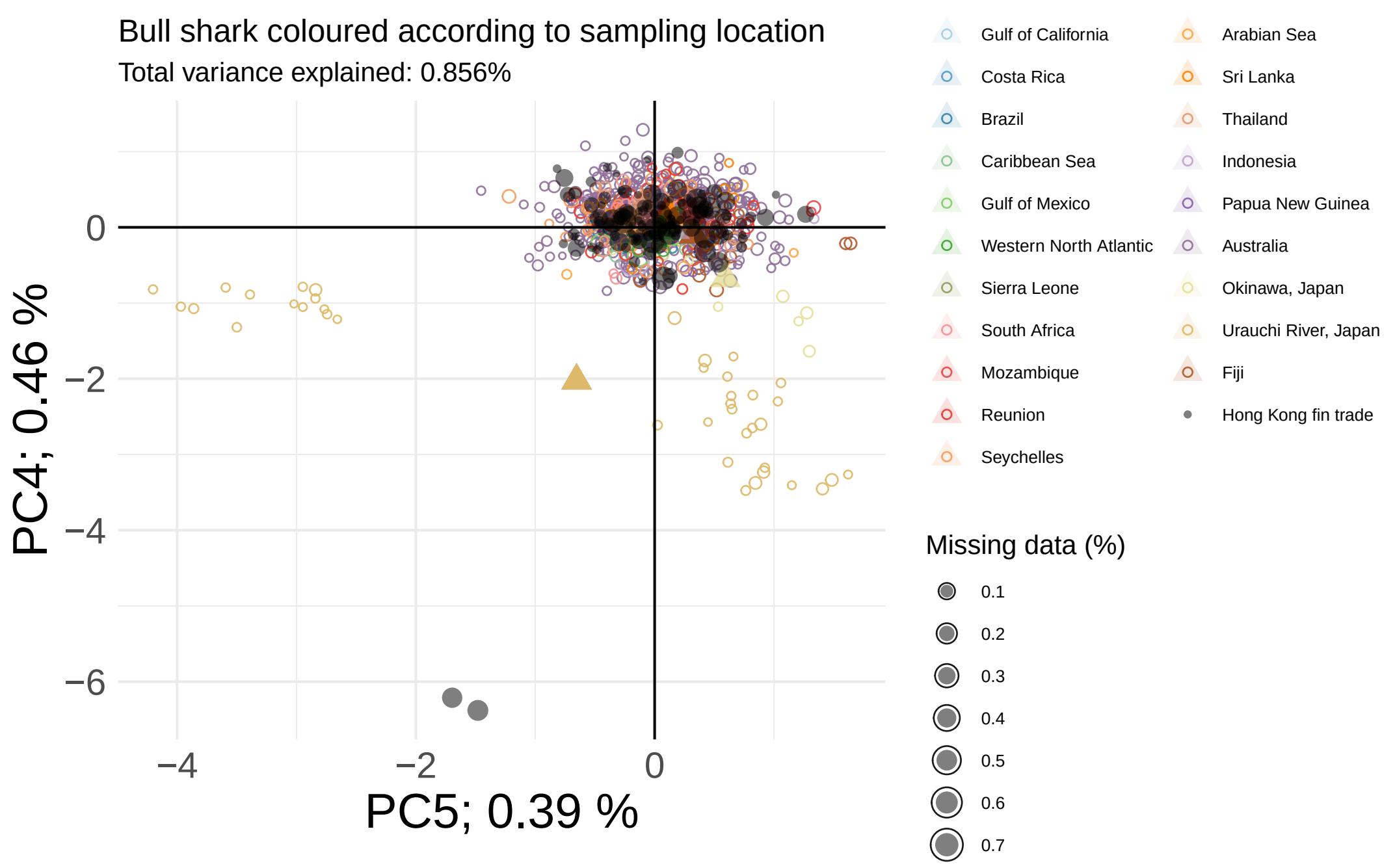
Bull shark coloured according to sampling location
Total variance explained: 0.856%
PC4; 0.46 %
PC5; 0.39 %
Gulf of California
Costa Rica
Brazil
Caribbean Sea
Gulf of Mexico
Western North Atlantic
Sierra Leone
South Africa
Mozambique
Reunion
Seychelles
Arabian Sea
Sri Lanka
Thailand
Indonesia
Papua New Guinea
Australia
Okinawa, Japan
Urauchi River, Japan
Fiji
Hong Kong fin trade
Missing data (%)
0.1
0.2
0.3
0.4
0.5
0.6
0.7

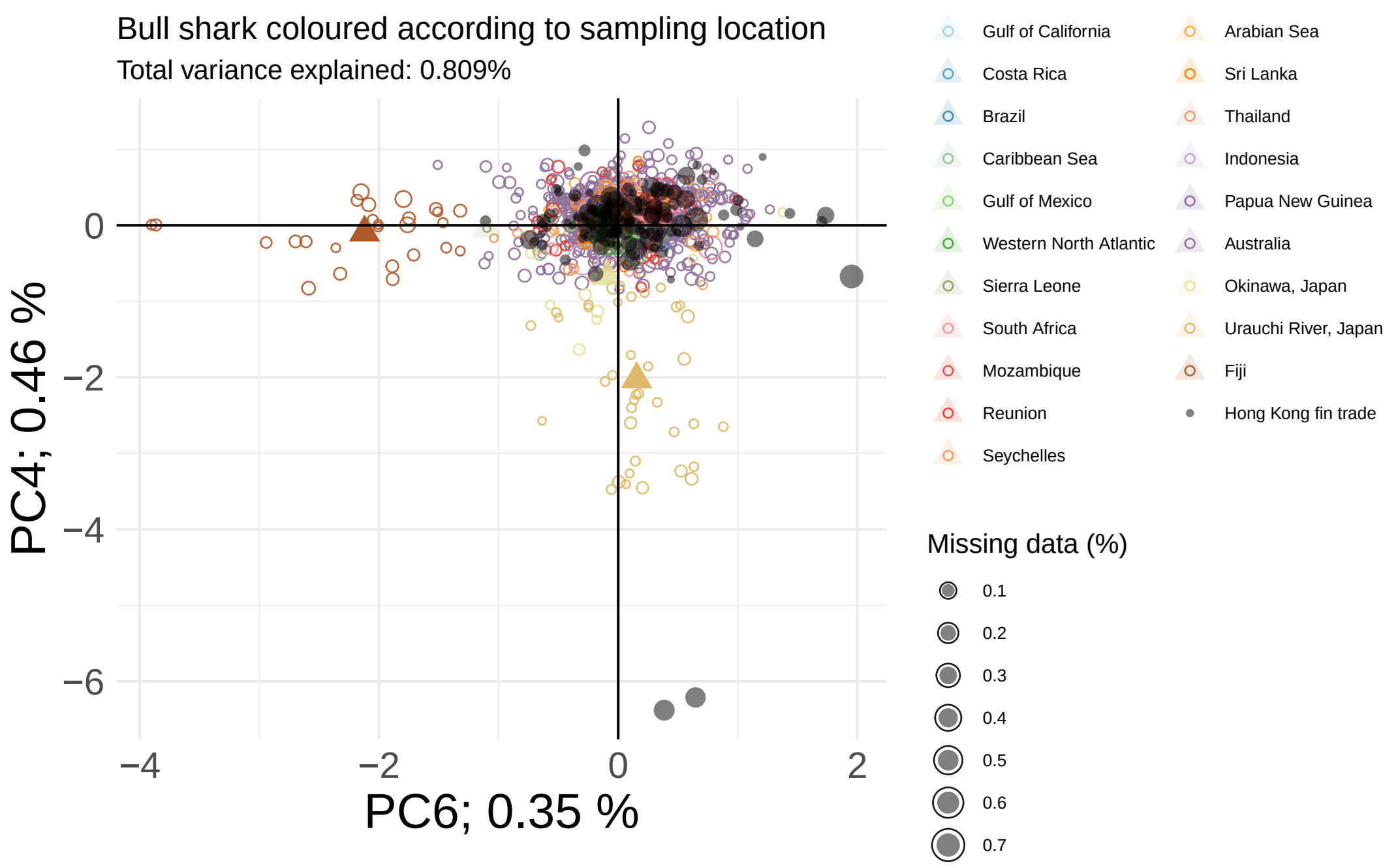

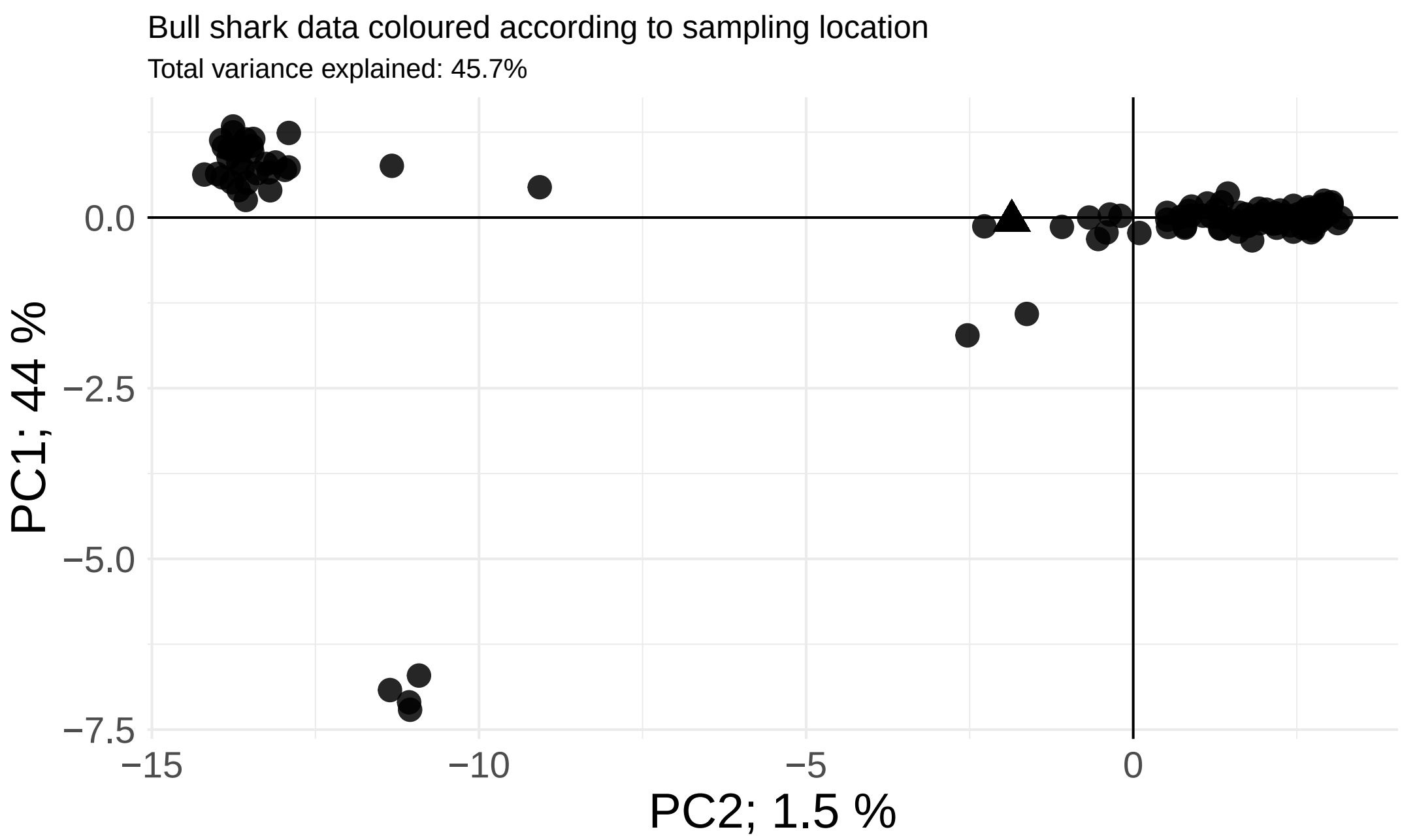

Without HKG

```
BS.dartcap.HK.gl.ref <- BS.dartcap.HK.gl[!BS.dartcap.HK.gl$pop == "Hong Kong fin trade",]
group <- BS.dartcap.HK.gl.ref$other$ind.metrics$Site
group[group == "Japan"] <- BS.dartcap.HK.gl.ref$other$ind.metrics$Site2[group == "Japan"]
group[group == "Okinawa"] <- "Okinawa, Japan"
group[group == "Urauchi River"] <- "Urauchi River, Japan"

pop.levels2 <- c("Gulf of California","Costa Rica","Brazil", "Caribbean Sea",
                 "Gulf of Mexico", "Western North Atlantic",  "Sierra Leone",
                 "South Africa","Mozambique", "Reunion","Seychelles","Arabian Sea",
                 "Sri Lanka","Thailand","Indonesia","Papua New Guinea",
```

```
                 "Australia","Okinawa, Japan", "Urauchi River, Japan","Fiji")
BS.dartcap.HK.gl.ref$pop <- factor(group, levels = pop.levels2)
```

```
pca3 <- adegenet::glPca(BS.dartcap.HK.gl.ref, nf = 5, parallel = TRUE,
                        n.cores = parallel::detectCores() - 1)
save(pca3, file = "DArTcap_HK-noHK_bySite_PCA.Rdata")
```

```
load("DArTcap_HK-noHK_bySite_PCA.Rdata")

var_frac <- pca3$eig/sum(pca3$eig)
print(paste0("Total variance explained: ",signif(sum(var_frac[1:5]) * 100, 3), "%"))
```

```
## [1] "Total variance explained: 46.3%"
```

```
barplot(var_frac[1:20]*100, main = "variance explained",
        col = heat.colors(length(var_frac[1:20])))

pca3$scores <- pca3$scores[order(match(rownames(pca3$scores),
                                       BS.dartcap.HK.gl.ref$ind.names)),]
########## PC1-2 ###################
pop <- BS.dartcap.HK.gl.ref$pop
data <- data.frame(pca3$scores, pop = pop)
data <- merge(data, aggregate(cbind(mean.x = PC1, mean.y = PC2) ~
                                pop, data, mean), by = "pop")

pca.plot <- ggplot2::ggplot(data, ggplot2::aes(x = PC1, y = PC2, color = pop)) +
  ggplot2::geom_point(size = 4, alpha = 0.85) +
  ggplot2::geom_point(ggplot2::aes(x = mean.x,
                                   y = mean.y, color = pop),
                      size = 5,
                      shape = 17,
                      alpha = 0.15) +
  ggplot2::scale_colour_manual(values = colours.20) +
  ggplot2::scale_fill_manual(values = colours.20) +
  ggplot2::guides(col = guide_legend(ncol = 2)) +
  ggplot2::geom_hline(yintercept = 0) +
  ggplot2::geom_vline(xintercept = 0) +
  ggplot2::labs(
    subtitle = paste0("Total variance explained: ",
                      signif(sum(var_frac[1:2]) * 100, 3), "%"),
    y = paste0("PC2; ", signif(var_frac[2] * 100, digits = 2), " %"),
    x = paste0("PC1; ", signif(var_frac[1] * 100, digits = 2), " %"),
    title = "Bull shark data coloured according to sampling site",
    caption = "") +
  ggplot2::theme_minimal() +
  ggplot2::theme(
    axis.text = ggplot2::element_text(size = 15),
    axis.title.x = ggplot2::element_text(size = 20),
    axis.title.y = ggplot2::element_text(size = 20),
    legend.text = ggplot2::element_text(size = 7)
  )

print(pca.plot)
ggplot2::ggsave(pca.plot,
                filename = "DArTcap_noHK_bySite_pca1-2_2142SNPs.png",
                width = 30, height = 15, units = "cm")
```

```r
########## PC1-3 ###################
pop <- BS.dartcap.HK.gl.ref$pop
data <- data.frame(pca3$scores, pop = pop)
data <- merge(data, aggregate(cbind(mean.x = PC1, mean.y = PC3) ~
                                pop, data, mean), by = "pop")

pca.plot <- ggplot2::ggplot(data, ggplot2::aes(x = PC1, y = PC3, color = pop)) +
  ggplot2::geom_point(size = 4, alpha = 0.85) +
  ggplot2::geom_point(ggplot2::aes(x = mean.x,
                                   y = mean.y, color = pop),
                      size = 5,
                      shape = 17,
                      alpha = 0.15) +
  ggplot2::scale_colour_manual(values = colours.20) +
  ggplot2::scale_fill_manual(values = colours.20) +
  ggplot2::guides(col = guide_legend(ncol = 2)) +
  ggplot2::geom_hline(yintercept = 0) +
  ggplot2::geom_vline(xintercept = 0) +
  ggplot2::labs(
    subtitle = paste0("Total variance explained: ",
                      signif(sum(var_frac[c(1,3)]) * 100, 3), "%"),
    y = paste0("PC3; ", signif(var_frac[3] * 100, digits = 2), " %"),
    x = paste0("PC1; ", signif(var_frac[1] * 100, digits = 2), " %"),
    title = "Bull Shark data coloured according to sampling site",
    caption = "") +
  ggplot2::theme_minimal() +
  ggplot2::theme(
    axis.text = ggplot2::element_text(size = 15),
    axis.title.x = ggplot2::element_text(size = 20),
    axis.title.y = ggplot2::element_text(size = 20),
    legend.text = ggplot2::element_text(size = 7)
  )

print(pca.plot)
ggplot2::ggsave(pca.plot,
                filename = "DArTcap_noHK_bySite_pca1-3_2142SNPs.png",
                width = 30, height = 15, units = "cm")

########## PC3-4 ###################
pop <- BS.dartcap.HK.gl.ref$pop
data <- data.frame(pca3$scores, pop = pop)
data <- merge(data, aggregate(cbind(mean.x = PC3, mean.y = PC4) ~
                                pop, data, mean), by = "pop")

pca.plot <- ggplot2::ggplot(data, ggplot2::aes(x = PC3, y = PC4, color = pop)) +
  ggplot2::geom_point(size = 4, alpha = 0.85) +
  ggplot2::geom_point(ggplot2::aes(x = mean.x,
                                   y = mean.y, color = pop),
                      size = 5,
                      shape = 17,
                      alpha = 0.15) +
  ggplot2::scale_colour_manual(values = colours.20) +
  ggplot2::scale_fill_manual(values = colours.20) +
  ggplot2::guides(col = guide_legend(ncol = 2)) +
  ggplot2::geom_hline(yintercept = 0) +
  ggplot2::geom_vline(xintercept = 0) +
```

```r
  ggplot2::labs(
    subtitle = paste0("Total variance explained: ",
                      signif(sum(var_frac[3:4]) * 100, 3), "%"),
    y = paste0("PC4; ", signif(var_frac[4] * 100, digits = 2), " %"),
    x = paste0("PC3; ", signif(var_frac[3] * 100, digits = 2), " %"),
    title = "Bull Shark data coloured according to sampling site",
    caption = "") +
  ggplot2::theme_minimal() +
  ggplot2::theme(
    axis.text = ggplot2::element_text(size = 15),
    axis.title.x = ggplot2::element_text(size = 20),
    axis.title.y = ggplot2::element_text(size = 20),
    legend.text = ggplot2::element_text(size = 7)
  )

print(pca.plot)
ggplot2::ggsave(pca.plot,
                filename = "DArTcap_noHK_bySite_PCA3-4_2142SNPs.png",
                width = 30, height = 15, units = "cm")


########## PC3-5 ###################
pop <- BS.dartcap.HK.gl.ref$pop
data <- data.frame(pca3$scores, pop = pop)
data <- merge(data, aggregate(cbind(mean.x = PC3, mean.y = PC5) ~
                                pop, data, mean), by = "pop")

pca.plot <- ggplot2::ggplot(data, ggplot2::aes(x = PC3, y = PC5, color = pop)) +
  ggplot2::geom_point(size = 4, alpha = 0.85) +
  ggplot2::geom_point(ggplot2::aes(x = mean.x,
                                   y = mean.y, color = pop),
                      size = 5,
                      shape = 17,
                      alpha = 0.15) +
  ggplot2::scale_colour_manual(values = colours.20) +
  ggplot2::scale_fill_manual(values = colours.20) +
  ggplot2::guides(col = guide_legend(ncol = 2)) +
  ggplot2::geom_hline(yintercept = 0) +
  ggplot2::geom_vline(xintercept = 0) +
  ggplot2::labs(
    subtitle = paste0("Total variance explained: ",
                      signif(sum(var_frac[c(3,5)]) * 100, 3), "%"),
    y = paste0("PC5; ", signif(var_frac[5] * 100, digits = 2), " %"),
    x = paste0("PC3; ", signif(var_frac[3] * 100, digits = 2), " %"),
    title = "Bull shark data coloured according to sampling site",
    caption = "") +
  ggplot2::theme_minimal() +
  ggplot2::theme(
    axis.text = ggplot2::element_text(size = 15),
    axis.title.x = ggplot2::element_text(size = 20),
    axis.title.y = ggplot2::element_text(size = 20),
    legend.text = ggplot2::element_text(size = 7)
  )

print(pca.plot)
ggplot2::ggsave(pca.plot,
                filename = "DArTcap_noHK_bySite_PCA3-5_2142SNPs.png",
```

```
            width = 30, height = 15, units = "cm")
```

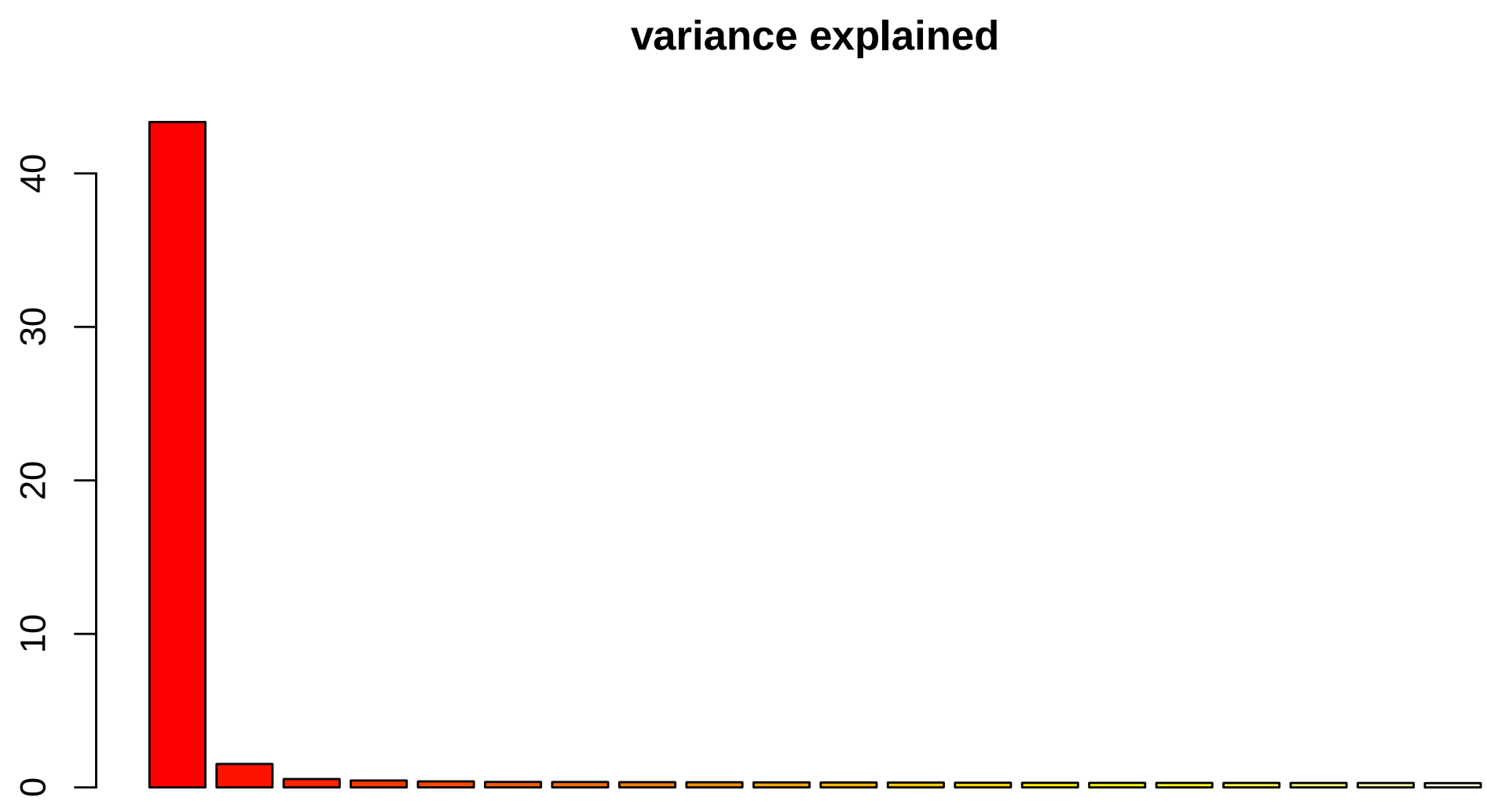

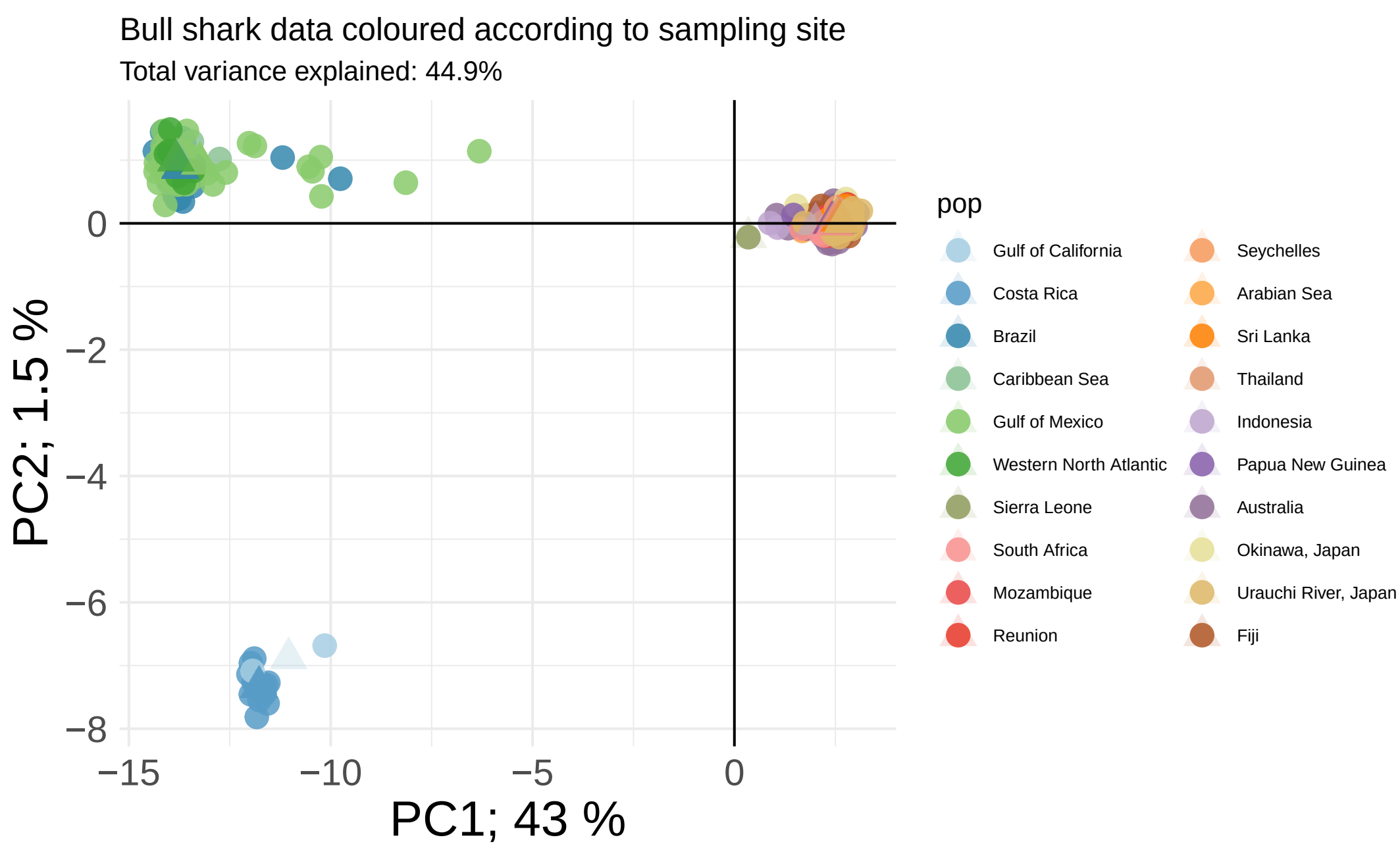

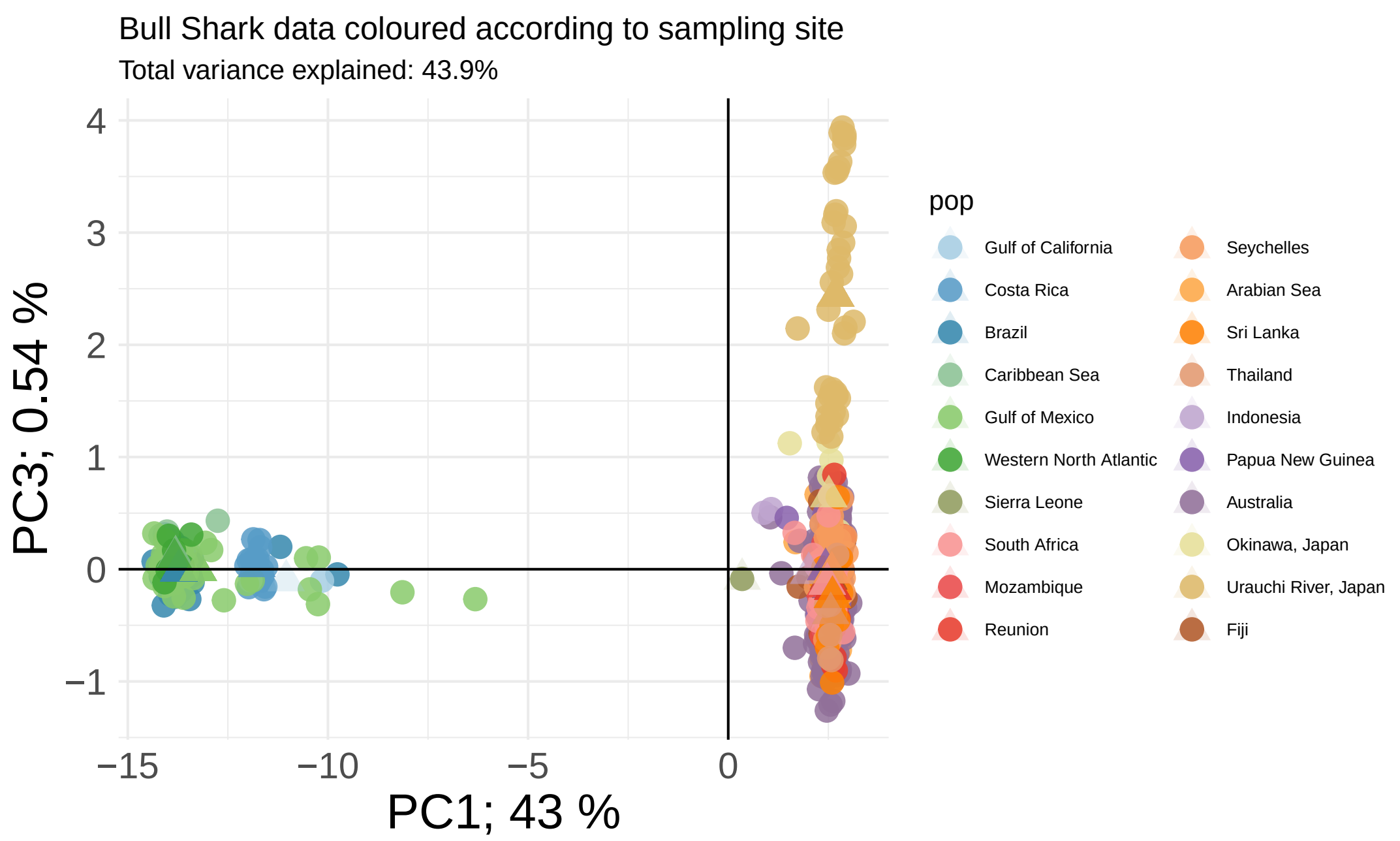
Bull Shark data coloured according to sampling site
Total variance explained: 43.9%
PC3; 0.54 %
PC1; 43 %
4
3
2
1
0
−1
−15
−10
−5
0
pop
Gulf of California
Costa Rica
Brazil
Caribbean Sea
Gulf of Mexico
Western North Atlantic
Sierra Leone
South Africa
Mozambique
Reunion
Seychelles
Arabian Sea
Sri Lanka
Thailand
Indonesia
Papua New Guinea
Australia
Okinawa, Japan
Urauchi River, Japan
Fiji

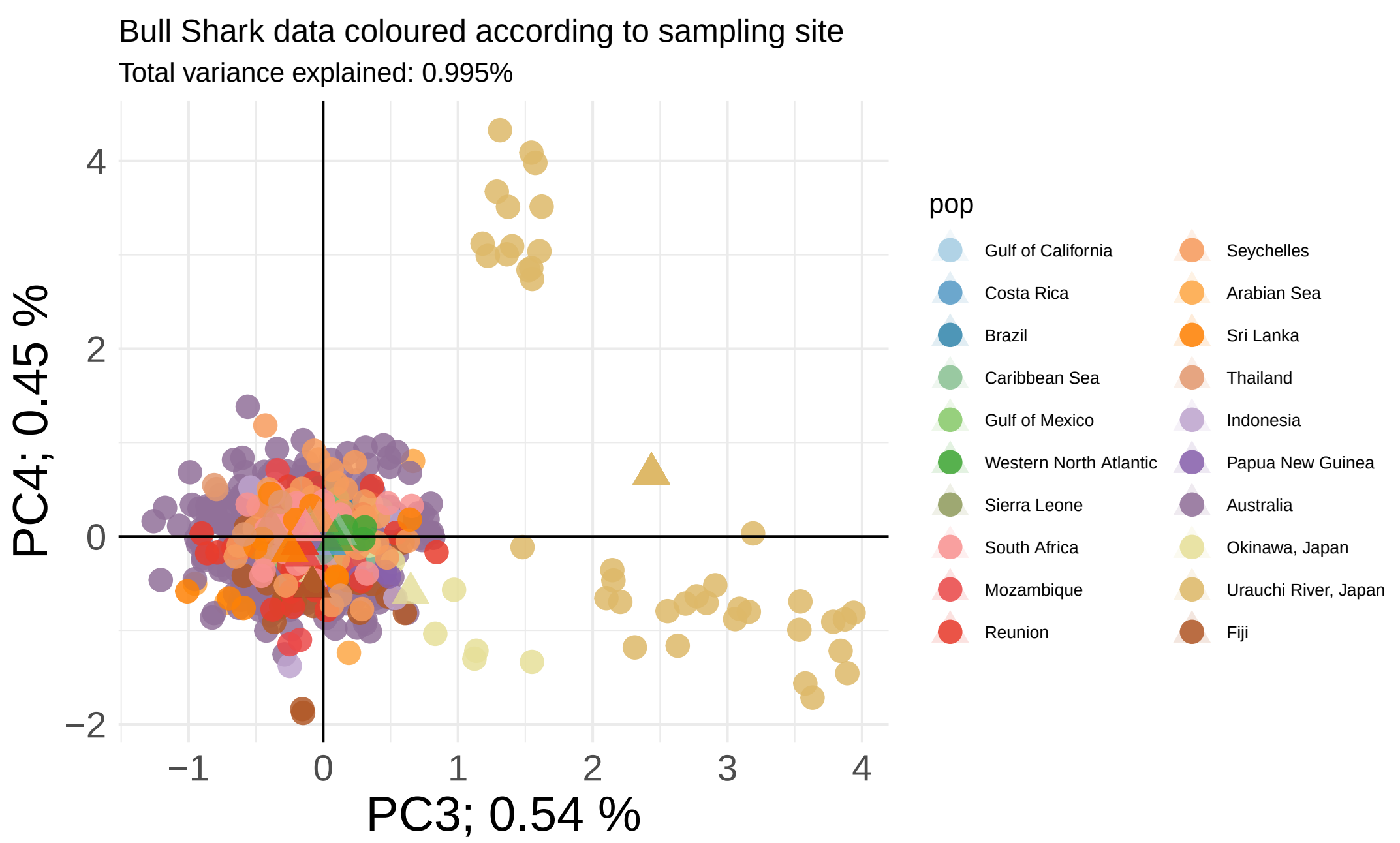
Bull Shark data coloured according to sampling site
Total variance explained: 0.995%
PC4; 0.45 %
PC3; 0.54 %
4
2
0
−2
−1
0
1
2
3
4
pop
Gulf of California
Costa Rica
Brazil
Caribbean Sea
Gulf of Mexico
Western North Atlantic
Sierra Leone
South Africa
Mozambique
Reunion
Seychelles
Arabian Sea
Sri Lanka
Thailand
Indonesia
Papua New Guinea
Australia
Okinawa, Japan
Urauchi River, Japan
Fiji

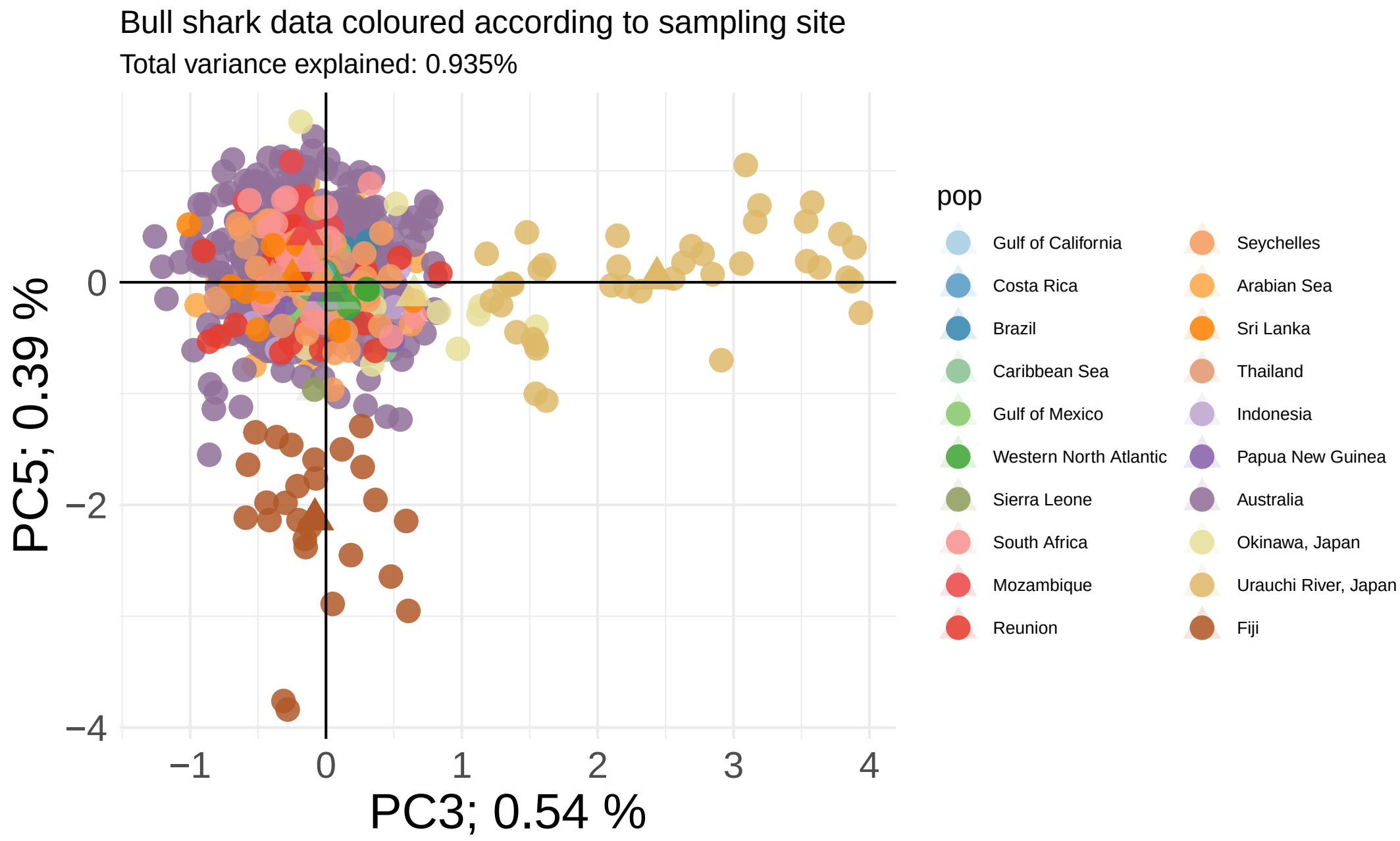

Bull shark data coloured according to sampling site
Total variance explained: 0.935%
PC5; 0.39 %
PC3; 0.54 %
0
−2
−4
−1
0
1
2
3
4
pop
Gulf of California
Costa Rica
Brazil
Caribbean Sea
Gulf of Mexico
Western North Atlantic
Sierra Leone
South Africa
Mozambique
Reunion
Seychelles
Arabian Sea
Sri Lanka
Thailand
Indonesia
Papua New Guinea
Australia
Okinawa, Japan
Urauchi River, Japan
Fiji

Provenance assignment

DAPC assignment

Split data in reference and HKG samples Given the unequal samples size, we also equalize the data sets per population. Specifically, we sub sample the IWP population to 120 samples (similar as W-ATL). see Foster et al. 2018 and Puechmaille 2016.

```
BS.dartcap.HK.gl.ref <- BS.dartcap.HK.gl[!BS.dartcap.HK.gl$pop == "Hong Kong fin trade",]
BS.dartcap.HK.gl.assign <- BS.dartcap.HK.gl[BS.dartcap.HK.gl$pop == "Hong Kong fin trade",]

# aus.sample <- c(BS.dartcap.HK.gl.ref$other$ind.metrics$INDIVIDUALS
#                 [BS.dartcap.HK.gl.ref$other$ind.metrics$Site != "Australia"],
#                 sample(BS.dartcap.HK.gl.ref$other$ind.metrics$INDIVIDUALS
#                        [BS.dartcap.HK.gl.ref$other$ind.metrics$Site == "Australia"],
#                        size = 60))

iwp.sample <- c(BS.dartcap.HK.gl.ref$other$ind.metrics$INDIVIDUALS
                [!BS.dartcap.HK.gl.ref$other$ind.metrics$Region2 == "IWP"],
                sample(BS.dartcap.HK.gl.ref$other$ind.metrics$INDIVIDUALS
                       [BS.dartcap.HK.gl.ref$other$ind.metrics$Region2  == "IWP"],
                       size = 120))
BS.dartcap.HK.gl.ref <- BS.dartcap.HK.gl.ref[
  BS.dartcap.HK.gl.ref$other$ind.metrics$INDIVIDUALS %in% iwp.sample,]

pop.levels2 <- c("E-PAC","W-ATL", "E-ATL", "IWP", "Japan", "Fiji")

BS.dartcap.HK.gl.ref$pop <- factor(BS.dartcap.HK.gl.ref$other$ind.metrics$Region2,
                                   levels = pop.levels2)
BS.dartcap.HK.gl.ref <- BS.dartcap.HK.gl.ref[order(
  BS.dartcap.HK.gl.ref$pop, BS.dartcap.HK.gl.ref$ind.names)]

save(BS.dartcap.HK.gl.ref,BS.dartcap.HK.gl.assign,
     file = "Bull_shark_HK_DArTcap_SPLIT_objects.Rdata")
```

```
set.seed(124)
grp <-
  adegenet::find.clusters(
    BS.dartcap.HK.gl.ref,
    max.n.clust = 18,
    n.pca = adegenet::nInd(BS.dartcap.HK.gl.ref) / 3,
    stat = "BIC",
    parallel = TRUE,
    n.cores = parallel::detectCores() - 1
  )

save(grp, file = "DArTcap_HK_grp_2.Rdata")
```

```
# load("DArTcap_HK_grp_.Rdata")
load("DArTcap_HK_grp_2.Rdata")

y <- as.numeric(grp$Kstat)
x <- 1:18
```

```r
data <- data.frame(x,y,stringsAsFactors = F)
plot <- ggplot2::ggplot(data, ggplot2::aes(x,y)) +
  ggplot2::geom_point(size = 5, shape = 1, color = "blue") +
  ggplot2::geom_line(linewidth = 1,color = "blue") +
  ggplot2::scale_x_continuous(name = waiver(),
                              breaks = seq(from = 0,
                                           to = nrow(BS.dartcap.HK.gl) - 1,
                                           by = 1)) +
  ggplot2::labs(subtitle = "",
                y = "Bayesian Information Criterion",
                x = "Number of clusters",
                title = "",
                caption = "") +
  ggplot2::theme_classic() +
  ggplot2::theme(
    axis.text = ggplot2::element_text(size = 5),
    axis.title.x = ggplot2::element_text(size = 20,vjust = 0, hjust = 0.5),
    axis.title.y = ggplot2::element_text(size = 20,vjust = 2, hjust = 0.5))
print(plot)

ggplot2::ggsave(plot, filename = "DArTcap_HK_grp.png", width = 30, height = 15,
                units = "cm")
```

Optimal number of clusters with neutral markers

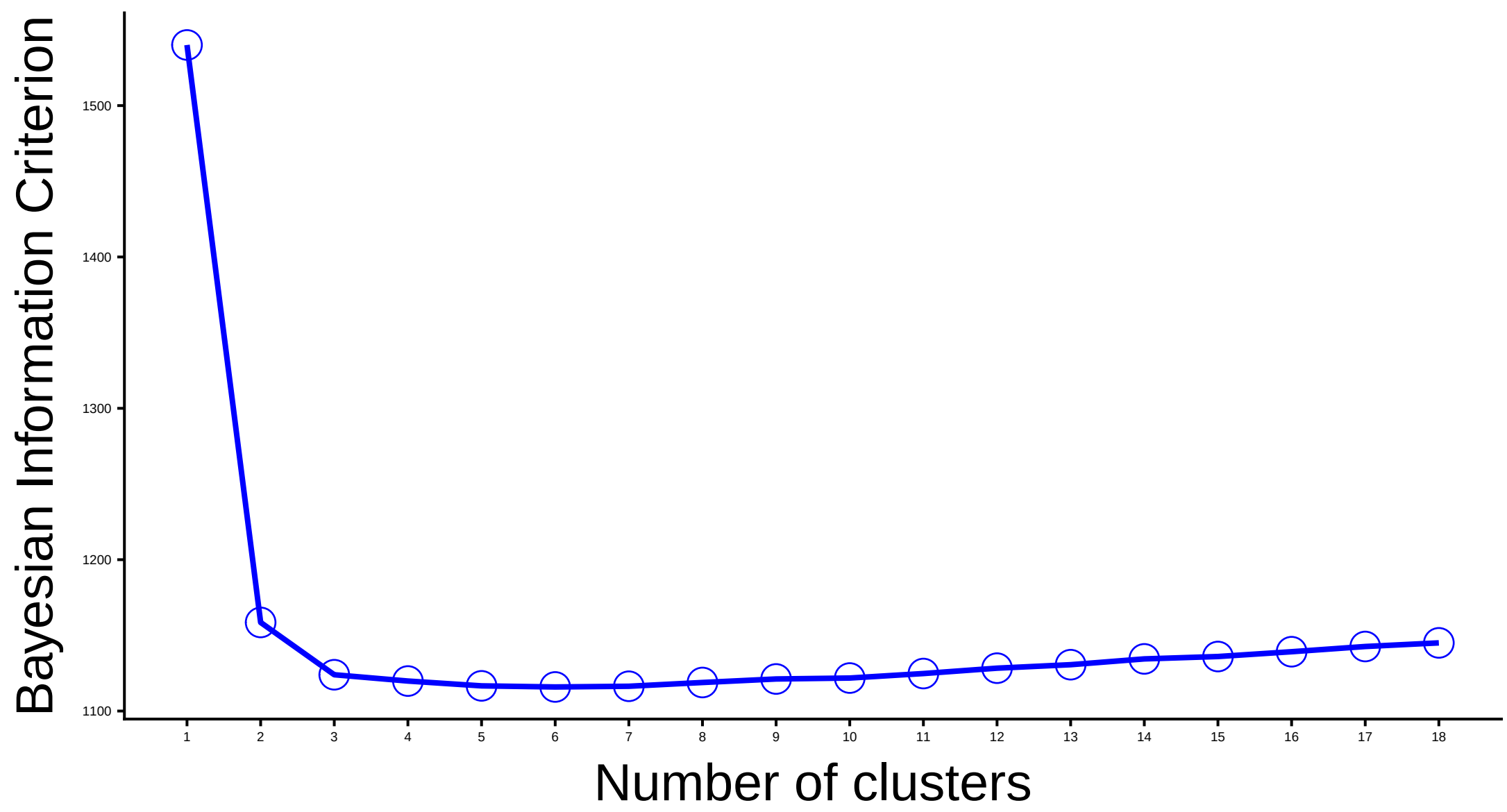

Cross-validation to identify the optimal number of PCs Uses a training - holdout set of individuals to check how reliable individuals can be assigned.

```r
set.seed(124)
x <- BS.dartcap.HK.gl.ref
# x <- x[x$pop != "Sierra Leone",]
x <- x[x$pop != "E-ATL",]

xval <-
```

```r
  adegenet::xvalDapc(
  tab(x, NA.method = "mean"),
  adegenet::pop(x),
  n.pca = c(seq(1, adegenet::nInd(x), by = 50),adegenet::nInd(x)),
  training.set = 0.9,
  result = "groupMean",
  center = TRUE,
  scale = FALSE,
  n.rep = 100,
  # parallel = "multicore",
  parallel = "snow",
  ncpus = 1,
  xval.plot = TRUE
  )
print(xval[2:6])
PC <- as.numeric(xval$`Number of PCs Achieving Lowest MSE`)

#refine the analysis
set.seed(124)
png(file="DArTcap_HK_xval.png")
xval <-
  adegenet::xvalDapc(
  tab(x, NA.method = "mean"),
  adegenet::pop(x),
  n.pca = (PC - 15):(PC + 15),
  n.rep = 10,
  # parallel = "multicore",
  parallel = "snow",
  ncpus = 1,
  xval.plot = TRUE
  )
dev.off()
# save(xval, file = "DArTcap_HK_xval.rdata")
save(xval, file = "DArTcap_HK_xval_2.rdata")
```

```r
# load("DArTcap_HK_xval.rdata")
load("DArTcap_HK_xval_2.rdata")

print(xval[2:6])
```

```
## $`Median and Confidence Interval for Random Chance`
##      2.5%       50%     97.5%
## 0.1558496 0.2007304 0.2409684
##
## $`Mean Successful Assignment by Number of PCs of PCA`
##        86        87        88        89        90        91        92        93        94
## 0.9870000 0.9756667 0.9693333 0.9686667 0.9716667 0.9766667 0.9880000 0.9933333 0.9773333 0.99666
##       101       102       103       104       105       106       107       108       109        1
## 0.9773333 0.9790000 0.9903333 0.9750000 0.9773333 0.9846667 0.9903333 0.9773333 0.9916667 0.97666
##       116
## 0.9750000
##
## $`Number of PCs Achieving Highest Mean Success`
## [1] "95"
##
## $`Root Mean Squared Error by Number of PCs of PCA`
##          86          87          88          89          90          91          92          93          9
```

```
## 0.02008316 0.02888675 0.03732738 0.04260934 0.03804968 0.03938415 0.02190890 0.01490712 0.0297396
##          99          100          101          102          103          104          105          106          10
## 0.02314207 0.01864880 0.02973961 0.03314949 0.02584140 0.03834058 0.03806427 0.02314207 0.0186488
##         112          113          114          115          116
## 0.01937926 0.01864880 0.01937926 0.02206052 0.03656045
##
## $`Number of PCs Achieving Lowest MSE`
## [1] "95"
```

```
PC <- as.numeric(xval$`Number of PCs Achieving Lowest MSE`)
```

```
dapc.all.object.names <- c()
for (K in 2:10) {
  set.seed(124)
  grp <- adegenet::find.clusters(BS.dartcap.HK.gl.ref, n.clust = K, n.pca = PC)
  set.seed(124)
  dapc.all.neutral <- adegenet::dapc(BS.dartcap.HK.gl.ref, grp$grp, n.da = K - 1,
                                     n.pca = PC)
  assign(paste0("dapc.all.", K),value = dapc.all.neutral)
  dapc.all.object.names <- c(dapc.all.object.names,
                                     paste0("dapc.all.", K))
}
# save(list = dapc.all.object.names, dapc.all.object.names,
#      file = "DArTcap_HK_DAPC3.Rdata")
save(list = dapc.all.object.names, dapc.all.object.names,
     file = "DArTcap_HK_DAPC4.Rdata")
```

DAPC barplot Group individuals according to DAPC posterior membership.

```
load("Bull_shark_HK_DArTcap_SPLIT_objects.Rdata")
load("DArTcap_HK_DAPC4.Rdata")

for (K in 2:10) {
  colour <- colours.prior[1:K]
  dapc <- get(dapc.all.object.names[K - 1])
  post <- as.matrix(dapc$posterior)
  colnames(post) <- paste0("Group", 1:nlevels(dapc$grp))
  locations <- BS.dartcap.HK.gl.ref$pop
  # post <- post[order(BS.dartcap.HK.gl.ref$pop),]

  plot.dapc.FDD(x = post,
                locations = locations,
                colour = colour,
                region.lwd = 1,
                plotTitle = paste0("DAPC: all Bull Sharks\n for K=",
                                   K, " & PC=", PC, sep = ""))
  dev.print(
    device = png,
    file = paste0("1.DArTcap_HK_DAPC_barplot4_K", K, ".png"),
    res = 300,
    width = 30,
    height = 15,
    units = "cm")
}
```

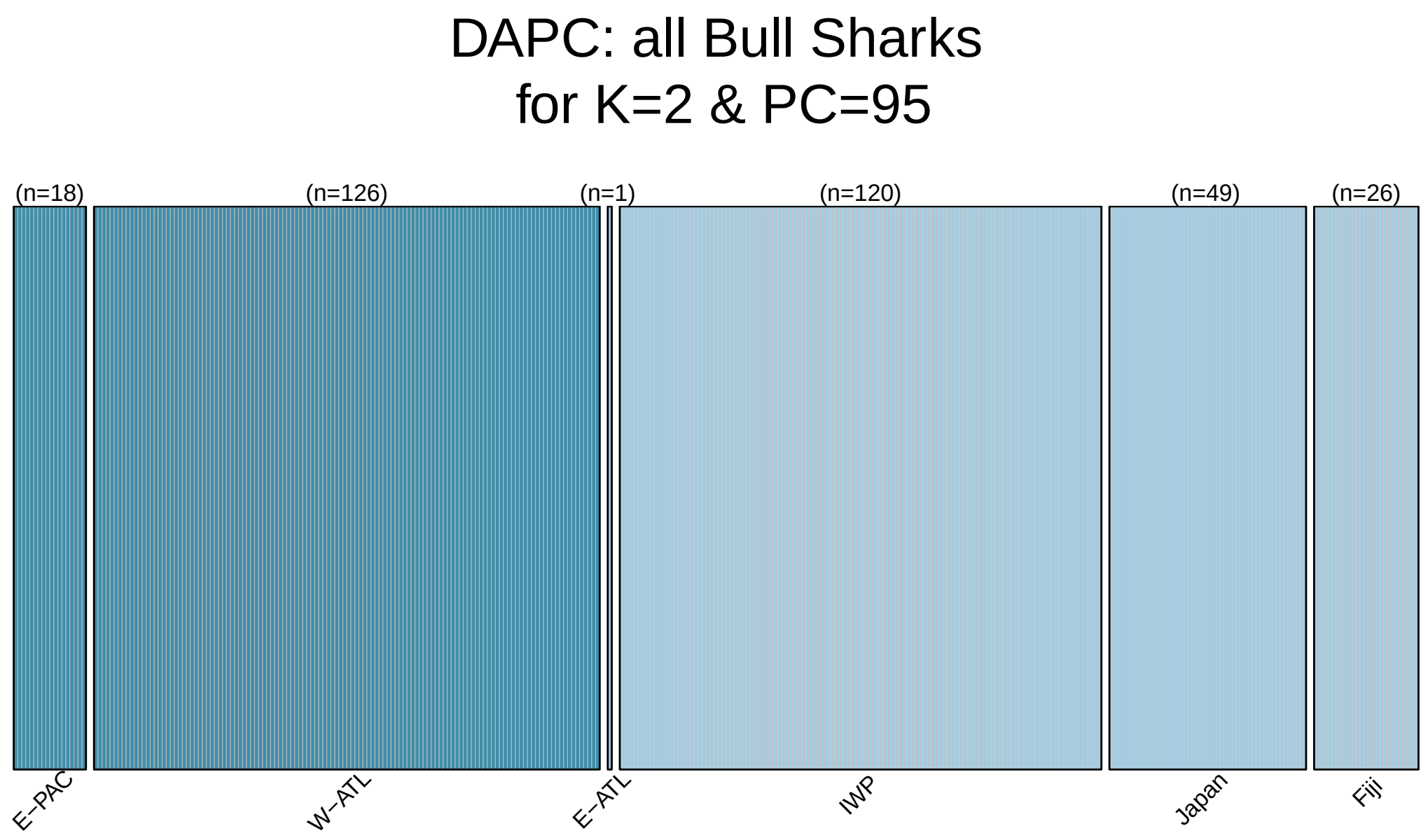
DAPC: all Bull Sharks
for K=2 & PC=95
(n=18)
(n=126)
(n=1)
(n=120)
(n=49)
(n=26)
E–PAC
W–ATL
E–ATL
IWP
Japan
Fiji

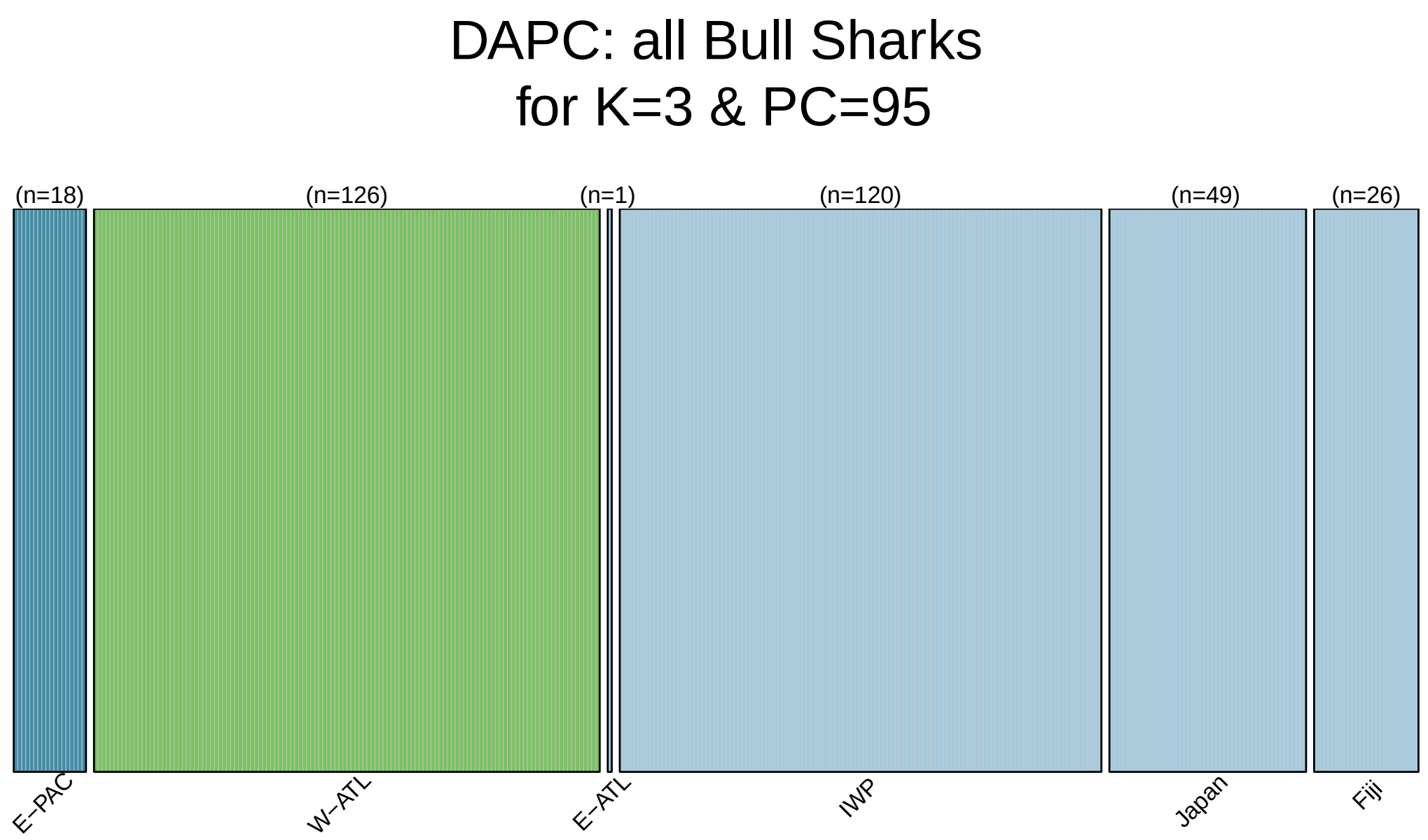
DAPC: all Bull Sharks
for K=3 & PC=95
(n=18)
(n=126)
(n=1)
(n=120)
(n=49)
(n=26)
E–PAC
W–ATL
E–ATL
IWP
Japan
Fiji

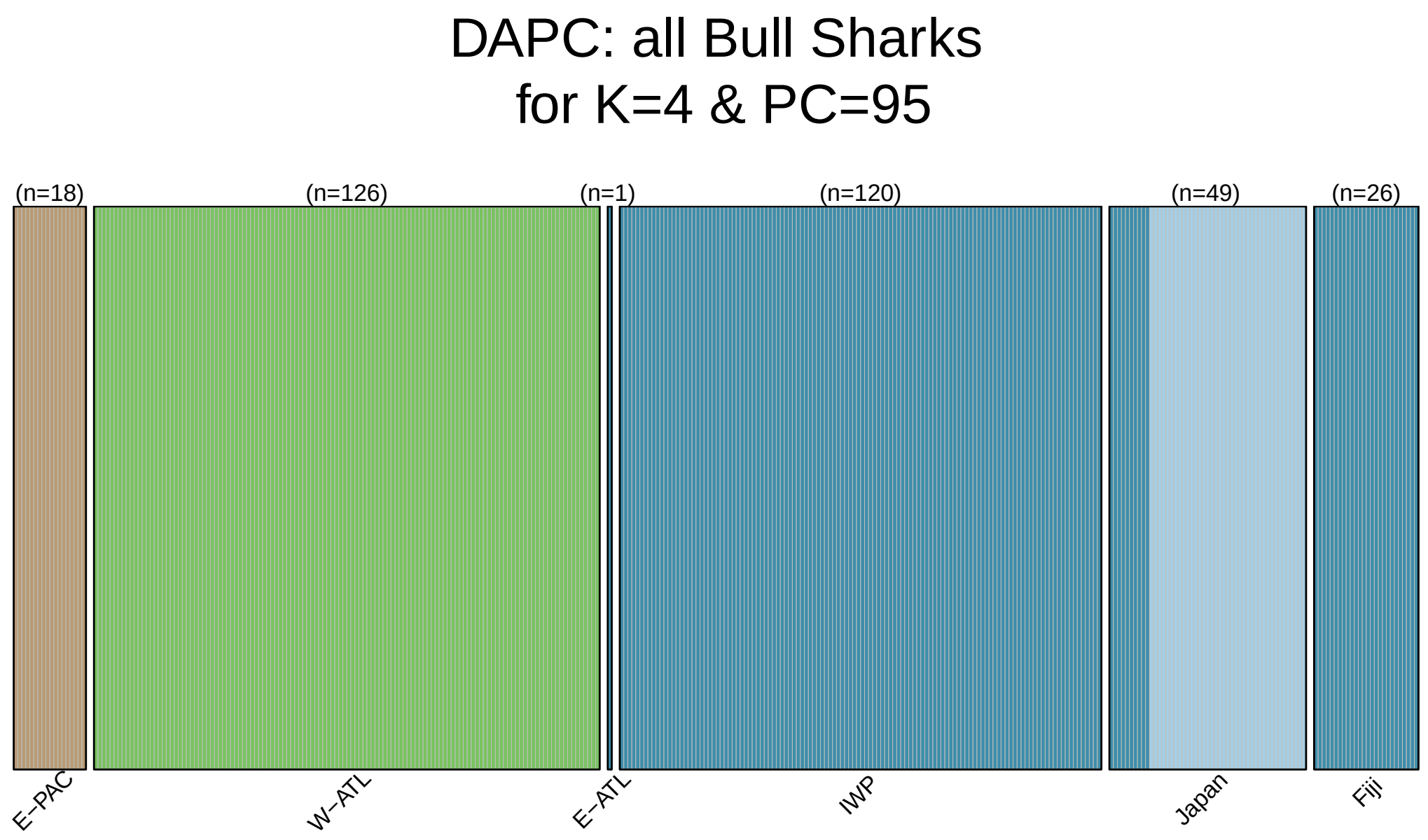
DAPC: all Bull Sharks
for K=4 & PC=95
(n=18)
(n=126)
(n=1)
(n=120)
(n=49)
(n=26)
E–PAC
W–ATL
E–ATL
IWP
Japan
Fiji

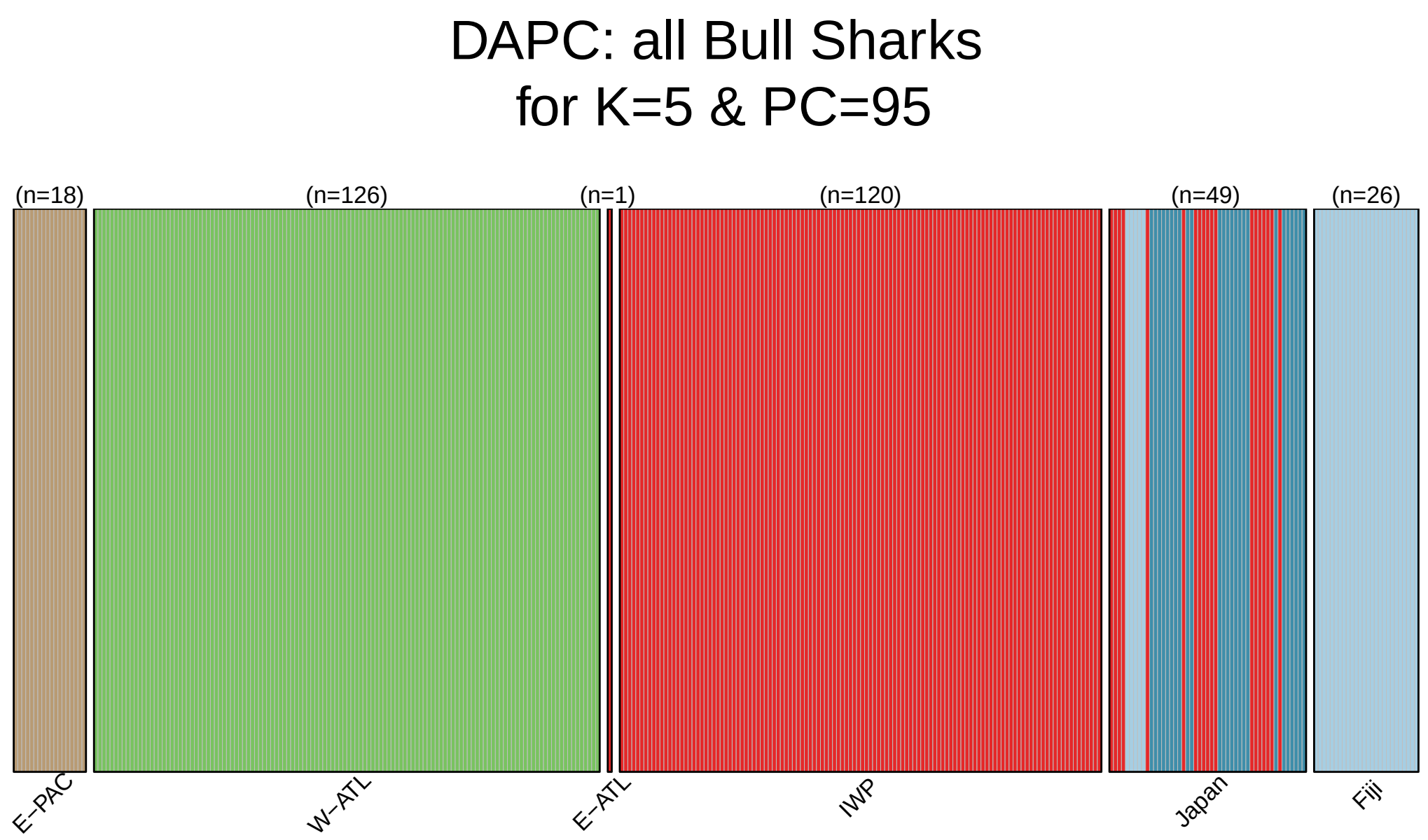
DAPC: all Bull Sharks
for K=5 & PC=95
(n=18)
(n=126)
(n=1)
(n=120)
(n=49)
(n=26)
E–PAC
W–ATL
E–ATL
IWP
Japan
Fiji

DAPC: all Bull Sharks
for K=6 & PC=95

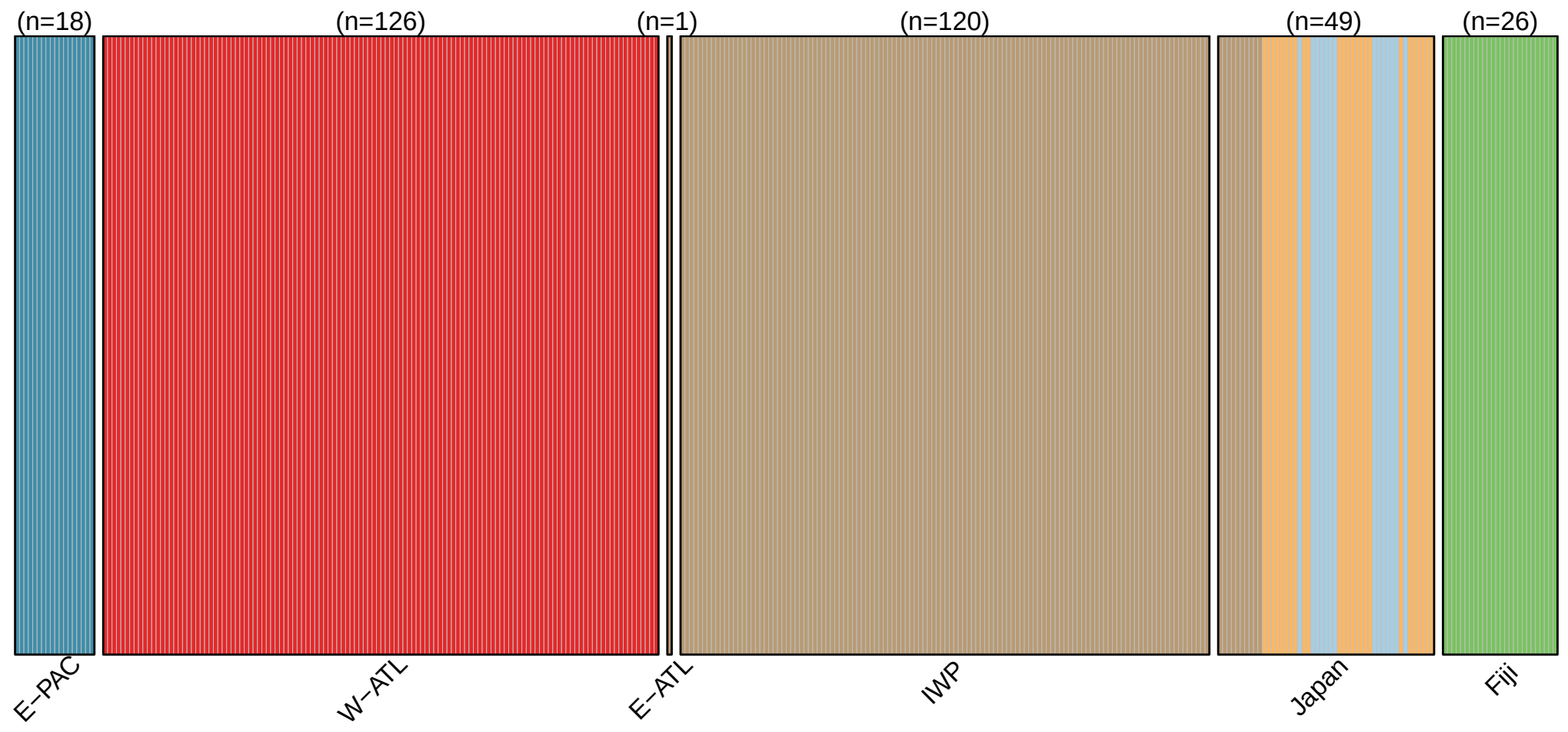

DAPC: all Bull Sharks
for K=7 & PC=95

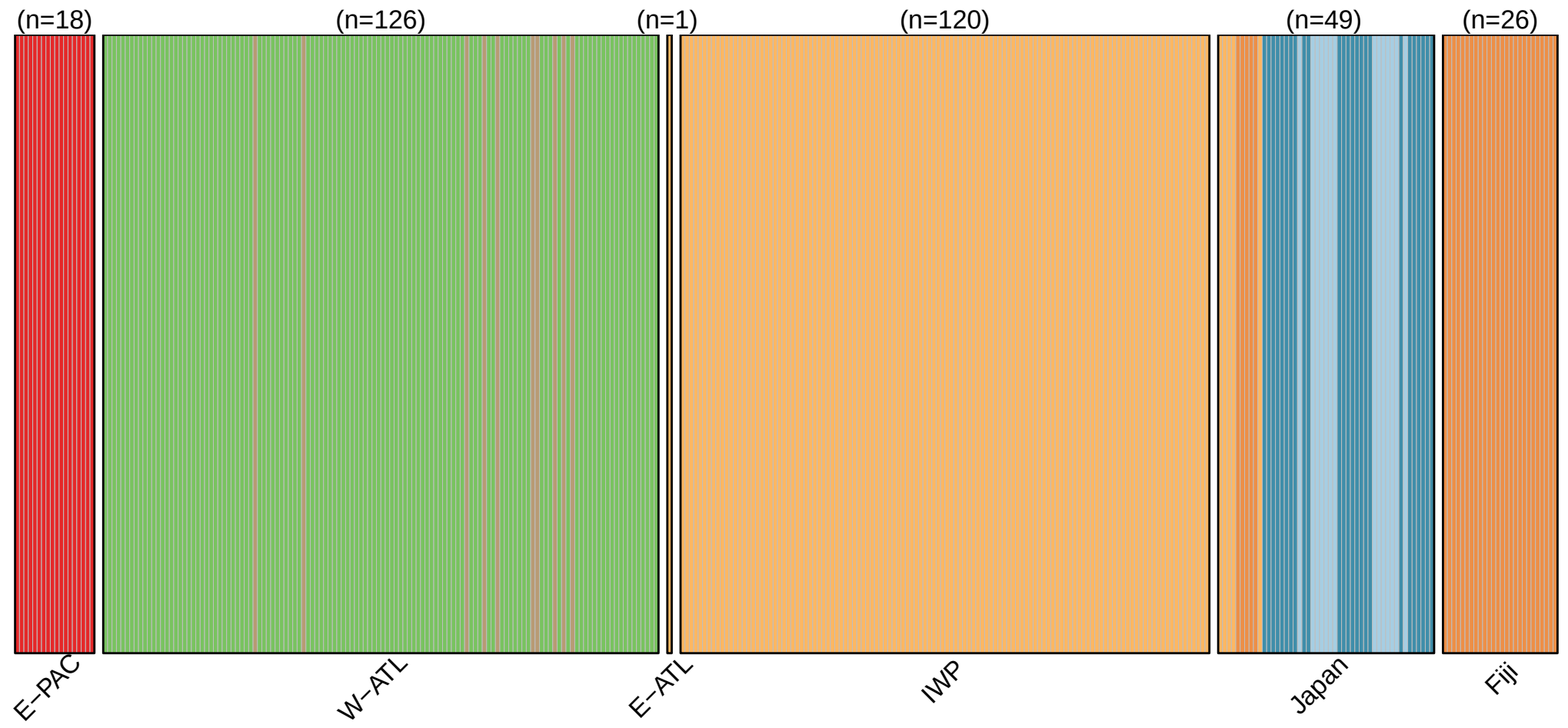

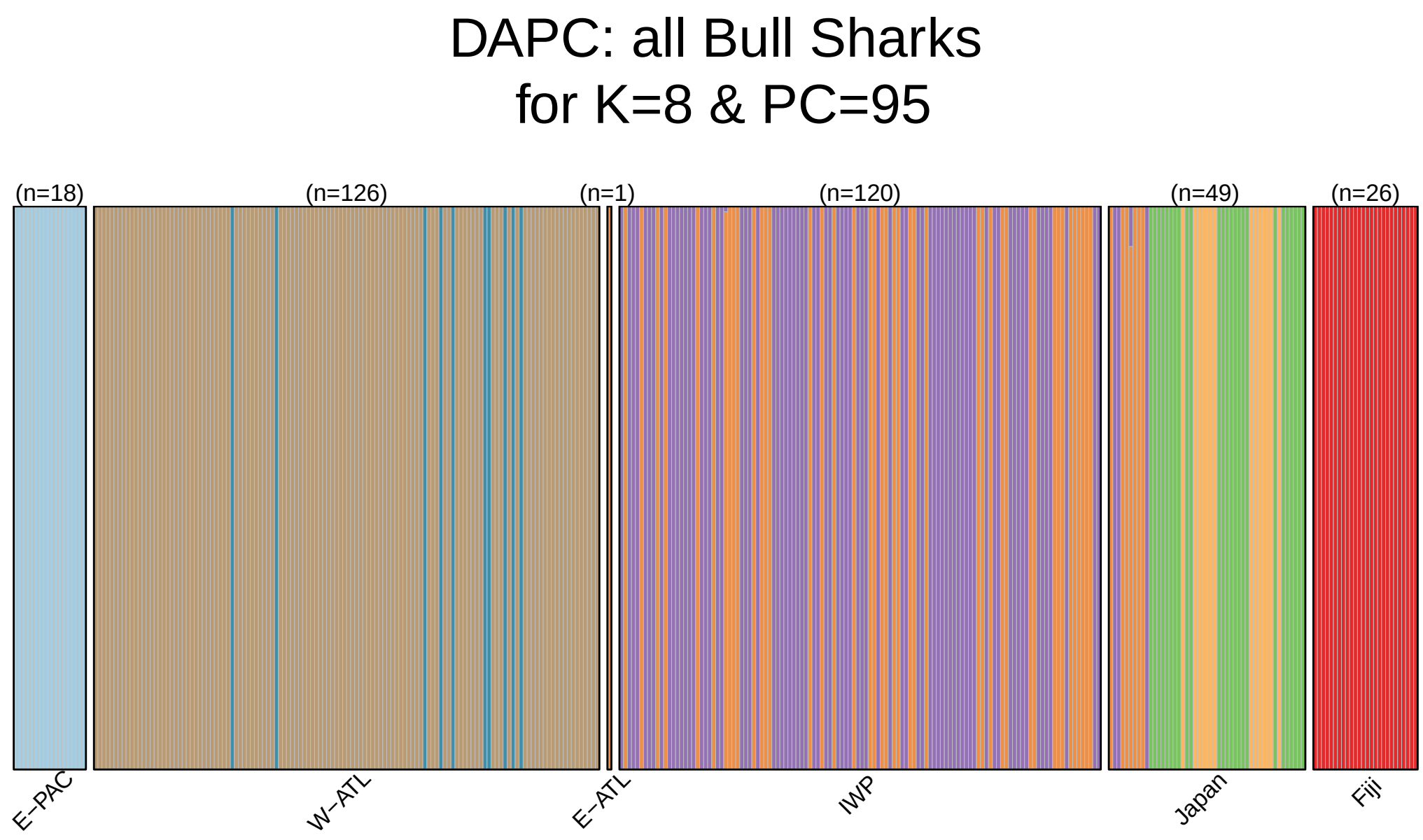
DAPC: all Bull Sharks
for K=8 & PC=95
(n=18)
(n=126)
(n=1)
(n=120)
(n=49)
(n=26)
E–PAC
W–ATL
E–ATL
IWP
Japan
Fiji

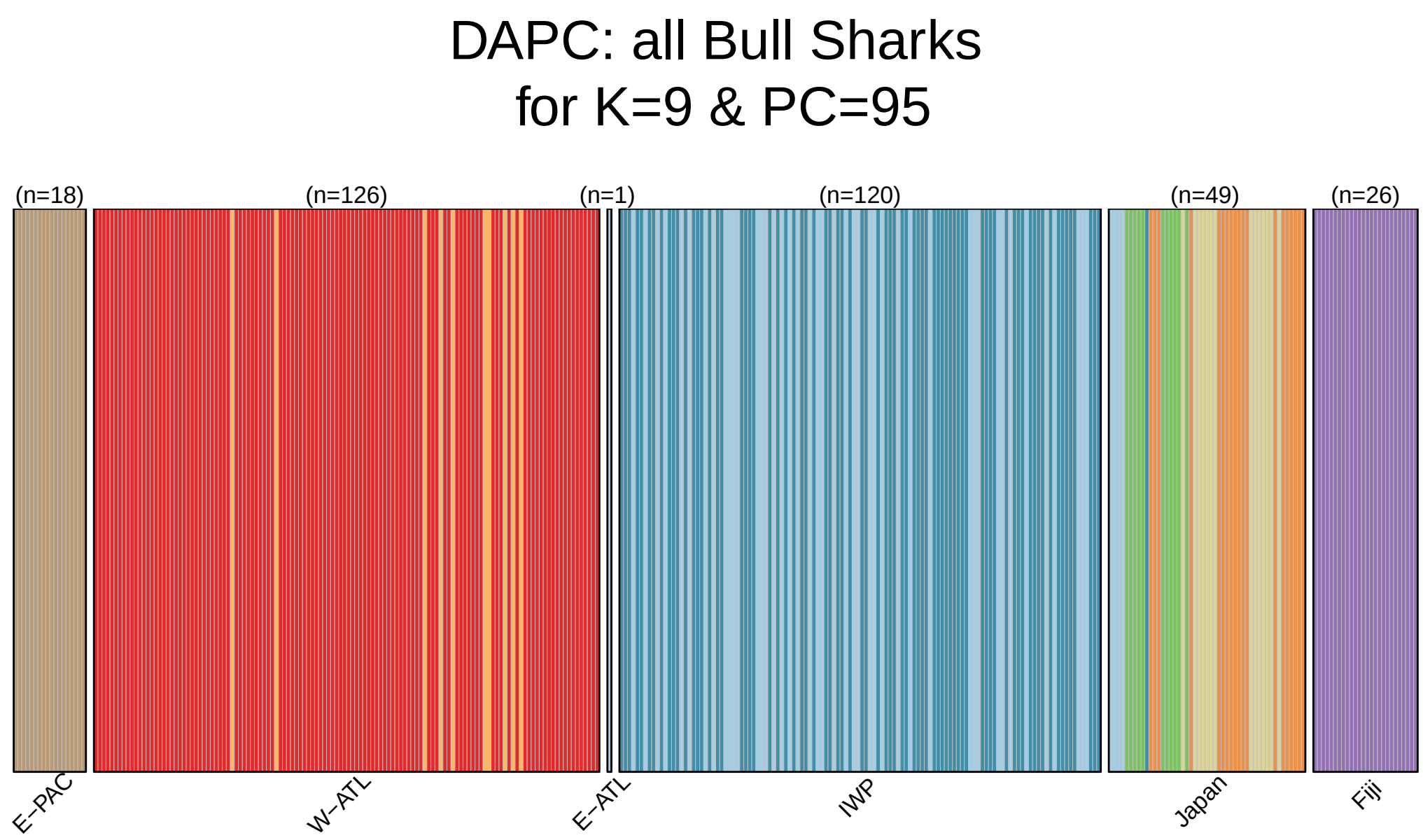
DAPC: all Bull Sharks
for K=9 & PC=95
(n=18)
(n=126)
(n=1)
(n=120)
(n=49)
(n=26)
E–PAC
W–ATL
E–ATL
IWP
Japan
Fiji

DAPC: all Bull Sharks
for K=10 & PC=95

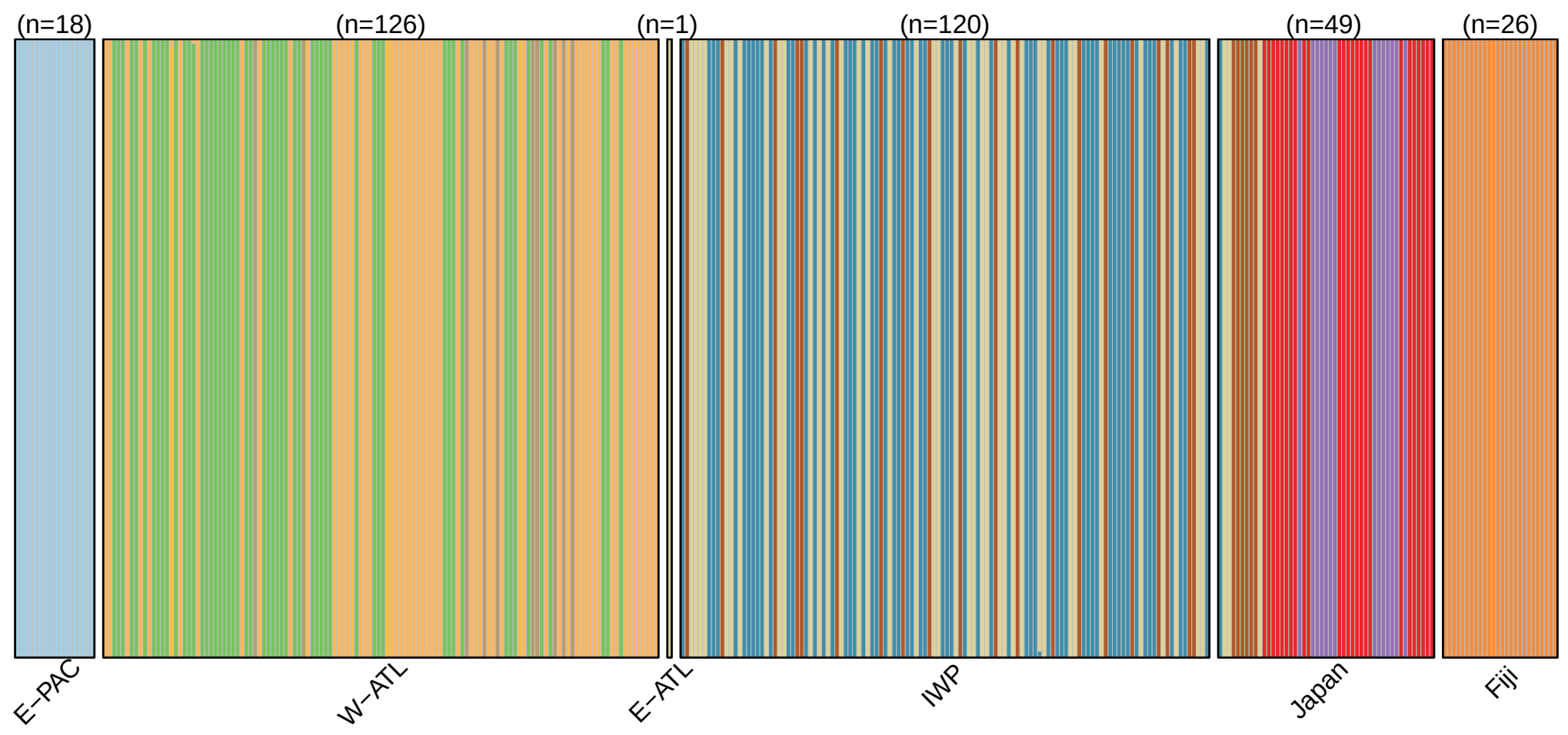


```r
# load("DArTcap_HK_DAPC3.Rdata")
load("DArTcap_HK_DAPC4.Rdata")

# colours.35 <- adegenet::funky(35)

for (K in 3:10) {
  dapc <- get(dapc.all.object.names[K - 1])
  if (K == 2) {
    posi.leg <- "bottomright"
  } else {posi.leg <- "bottomleft"}
  dapc.plot <- ade4::scatter(dapc, grp = pop(BS.dartcap.HK.gl.ref), cex = 2,
                             legend = TRUE, col = colours.21,
                             clabel = FALSE, posi.leg = posi.leg,
                             scree.pca = FALSE, posi.pca = "topleft", cleg = 0.75,
                             xax = 1, yax = 2, inset.solid = 0.3)
  dev.print(
    device = png,
    file = paste0("DArTcap_HK_DAPC_scatterplot4_K", K, ".png"),
    width = 30,
    height = 15,
    units = "cm",
    res = 300
  )
}

for (K in 2:10) {
  colour <- colours.prior[1:K]
  dapc <- get(dapc.all.object.names[K - 1])
  dapc.plot <- ade4::scatter(dapc, cex = 2, legend = TRUE, col = colour,
                             clabel = FALSE, posi.leg = "bottomleft",
                             scree.pca = FALSE, posi.pca = "topleft", cleg = 0.75,
                             xax = 1, yax = 2, inset.solid = 0.3)
  dev.print(
```

```
    device = png,
    file = paste0("DArTcap_HK_DAPC_scatterplot4_pergroups_K",
                  K,".png"),
    width = 30,
    height = 15,
    units = "cm",
    res = 300
  )
}
```

DAPC scatterplot

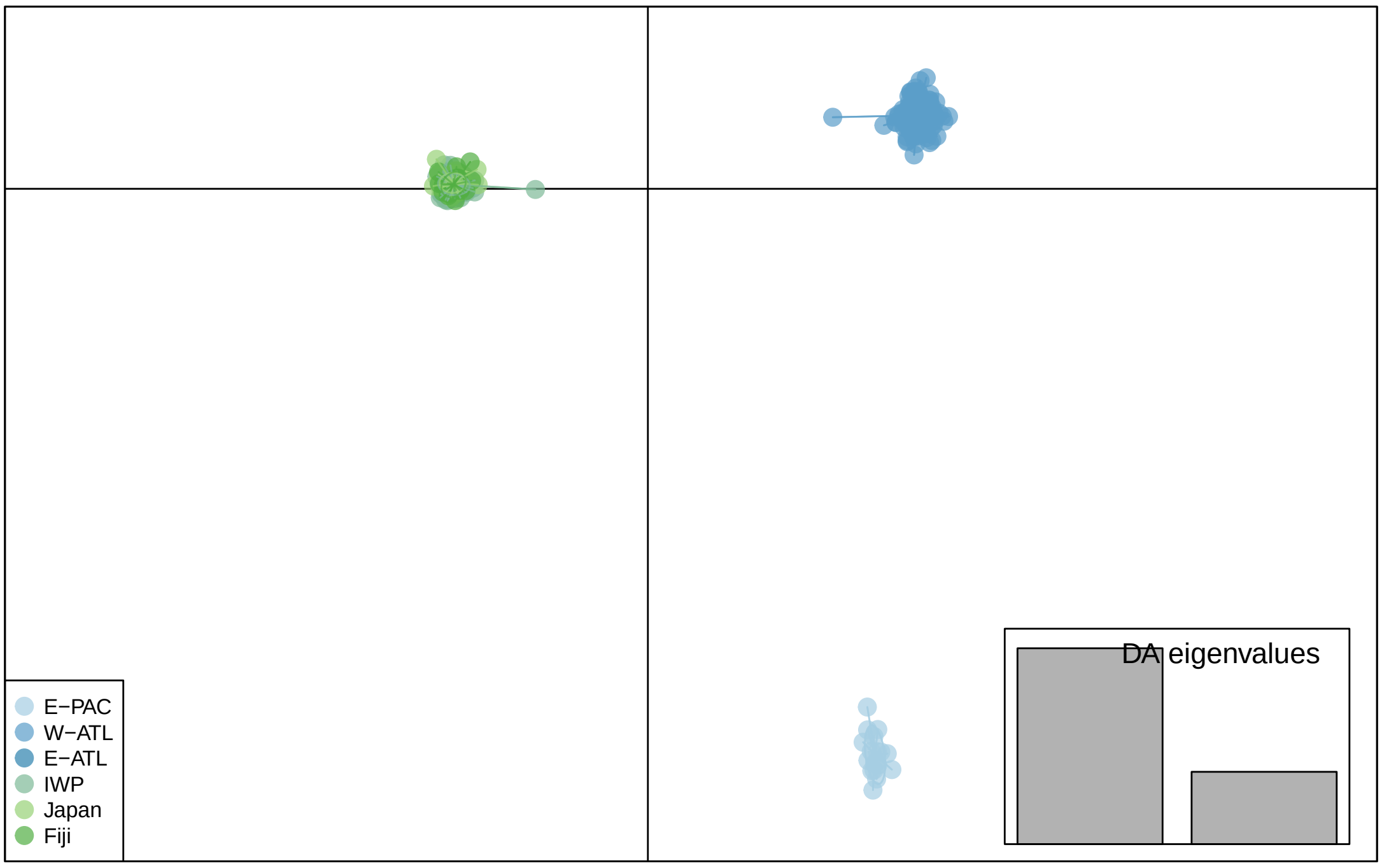

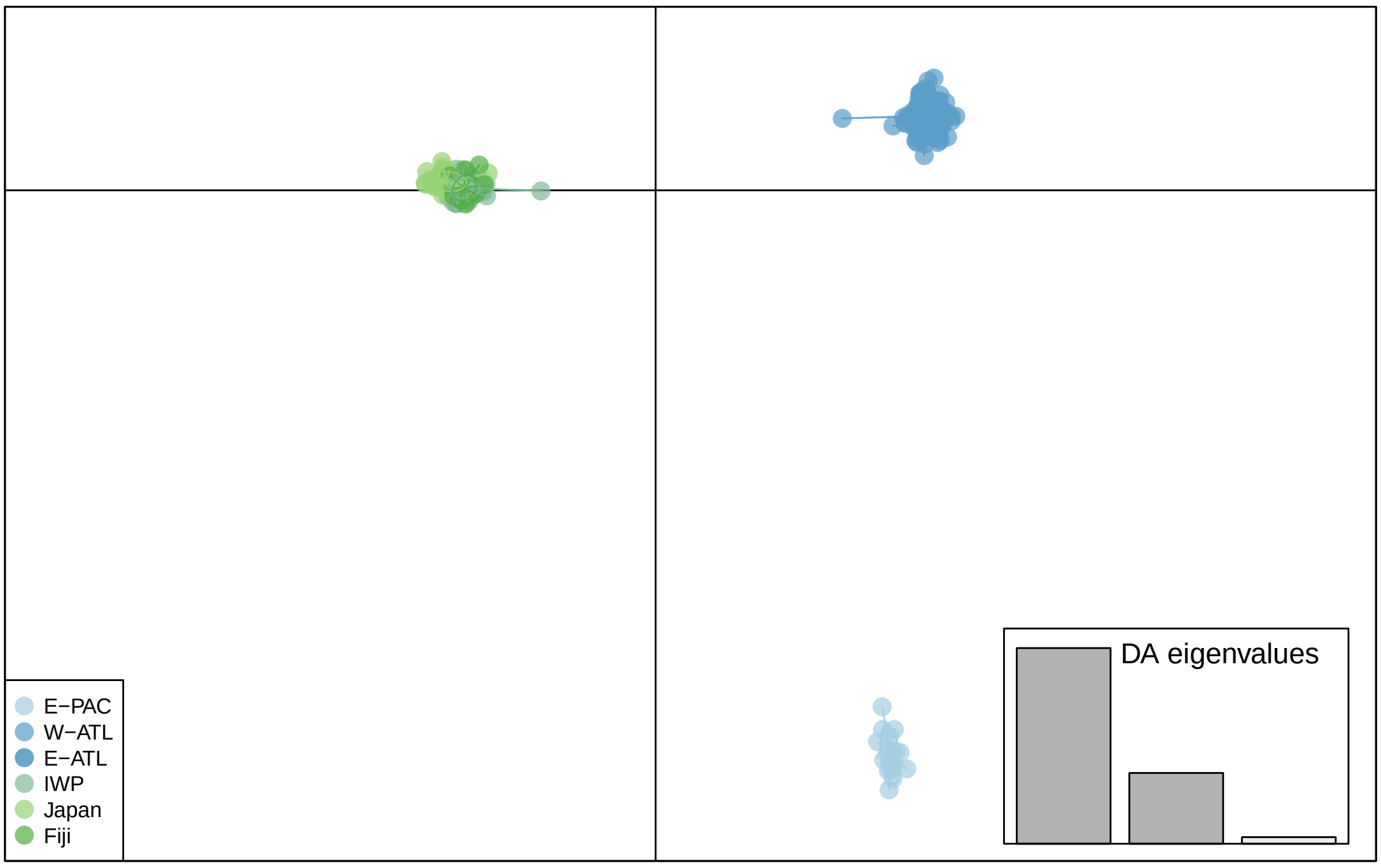

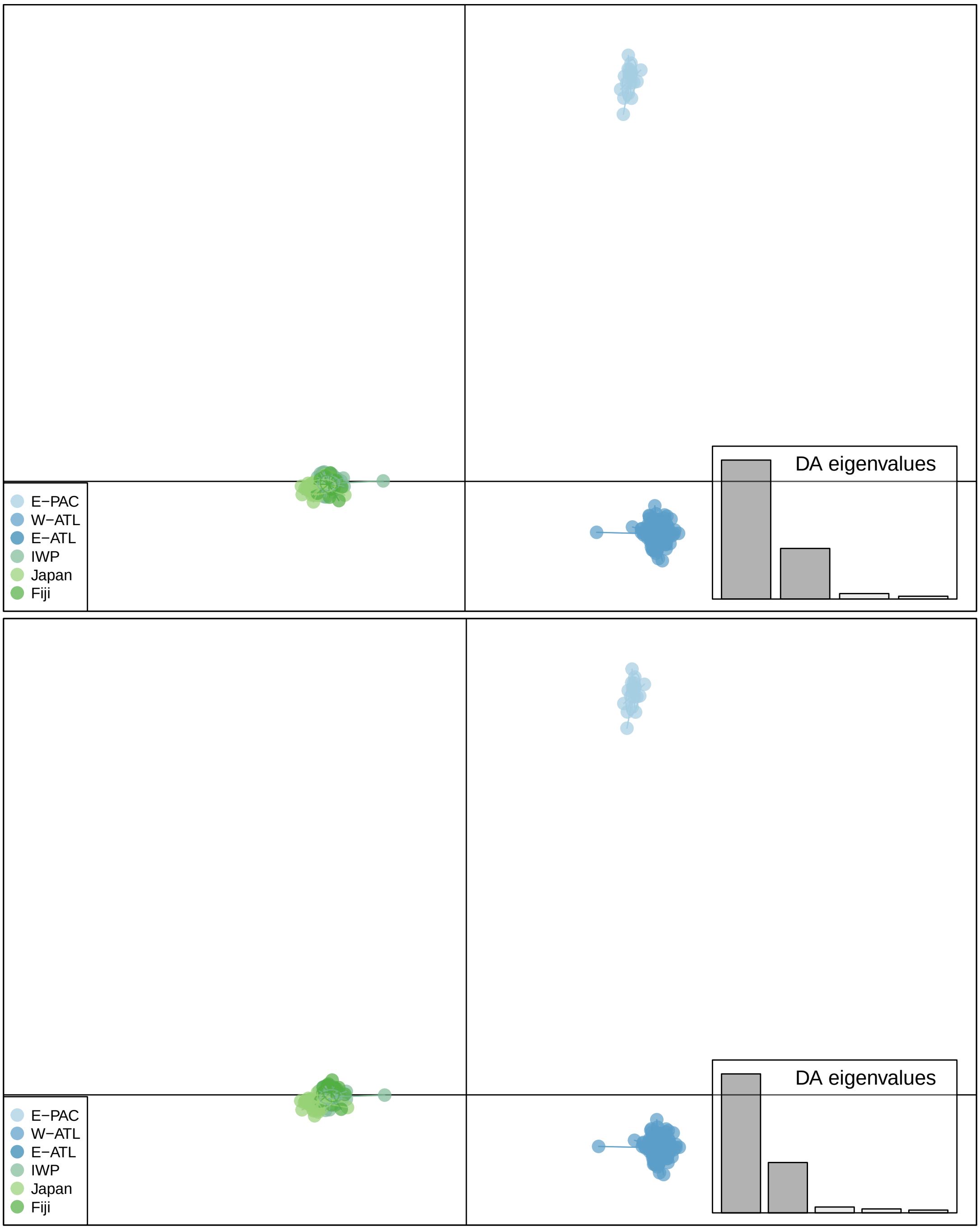
DA eigenvalues
E−PAC
W−ATL
E−ATL
IWP
Japan
Fiji
DA eigenvalues
E−PAC
W−ATL
E−ATL
IWP
Japan
Fiji

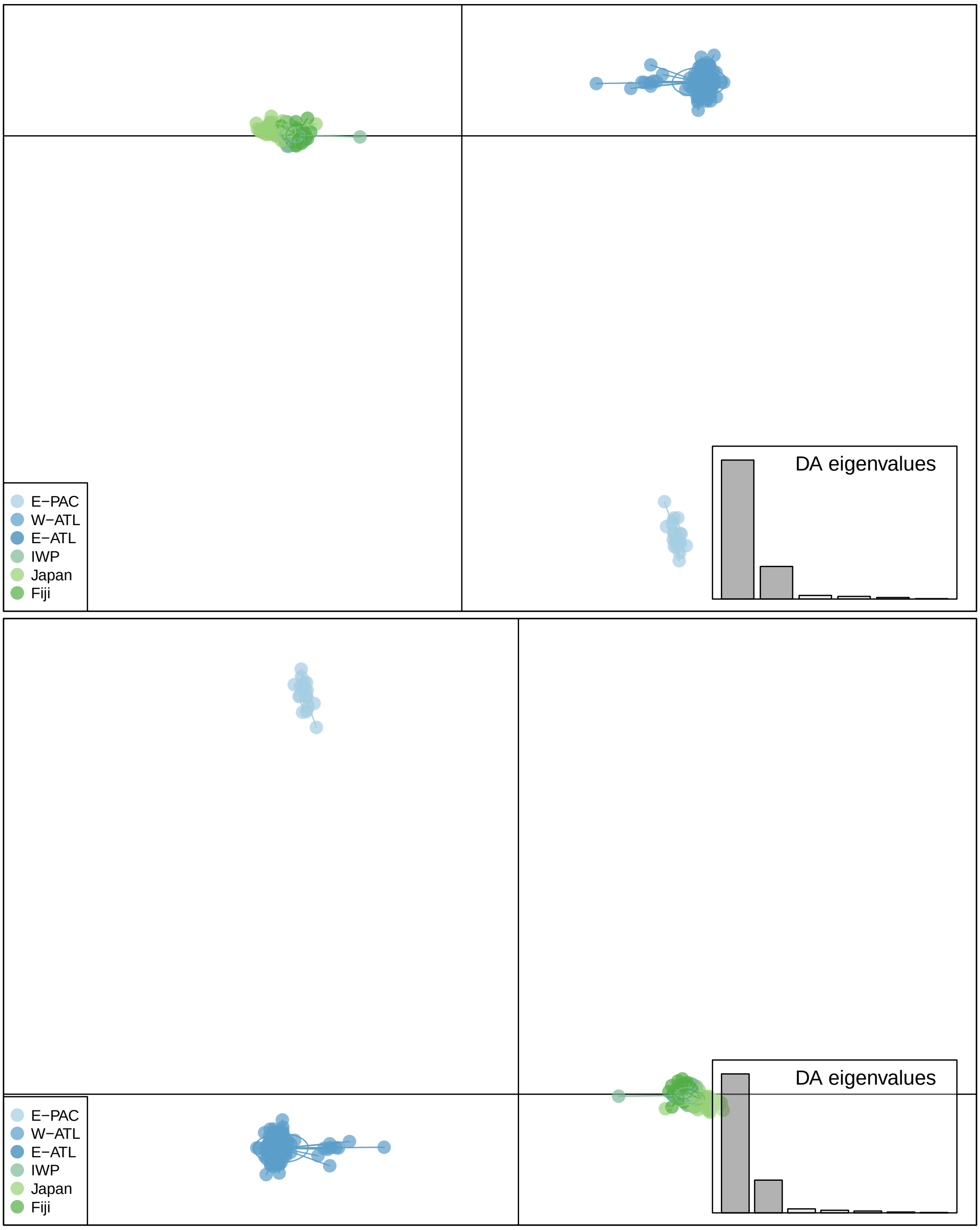

E−PAC
W−ATL
E−ATL
IWP
Japan
Fiji
DA eigenvalues

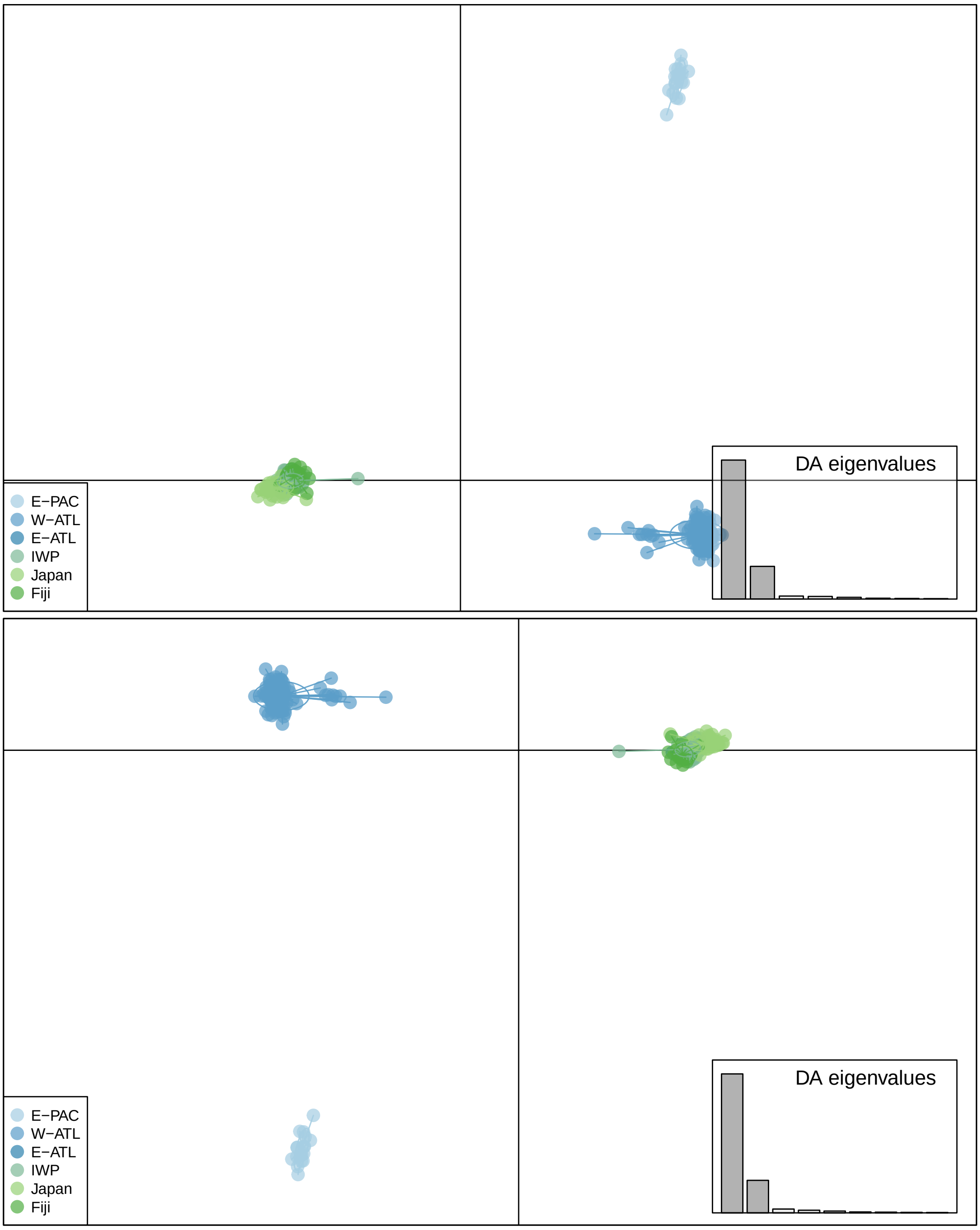
DA eigenvalues
E−PAC
W−ATL
E−ATL
IWP
Japan
Fiji
DA eigenvalues
E−PAC
W−ATL
E−ATL
IWP
Japan
Fiji

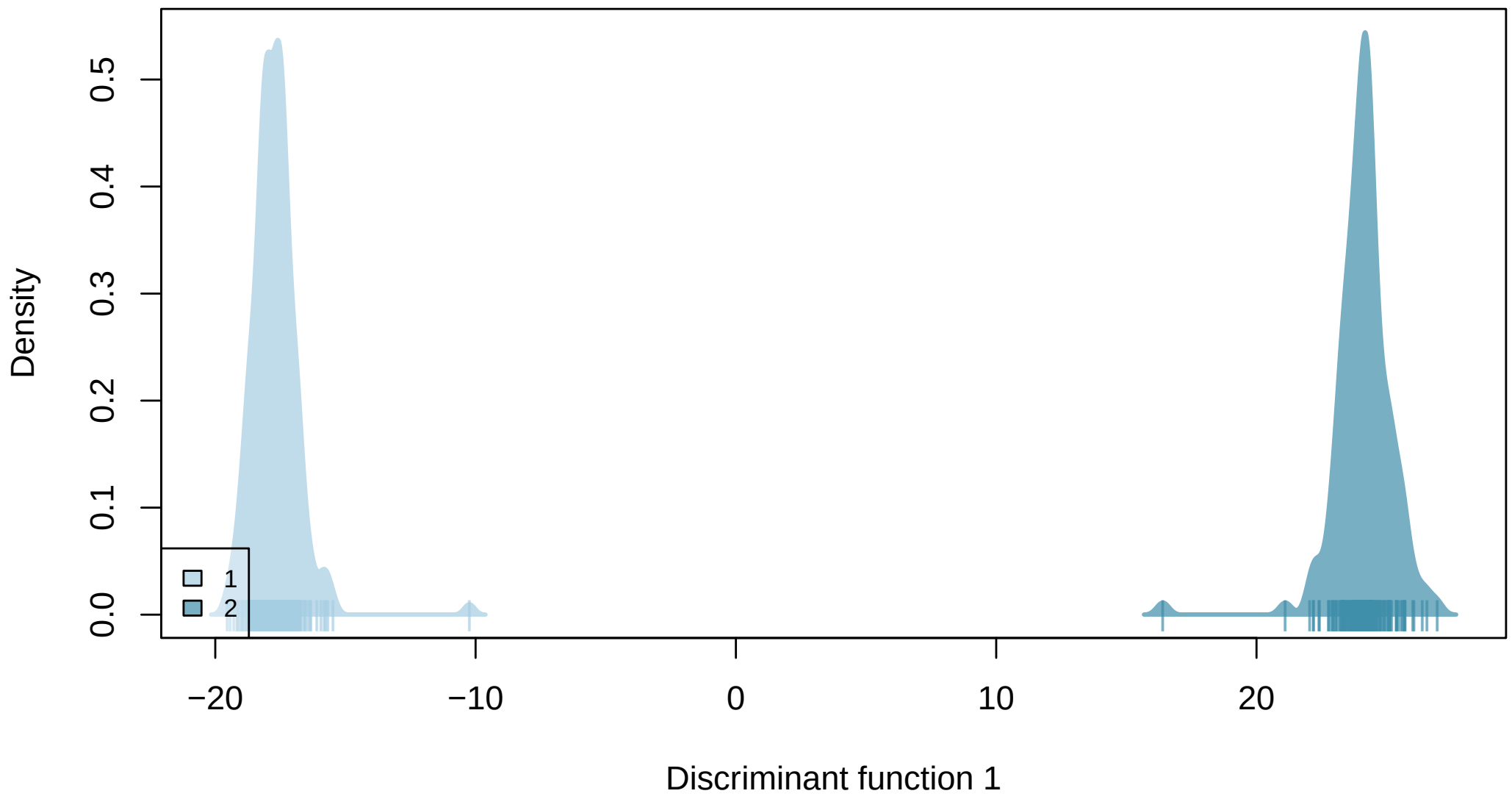

Density
0.0
0.1
0.2
0.3
0.4
0.5
1
2
−20
−10
0
10
20
Discriminant function 1

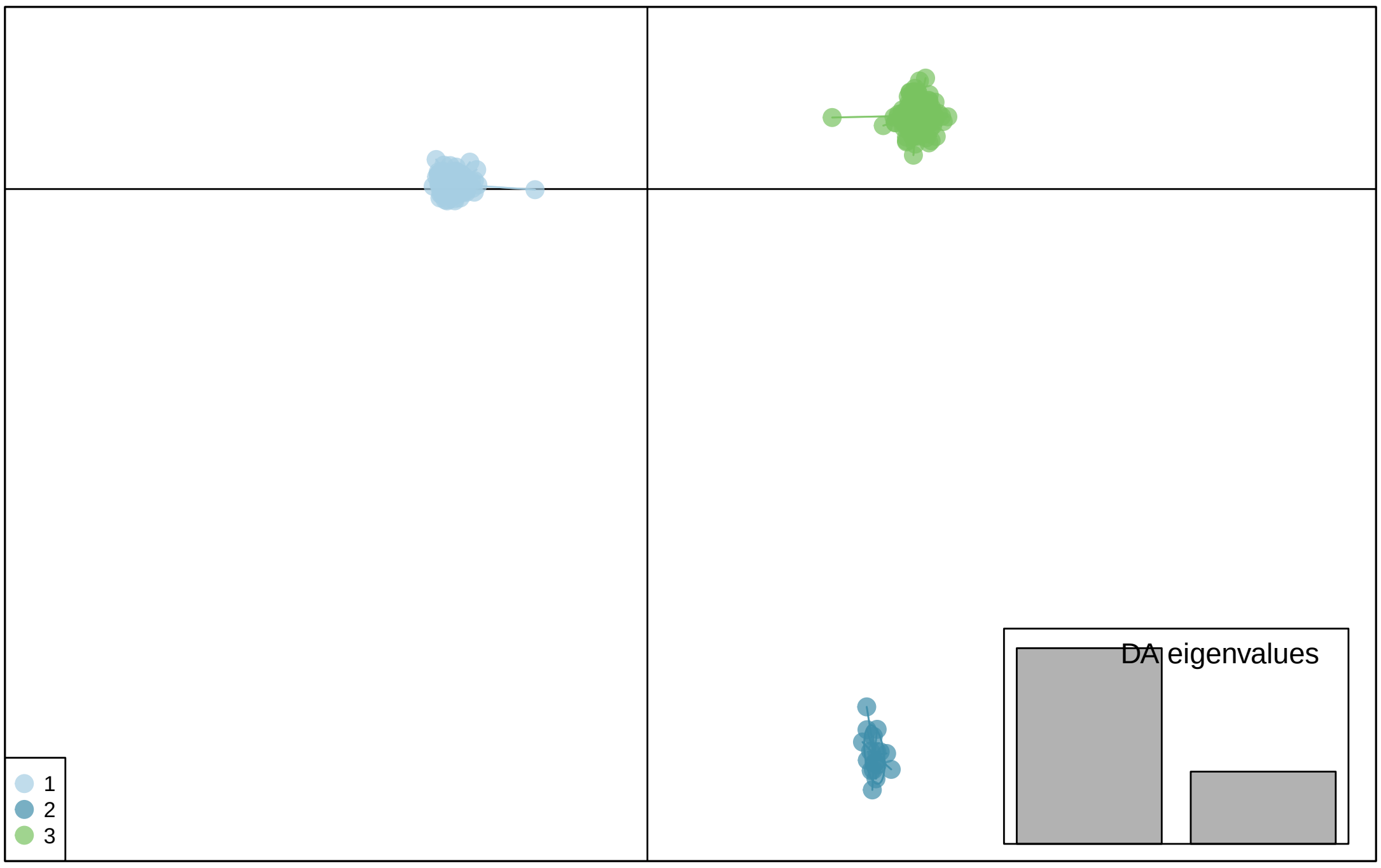

DA eigenvalues
1
2
3

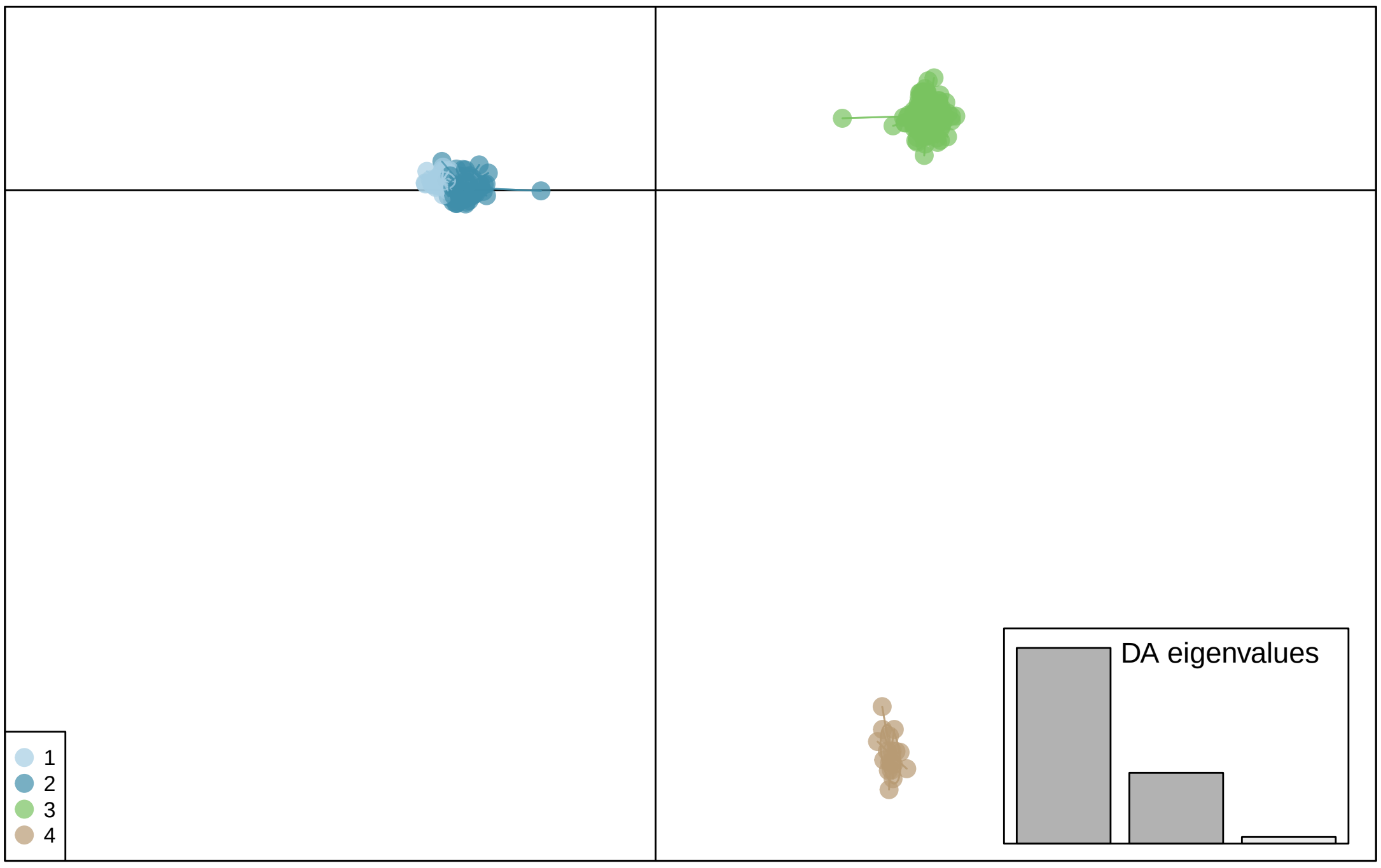
DA eigenvalues
1
2
3
4

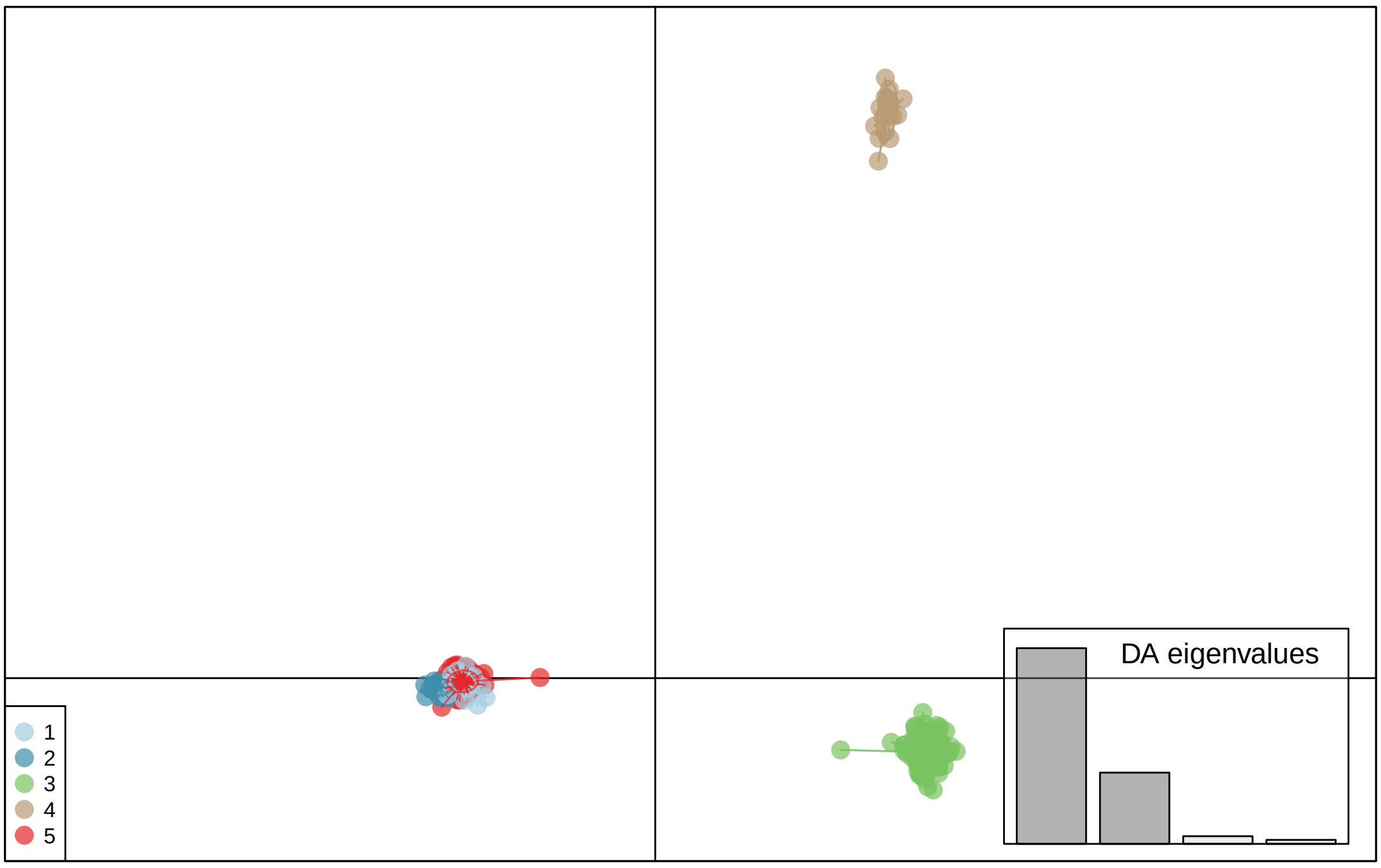
DA eigenvalues
1
2
3
4
5

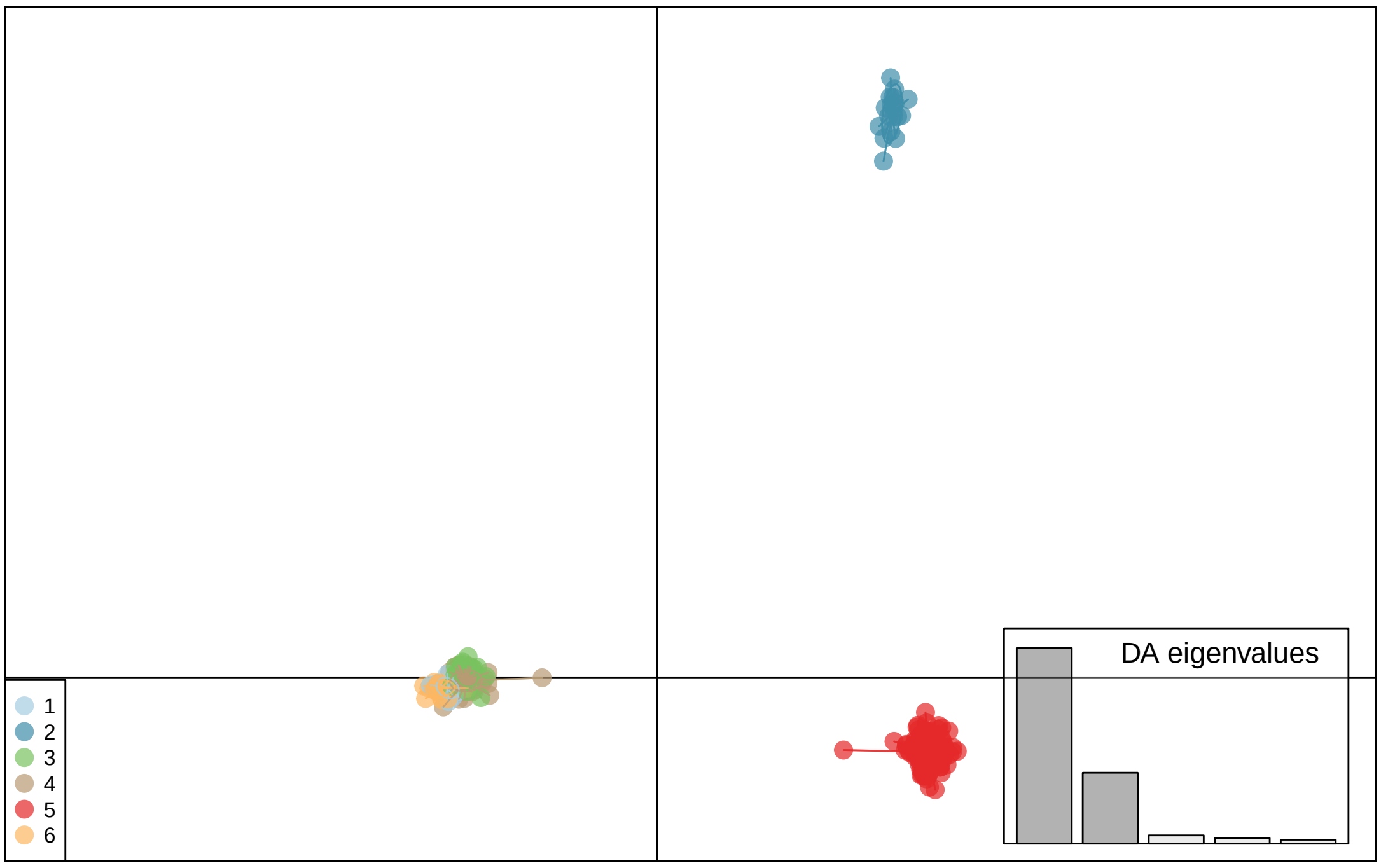
1
2
3
4
5
6
DA eigenvalues

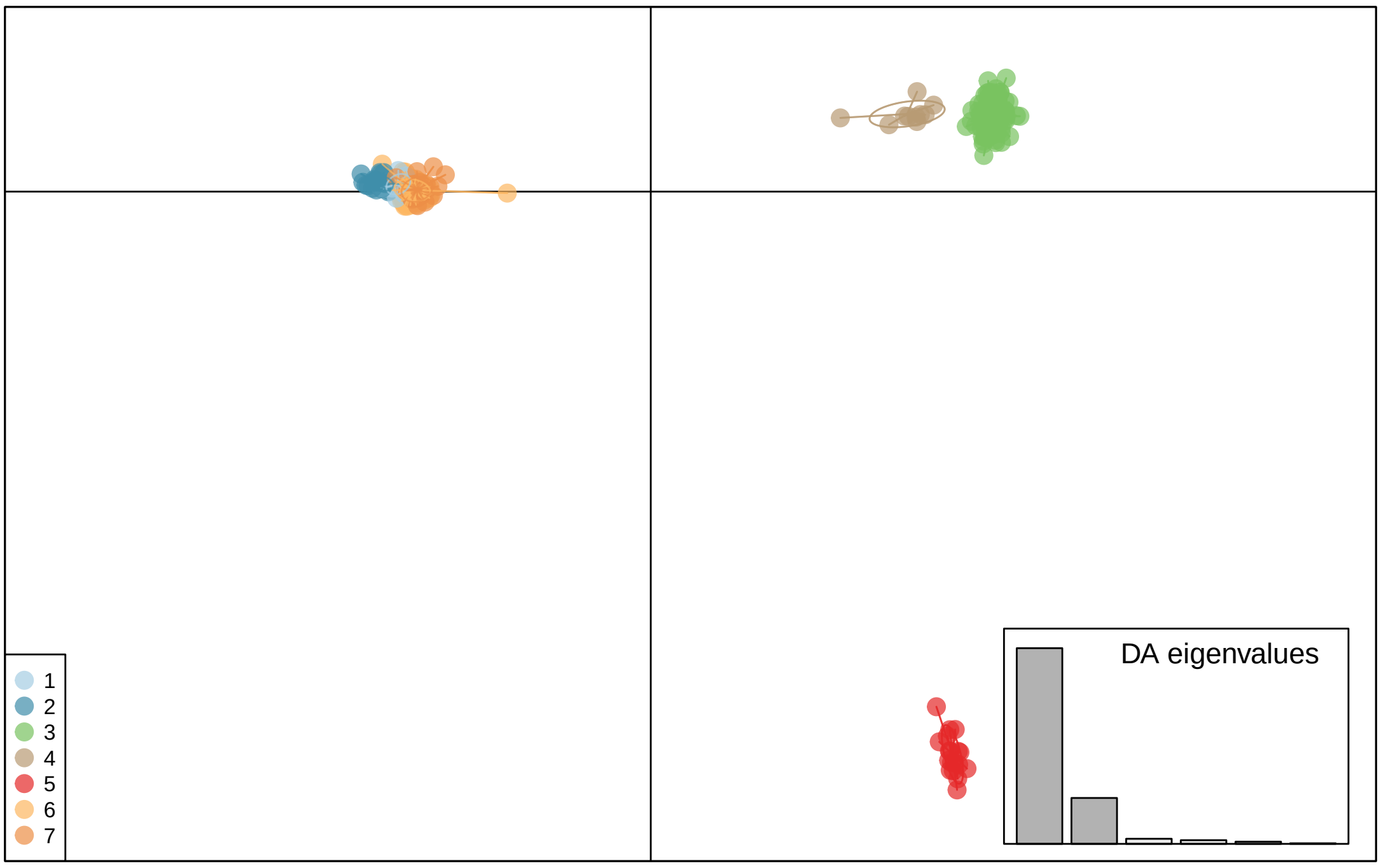
1
2
3
4
5
6
7
DA eigenvalues

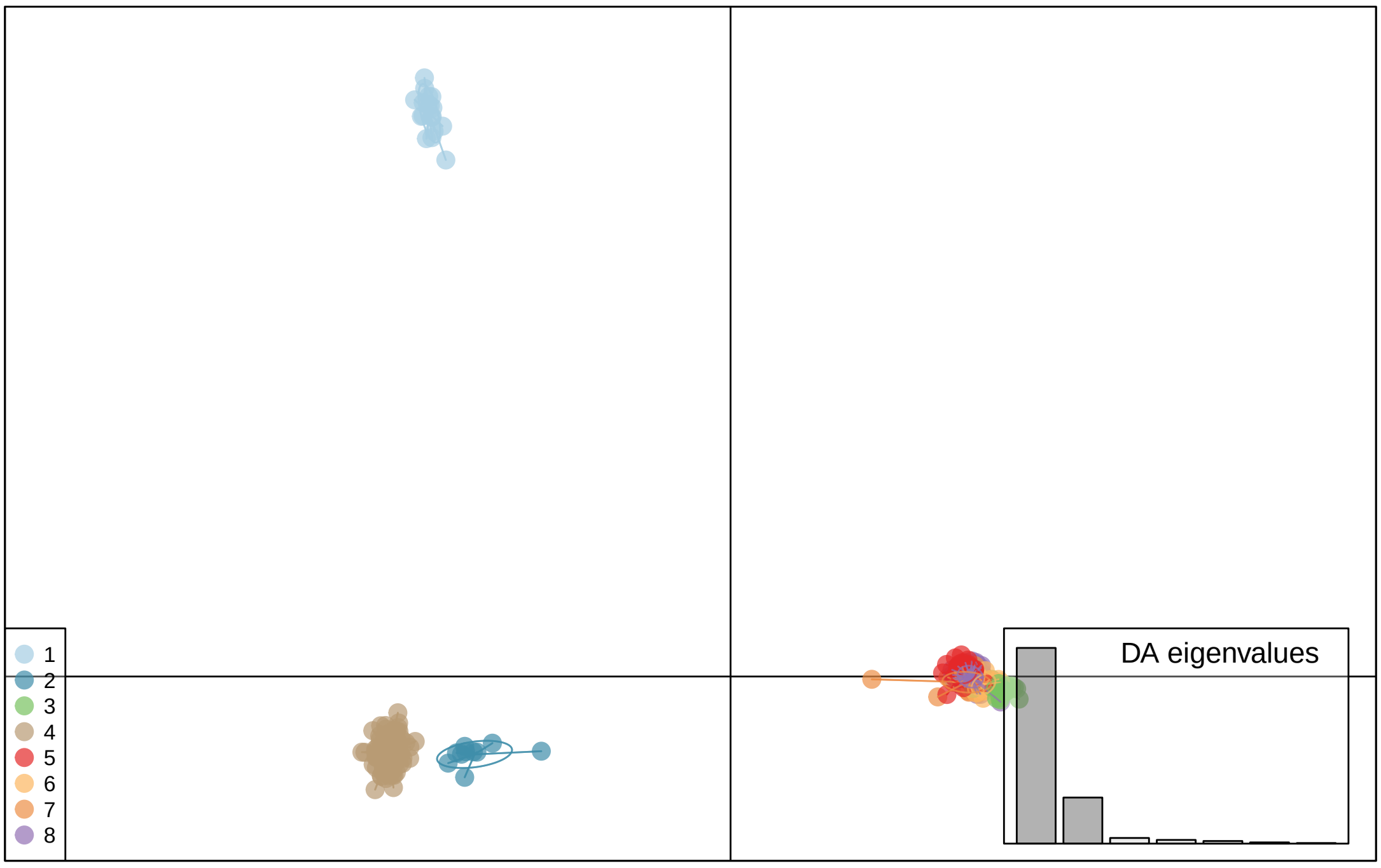
1
2
3
4
5
6
7
8
DA eigenvalues

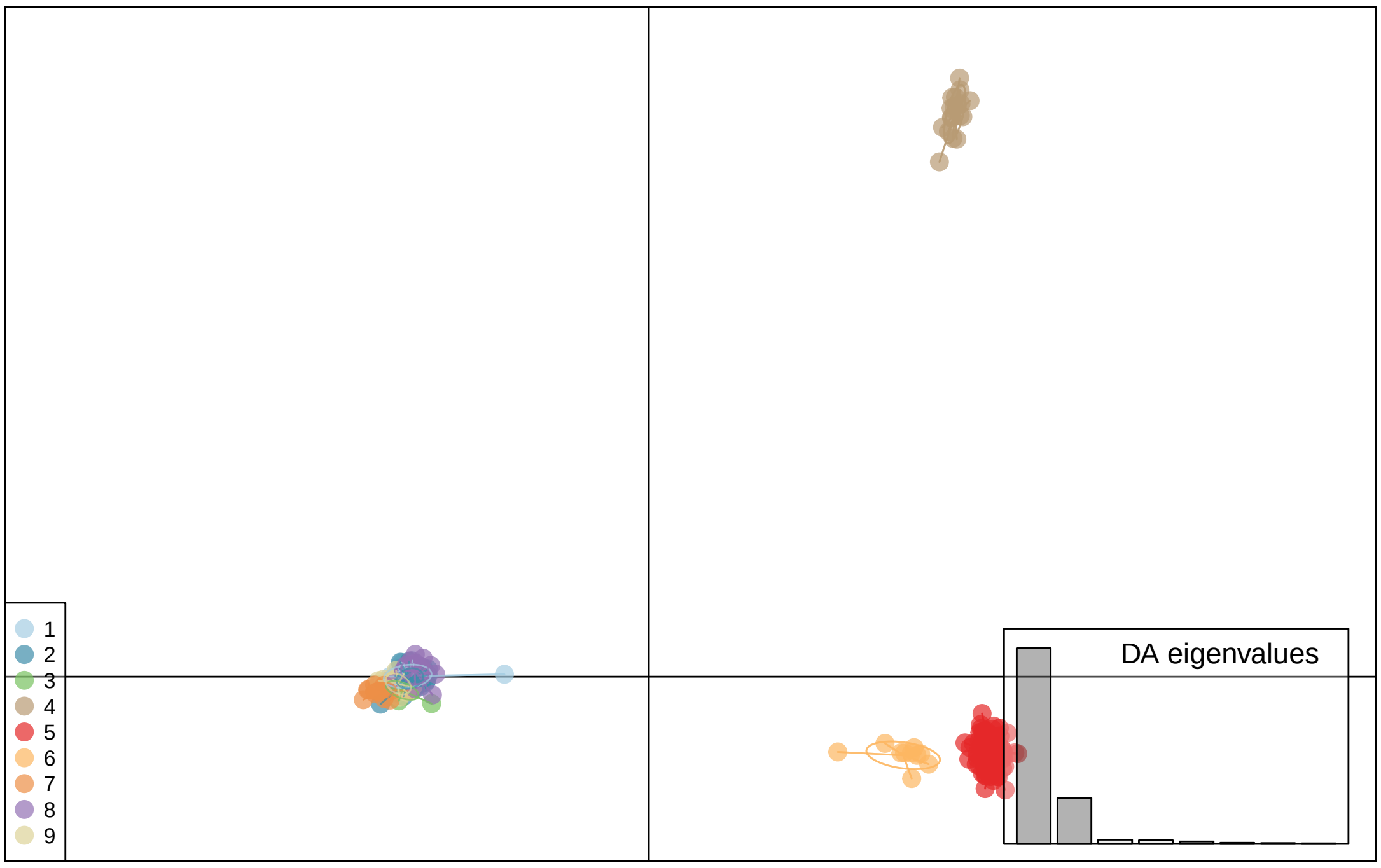
1
2
3
4
5
6
7
8
9
DA eigenvalues

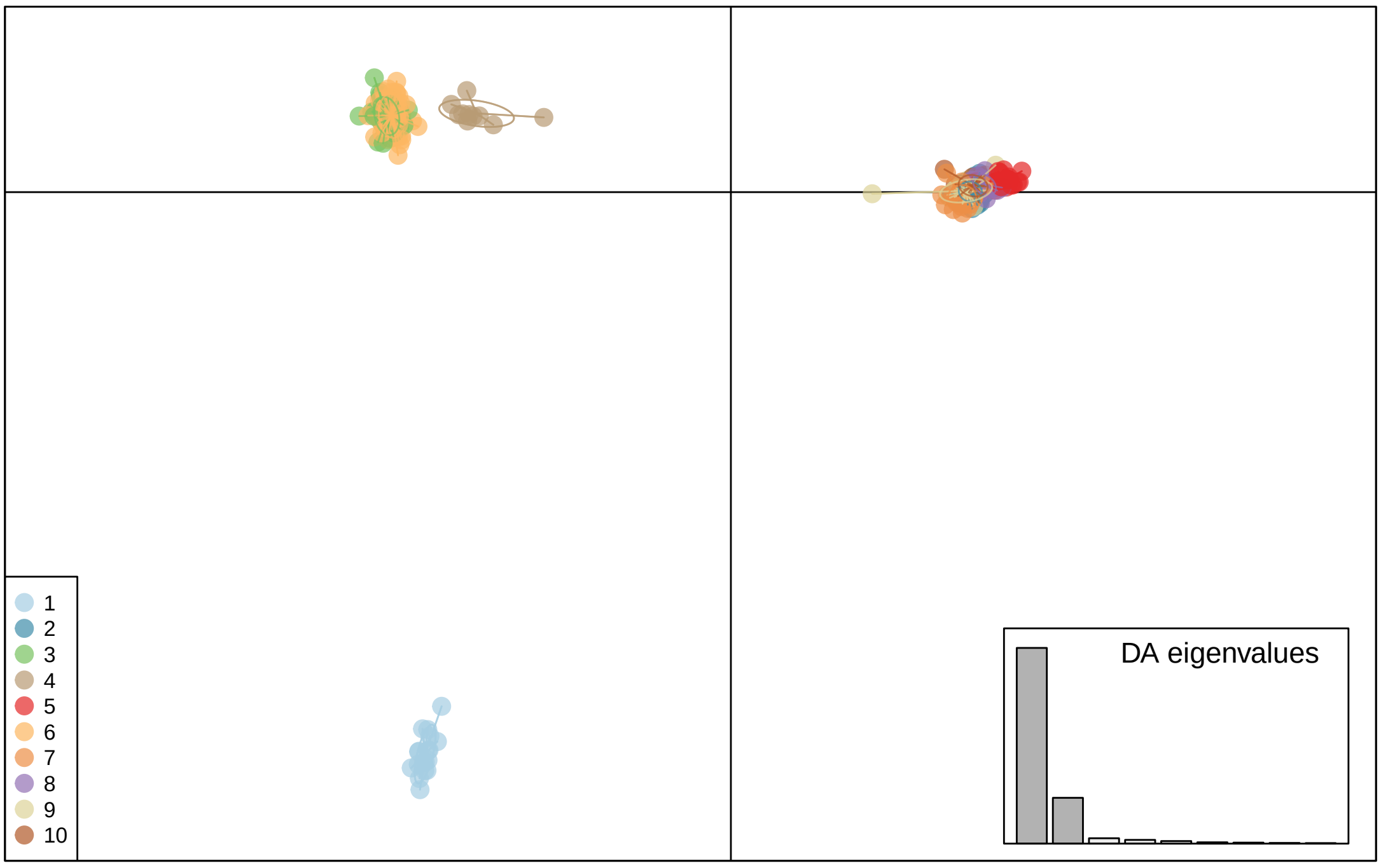

DAPC scatterplot - FLO This code will *colour* individuals according to *sampling location* and **group** individuals according to DAPC **posterior membership**.

```
for (K in 3:10) {
  dapc <- get(dapc.all.object.names[K - 1])
  x <- dapc$ind.coord[,1]
  y <- dapc$ind.coord[,2]
  group <- paste("Group", as.numeric(dapc$assign))
  pop <- BS.dartcap.HK.gl.ref$pop
  df <- data.frame(x, y, group, pop)
  colnames(df) <- c("x","y","group","pop")
  gg <- merge(df,aggregate(cbind(mean.x = x, mean.y = y)~group, df, mean),
              by = "group")

  plot <- ggplot2::ggplot(gg, ggplot2::aes(x,y,color = pop)) +
    ggplot2::geom_point(size = 5) +
    ggplot2::geom_point(ggplot2::aes(x = mean.x, y = mean.y), size = 5, shape = 17,
                        color = "black") +
    ggplot2::geom_segment(ggplot2::aes(x = mean.x, y = mean.y, xend = x, yend = y)) +
    # ggplot2::scale_colour_manual(values = colours.21) +
    # ggplot2::scale_fill_manual(values = colours.21) +
    ggplot2::scale_colour_manual(values = colours.6) +
    ggplot2::scale_fill_manual(values = colours.6) +
    ggplot2::geom_hline(yintercept = 0) +
    ggplot2::geom_vline(xintercept = 0) +
    ggplot2::labs(subtitle = "",
         y = "PC2",
         x = "PC1",
         title = paste0("K=",K," & PC=",PC, sep = ""),
         caption = "") +
    ggplot2::theme_minimal() +
    ggplot2::theme(
      axis.text = ggplot2::element_text(size = 5),
      axis.title.x = ggplot2::element_text(size = 10),
      axis.title.y = ggplot2::element_text(size = 10),
```

```
      legend.text = ggplot2::element_text(size = 7))
  print(plot)
  ggplot2::ggsave(plot,
                  filename = paste0("DArTcap_HK_DAPC_scatterplot4_K", K, ".png"),
                  width = 30, height = 15,units = "cm")
}
```

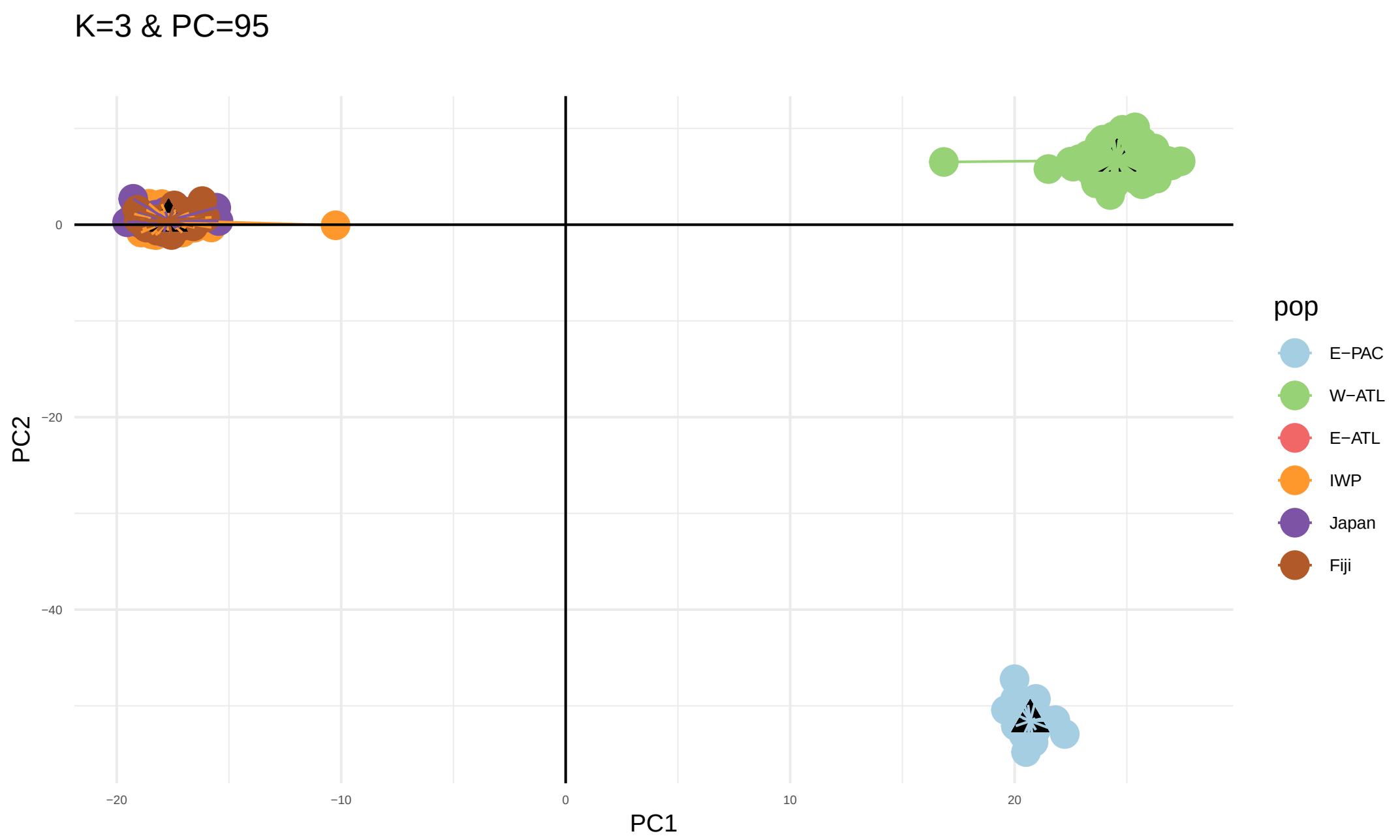

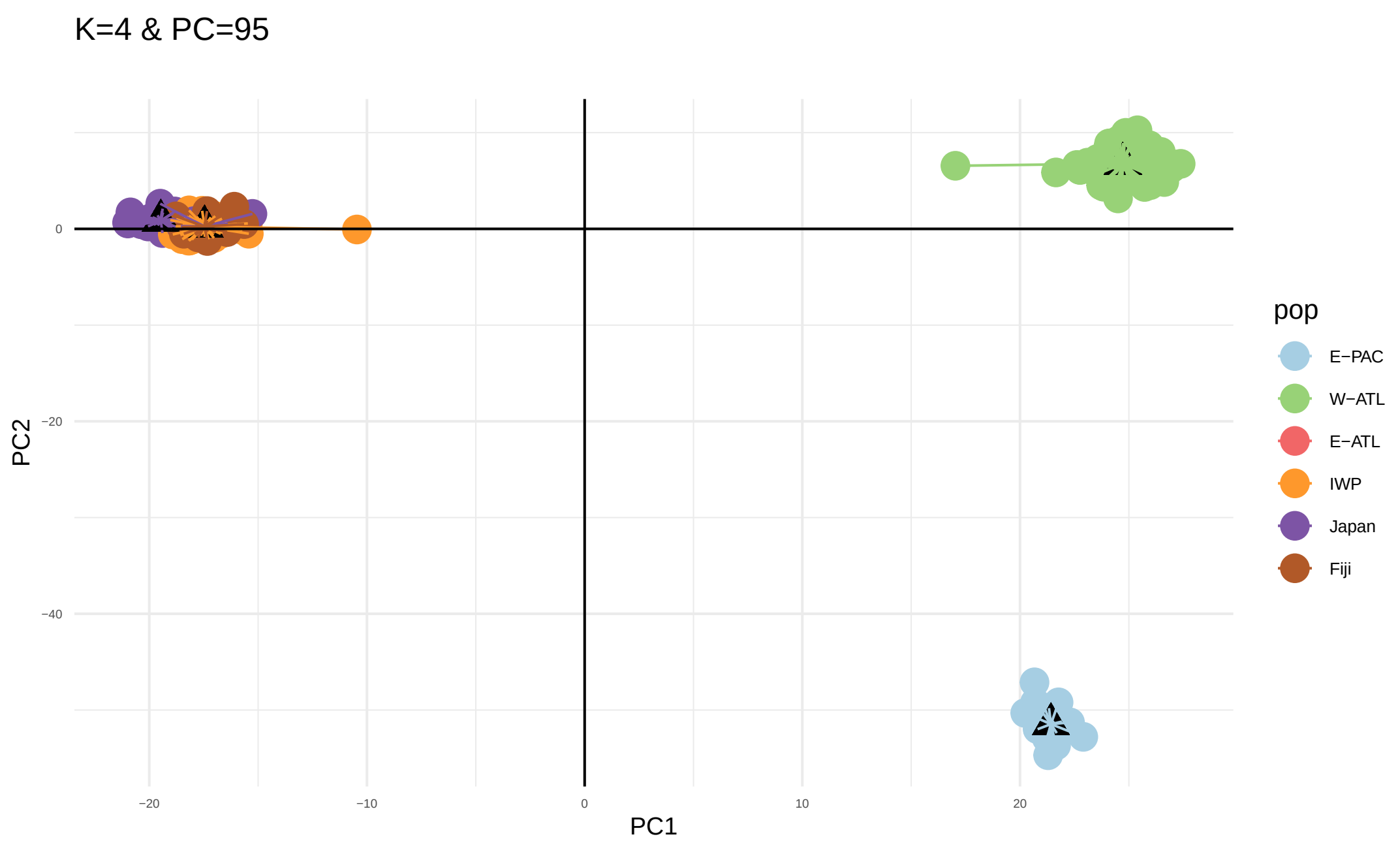

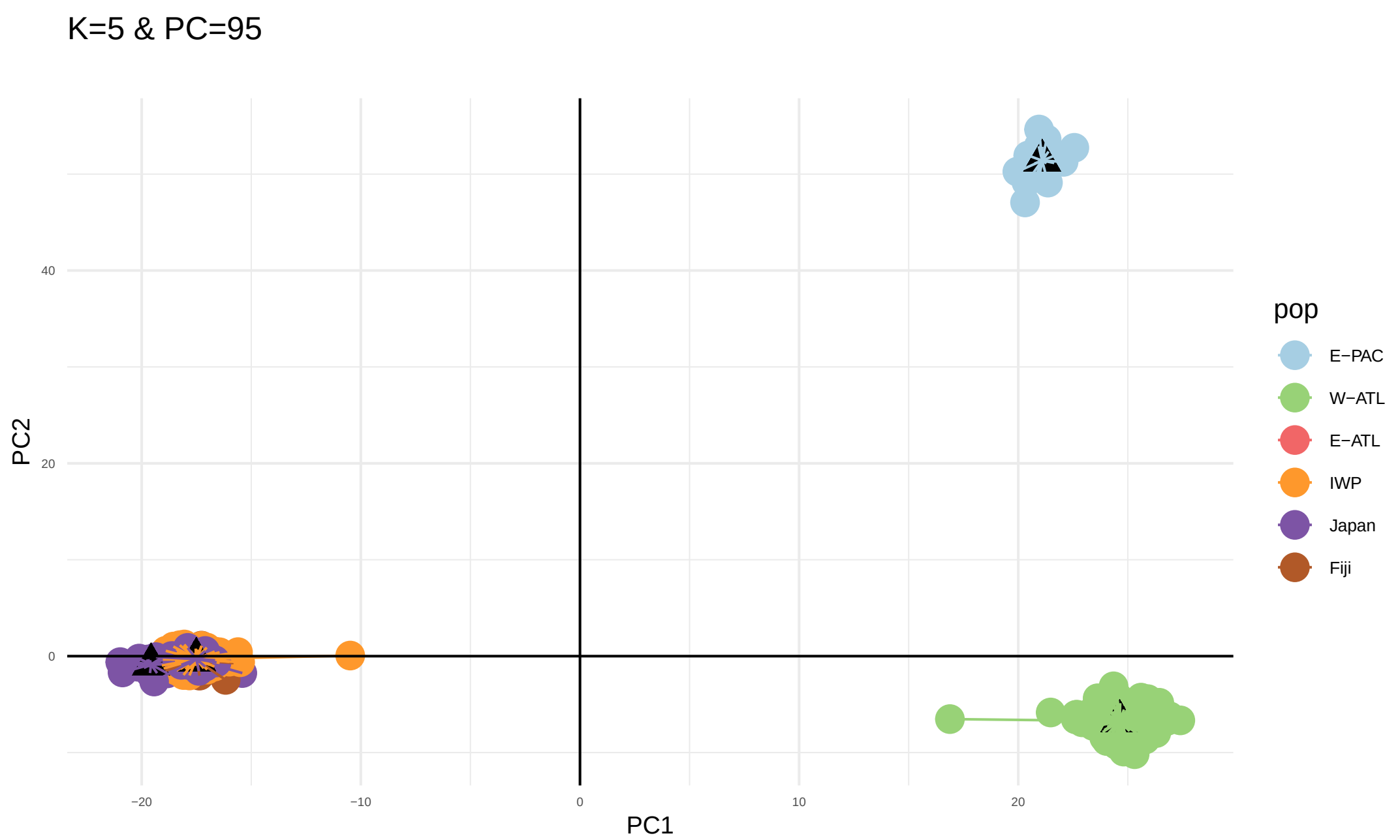
K=5 & PC=95
PC2
PC1
40
20
0
−20
−10
0
10
20
pop
E−PAC
W−ATL
E−ATL
IWP
Japan
Fiji

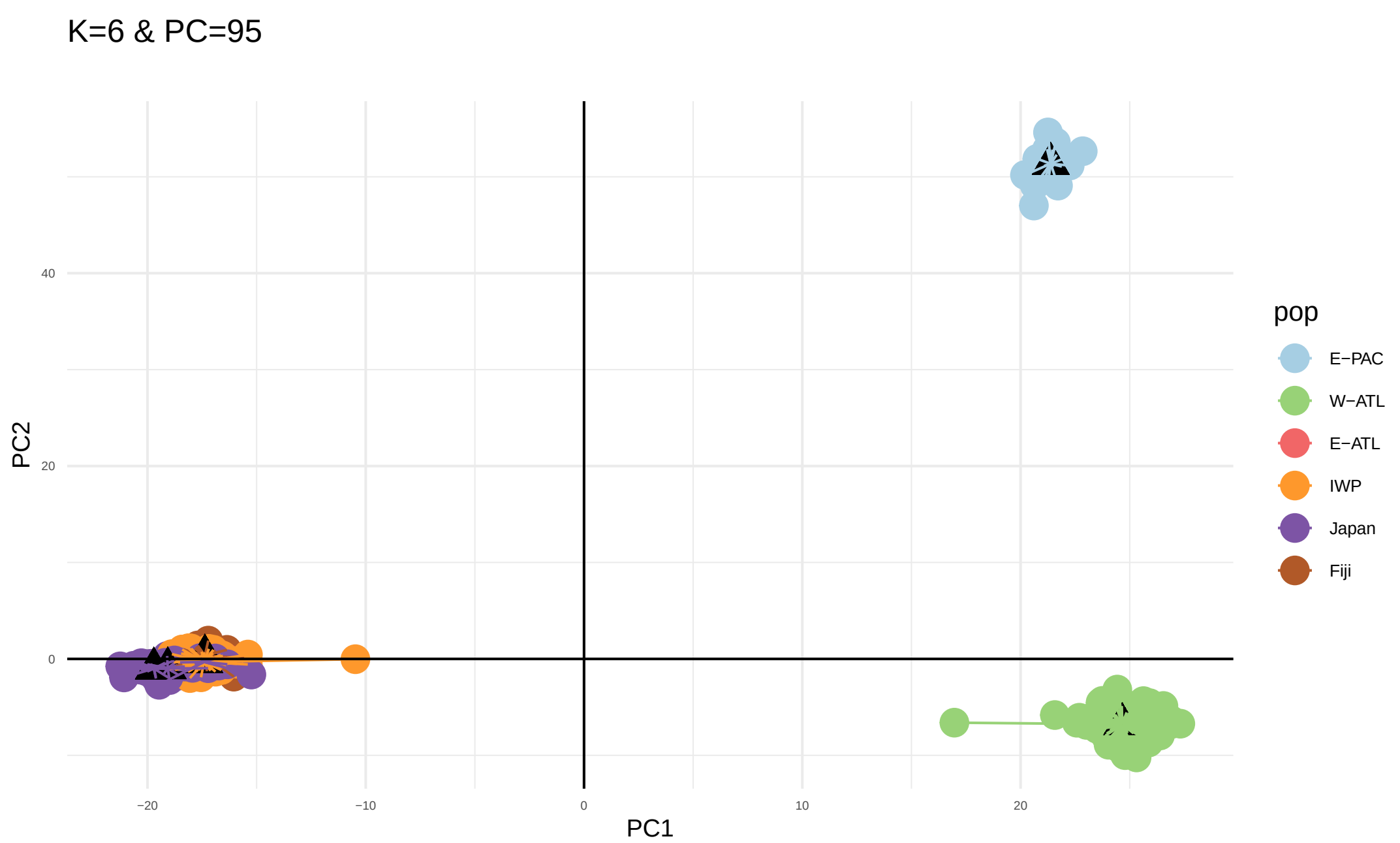
K=6 & PC=95
PC2
PC1
40
20
0
−20
−10
0
10
20
pop
E−PAC
W−ATL
E−ATL
IWP
Japan
Fiji

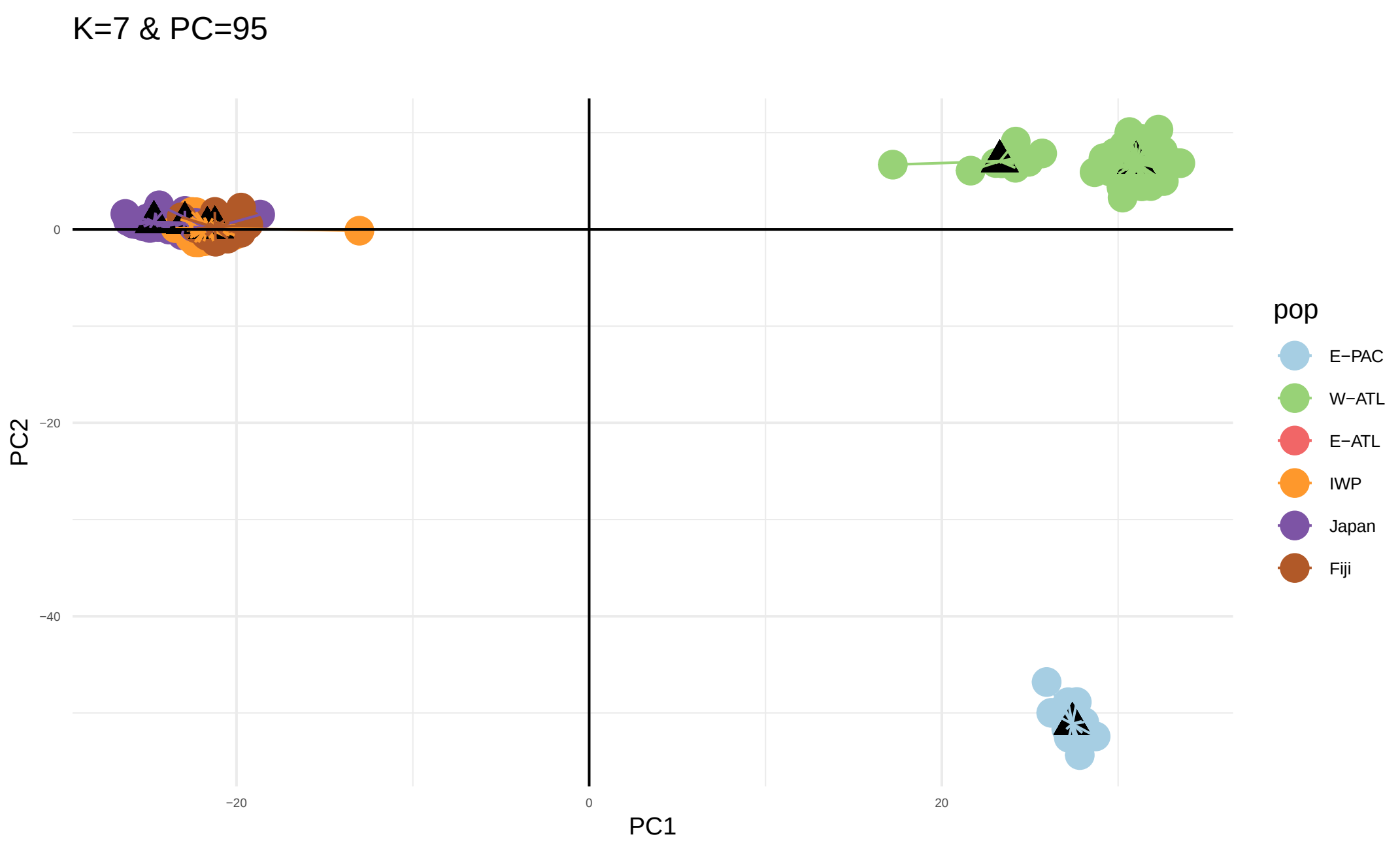
K=7 & PC=95
PC2
PC1
0
−20
−40
−20
0
20
pop
E−PAC
W−ATL
E−ATL
IWP
Japan
Fiji

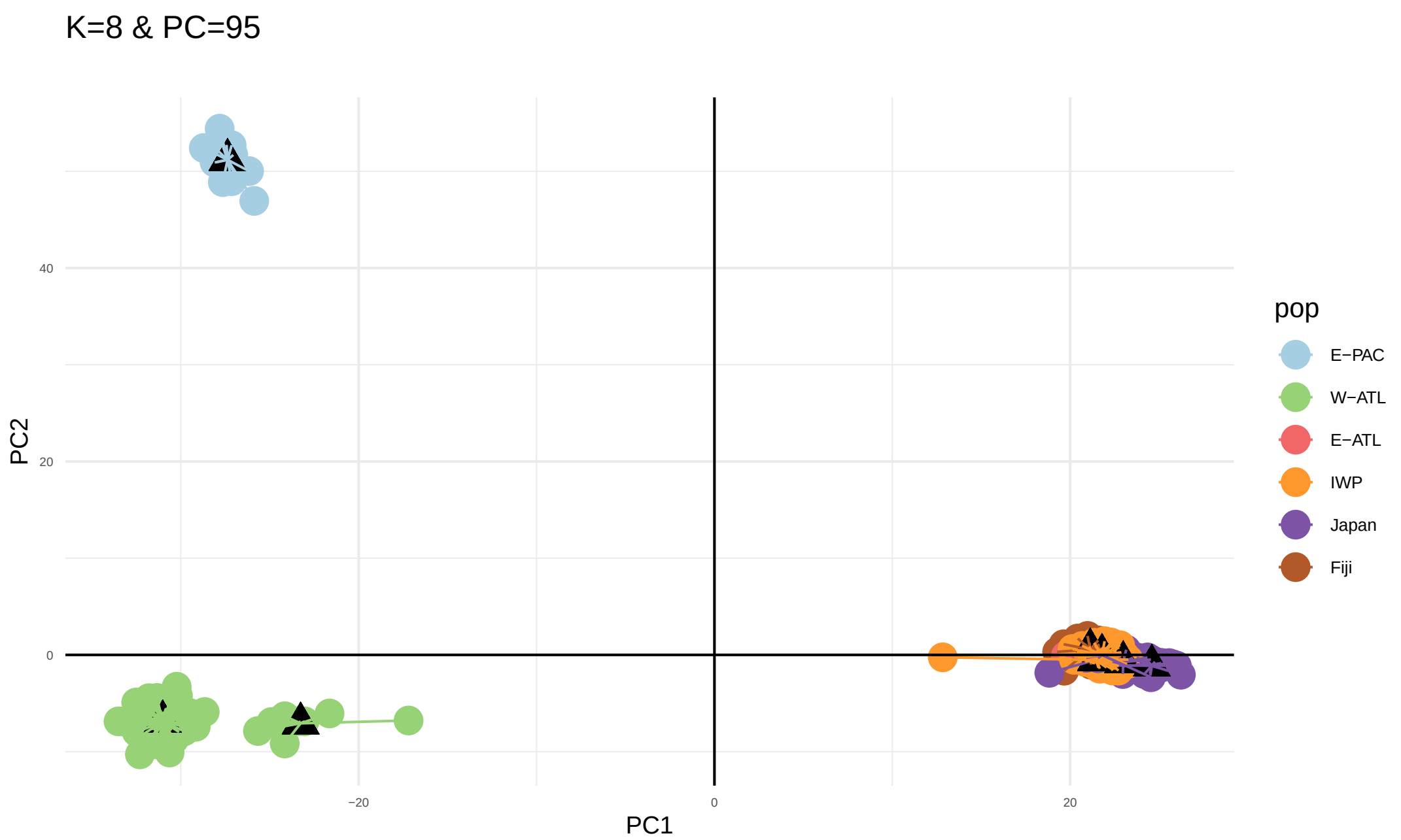
K=8 & PC=95
PC2
PC1
40
20
0
−20
0
20
pop
E−PAC
W−ATL
E−ATL
IWP
Japan
Fiji

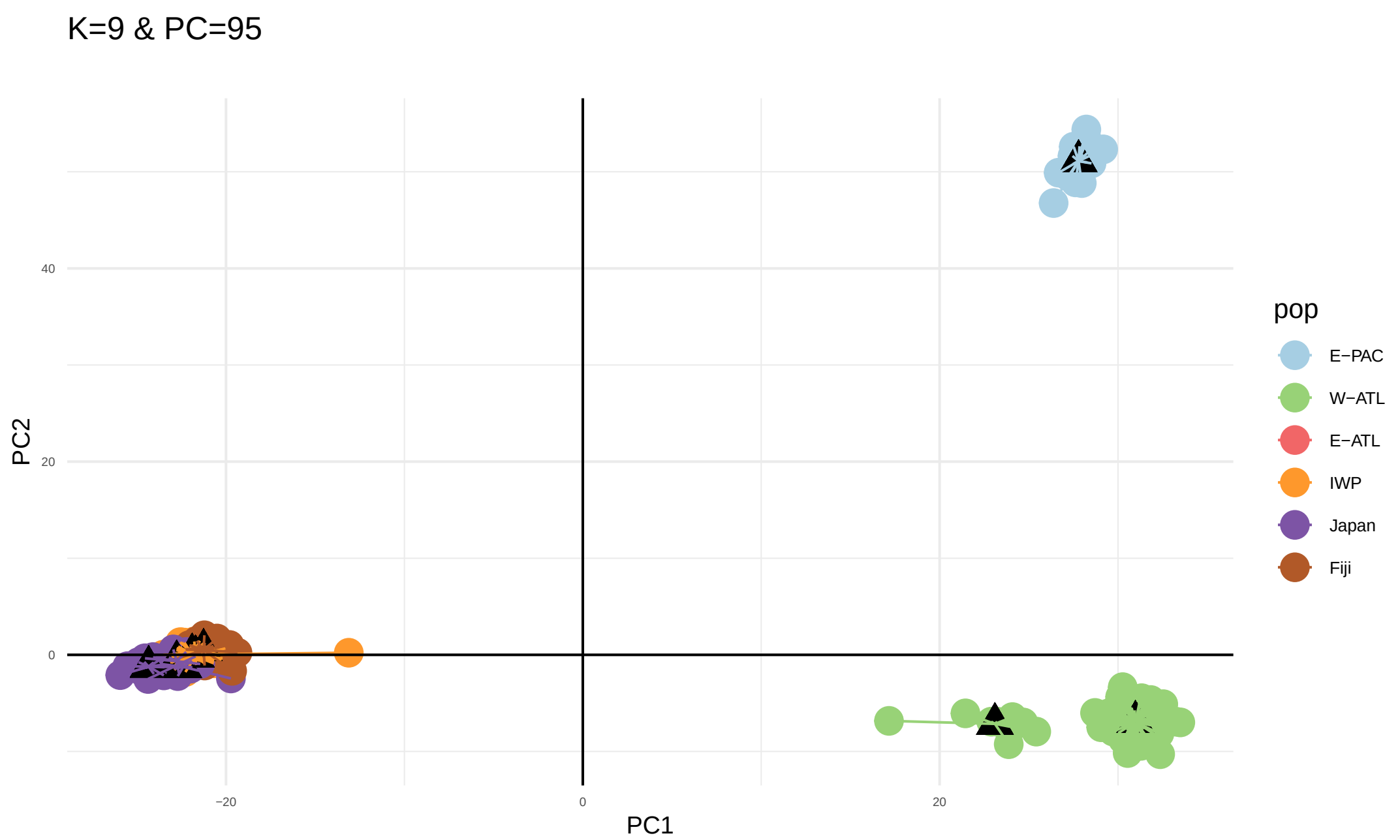

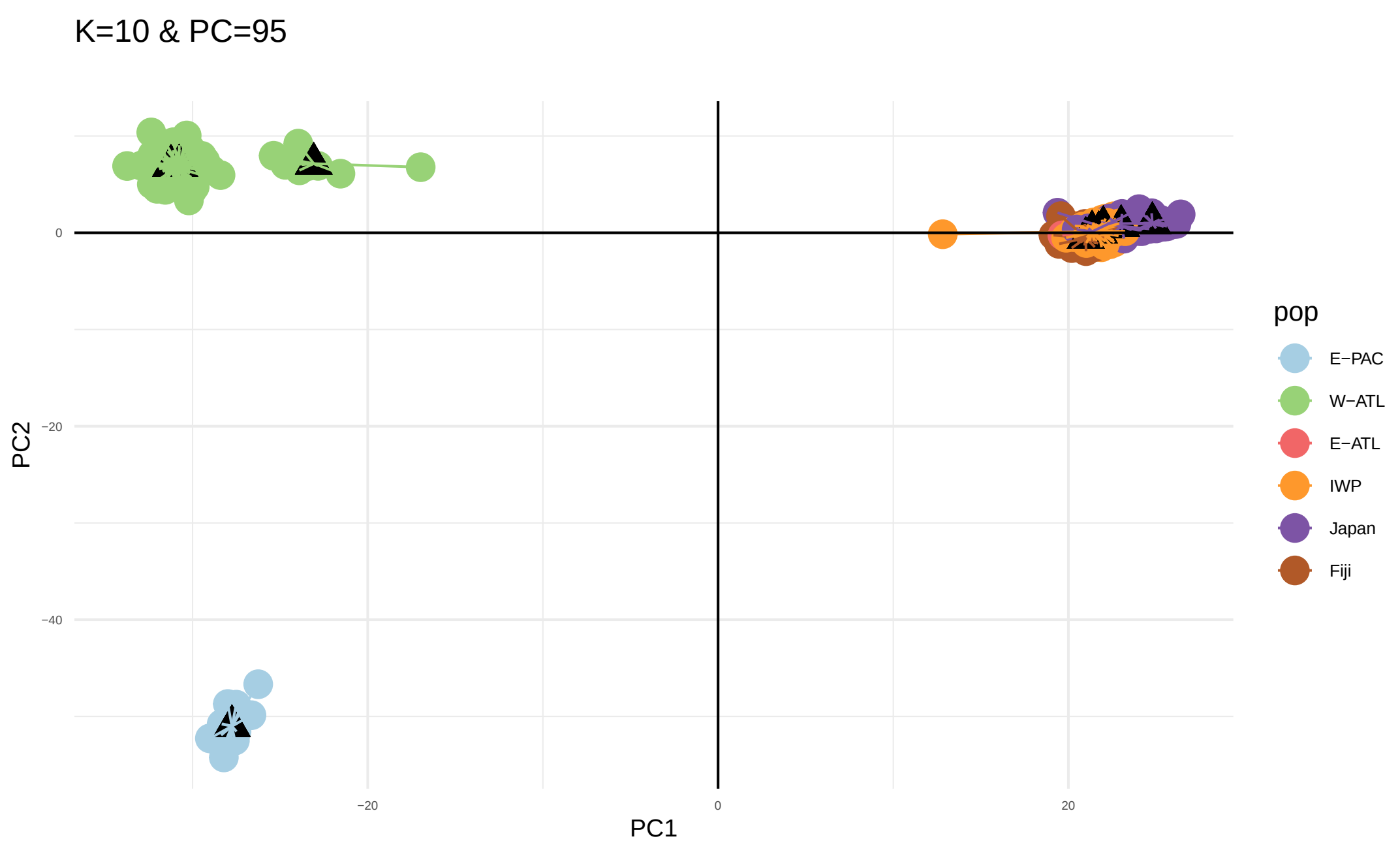

DAPC informative markers by LD contribution Using a 50% training dataset

```
load("Bull_shark_HK_DArTcap_SPLIT_objects.Rdata")

# BS.dartcap.HK.gl.assign <- BS.dartcap.HK.gl[!BS.dartcap.HK.gl$pop ==
#                                               "Hong Kong fin trade",]

BS.dartcap.gl1 <- BS.dartcap.HK.gl.ref[!BS.dartcap.HK.gl.ref@other$ind.metrics$Site4 %in%
                                            "Okinawa, Japan"]
pop.levels2 <- c("E-PAC","W-ATL", "E-ATL", "IWP", "Japan", "Fiji")
BS.dartcap.gl1$pop <- factor(BS.dartcap.gl1@other$ind.metrics$Region2, levels = pop.levels2)

subset.names <- c()
for (i in levels(BS.dartcap.gl1$pop)) {
```

```r
  gl <- BS.dartcap.gl1[BS.dartcap.gl1$pop == i,]
  subset.names <- c(subset.names,sample(gl$ind.names,
                                        size = ceiling(adegenet::nInd(gl)/2)))
}
BS.dartcap.gl2 <- BS.dartcap.gl1[BS.dartcap.gl1$ind.names %in% subset.names,]
BS.dartcap.gl3 <- BS.dartcap.gl1[!BS.dartcap.gl1$ind.names %in% subset.names[
  subset.names != "CL-SIL001-F"],] #only 1 sample


dapc1 <- adegenet::dapc(BS.dartcap.gl2, n.da = 5, n.pca = 76)
assign.success <- summary(dapc1)$assign.per.pop*100

plot.data <- data.frame(dapc1$ind.coord, pop = BS.dartcap.gl2$pop)
ggplot(plot.data, aes(x = LD1, y = LD2, col = pop)) + ggplot2::geom_point() +
  ggplot2::scale_color_manual(values = adegenet::funky(6))
ggplot(plot.data, aes(x = LD3, y = LD5, col = pop)) + ggplot2::geom_point() +
  ggplot2::scale_color_manual(values = adegenet::funky(6))
ggplot(plot.data, aes(x = LD3, y = LD4, col = pop)) + ggplot2::geom_point() +
  ggplot2::scale_color_manual(values = adegenet::funky(6))
ggplot(plot.data, aes(x = LD4, y = LD5, col = pop)) + ggplot2::geom_point() +
  ggplot2::scale_color_manual(values = adegenet::funky(6))

marker.sample <- c(seq(5,100, by = 5), 125, 250, 375, 500)
loc.number <- list()
loc.names <- list()
assign.success <- data.frame(Pop =  levels(BS.dartcap.gl2$pop))
for (i in 1:length(marker.sample)) {
  loc.number[[i]] <- c(
    rownames(dapc1$var.contr %>%
        as.data.frame() %>%
        dplyr::slice_max(n = marker.sample[i] / 5, with_ties  = FALSE, order_by = LD1)
    ),
    rownames(dapc1$var.contr %>%
        as.data.frame() %>%
        dplyr::slice_max(n = marker.sample[i] / 5, with_ties  = FALSE, order_by = LD2)
    ),
    rownames(dapc1$var.contr %>%
        as.data.frame() %>%
        dplyr::slice_max(n = marker.sample[i] / 5, with_ties  = FALSE, order_by = LD3)
    ),
    rownames(dapc1$var.contr %>%
        as.data.frame() %>%
        dplyr::slice_max(n = marker.sample[i] / 5, with_ties  = FALSE, order_by = LD4)
    ),
    rownames(dapc1$var.contr %>%
        as.data.frame() %>%
        dplyr::slice_max(n = marker.sample[i] / 5, with_ties  = FALSE, order_by = LD5)
    ))
  loc.names[[i]] <- BS.dartcap.gl2$loc.names[as.integer(loc.number[[i]])]
  BS.dartcap.gl4 <- BS.dartcap.gl3[, as.integer(loc.number[[i]])]
  dapc2 <- adegenet::dapc(BS.dartcap.gl4,n.da = 5, n.pca = 76)
  assign.success[[i + 1]] <- as.double(summary(dapc2)$assign.per.pop*100)
}
colnames(assign.success) <- c("pop", as.character(marker.sample))
save(assign.success,marker.sample, loc.number,loc.names, subset.names, assign.success,
     file = "DArTcap_ALL_Informative_DAPC_markers_NEW.Rdata")
```

```r
load("DArTcap_ALL_Informative_DAPC_markers_NEW.Rdata")

mean.assign.success <- colMeans(assign.success[,-1])

region.levels <- c("E-PAC", "W-ATL", "E-ATL", "IWP","Japan", "Fiji")

plot.data <- tidyr::gather(assign.success, "Markers", "Assignment", 2:ncol(assign.success))
plot.data$Markers <- as.integer(plot.data$Markers)
plot.data$pop <- factor(plot.data$pop, levels = region.levels)
plot.data <- plot.data[order(as.integer(plot.data$Markers), plot.data$pop),]
assign.plot <- ggplot2::ggplot(plot.data,
                               aes(x = Markers,
                                   y = Assignment,
                                   col = pop,
                                   group = 1)) +
  ylab("Assignmenet success (%)") +
  xlab("Number of informative markers") +
  ggplot2::scale_color_manual(values = funky(6)) +
  ggplot2::geom_point() +
  ggformula::geom_spline(aes(group = pop))

print(assign.plot)
ggplot2::ggsave(assign.plot,
                filename = "DArTcap_ALL_Assign_DAPC_NEW.png",
                width = 30, height = 15, units = "cm")
```

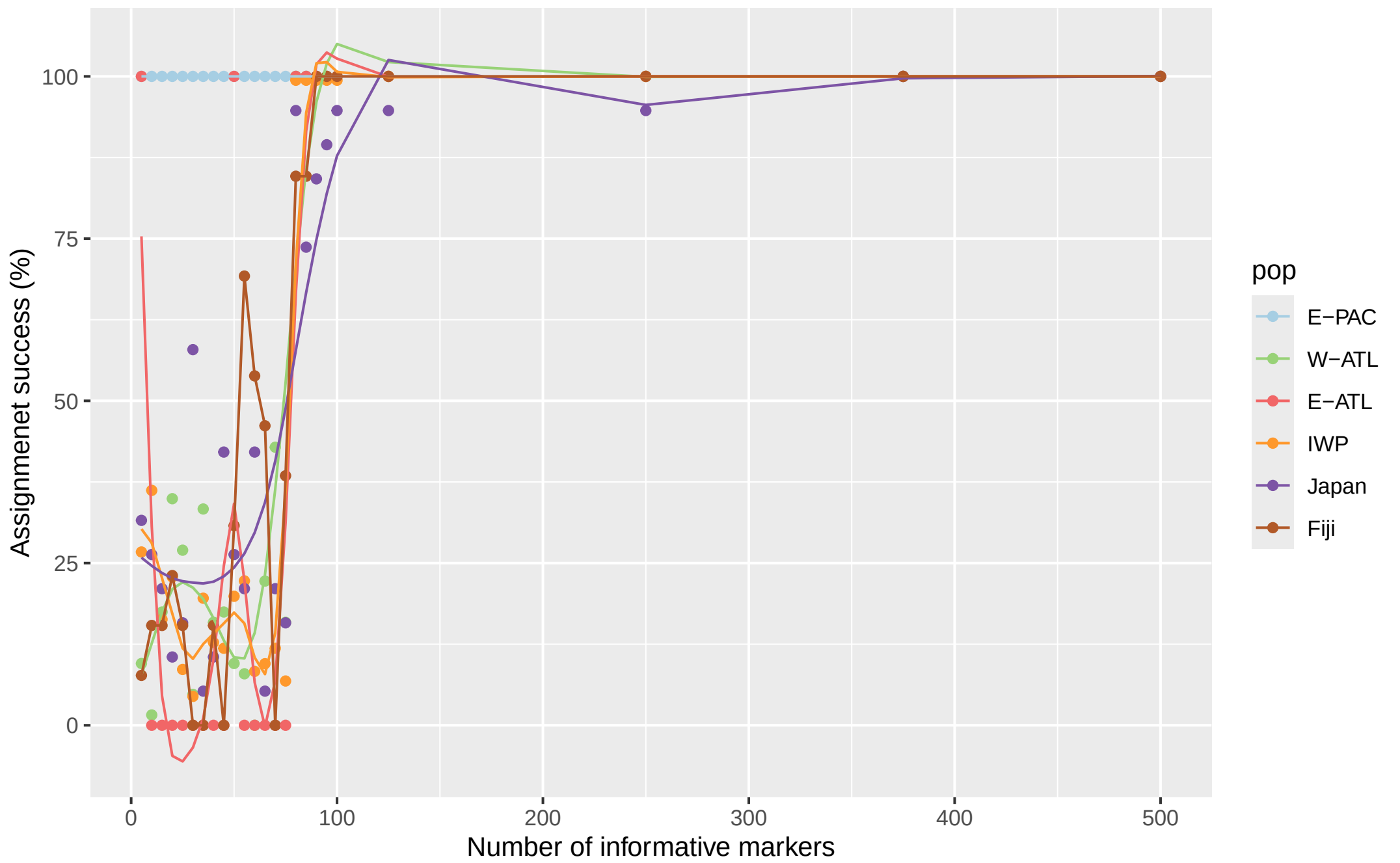

```r
load("Bull_shark_HK_DArTcap_SPLIT_objects.Rdata")
load("DArTcap_HK_DAPC4.Rdata")

for (K in 2:10) {
  colour <- colours.prior[1:K]

  dapc <- get(dapc.all.object.names[K-1])
  dat <- data.frame(BS.dartcap.HK.gl.ref$pop, names(dapc$grp), dapc$grp)
  df <- table(dat$BS.dartcap.HK.gl.ref.pop, dat$dapc.grp)
  print(knitr::kable(df))

  HK.assigned <- adegenet::predict.dapc(dapc, BS.dartcap.HK.gl.assign)
  print(knitr::kable(t(table(HK.assigned$assign))))

  barplot(t(as.data.frame(HK.assigned$posterior)), col = colour, las = 2,
          cex.names = 0.5)
  dev.print(
    device = png,
    file = paste0("2.DArTcap_HK_DAPC_assign_posterior_barplot4_K",
                  K,".png"),
    width = 30,
    height = 15,
    units = "cm",
    res = 300
  )

  assigned_pop <- apply(df, 2, function(col) rownames(df)[which.max(col)])
  HK.dat <- data.frame(individuals = rownames(HK.assigned$posterior),
                       group = HK.assigned$assign,
                       assigned_pop = HK.assigned$assign)
  HK.dat$assigned_pop <- assigned_pop[as.character(HK.dat$assigned_pop)]

  write_tsv(HK.dat, file = paste0("BS_HK_Group_assignment2_K", K, ".tsv"))
}
```

DAPC provenance of HKG samples

	1	2
E-PAC	0	18
W-ATL	0	126
E-ATL	1	0
IWP	120	0
Japan	49	0
Fiji	26	0

1	2
105	35

	1	2	3
E-PAC	0	18	0
W-ATL	0	0	126

	1	2	3
E-ATL	1	0	0
IWP	120	0	0
Japan	49	0	0
Fiji	26	0	0

1	2	3
105	4	31

	1	2	3	4
E-PAC	0	0	0	18
W-ATL	0	0	126	0
E-ATL	0	1	0	0
IWP	0	120	0	0
Japan	39	10	0	0
Fiji	0	26	0	0

1	2	3	4
0	105	31	4

	1	2	3	4	5
E-PAC	0	0	0	18	0
W-ATL	0	0	126	0	0
E-ATL	0	0	0	0	1
IWP	0	0	0	0	120
Japan	5	25	0	0	19
Fiji	26	0	0	0	0

1	2	3	4	5
0	0	31	4	105

	1	2	3	4	5	6
E-PAC	0	18	0	0	0	0
W-ATL	0	0	0	0	126	0
E-ATL	0	0	0	1	0	0
IWP	0	0	0	120	0	0
Japan	14	0	0	10	0	25
Fiji	0	0	26	0	0	0

1	2	3	4	5	6
0	4	0	105	31	0

	1	2	3	4	5	6	7
E-PAC	0	0	0	0	18	0	0
W-ATL	0	0	116	10	0	0	0
E-ATL	0	0	0	0	0	1	0
IWP	0	0	0	0	0	120	0
Japan	14	25	0	0	0	5	5
Fiji	0	0	0	0	0	0	26

1	2	3	4	5	6	7
0	0	30	1	4	105	0

	1	2	3	4	5	6	7	8
E-PAC	18	0	0	0	0	0	0	0
W-ATL	0	10	0	116	0	0	0	0
E-ATL	0	0	0	0	0	0	1	0
IWP	0	0	0	0	0	0	43	77
Japan	0	0	25	0	0	14	7	3
Fiji	0	0	0	0	26	0	0	0

1	2	3	4	5	6	7	8
4	1	0	30	0	0	27	78

	1	2	3	4	5	6	7	8	9
E-PAC	0	0	0	18	0	0	0	0	0
W-ATL	0	0	0	0	116	10	0	0	0
E-ATL	1	0	0	0	0	0	0	0	0
IWP	44	76	0	0	0	0	0	0	0
Japan	4	1	11	0	0	0	19	0	14
Fiji	0	0	0	0	0	0	0	26	0

1	2	3	4	5	6	7	8	9
34	71	0	4	30	1	0	0	0

	1	2	3	4	5	6	7	8	9	10
E-PAC	18	0	0	0	0	0	0	0	0	0
W-ATL	0	0	55	10	0	61	0	0	0	0
E-ATL	0	0	0	0	0	0	0	0	1	0
IWP	0	61	0	0	0	0	0	0	39	20
Japan	0	1	0	0	25	0	0	14	3	6
Fiji	0	0	0	0	0	0	26	0	0	0

1	2	3	4	5	6	7	8	9	10
4	57	13	1	0	17	0	0	48	0

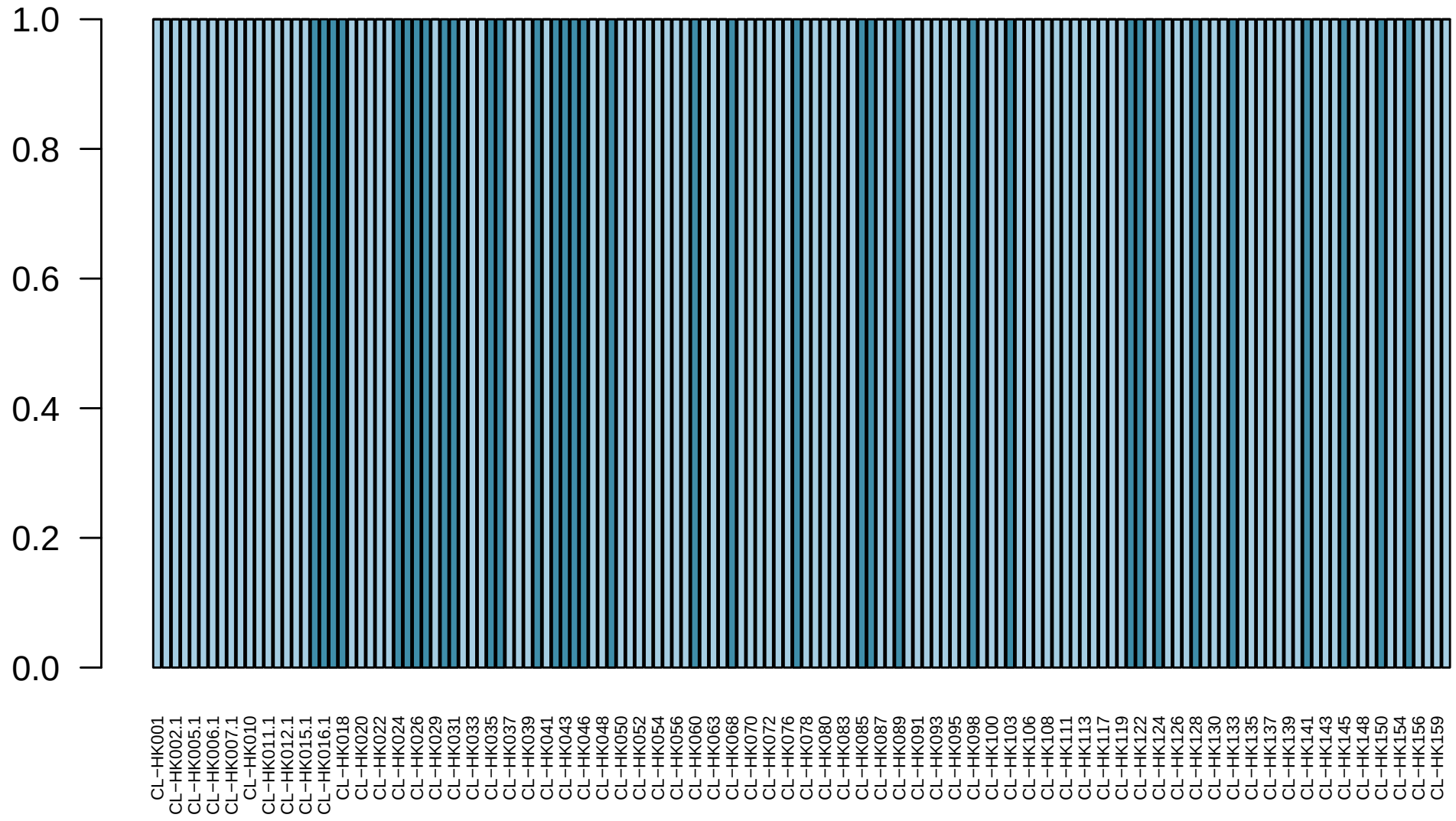
1.0
0.8
0.6
0.4
0.2
0.0
CL−HK001
CL−HK002.1
CL−HK005.1
CL−HK006.1
CL−HK007.1
CL−HK010
CL−HK011.1
CL−HK012.1
CL−HK015.1
CL−HK016.1
CL−HK018
CL−HK020
CL−HK022
CL−HK024
CL−HK026
CL−HK029
CL−HK031
CL−HK033
CL−HK035
CL−HK037
CL−HK039
CL−HK041
CL−HK043
CL−HK046
CL−HK048
CL−HK050
CL−HK052
CL−HK054
CL−HK056
CL−HK060
CL−HK063
CL−HK068
CL−HK070
CL−HK072
CL−HK076
CL−HK078
CL−HK080
CL−HK083
CL−HK085
CL−HK087
CL−HK089
CL−HK091
CL−HK093
CL−HK095
CL−HK098
CL−HK100
CL−HK103
CL−HK106
CL−HK108
CL−HK111
CL−HK113
CL−HK117
CL−HK119
CL−HK122
CL−HK124
CL−HK126
CL−HK128
CL−HK130
CL−HK133
CL−HK135
CL−HK137
CL−HK139
CL−HK141
CL−HK143
CL−HK145
CL−HK148
CL−HK150
CL−HK154
CL−HK156
CL−HK159

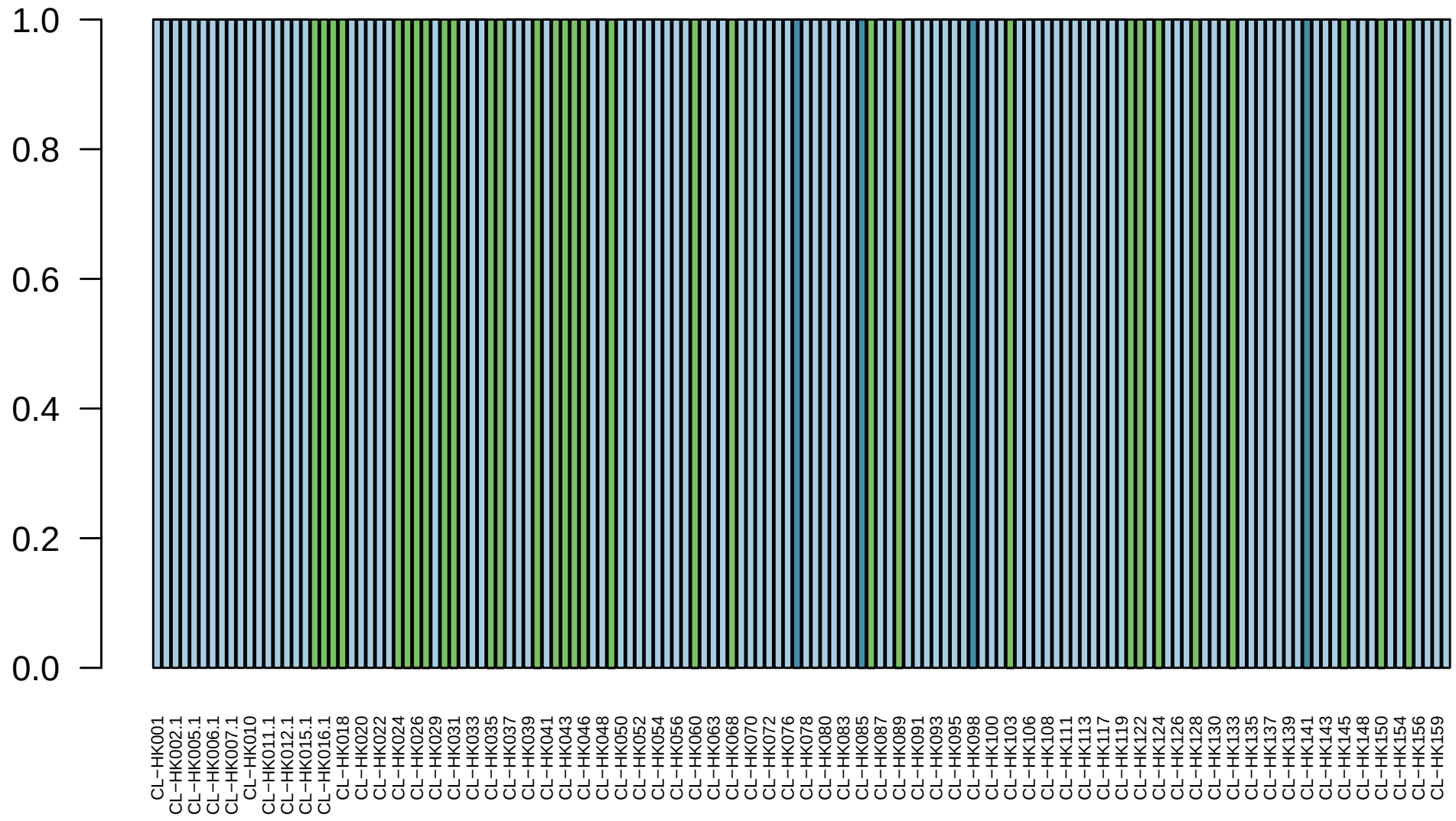
1.0
0.8
0.6
0.4
0.2
0.0
CL−HK001
CL−HK002.1
CL−HK005.1
CL−HK006.1
CL−HK007.1
CL−HK010
CL−HK011.1
CL−HK012.1
CL−HK015.1
CL−HK016.1
CL−HK018
CL−HK020
CL−HK022
CL−HK024
CL−HK026
CL−HK029
CL−HK031
CL−HK033
CL−HK035
CL−HK037
CL−HK039
CL−HK041
CL−HK043
CL−HK046
CL−HK048
CL−HK050
CL−HK052
CL−HK054
CL−HK056
CL−HK060
CL−HK063
CL−HK068
CL−HK070
CL−HK072
CL−HK076
CL−HK078
CL−HK080
CL−HK083
CL−HK085
CL−HK087
CL−HK089
CL−HK091
CL−HK093
CL−HK095
CL−HK098
CL−HK100
CL−HK103
CL−HK106
CL−HK108
CL−HK111
CL−HK113
CL−HK117
CL−HK119
CL−HK122
CL−HK124
CL−HK126
CL−HK128
CL−HK130
CL−HK133
CL−HK135
CL−HK137
CL−HK139
CL−HK141
CL−HK143
CL−HK145
CL−HK148
CL−HK150
CL−HK154
CL−HK156
CL−HK159

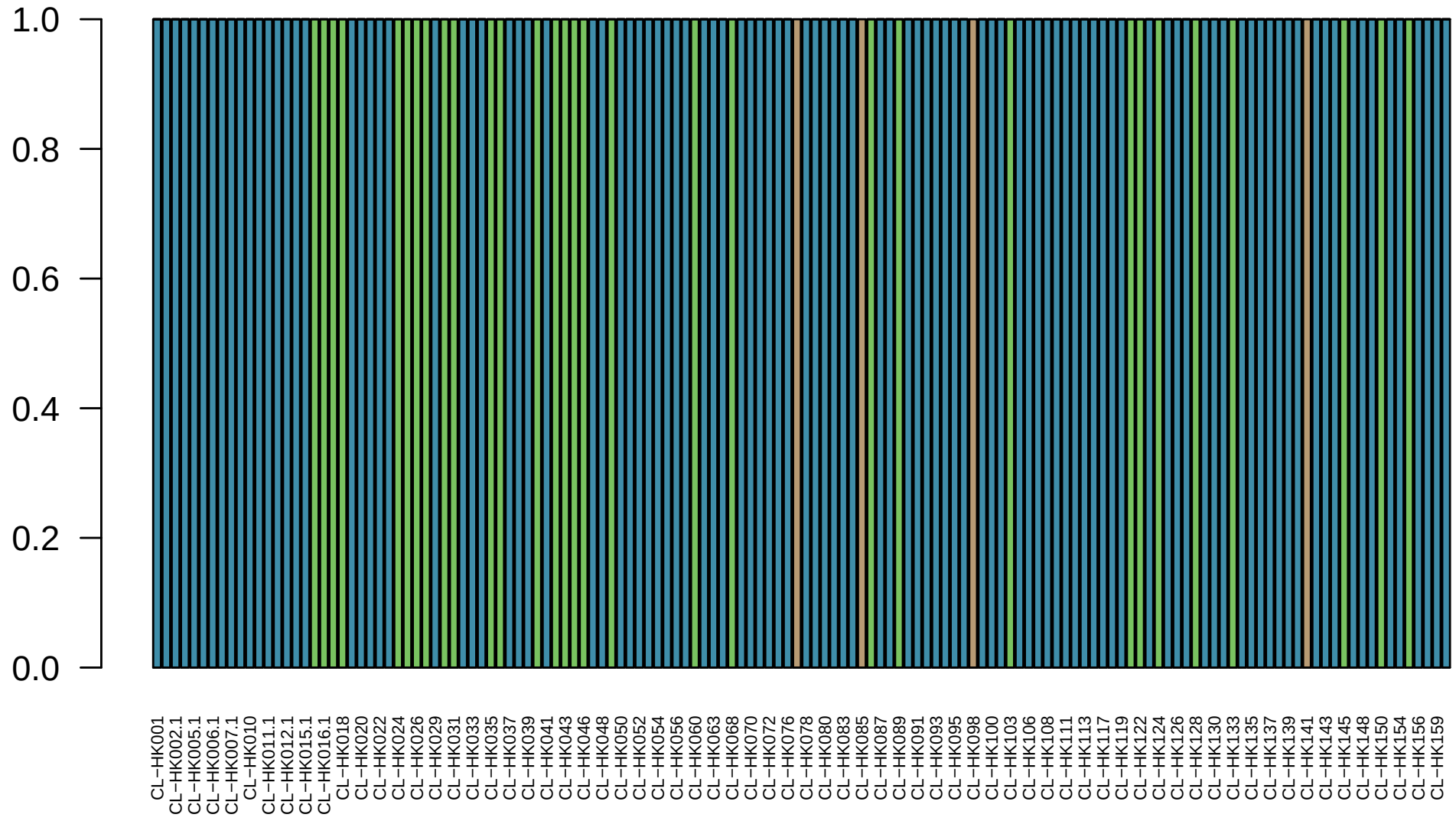
1.0
0.8
0.6
0.4
0.2
0.0
CL−HK001
CL−HK002.1
CL−HK005.1
CL−HK006.1
CL−HK007.1
CL−HK010
CL−HK011.1
CL−HK012.1
CL−HK015.1
CL−HK016.1
CL−HK018
CL−HK020
CL−HK022
CL−HK024
CL−HK026
CL−HK029
CL−HK031
CL−HK033
CL−HK035
CL−HK037
CL−HK039
CL−HK041
CL−HK043
CL−HK046
CL−HK048
CL−HK050
CL−HK052
CL−HK054
CL−HK056
CL−HK060
CL−HK063
CL−HK068
CL−HK070
CL−HK072
CL−HK076
CL−HK078
CL−HK080
CL−HK083
CL−HK085
CL−HK087
CL−HK089
CL−HK091
CL−HK093
CL−HK095
CL−HK098
CL−HK100
CL−HK103
CL−HK106
CL−HK108
CL−HK111
CL−HK113
CL−HK117
CL−HK119
CL−HK122
CL−HK124
CL−HK126
CL−HK128
CL−HK130
CL−HK133
CL−HK135
CL−HK137
CL−HK139
CL−HK141
CL−HK143
CL−HK145
CL−HK148
CL−HK150
CL−HK154
CL−HK156
CL−HK159

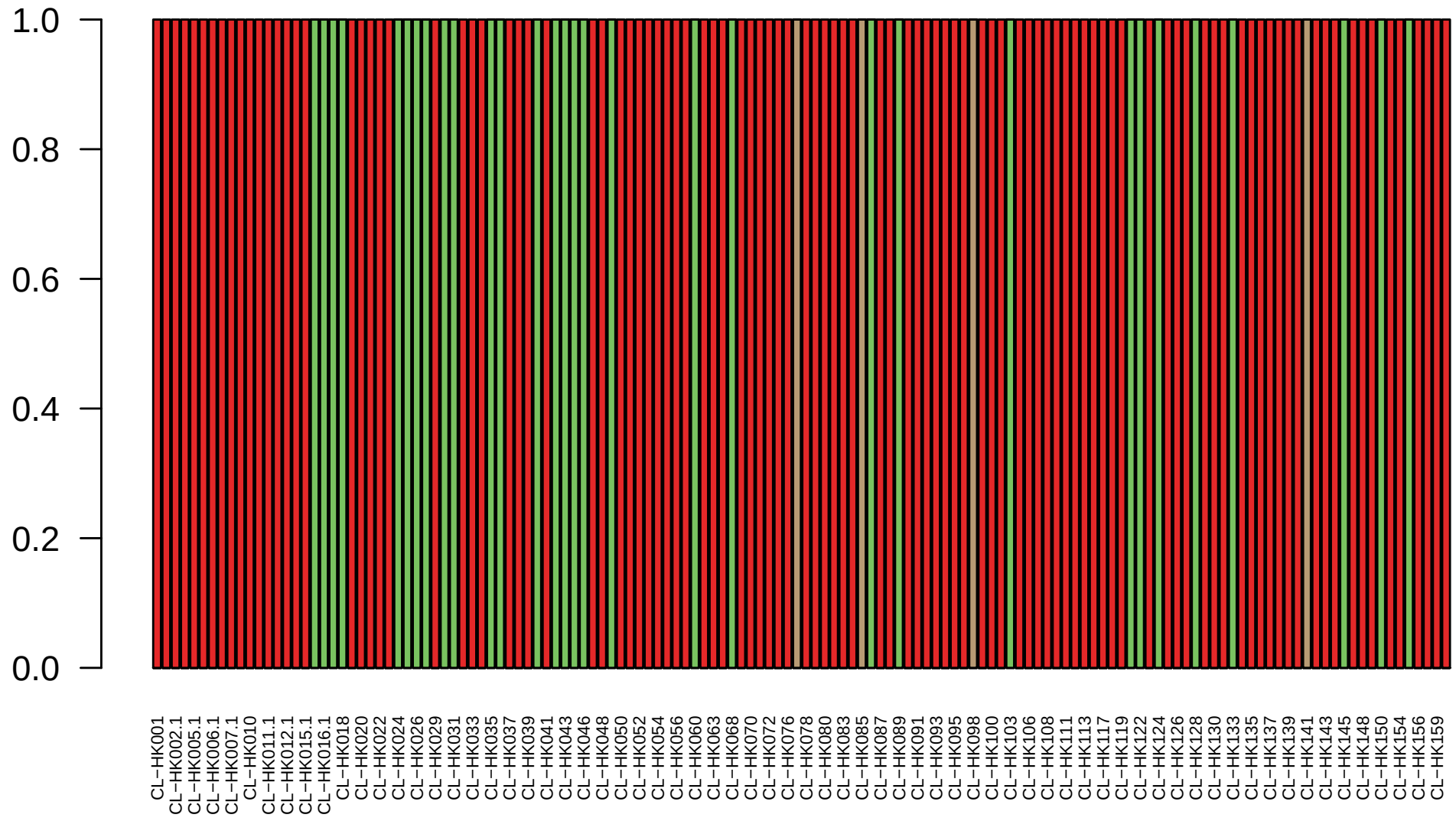
1.0
0.8
0.6
0.4
0.2
0.0
CL−HK001
CL−HK002.1
CL−HK005.1
CL−HK006.1
CL−HK007.1
CL−HK010
CL−HK011.1
CL−HK012.1
CL−HK015.1
CL−HK016.1
CL−HK018
CL−HK020
CL−HK022
CL−HK024
CL−HK026
CL−HK029
CL−HK031
CL−HK033
CL−HK035
CL−HK037
CL−HK039
CL−HK041
CL−HK043
CL−HK046
CL−HK048
CL−HK050
CL−HK052
CL−HK054
CL−HK056
CL−HK060
CL−HK063
CL−HK068
CL−HK070
CL−HK072
CL−HK076
CL−HK078
CL−HK080
CL−HK083
CL−HK085
CL−HK087
CL−HK089
CL−HK091
CL−HK093
CL−HK095
CL−HK098
CL−HK100
CL−HK103
CL−HK106
CL−HK108
CL−HK111
CL−HK113
CL−HK117
CL−HK119
CL−HK122
CL−HK124
CL−HK126
CL−HK128
CL−HK130
CL−HK133
CL−HK135
CL−HK137
CL−HK139
CL−HK141
CL−HK143
CL−HK145
CL−HK148
CL−HK150
CL−HK154
CL−HK156
CL−HK159

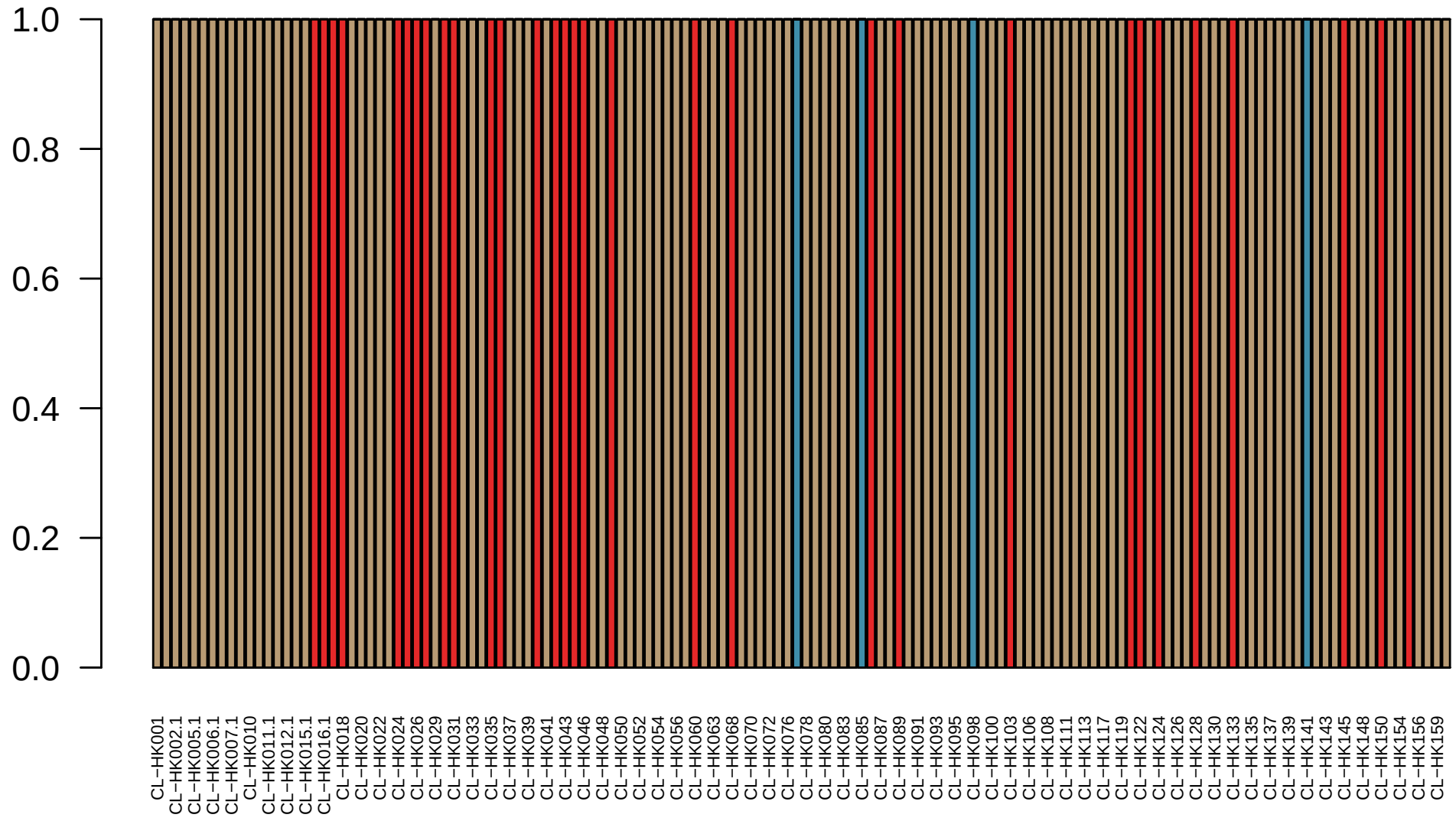
1.0
0.8
0.6
0.4
0.2
0.0
CL−HK001
CL−HK002.1
CL−HK005.1
CL−HK006.1
CL−HK007.1
CL−HK010
CL−HK011.1
CL−HK012.1
CL−HK015.1
CL−HK016.1
CL−HK018
CL−HK020
CL−HK022
CL−HK024
CL−HK026
CL−HK029
CL−HK031
CL−HK033
CL−HK035
CL−HK037
CL−HK039
CL−HK041
CL−HK043
CL−HK046
CL−HK048
CL−HK050
CL−HK052
CL−HK054
CL−HK056
CL−HK060
CL−HK063
CL−HK068
CL−HK070
CL−HK072
CL−HK076
CL−HK078
CL−HK080
CL−HK083
CL−HK085
CL−HK087
CL−HK089
CL−HK091
CL−HK093
CL−HK095
CL−HK098
CL−HK100
CL−HK103
CL−HK106
CL−HK108
CL−HK111
CL−HK113
CL−HK117
CL−HK119
CL−HK122
CL−HK124
CL−HK126
CL−HK128
CL−HK130
CL−HK133
CL−HK135
CL−HK137
CL−HK139
CL−HK141
CL−HK143
CL−HK145
CL−HK148
CL−HK150
CL−HK154
CL−HK156
CL−HK159

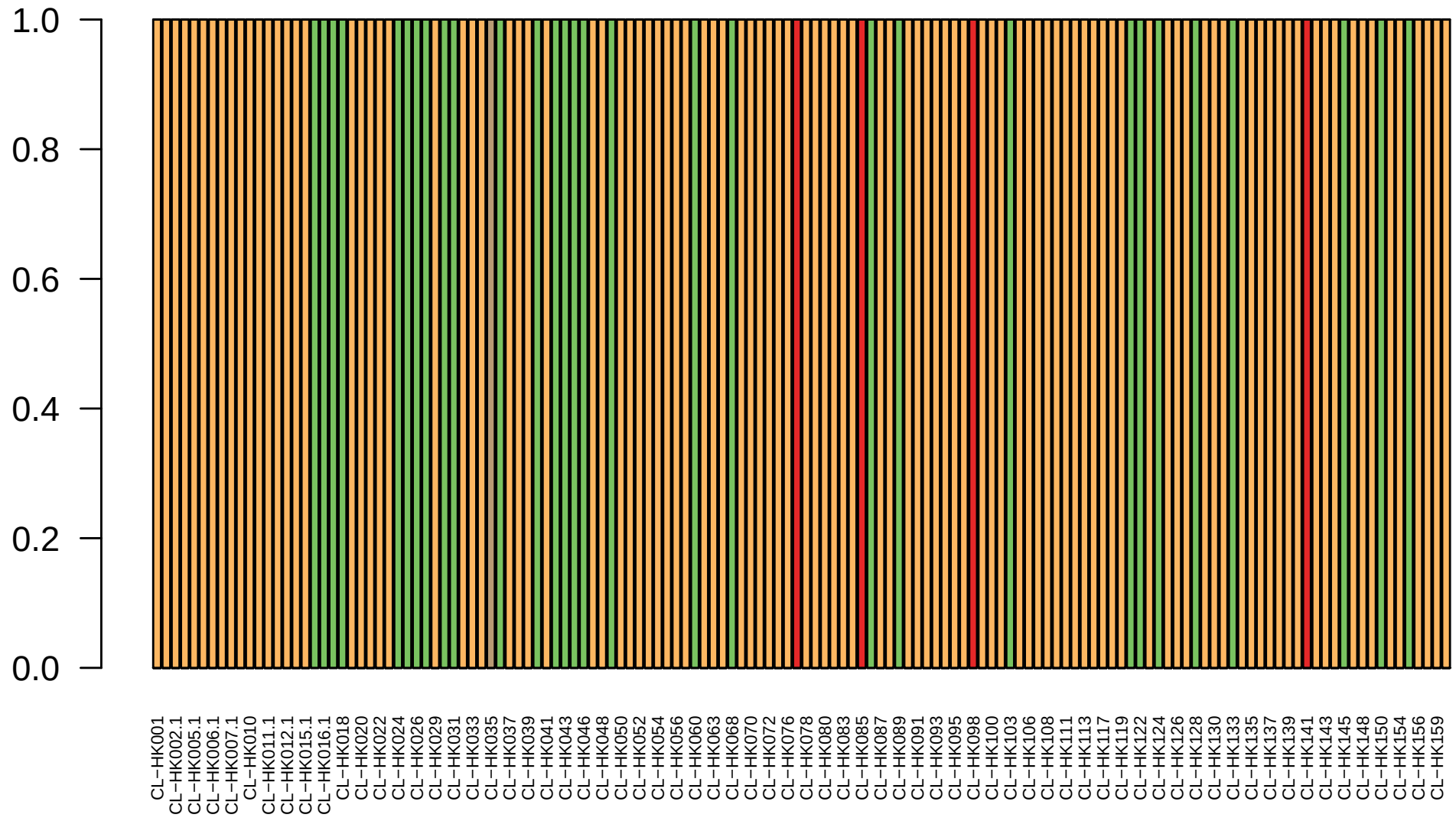
1.0
0.8
0.6
0.4
0.2
0.0
CL−HK001
CL−HK002.1
CL−HK005.1
CL−HK006.1
CL−HK007.1
CL−HK010
CL−HK011.1
CL−HK012.1
CL−HK015.1
CL−HK016.1
CL−HK018
CL−HK020
CL−HK022
CL−HK024
CL−HK026
CL−HK029
CL−HK031
CL−HK033
CL−HK035
CL−HK037
CL−HK039
CL−HK041
CL−HK043
CL−HK046
CL−HK048
CL−HK050
CL−HK052
CL−HK054
CL−HK056
CL−HK060
CL−HK063
CL−HK068
CL−HK070
CL−HK072
CL−HK076
CL−HK078
CL−HK080
CL−HK083
CL−HK085
CL−HK087
CL−HK089
CL−HK091
CL−HK093
CL−HK095
CL−HK098
CL−HK100
CL−HK103
CL−HK106
CL−HK108
CL−HK111
CL−HK113
CL−HK117
CL−HK119
CL−HK122
CL−HK124
CL−HK126
CL−HK128
CL−HK130
CL−HK133
CL−HK135
CL−HK137
CL−HK139
CL−HK141
CL−HK143
CL−HK145
CL−HK148
CL−HK150
CL−HK154
CL−HK156
CL−HK159

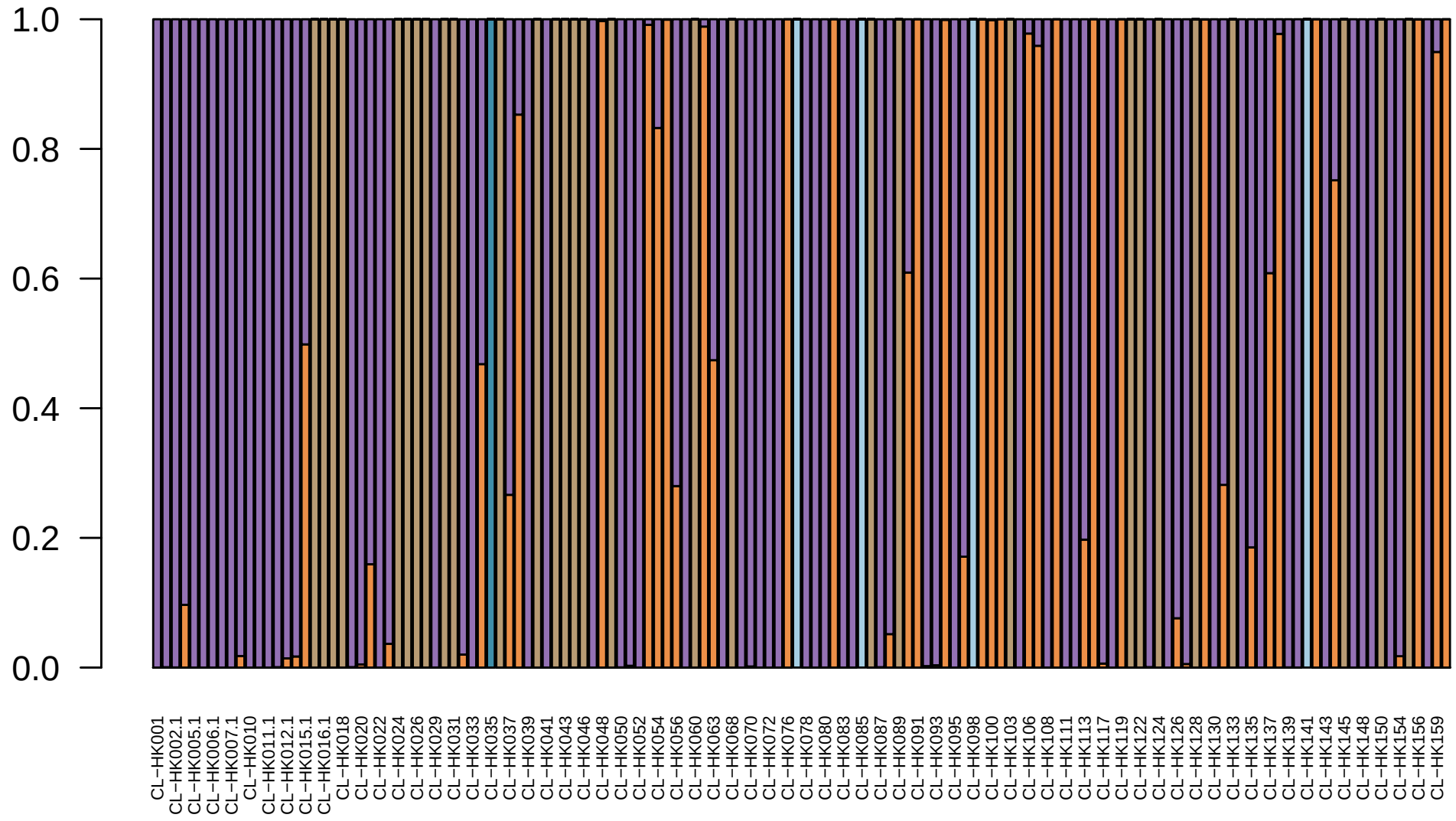
1.0
0.8
0.6
0.4
0.2
0.0
CL−HK001
CL−HK002.1
CL−HK005.1
CL−HK006.1
CL−HK007.1
CL−HK010
CL−HK011.1
CL−HK012.1
CL−HK015.1
CL−HK016.1
CL−HK018
CL−HK020
CL−HK022
CL−HK024
CL−HK026
CL−HK029
CL−HK031
CL−HK033
CL−HK035
CL−HK037
CL−HK039
CL−HK041
CL−HK043
CL−HK046
CL−HK048
CL−HK050
CL−HK052
CL−HK054
CL−HK056
CL−HK060
CL−HK063
CL−HK068
CL−HK070
CL−HK072
CL−HK076
CL−HK078
CL−HK080
CL−HK083
CL−HK085
CL−HK087
CL−HK089
CL−HK091
CL−HK093
CL−HK095
CL−HK098
CL−HK100
CL−HK103
CL−HK106
CL−HK108
CL−HK111
CL−HK113
CL−HK117
CL−HK119
CL−HK122
CL−HK124
CL−HK126
CL−HK128
CL−HK130
CL−HK133
CL−HK135
CL−HK137
CL−HK139
CL−HK141
CL−HK143
CL−HK145
CL−HK148
CL−HK150
CL−HK154
CL−HK156
CL−HK159

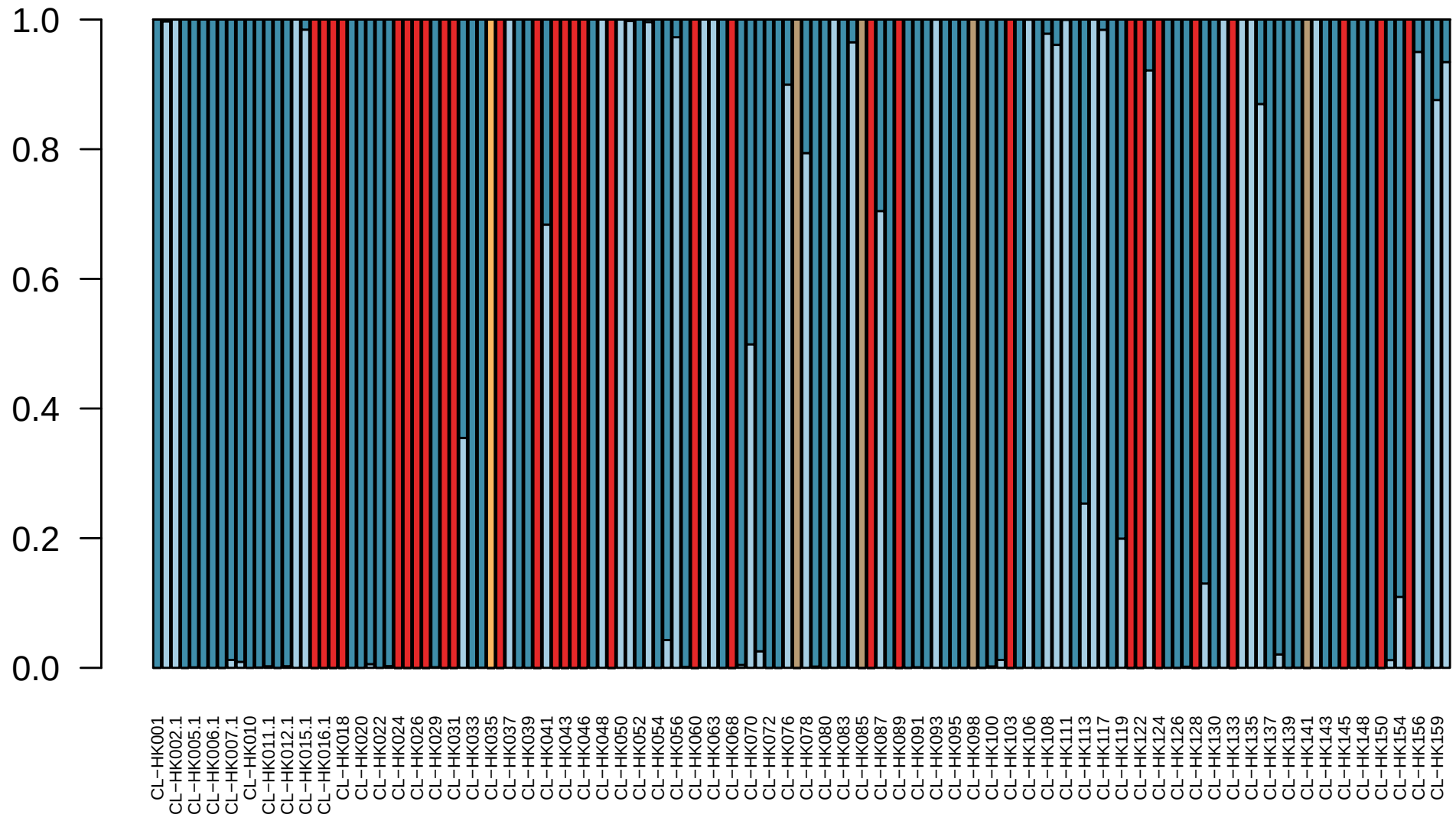
1.0
0.8
0.6
0.4
0.2
0.0
CL−HK001
CL−HK002.1
CL−HK005.1
CL−HK006.1
CL−HK007.1
CL−HK010
CL−HK011.1
CL−HK012.1
CL−HK015.1
CL−HK016.1
CL−HK018
CL−HK020
CL−HK022
CL−HK024
CL−HK026
CL−HK029
CL−HK031
CL−HK033
CL−HK035
CL−HK037
CL−HK039
CL−HK041
CL−HK043
CL−HK046
CL−HK048
CL−HK050
CL−HK052
CL−HK054
CL−HK056
CL−HK060
CL−HK063
CL−HK068
CL−HK070
CL−HK072
CL−HK076
CL−HK078
CL−HK080
CL−HK083
CL−HK085
CL−HK087
CL−HK089
CL−HK091
CL−HK093
CL−HK095
CL−HK098
CL−HK100
CL−HK103
CL−HK106
CL−HK108
CL−HK111
CL−HK113
CL−HK117
CL−HK119
CL−HK122
CL−HK124
CL−HK126
CL−HK128
CL−HK130
CL−HK133
CL−HK135
CL−HK137
CL−HK139
CL−HK141
CL−HK143
CL−HK145
CL−HK148
CL−HK150
CL−HK154
CL−HK156
CL−HK159

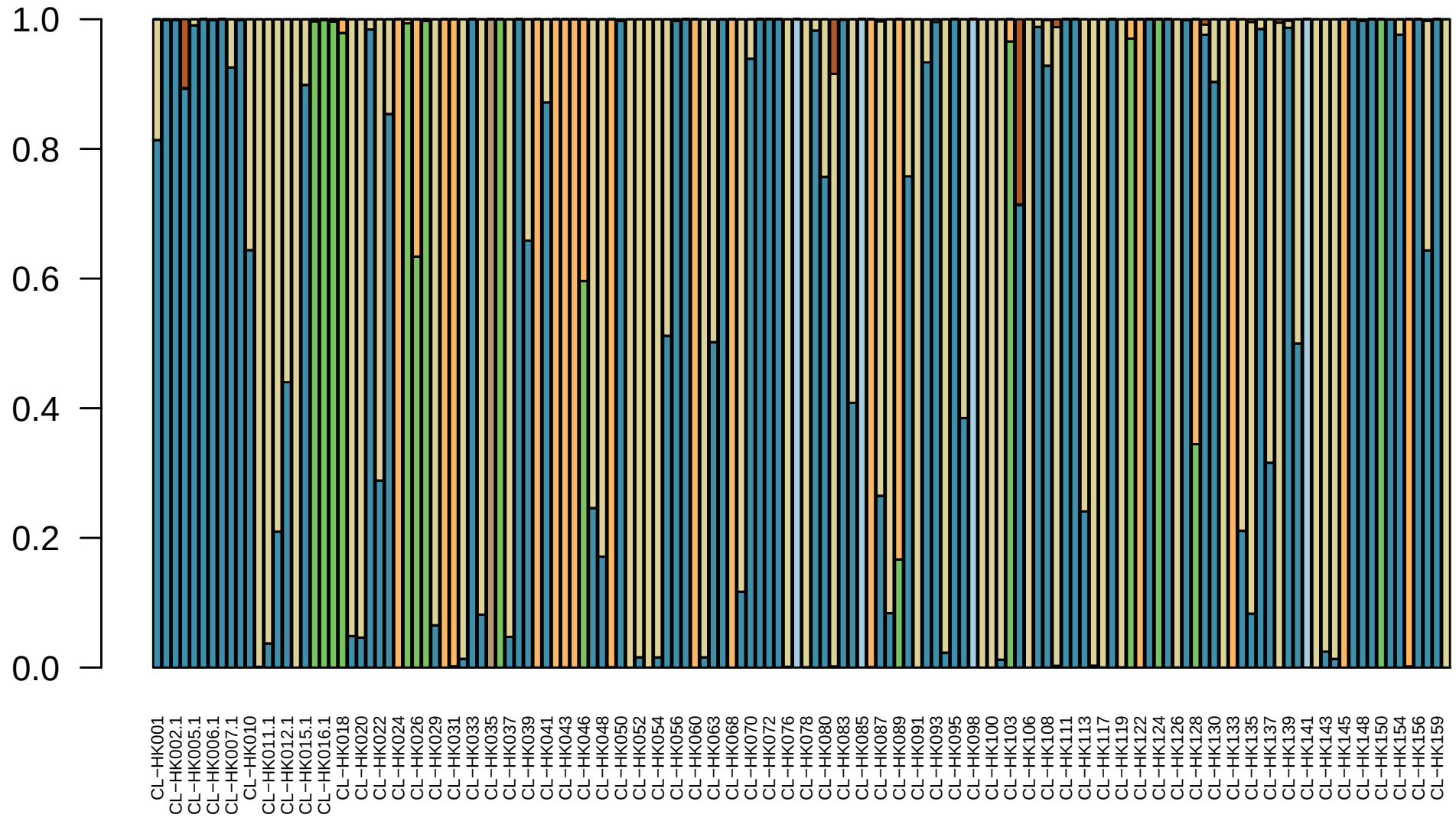
1.0
0.8
0.6
0.4
0.2
0.0
CL−HK001
CL−HK002.1
CL−HK005.1
CL−HK006.1
CL−HK007.1
CL−HK010
CL−HK011.1
CL−HK012.1
CL−HK015.1
CL−HK016.1
CL−HK018
CL−HK020
CL−HK022
CL−HK024
CL−HK026
CL−HK029
CL−HK031
CL−HK033
CL−HK035
CL−HK037
CL−HK039
CL−HK041
CL−HK043
CL−HK046
CL−HK048
CL−HK050
CL−HK052
CL−HK054
CL−HK056
CL−HK060
CL−HK063
CL−HK068
CL−HK070
CL−HK072
CL−HK076
CL−HK078
CL−HK080
CL−HK083
CL−HK085
CL−HK087
CL−HK089
CL−HK091
CL−HK093
CL−HK095
CL−HK098
CL−HK100
CL−HK103
CL−HK106
CL−HK108
CL−HK111
CL−HK113
CL−HK117
CL−HK119
CL−HK122
CL−HK124
CL−HK126
CL−HK128
CL−HK130
CL−HK133
CL−HK135
CL−HK137
CL−HK139
CL−HK141
CL−HK143
CL−HK145
CL−HK148
CL−HK150
CL−HK154
CL−HK156
CL−HK159

Rubias assignment

```
load("DArTcap_ALL_Informative_DAPC_markers.Rdata")

# BS.dartcap.HK.tidy$STRATA <- BS.dartcap.HK.tidy$POP_ID
# BS.dartcap.HK.tidy$POP_ID <- NULL
# BS.dartcap.HK.rubias2 <- write_rubias(
#   BS.dartcap.HK.tidy,
#   strata = NULL,
#   filename = NULL,
#   parallel.core =  1
# )

colnames(BS.dartcap.rubias2)
BS.dartcap.rubias2 <- BS.dartcap.HK.rubias2
BS.dartcap.rubias2 <- BS.dartcap.rubias2[match(BS.dartcap.HK.gl$ind.names,
                                               BS.dartcap.rubias2$indiv),]
# BS.dartcap.rubias2 <- BS.dartcap.rubias2[, !grepl("SPLIT_VEC",
#                                                     colnames(BS.dartcap.rubias2))]

pop.levels2 <- c("Gulf of California","Costa Rica","Brazil", "Caribbean Sea",
                 "Gulf of Mexico", "Western North Atlantic",  "Sierra Leone",
                 "South Africa","Mozambique", "Reunion","Seychelles","Arabian Sea",
                 "Sri Lanka","Thailand","Indonesia","Papua New Guinea","Australia",
                 "Okinawa, Japan", "Urauchi River, Japan","Fiji", "Hong Kong fin trade")
group <- BS.dartcap.HK.gl$other$ind.metrics$Site
group[group == "Japan"] <- BS.dartcap.HK.gl$other$ind.metrics$Site2[group == "Japan"]
group[group == "Okinawa"] <- "Okinawa, Japan"
group[group == "Urauchi River"] <- "Urauchi River, Japan"
BS.dartcap.rubias2$collection <- factor(group, levels = pop.levels2)

region.levels <- c("E-PAC","W-ATL","E-ATL", "IWP", "Japan","Fiji",
                   "Hong Kong fin trade")
region <- BS.dartcap.HK.gl$other$ind.metrics$Region
region[region %in% c("W-IO","N-IO", "E-IO", "E-IO/W-PAC", "W-PAC")] <- "IWP"
BS.dartcap.rubias2$repunit <- factor(region, levels = region.levels)

BS.dartcap.rubias2$sample_type[is.na(BS.dartcap.rubias2$repunit)] <- "mixture"
BS.dartcap.rubias2 <- BS.dartcap.rubias2[!BS.dartcap.rubias2$collection %in%
                                           "Okinawa, Japan",]

BS.dartcap.rubias2.HK <- BS.dartcap.rubias2[BS.dartcap.rubias2$collection %in%
                                              "Hong Kong fin trade",]
BS.dartcap.rubias2 <- BS.dartcap.rubias2[!BS.dartcap.rubias2$collection %in%
                                           "Hong Kong fin trade",]


# BS.dartcap.rubias2 <- BS.dartcap.rubias2[order(BS.dartcap.rubias2$repunit,
#                                                  BS.dartcap.rubias2$collection),]

levels1 <- c("Gulf of California","Costa Rica","Brazil", "Caribbean Sea",
             "Gulf of Mexico", "Western North Atlantic",  "Sierra Leone",
             "South Africa","Mozambique", "Reunion","Seychelles","Arabian Sea",
             "Sri Lanka","Thailand","Indonesia","Papua New Guinea","Australia",
             "Urauchi River, Japan","Fiji")
levels2 <- c("E-PAC","W-ATL","E-ATL", "IWP", "Japan","Fiji")
```

```r
BS.dartcap.rubias2 %<>%
  dplyr::mutate_all(as.character)

res <- data.frame(iter = integer(), indiv = integer(),
                  simulated_collection = factor(),
                  repunit = factor(), repu_PofZ = double(), Nmarkers = double())
for (i in 1:length(marker.sample)) {
  locus.rubias <- stringr::str_match(pattern = "__\\s*(.*?)\\s*__",
                                     string =  colnames(BS.dartcap.rubias2))[,2]
  locus.rubias <- c("sample_type","repunit","collection","indiv",
                    locus.rubias[-c(1:4)])
  BS.dartcap.rubias3 <- BS.dartcap.rubias2[,locus.rubias %in% c("sample_type","repunit","collection"
  BS_sims <- rubias::assess_reference_loo(reference = BS.dartcap.rubias3,
                     gen_start_col = 5,
                     reps = 5,
                     mixsize = 200,
                     return_indiv_posteriors = TRUE)
  repu_pofzs <- BS_sims$indiv_posteriors %>%
    dplyr::filter(repunit == simulated_repunit) %>%
    dplyr::group_by(iter, indiv, simulated_collection, repunit) %>%
    dplyr::summarise(repu_PofZ = sum(PofZ)) %>%
    dplyr::ungroup() %>%
    dplyr::arrange(repunit, simulated_collection) %>%
    dplyr::mutate(simulated_collection = factor(simulated_collection,
                                          levels = unique(simulated_collection)))

  num_simmed <- BS_sims$indiv_posteriors %>%
    dplyr::group_by(iter, indiv) %>%
    dplyr::slice(1) %>%
    dplyr::ungroup() %>%
    dplyr::count(simulated_collection)

  num_simmed <- num_simmed[order(factor(num_simmed$simulated_collection,
                                        levels = levels1)),]
  repu_pofzs$repunit <- factor(repu_pofzs$repunit, levels = levels2)
  repu_pofzs$simulated_collection <- factor(repu_pofzs$simulated_collection,
                                            levels = levels1)
  repu_pofzs <- repu_pofzs[order(repu_pofzs$repunit, repu_pofzs$simulated_collection),]
  repu_pofzs$Nmarkers <- marker.sample[[i]]
  res <- rbind(res,repu_pofzs)
}
save(res, file = "DArTcap_ALL_Informative_DAPC_markers_rubias_assignment_NEW.Rdata")
```

```r
load("DArTcap_ALL_Informative_DAPC_markers_rubias_assignment_NEW.Rdata")
plot <- ggplot2::ggplot(res, ggplot2::aes(x = as.factor(Nmarkers), y = repu_PofZ)) +
  ggplot2::geom_boxplot(ggplot2::aes(colour = repunit)) +
  ggplot2::scale_color_manual(values = colours.6) +
  ggplot2::facet_wrap(~simulated_collection) +
  ggplot2::labs(subtitle = "",
         y = "Assignment success",
         x = "Number of informative markers") +
  ggplot2::theme_bw() +
  ggplot2::theme(axis.text.x = ggplot2::element_text(angle = 90, hjust = 1,
                                                     size = 7, vjust = 0.5)) +
  ggplot2::ylim(c(NA, 1.05))
print(plot)
```

```r
ggplot2::ggsave(plot,
                filename = "DArTcap_ALL_Informative_DAPC_markers_rubias assignment_NEW.png",
                width = 30, height = 15,units = "cm")
```

Test DAPC informative markers with rubias assignment of simulated genotypes

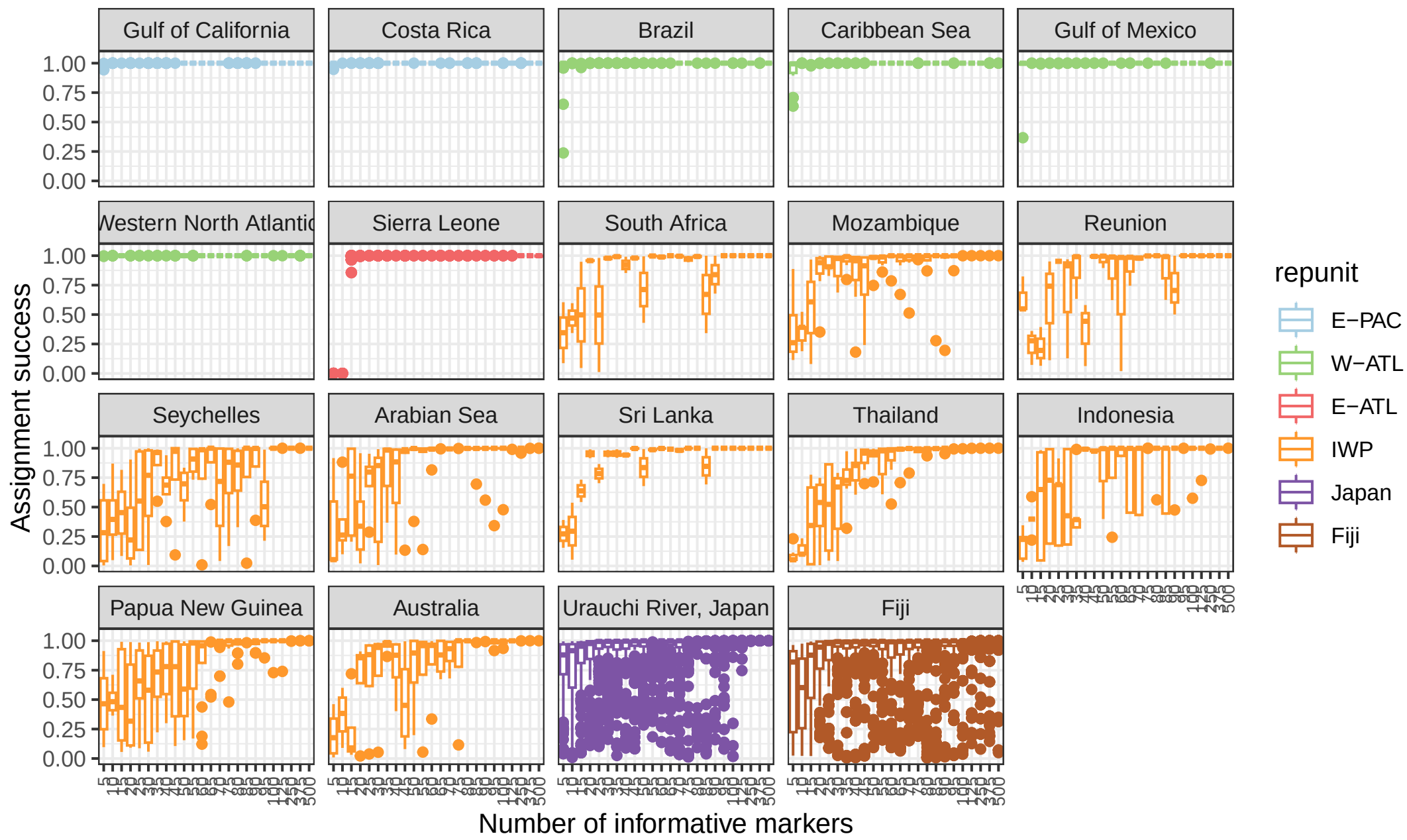


```r
pop.levels <- c("Gulf of California", "Costa Rica" , "Brazil","Caribbean Sea",
                "Gulf of Mexico",  "Western North Atlantic", "Sierra Leone",
                "South Africa", "Mozambique", "Reunion" , "Seychelles", "Arabian Sea",
                "Sri Lanka", "Thailand", "Indonesia", "Papua New Guinea" , "Australia",
                "Japan", "Fiji", "Hong Kong fin trade")
pop.levels2 <- c("E-PAC","W-ATL","E-ATL","IWP", "Japan","Fiji")

BS.dartcap.rubias2 <- BS.dartcap.HK.rubias
# BS.dartcap.rubias2$indiv == BS.dartcap.gt@schemes$INDIVIDUALS

strata <- BS.dartcap.HK.gl@other$ind.metrics$Region2
# strata[strata %in% c("W-IO","N-IO","E-IO","W-PAC","E-IO/W-PAC")] <- "IWP"
BS.dartcap.rubias2$collection <- factor(BS.dartcap.HK.gl@other$ind.metrics$Site,
                                        levels = pop.levels)
BS.dartcap.rubias2$repunit <- factor(strata, levels = pop.levels2)
BS.dartcap.rubias2 <- BS.dartcap.rubias2[order(
  BS.dartcap.rubias2$repunit,BS.dartcap.rubias2$collection),]
BS.dartcap.rubias2 <- BS.dartcap.rubias2[, !grepl("SPLIT_VEC",
                                                  colnames(BS.dartcap.rubias2))]


BS.dartcap.rubias2 %<>%
  dplyr::mutate_all(as.character)

BS_sims <- rubias::assess_reference_loo(reference = BS.dartcap.rubias2,
                     gen_start_col = 5,
```

```
                     reps = 5,
                     mixsize = 200,
                     return_indiv_posteriors = TRUE,
                     resampling_unit = "gene_copies")

repu_pofzs <- BS_sims$indiv_posteriors %>%
  dplyr::filter(repunit == simulated_repunit) %>%
  dplyr::group_by(iter, indiv, simulated_collection, repunit) %>%
  dplyr::summarise(repu_PofZ = sum(PofZ)) %>%
  dplyr::ungroup() %>%
  dplyr::arrange(repunit, simulated_collection) %>%
  dplyr::mutate(simulated_collection = factor(simulated_collection,
                                              levels = unique(simulated_collection)))

num_simmed <- BS_sims$indiv_posteriors %>%
  dplyr::group_by(iter, indiv) %>%
  dplyr::slice(1) %>%
  dplyr::ungroup() %>%
  dplyr::count(simulated_collection)

num_simmed <- num_simmed[order(factor(num_simmed$simulated_collection,
                                      levels = pop.levels)),]
repu_pofzs$repunit <- factor(repu_pofzs$repunit, levels = pop.levels2)
repu_pofzs$simulated_collection <- factor(repu_pofzs$simulated_collection,
                                          levels = pop.levels)
repu_pofzs <- repu_pofzs[order(repu_pofzs$repunit, repu_pofzs$simulated_collection),]

plot <- ggplot2::ggplot(repu_pofzs, ggplot2::aes(x = simulated_collection,
                                                 y = repu_PofZ)) +
  ggplot2::geom_boxplot(ggplot2::aes(colour = repunit)) +
  ggplot2::scale_color_manual(values = colours.6) +
  ggplot2::geom_text(data = num_simmed, mapping = aes(y = 1.025, label = n),
                     angle = 90, hjust = 0, vjust = 0.5, size = 3) +
  ggplot2::labs(subtitle = "",
         y = "Assignment success",
         x = "Collection site") +
  ggplot2::theme(axis.text.x = ggplot2::element_text(angle = 90, hjust = 1,
                                                     size = 9, vjust = 0.5)) +
  ggplot2::ylim(c(NA, 1.05))
print(plot)
ggplot2::ggsave(plot,
                filename = "DArTcap_HK_Assign_Rubias_Regions_Assignment_success.png",
                width = 30, height = 15,units = "cm")

repunit_BS <- unique(BS.dartcap.rubias2$repunit)
BS_corr <- BS_sims$mixing_proportions %>%
  dplyr::mutate(repunit = ifelse(repunit %in% repunit_BS, repunit, "OTHER")) %>%
  dplyr::group_by(iter, repunit) %>%
  dplyr::summarise(true_repprop = sum(true_pi),
            reprop_posterior_mean = sum(post_mean_pi),
            repu_n = sum(n)) %>%
  dplyr::mutate(repu_n_prop = repu_n / sum(repu_n))

BS_corr$repunit <- factor(BS_corr$repunit, levels = pop.levels2)
plot <- ggplot2::ggplot(BS_corr, ggplot2::aes(x = repu_n_prop,
                                              y = reprop_posterior_mean,
                                              colour = repunit)) +
  ggplot2::geom_point(size = 5) +
```

```
  ggplot2::scale_color_manual(values = colours.6) +
  ggplot2::geom_abline(intercept = 0, slope = 1) +
  ggplot2::facet_wrap(~ repunit)
print(plot)
ggplot2::ggsave(plot, filename = "DArTcap_HK_Assign_Rubias_Regions_correlation.png",
                width = 30, height = 15,units = "cm")
```

Rubias provenance of HKG samples

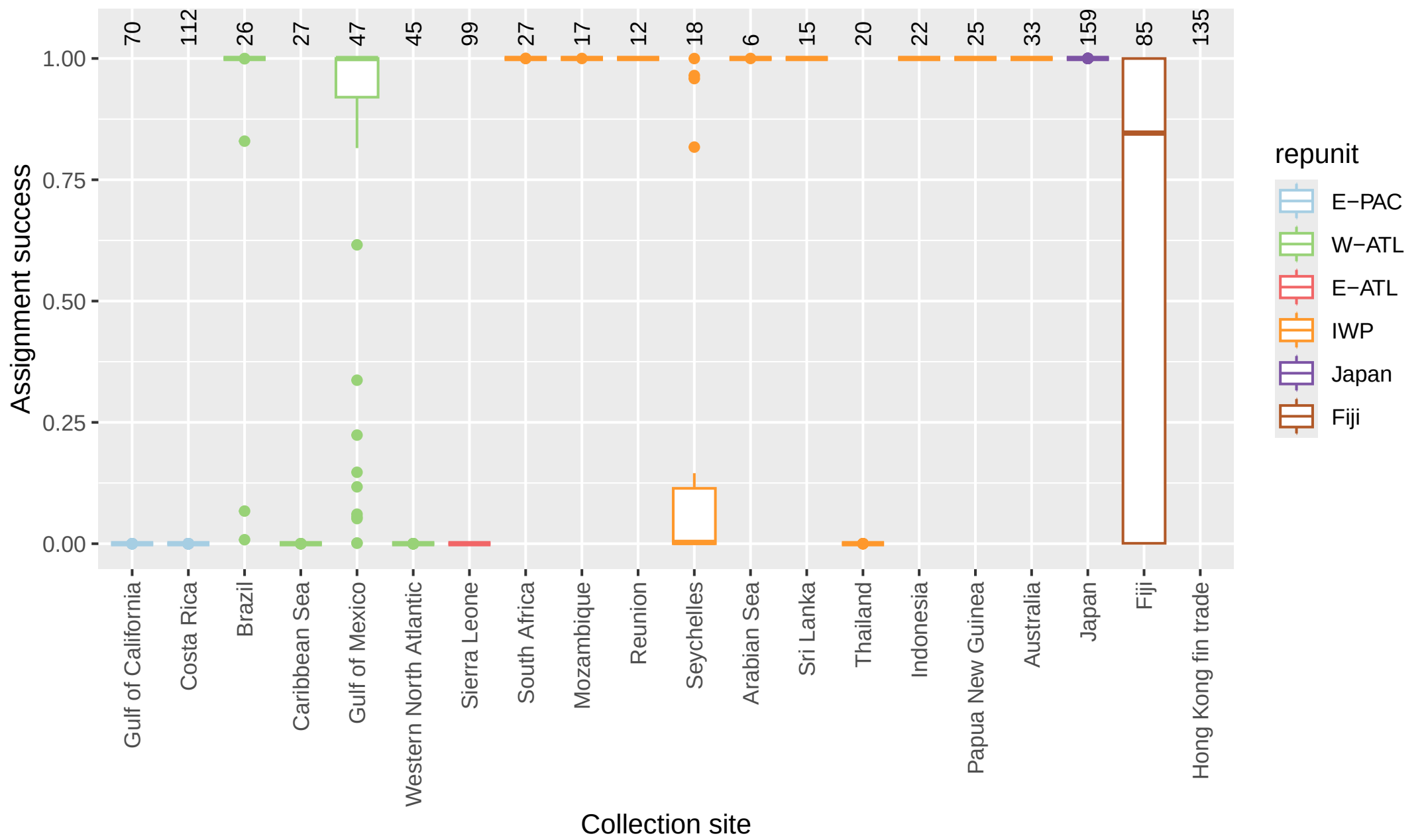

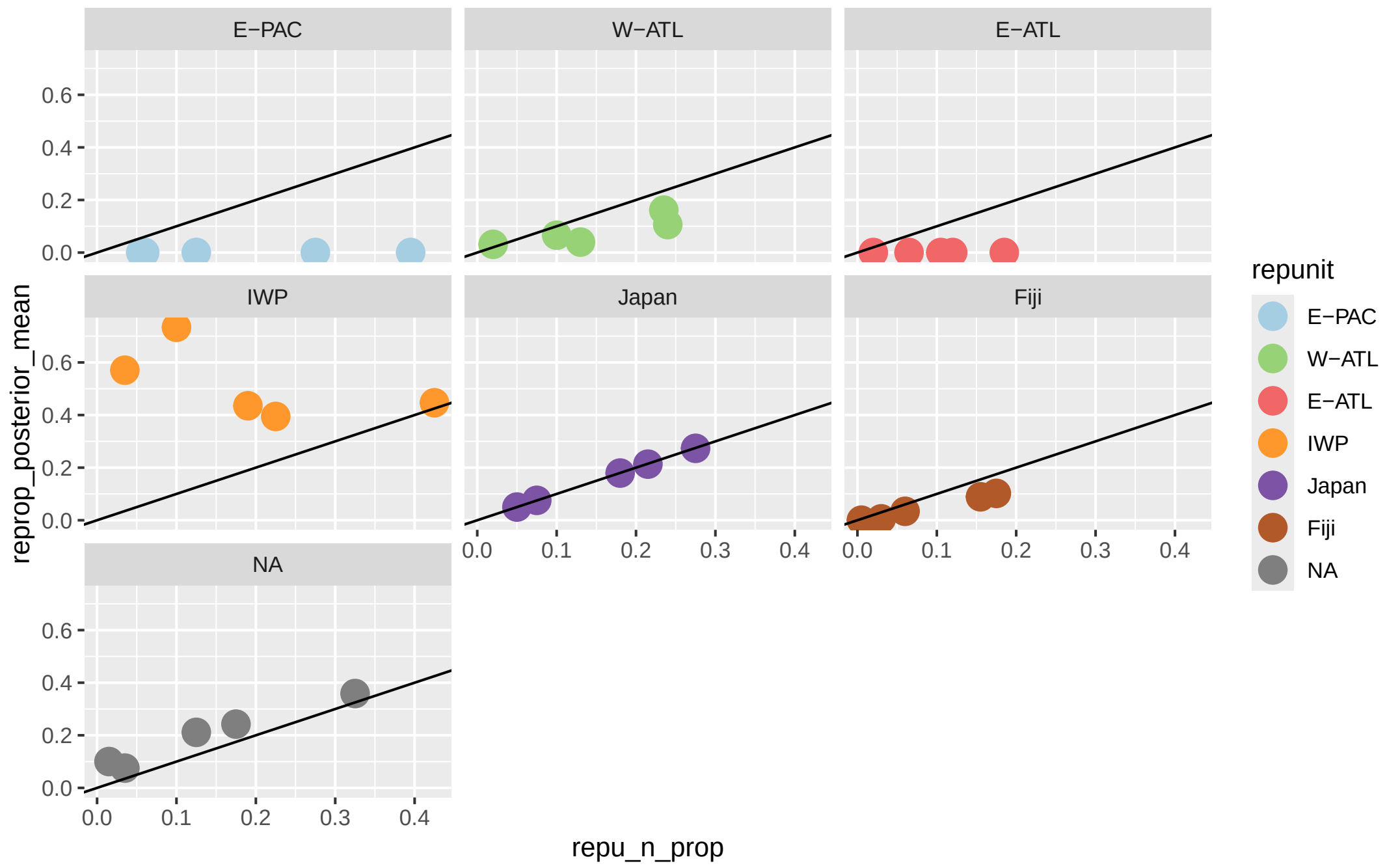


```
BS.dartcap.rubias2 <- BS.dartcap.HK.rubias2
BS.dartcap.rubias2 <- BS.dartcap.rubias2[match(BS.dartcap.HK.gl$ind.names,
                                          BS.dartcap.rubias2$indiv),]
# BS.dartcap.rubias2 <- BS.dartcap.rubias2[, !grepl("SPLIT_VEC",
#                                                   colnames(BS.dartcap.rubias2))]
```

```r
pop.levels2 <- c("Gulf of California","Costa Rica","Brazil", "Caribbean Sea",
                 "Gulf of Mexico", "Western North Atlantic",  "Sierra Leone",
                 "South Africa","Mozambique", "Reunion","Seychelles","Arabian Sea",
                 "Sri Lanka","Thailand","Indonesia","Papua New Guinea","Australia",
                 "Okinawa, Japan", "Urauchi River, Japan","Fiji", "Hong Kong fin trade")
group <- BS.dartcap.HK.gl$other$ind.metrics$Site
group[group == "Japan"] <- BS.dartcap.HK.gl$other$ind.metrics$Site2[group == "Japan"]
group[group == "Okinawa"] <- "Okinawa, Japan"
group[group == "Urauchi River"] <- "Urauchi River, Japan"
BS.dartcap.rubias2$collection <- factor(group, levels = pop.levels2)

region.levels <- c("E-PAC","W-ATL","E-ATL", "IWP", "Japan","Fiji", "Hong Kong fin trade")
region <- BS.dartcap.HK.gl$other$ind.metrics$Region
region[region %in% c("W-IO","N-IO", "E-IO", "E-IO/W-PAC", "W-PAC")] <- "IWP"
BS.dartcap.rubias2$repunit <- factor(region, levels = region.levels)

BS.dartcap.rubias2$sample_type[is.na(BS.dartcap.rubias2$repunit)] <- "mixture"
BS.dartcap.rubias2 <- BS.dartcap.rubias2[!BS.dartcap.rubias2$collection %in%
                                           "Okinawa, Japan",]

BS.dartcap.rubias2.HK <- BS.dartcap.rubias2[BS.dartcap.rubias2$collection %in%
                                              "Hong Kong fin trade",]
BS.dartcap.rubias2 <- BS.dartcap.rubias2[!BS.dartcap.rubias2$collection %in%
                                           "Hong Kong fin trade",]


BS.dartcap.rubias2 %<>%
  dplyr::mutate_all(as.character)
BS.dartcap.rubias2.HK %<>%
  dplyr::mutate_all(as.character)
BS.dartcap.rubias2.HK$repunit <- NA

mix_est.MCMC <- infer_mixture(
  reference = BS.dartcap.rubias2,
  mixture = BS.dartcap.rubias2.HK,
  gen_start_col = 5,
  method = "MCMC",
  reps = 10000,
  burn_in = 1000
  )
mix_est.PB <- infer_mixture(
  reference = BS.dartcap.rubias2,
  mixture = BS.dartcap.rubias2.HK,
  gen_start_col = 5,
  method = "PB",
  reps = 10000,
  burn_in = 1000
  )
mix_est.BR <- infer_mixture(
  reference = BS.dartcap.rubias2,
  mixture = BS.dartcap.rubias2.HK,
  gen_start_col = 5,
  method = "BR",
  reps = 10000,
  burn_in = 1000
  )
save(mix_est.MCMC, mix_est.PB, mix_est.BR,
```

```
     file = "DArTcap_ALL_Rubias_assigment_HK.Rdata")
```

```
load("DArTcap_ALL_Rubias_assigment_HK.Rdata")

rep_indiv_ests.MCMC <- mix_est.MCMC$indiv_posteriors %>%
  group_by(mixture_collection, indiv, repunit) %>%
  summarise(rep_pofz = sum(PofZ, na.rm = TRUE),
            rep_Z_score = mean(z_score, na.rm = TRUE))
map_rows.MCMC <- rep_indiv_ests.MCMC %>%
  group_by(indiv) %>%
  top_n(1, rep_pofz) %>%
  ungroup()
map_rows.MCMC$missing_data <- BS.dartcap.HK.gl.assign@other$ind.metrics$missingness
readr::write_csv(map_rows.MCMC,
                file = "0.HongKong_assignment_according_to_Rubias_MCMC.csv")

normo <- tibble(rep_Z_score = rnorm(1e06))
ggplot(map_rows.MCMC, aes(x = rep_Z_score)) +
  geom_density(colour = "blue") +
  geom_density(data = normo, colour = "black")


# Low z score correlates with missing data
map_rows.MCMC2 <- dplyr::filter(map_rows.MCMC, rep_Z_score < 5)
ggplot(map_rows.MCMC2, aes(x = rep_Z_score, y = missing_data)) +
  geom_point() +
  geom_smooth(method = "lm") +
  ggpubr::stat_cor(
    method = "pearson",
    label.x = min(map_rows.MCMC2$rep_Z_score),
    label.y = max(map_rows.MCMC2$missing_data)
  )


knitr::kable(map_rows.MCMC[map_rows.MCMC$rep_Z_score < -5,])
```

Markov-chain Monte Carlo conditional on the reference allele frequencies

mixture_collection	indiv	repunit	rep_pofz	rep_Z_score	missing_data
Hong Kong fin trade	CL-HK005.1	IWP	1.0000000	-13.228316	0.2852474
Hong Kong fin trade	CL-HK007	IWP	1.0000000	-6.916145	0.3660131
Hong Kong fin trade	CL-HK007.1	IWP	1.0000000	-5.903669	0.3295985
Hong Kong fin trade	CL-HK012	IWP	1.0000000	-15.743281	0.2254902
Hong Kong fin trade	CL-HK012.1	IWP	1.0000000	-18.230694	0.2357610
Hong Kong fin trade	CL-HK015	IWP	1.0000000	-7.847739	0.0410831
Hong Kong fin trade	CL-HK015.1	IWP	1.0000000	-8.590984	0.0522876
Hong Kong fin trade	CL-HK018	W-ATL	1.0000000	-9.277854	0.1125117
Hong Kong fin trade	CL-HK020	IWP	1.0000000	-24.029743	0.5107376
Hong Kong fin trade	CL-HK032	IWP	1.0000000	-8.939869	0.1255836
Hong Kong fin trade	CL-HK034	IWP	1.0000000	-9.226644	0.7231559
Hong Kong fin trade	CL-HK035	W-ATL	1.0000000	-11.237750	0.2609711
Hong Kong fin trade	CL-HK037	IWP	1.0000000	-15.591606	0.2100840
Hong Kong fin trade	CL-HK041	IWP	1.0000000	-7.036649	0.0382820

mixture_collection	indiv	repunit	rep_pofz	rep_Z_score	missing_data
Hong Kong fin trade	CL-HK054	IWP	1.0000000	-32.843256	0.3912232
Hong Kong fin trade	CL-HK069	IWP	1.0000000	-11.304455	0.0494865
Hong Kong fin trade	CL-HK070	IWP	1.0000000	-7.829391	0.2208217
Hong Kong fin trade	CL-HK100	IWP	1.0000000	-5.085646	0.1353875
Hong Kong fin trade	CL-HK105	IWP	1.0000000	-10.876880	0.0812325
Hong Kong fin trade	CL-HK106	IWP	1.0000000	-8.069093	0.5340803
Hong Kong fin trade	CL-HK114	IWP	1.0000000	-6.321026	0.0835668
Hong Kong fin trade	CL-HK119	IWP	1.0000000	-21.832024	0.5873016
Hong Kong fin trade	CL-HK137	E-ATL	1.0000000	-Inf	0.7366947
Hong Kong fin trade	CL-HK142	IWP	1.0000000	-13.047113	0.2030812
Hong Kong fin trade	CL-HK144	IWP	0.9999999	-5.575656	0.6115780

```
knitr::kable(map_rows.MCMC[map_rows.MCMC$rep_Z_score > 5,])
```

mixture_collection	indiv	repunit	rep_pofz	rep_Z_score	missing_data
Hong Kong fin trade	CL-HK019	E-ATL	1.0000000	Inf	0.5186741
Hong Kong fin trade	CL-HK077	E-PAC	1.0000000	66.31108	0.0135387
Hong Kong fin trade	CL-HK085	E-PAC	1.0000000	63.09005	0.0070028
Hong Kong fin trade	CL-HK098	E-PAC	1.0000000	63.55109	0.0396825
Hong Kong fin trade	CL-HK099	E-ATL	0.9999998	Inf	0.7469655
Hong Kong fin trade	CL-HK126	E-ATL	1.0000000	Inf	0.4775910
Hong Kong fin trade	CL-HK141	E-PAC	1.0000000	67.20507	0.0079365

```
map_rows.MCMC <- mix_est.MCMC$indiv_posteriors %>%
  group_by(indiv) %>%
  top_n(1, PofZ) %>%
  ungroup()
readr::write_csv(map_rows.MCMC[,-10],
                 file = "0.HongKong_assignment_according_to_Rubias_MCMC_2.csv")
```

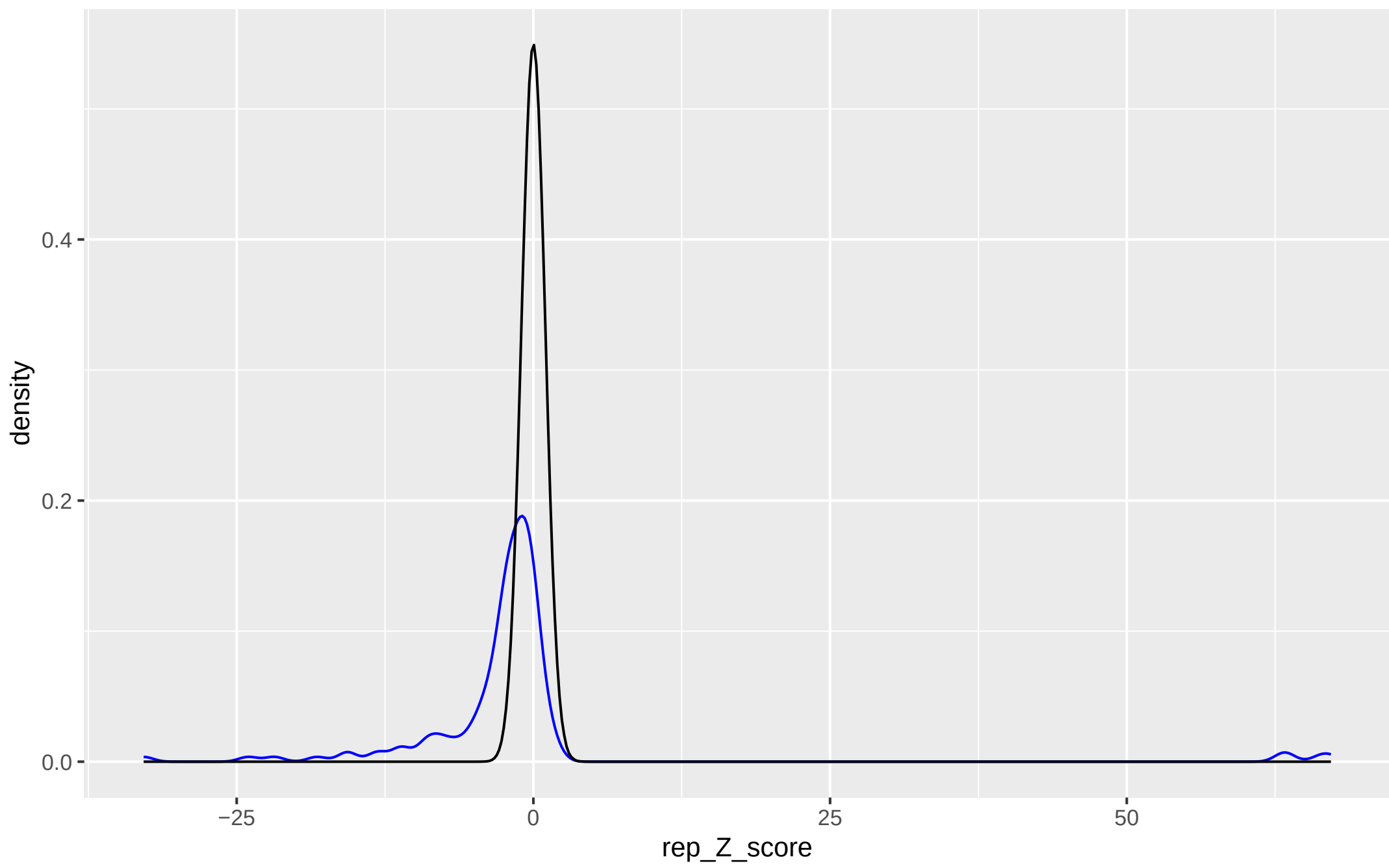

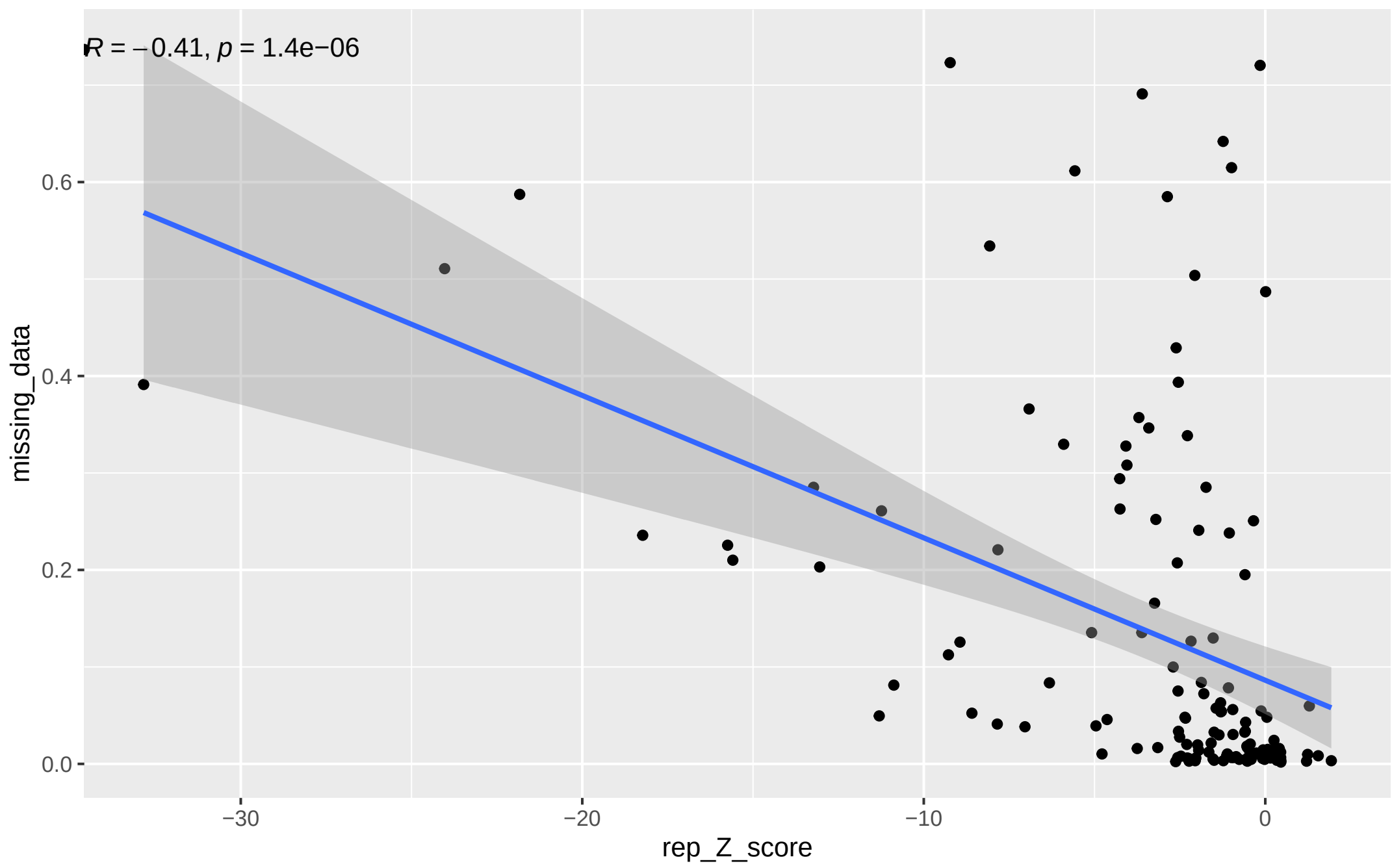


```
rep_indiv_ests.PB <- mix_est.PB$indiv_posteriors %>%
  group_by(mixture_collection, indiv, repunit) %>%
  summarise(rep_pofz = sum(PofZ, na.rm = TRUE),
            rep_Z_score = mean(z_score, na.rm = TRUE))
map_rows.PB <- rep_indiv_ests.MCMC %>%
  group_by(indiv) %>%
  top_n(1, rep_pofz) %>%
  ungroup()
map_rows.PB$missing_data <- BS.dartcap.HK.gl.assign@other$ind.metrics$missingness
readr::write_csv(map_rows.PB,
                 file = "0.HongKong_assignment_according_to_Rubias_PB.csv")

normo <- tibble(rep_Z_score = rnorm(1e06))
ggplot(map_rows.PB, aes(x = rep_Z_score)) +
  geom_density(colour = "blue") +
  geom_density(data = normo, colour = "black")

# Low z score correlates with missing data
map_rows.PB2 <- dplyr::filter(map_rows.PB, rep_Z_score < 5)
ggplot(map_rows.PB2, aes(x = rep_Z_score, y = missing_data)) +
  geom_point() +
  geom_smooth(method = "lm") +
  ggpubr::stat_cor(
    method = "pearson",
    label.x = min(map_rows.PB2$rep_Z_score),
    label.y = max(map_rows.PB2$missing_data)
  )

knitr::kable(map_rows.PB[map_rows.PB$rep_Z_score < -5,])
```

Parametric bootstrapping correction

mixture_collection	indiv	repunit	rep_pofz	rep_Z_score	missing_data
Hong Kong fin trade	CL-HK005.1	IWP	1.0000000	-13.228316	0.2852474
Hong Kong fin trade	CL-HK007	IWP	1.0000000	-6.916145	0.3660131
Hong Kong fin trade	CL-HK007.1	IWP	1.0000000	-5.903669	0.3295985
Hong Kong fin trade	CL-HK012	IWP	1.0000000	-15.743281	0.2254902
Hong Kong fin trade	CL-HK012.1	IWP	1.0000000	-18.230694	0.2357610
Hong Kong fin trade	CL-HK015	IWP	1.0000000	-7.847739	0.0410831
Hong Kong fin trade	CL-HK015.1	IWP	1.0000000	-8.590984	0.0522876
Hong Kong fin trade	CL-HK018	W-ATL	1.0000000	-9.277854	0.1125117
Hong Kong fin trade	CL-HK020	IWP	1.0000000	-24.029743	0.5107376
Hong Kong fin trade	CL-HK032	IWP	1.0000000	-8.939869	0.1255836
Hong Kong fin trade	CL-HK034	IWP	1.0000000	-9.226644	0.7231559
Hong Kong fin trade	CL-HK035	W-ATL	1.0000000	-11.237750	0.2609711
Hong Kong fin trade	CL-HK037	IWP	1.0000000	-15.591606	0.2100840
Hong Kong fin trade	CL-HK041	IWP	1.0000000	-7.036649	0.0382820
Hong Kong fin trade	CL-HK054	IWP	1.0000000	-32.843256	0.3912232
Hong Kong fin trade	CL-HK069	IWP	1.0000000	-11.304455	0.0494865
Hong Kong fin trade	CL-HK070	IWP	1.0000000	-7.829391	0.2208217
Hong Kong fin trade	CL-HK100	IWP	1.0000000	-5.085646	0.1353875
Hong Kong fin trade	CL-HK105	IWP	1.0000000	-10.876880	0.0812325
Hong Kong fin trade	CL-HK106	IWP	1.0000000	-8.069093	0.5340803
Hong Kong fin trade	CL-HK114	IWP	1.0000000	-6.321026	0.0835668
Hong Kong fin trade	CL-HK119	IWP	1.0000000	-21.832024	0.5873016
Hong Kong fin trade	CL-HK137	E-ATL	1.0000000	-Inf	0.7366947
Hong Kong fin trade	CL-HK142	IWP	1.0000000	-13.047113	0.2030812
Hong Kong fin trade	CL-HK144	IWP	0.9999999	-5.575656	0.6115780

```
knitr::kable(map_rows.PB[map_rows.PB$rep_Z_score > 5,])
```

mixture_collection	indiv	repunit	rep_pofz	rep_Z_score	missing_data
Hong Kong fin trade	CL-HK019	E-ATL	1.0000000	Inf	0.5186741
Hong Kong fin trade	CL-HK077	E-PAC	1.0000000	66.31108	0.0135387
Hong Kong fin trade	CL-HK085	E-PAC	1.0000000	63.09005	0.0070028
Hong Kong fin trade	CL-HK098	E-PAC	1.0000000	63.55109	0.0396825
Hong Kong fin trade	CL-HK099	E-ATL	0.9999998	Inf	0.7469655
Hong Kong fin trade	CL-HK126	E-ATL	1.0000000	Inf	0.4775910
Hong Kong fin trade	CL-HK141	E-PAC	1.0000000	67.20507	0.0079365

```
map_rows.PB <- mix_est.PB$indiv_posteriors %>%
  group_by(indiv) %>%
  top_n(1, PofZ ) %>%
  ungroup()
readr::write_csv(map_rows.PB[,-10],
                 file = "0.HongKong_assignment_according_to_Rubias_PB_2.csv")
```

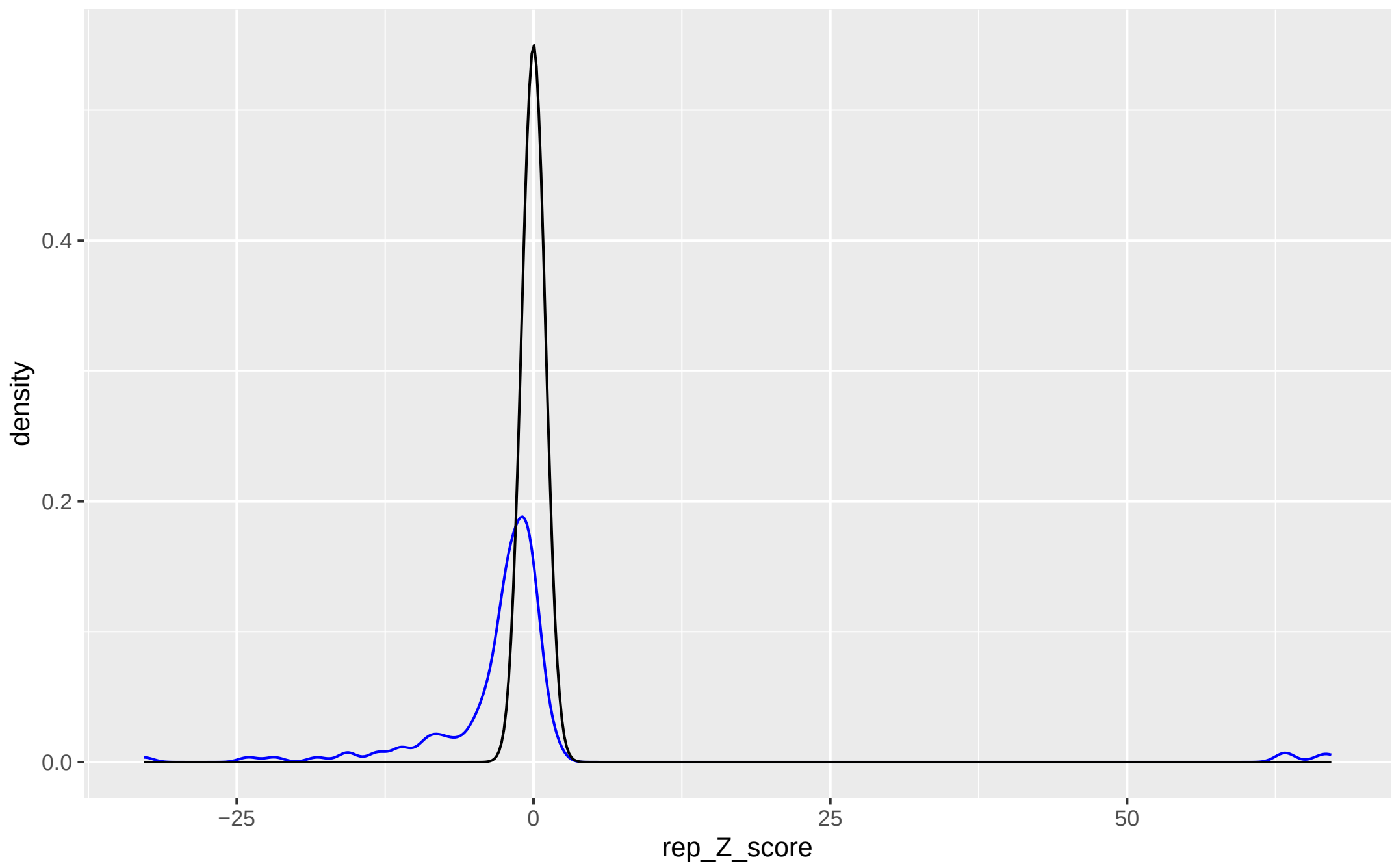

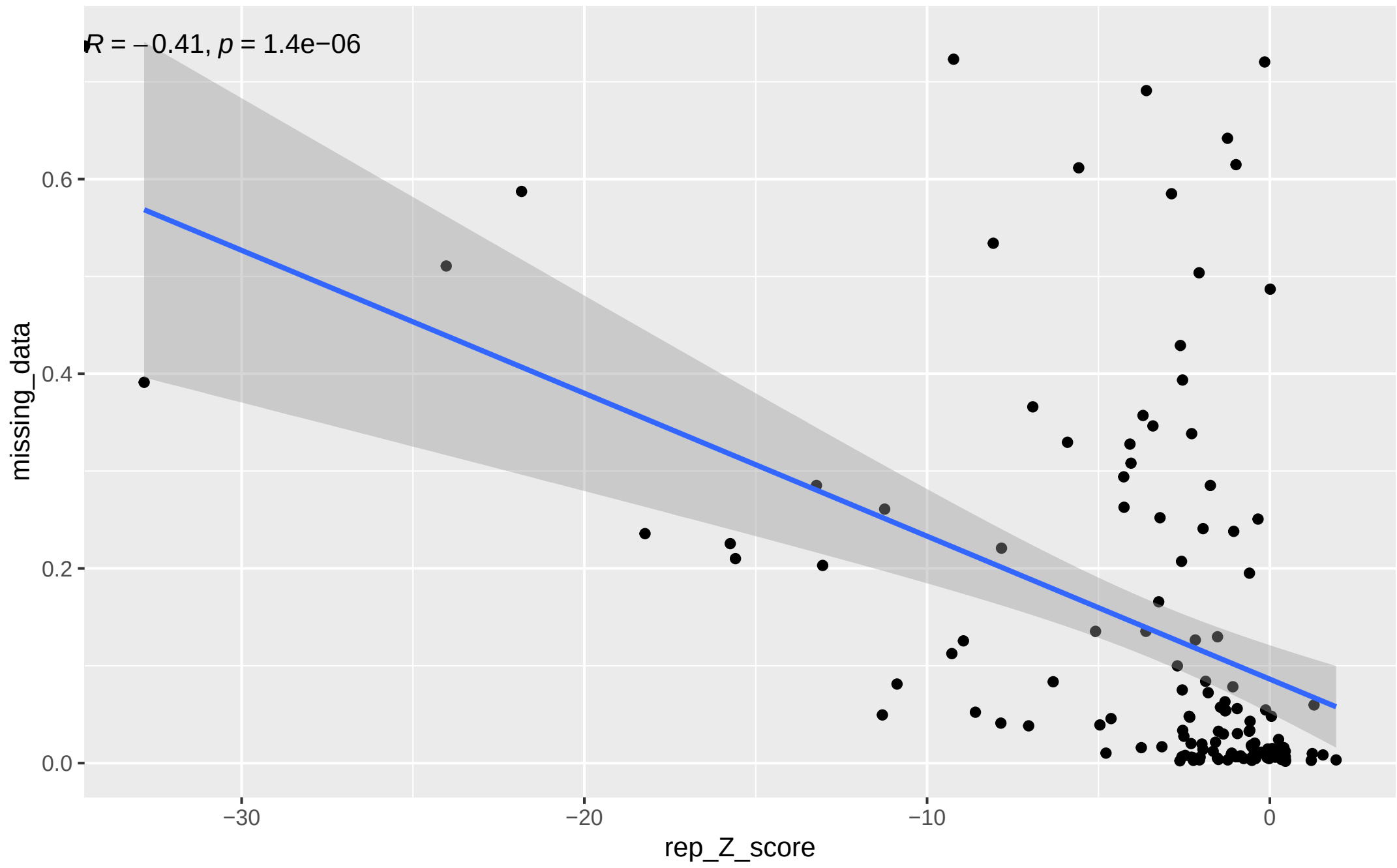

Baseline Resampling MCMC sweeps while simulating reference allele frequencies using the genotypes of mixture individuals and allocations from the previous sweep.

```
rep_indiv_ests.BR <- mix_est.BR$indiv_posteriors %>%
  group_by(mixture_collection, indiv, repunit) %>%
  summarise(rep_pofz = sum(PofZ, na.rm = TRUE),
            rep_Z_score = mean(z_score, na.rm = TRUE))
map_rows.BR <- rep_indiv_ests.BR %>%
  group_by(indiv) %>%
  top_n(1, rep_pofz) %>%
  ungroup()

df <- data.frame(indiv   = BS.dartcap.HK.gl.assign@other$ind.metrics$INDIVIDUALS,
                 missing_data   = BS.dartcap.HK.gl.assign@other$ind.metrics$missingness)
```

```
map_rows.BR <- dplyr::left_join(map_rows.BR, df, by = "indiv")
readr::write_csv(map_rows.BR,
                 file = "0.HongKong_assignment_according_to_Rubias_BR.csv")

normo <- tibble(rep_Z_score = rnorm(1e06))
ggplot(map_rows.BR, aes(x = rep_Z_score)) +
  geom_density(colour = "blue") +
  geom_density(data = normo, colour = "black")
# map_rows.BR[map_rows.BR$rep_Z_score < -5,]
# map_rows.BR[map_rows.BR$rep_Z_score > 5,]


map_rows.BR <- mix_est.BR$indiv_posteriors %>%
  group_by(indiv) %>%
  top_n(1, PofZ ) %>%
  ungroup()
readr::write_csv(map_rows.BR[,-10],
                 file = "0.HongKong_assignment_according_to_Rubias_BR_2.csv")
```

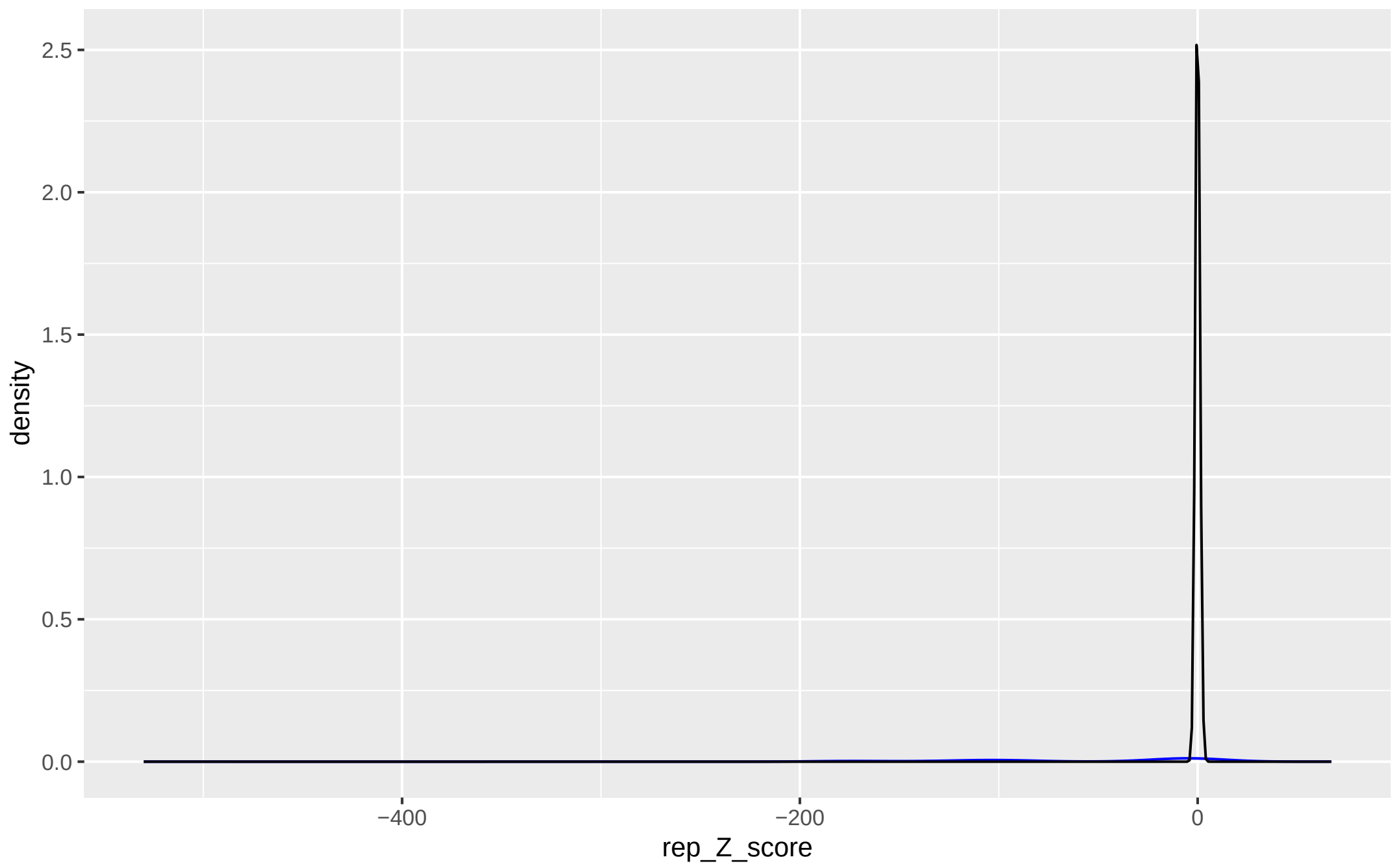


```
region.levels <- c("E-PAC","W-ATL","E-ATL", "IWP", "Japan","Fiji")
map_rows.MCMC$repunit <- factor(map_rows.MCMC$repunit, levels = region.levels)
knitr::kable(as.matrix(t(table(map_rows.MCMC$repunit))),
             caption = "Rubias: Provencance of Hong Kong samples according to
             MCMC algorithm") %>%
  kableExtra::kable_styling(full_width = FALSE)
```

Summary

Table 30: Rubias: Provencance of Hong Kong samples according to MCMC algorithm

E-PAC	W-ATL	E-ATL	IWP	Japan	Fiji
4	31	4	101	0	0

```
map_rows.PB$repunit <- factor(map_rows.PB$repunit, levels = region.levels)
knitr::kable(as.matrix(t(table(map_rows.PB$repunit))),
             caption = "Rubias: Provencance of Hong Kong samples according to
             parametric bootstrapping algorithm") %>%
  kableExtra::kable_styling(full_width = FALSE)
```

Table 31: Rubias: Provencance of Hong Kong samples according to parametric bootstrapping algorithm

E-PAC	W-ATL	E-ATL	IWP	Japan	Fiji
4	31	4	101	0	0

```
knitr::kable(table(map_rows.MCMC$repunit, map_rows.PB$repunit),
             caption = "Rubias: Provencance of Hong Kong samples comparing
             the MCMC and parametric bootstrapping methods") %>%
  kableExtra::kable_styling(full_width = FALSE)
```

Table 32: Rubias: Provencance of Hong Kong samples comparing the MCMC and parametric bootstrapping methods

	E-PAC	W-ATL	E-ATL	IWP	Japan	Fiji
E-PAC	4	0	0	0	0	0
W-ATL	0	31	0	0	0	0
E-ATL	0	0	4	0	0	0
IWP	0	0	0	101	0	0
Japan	0	0	0	0	0	0
Fiji	0	0	0	0	0	0

Compare DAPC to rubias

For rubias, MCMC and PB gave the same results, so I compare against MCMC.

for DAPC, I use K=6 as the most likely number of groups.

```
DAPC <- readr::read_tsv(paste0("BS_HK_Group_assignment2_K6.tsv"))
region.levels <- c("E-PAC", "W-ATL", "E-ATL", "IWP","Japan", "Fiji")
DAPC$assigned_pop <- factor(DAPC$assigned_pop, levels = region.levels)

knitr::kable(table(DAPC$assigned_pop, map_rows.MCMC$repunit),
             caption = "6 reference populations: Comparing the provencance of Hong Kong samples for
             the DAPC and MCMC methods") %>%
  kableExtra::kable_styling(full_width = FALSE)
```

Table 33: 6 reference populations: Comparing the provencance of Hong Kong samples for the DAPC and MCMC methods

	E-PAC	W-ATL	E-ATL	IWP	Japan	Fiji
E-PAC	4	0	0	0	0	0

W-ATL	0	31	0	0	0	0
E-ATL	0	0	0	0	0	0
IWP	0	0	4	101	0	0
Japan	0	0	0	0	0	0
Fiji	0	0	0	0	0	0

Reference

Devloo-Delva F, Burridge CP, Kyne PM, Brunnschweiler JM, Chapman DD, Charvet P et al. (2023). From rivers to ocean basins: The role of ocean barriers and philopatry in the genetic structuring of a cosmopolitan coastal predator. Ecol Evol 13(2): e9837.

Foster, S. D., Feutry, P., Grewe, P. M., Berry, O., Hui, F. K., & Davies, C. R. (2018). Reliably discriminating stock structure with genetic markers: Mixture models with robust and fast computation. Molecular Ecology Resources, 1–16.

Puechmaille, S. J. (2016). The program structure does not reliably recover the correct population structure when sampling is uneven: subsampling and new estimators alleviate the problem. Molecular Ecology Resources, 16(3), 608-627.

Citations for packages

```
packages <- c(
  "radiator",
  "ggplot2",
  "adegenet",
  "dartRverse",
  "ggplot2",
  "gtools",
  "kableExtra",
  "knitr",
  "rubias",
  "strataG"
)

for (P in packages) {
  p <- utils::citation(P)
  print(attr(unclass(p)[[1]], "textVersion"))
}
```

```
## [1] "Gosselin, T. (2020). radiator: RADseq Data Exploration, Manipulation and Visualization using
## [1] "H. Wickham. ggplot2: Elegant Graphics for Data Analysis. Springer-Verlag New York, 2016."
## [1] "Jombart, T. (2008) adegenet: a R package for the multivariate analysis of genetic markers. B
## [1] "Gruber, B, Unmack, PJ, Berry, OF, Georges, A. (2018). dartr: An r package to facilitate anal
## [1] "H. Wickham. ggplot2: Elegant Graphics for Data Analysis. Springer-Verlag New York, 2016."
## NULL
## NULL
## NULL
## NULL
## [1] "Archer, F. I., Adams, P. E. and Schneiders, B. B. (2016) strataG: An R package for manipulat
```

Session info

```
sessioninfo::session_info()
```

```
## - Session info ---------------------------------------------------------------------------------
##  setting  value
##  version  R version 4.4.2 (2024-10-31)
##  os       Ubuntu 22.04.5 LTS
```

```
##  system   x86_64, linux-gnu
##  ui       X11
##  language (EN)
##  collate  en_US.UTF-8
##  ctype    en_US.UTF-8
##  tz       Etc/UTC
##  date     2026-05-05
##  pandoc   3.2 @ /usr/lib/rstudio-server/bin/quarto/bin/tools/x86_64/ (via rmarkdown)
##  quarto   1.6.40 @ /usr/local/bin/quarto
##
## - Packages -------------------------------------------------------------------------------------
##  package           * version    date (UTC) lib source
##  ade4              * 1.7-23     2025-02-14 [1] RSPM (R 4.4.0)
##  adegenet          * 2.1.11     2025-02-06 [1] RSPM (R 4.4.0)
##  adehabitatMA        0.3.17     2024-09-06 [1] RSPM (R 4.4.0)
##  ape               * 5.8-1      2024-12-16 [1] RSPM (R 4.4.0)
##  apex              * 1.0.6      2024-01-31 [1] RSPM (R 4.4.0)
##  arsenal             3.6.3      2021-06-04 [1] RSPM (R 4.4.0)
##  assignPOP         * 1.3.1      2025-09-12 [1] RSPM (R 4.4.0)
##  backports           1.5.1      2026-04-03 [1] RSPM (R 4.4.0)
##  base64enc           0.1-3      2015-07-28 [2] RSPM (R 4.4.0)
##  BiocGenerics        0.52.0     2024-10-29 [1] Bioconductor 3.20 (R 4.4.2)
##  bit                 4.6.0      2025-03-06 [1] RSPM (R 4.4.0)
##  bit64               4.8.0      2026-04-21 [1] RSPM (R 4.4.0)
##  bitops              1.0-9      2024-10-03 [2] RSPM (R 4.4.0)
##  blob                1.2.4      2023-03-17 [1] RSPM (R 4.4.0)
##  cachem              1.1.0      2024-05-16 [2] RSPM (R 4.4.0)
##  caret               7.0-1      2024-12-10 [1] RSPM (R 4.4.0)
##  checkmate           2.3.2      2024-07-29 [1] RSPM (R 4.4.0)
##  class               7.3-22     2023-05-03 [3] CRAN (R 4.4.2)
##  cli                 3.6.6      2026-04-09 [1] RSPM (R 4.4.0)
##  cluster             2.1.6      2023-12-01 [3] CRAN (R 4.4.2)
##  coda                0.19-4.1   2024-01-31 [1] RSPM (R 4.4.0)
##  codetools           0.2-20     2024-03-31 [3] CRAN (R 4.4.2)
##  colorspace          2.1-1      2024-07-26 [1] RSPM (R 4.4.0)
##  crayon              1.5.3      2024-06-20 [2] RSPM (R 4.4.0)
##  dartR.base        * 1.0.5      2025-03-04 [1] RSPM (R 4.4.2)
##  dartR.data        * 1.0.8      2024-05-30 [1] RSPM (R 4.4.0)
##  dartR.sexlinked   * 1.2.2      2026-03-20 [1] RSPM (R 4.4.2)
##  dartRverse        * 1.0.6      2025-02-18 [1] RSPM (R 4.4.0)
##  data.table        * 1.17.8     2025-07-10 [1] RSPM (R 4.4.0)
##  DBI                 1.2.3      2024-06-02 [1] RSPM (R 4.4.0)
##  devtools            2.4.5      2022-10-11 [1] RSPM (R 4.4.0)
##  dichromat           2.0-0.1    2022-05-02 [1] RSPM (R 4.4.0)
##  digest              0.6.39     2025-11-19 [1] RSPM (R 4.4.0)
##  doParallel          1.0.17     2022-02-07 [1] RSPM (R 4.4.0)
##  dplyr             * 1.2.1      2026-04-03 [1] RSPM (R 4.4.0)
##  e1071               1.7-16     2024-09-16 [1] RSPM (R 4.4.0)
##  ellipsis            0.3.2      2021-04-29 [1] RSPM (R 4.4.0)
##  evaluate            1.0.3      2025-01-10 [2] RSPM (R 4.4.0)
##  farver              2.1.2      2024-05-13 [1] RSPM (R 4.4.0)
##  fastmap             1.2.0      2024-05-15 [2] RSPM (R 4.4.0)
##  fastmatch           1.1-6      2024-12-23 [1] RSPM (R 4.4.0)
##  fontBitstreamVera   0.1.1      2017-02-01 [1] RSPM (R 4.4.0)
##  fontLiberation      0.1.0      2016-10-15 [1] RSPM (R 4.4.0)
##  fontquiver          0.2.1      2017-02-01 [1] RSPM (R 4.4.0)
##  forcats           * 1.0.1      2025-09-25 [1] RSPM (R 4.4.0)
##  foreach             1.5.2      2022-02-02 [1] RSPM (R 4.4.0)
```

```
##  foreign          * 0.8-87     2024-06-26 [3] CRAN (R 4.4.2)
##  formatR          * 1.14       2023-01-17 [1] RSPM (R 4.4.0)
##  Formula            1.2-5      2023-02-24 [1] RSPM (R 4.4.0)
##  fossil           * 0.4.0      2020-03-23 [1] RSPM (R 4.4.0)
##  fs                 1.6.5      2024-10-30 [2] RSPM (R 4.4.0)
##  future             1.70.0     2026-03-14 [1] RSPM (R 4.4.0)
##  future.apply       1.20.2     2026-02-20 [1] RSPM (R 4.4.0)
##  gdistance          1.6.5      2025-09-27 [1] RSPM (R 4.4.0)
##  gdsfmt             1.42.1     2024-12-26 [1] Bioconductor 3.20 (R 4.4.2)
##  gdtools            0.5.0      2026-02-09 [1] RSPM (R 4.4.0)
##  generics           0.1.4      2025-05-09 [1] RSPM (R 4.4.0)
##  ggformula          1.0.1      2026-01-17 [1] RSPM (R 4.4.0)
##  ggiraph            0.9.6      2026-02-21 [1] RSPM (R 4.4.0)
##  ggplot2          * 4.0.3      2026-04-22 [1] RSPM (R 4.4.0)
##  ggridges           0.5.7      2025-08-27 [1] RSPM (R 4.4.0)
##  globals            0.19.1     2026-03-13 [1] RSPM (R 4.4.0)
##  glue               1.8.1      2026-04-17 [1] RSPM (R 4.4.0)
##  gower              1.0.2      2024-12-17 [1] RSPM (R 4.4.0)
##  gridExtra        * 2.3        2017-09-09 [1] RSPM (R 4.4.0)
##  gtable             0.3.6      2024-10-25 [1] RSPM (R 4.4.0)
##  gtools           * 3.9.5      2023-11-20 [1] RSPM (R 4.4.0)
##  haplo.stats        1.9.7      2024-09-19 [1] RSPM (R 4.4.0)
##  haplotypes       * 1.1.3.1    2023-07-15 [1] RSPM (R 4.4.0)
##  hardhat            1.4.3      2026-04-04 [1] RSPM (R 4.4.0)
##  haven              2.5.5      2025-05-30 [1] RSPM (R 4.4.0)
##  Hmisc              5.2-3      2025-03-16 [1] RSPM (R 4.4.0)
##  hms                1.1.4      2025-10-17 [1] RSPM (R 4.4.0)
##  htmlTable          2.4.3      2024-07-21 [1] RSPM (R 4.4.0)
##  htmltools          0.5.8.1    2024-04-04 [2] RSPM (R 4.4.0)
##  htmlwidgets        1.6.4      2023-12-06 [1] RSPM (R 4.4.0)
##  httpuv             1.6.15     2024-03-26 [2] RSPM (R 4.4.0)
##  httr               1.4.7      2023-08-15 [1] RSPM (R 4.4.0)
##  igraph             2.1.4      2025-01-23 [1] RSPM (R 4.4.0)
##  ipred              0.9-15     2024-07-18 [1] RSPM (R 4.4.0)
##  iterators          1.0.14     2022-02-05 [1] RSPM (R 4.4.0)
##  kableExtra       * 1.4.0      2024-01-24 [1] RSPM (R 4.4.0)
##  knitr            * 1.50       2025-03-16 [2] RSPM (R 4.4.0)
##  labeling           0.4.3      2023-08-29 [1] RSPM (R 4.4.0)
##  labelled           2.16.0     2025-10-22 [1] RSPM (R 4.4.0)
##  later              1.4.2      2025-04-08 [2] RSPM (R 4.4.0)
##  lattice            0.22-6     2024-03-20 [3] CRAN (R 4.4.2)
##  lava               1.8.1      2025-01-12 [1] RSPM (R 4.4.0)
##  lifecycle          1.0.5      2026-01-08 [1] RSPM (R 4.4.0)
##  listenv            0.10.1     2026-03-10 [1] RSPM (R 4.4.0)
##  lubridate        * 1.9.5      2026-02-04 [1] RSPM (R 4.4.0)
##  magrittr         * 2.0.5      2026-04-04 [1] RSPM (R 4.4.0)
##  maps             * 3.4.3      2025-05-26 [1] RSPM (R 4.4.0)
##  marmap           * 1.0.12     2025-07-30 [1] RSPM (R 4.4.0)
##  MASS               7.3-61     2024-06-13 [3] CRAN (R 4.4.2)
##  Matrix             1.7-1      2024-10-18 [3] CRAN (R 4.4.2)
##  MatrixModels       0.5-4      2025-03-26 [1] RSPM (R 4.4.0)
##  memoise            2.0.1      2021-11-26 [2] RSPM (R 4.4.0)
##  mgcv               1.9-1      2023-12-21 [3] CRAN (R 4.4.2)
##  mime               0.13       2025-03-17 [2] RSPM (R 4.4.0)
##  miniUI             0.1.2      2025-04-17 [1] RSPM (R 4.4.0)
##  ModelMetrics       1.2.2.2    2020-03-17 [1] RSPM (R 4.4.0)
##  mosaicCore         0.9.5      2025-07-30 [1] RSPM (R 4.4.0)
##  multcomp           1.4-28     2025-01-29 [1] RSPM (R 4.4.0)
```

```
##  mvbutils         * 2.8.232    2018-12-12 [1] RSPM (R 4.4.0)
##  mvtnorm            1.3-3      2025-01-10 [1] RSPM (R 4.4.0)
##  ncdf4              1.24       2025-03-25 [1] RSPM (R 4.4.0)
##  network            1.19.0     2024-12-09 [1] RSPM (R 4.4.0)
##  nlme               3.1-166    2024-08-14 [3] CRAN (R 4.4.2)
##  nnet               7.3-19     2023-05-03 [3] CRAN (R 4.4.2)
##  parallelly         1.47.0     2026-04-17 [1] RSPM (R 4.4.0)
##  patchwork          1.3.1      2025-06-21 [1] RSPM (R 4.4.0)
##  pegas            * 1.3        2023-12-13 [1] RSPM (R 4.4.0)
##  permute          * 0.9-8      2025-06-25 [1] RSPM (R 4.4.0)
##  phangorn         * 2.12.1     2024-09-17 [1] RSPM (R 4.4.0)
##  pillar             1.11.1     2025-09-17 [1] RSPM (R 4.4.0)
##  pkgbuild           1.4.7      2025-03-24 [2] RSPM (R 4.4.0)
##  pkgconfig          2.0.3      2019-09-22 [1] RSPM (R 4.4.0)
##  pkgload            1.4.0      2024-06-28 [2] RSPM (R 4.4.0)
##  plotrix            3.8-4      2023-11-10 [1] RSPM (R 4.4.0)
##  plyr               1.8.9      2023-10-02 [1] RSPM (R 4.4.0)
##  poisbinom          1.0.2      2025-06-02 [1] RSPM (R 4.4.0)
##  polspline          1.1.25     2024-05-10 [1] RSPM (R 4.4.0)
##  pROC               1.18.5     2023-11-01 [1] RSPM (R 4.4.0)
##  prodlim            2025.04.28 2025-04-28 [1] RSPM (R 4.4.0)
##  profvis            0.4.0      2024-09-20 [1] RSPM (R 4.4.0)
##  promises           1.3.2      2024-11-28 [2] RSPM (R 4.4.0)
##  proxy              0.4-27     2022-06-09 [1] RSPM (R 4.4.0)
##  purrr            * 1.1.0      2025-07-10 [1] RSPM (R 4.4.0)
##  quadprog           1.5-8      2019-11-20 [1] RSPM (R 4.4.0)
##  quantreg           6.1        2025-03-10 [1] RSPM (R 4.4.0)
##  R6                 2.6.1      2025-02-15 [2] RSPM (R 4.4.0)
##  radiator         * 1.4.0      2026-05-04 [1] Github (thierrygosselin/radiator@9033c86)
##  ragg               1.4.0      2025-04-10 [1] RSPM (R 4.4.0)
##  randomForest       4.7-1.2    2024-09-22 [1] RSPM (R 4.4.0)
##  raster             3.6-32     2025-03-28 [1] RSPM (R 4.4.0)
##  RColorBrewer       1.1-3      2022-04-03 [1] RSPM (R 4.4.0)
##  Rcpp               1.1.1-1.1  2026-04-24 [1] RSPM (R 4.4.0)
##  RcppParallel       5.1.11-2   2026-03-05 [1] RSPM (R 4.4.0)
##  RCurl              1.98-1.18  2026-03-21 [1] RSPM (R 4.4.0)
##  readr            * 2.2.0      2026-02-19 [1] RSPM (R 4.4.0)
##  recipes            1.3.2      2026-04-02 [1] RSPM (R 4.4.0)
##  remotes            2.5.0      2024-03-17 [1] RSPM (R 4.4.0)
##  reshape2           1.4.4      2020-04-09 [1] RSPM (R 4.4.0)
##  rlang              1.2.0      2026-04-06 [1] RSPM (R 4.4.0)
##  rmarkdown          2.29       2024-11-04 [2] RSPM (R 4.4.0)
##  rms                8.0-0      2025-04-04 [1] RSPM (R 4.4.0)
##  rpart              4.1.23     2023-12-05 [3] CRAN (R 4.4.2)
##  RSQLite            2.4.1      2025-06-08 [1] RSPM (R 4.4.0)
##  rstudioapi         0.18.0     2026-01-16 [1] RSPM (R 4.4.0)
##  rubias           * 0.4.1      2026-05-04 [1] Github (eriqande/rubias@68f3d83)
##  S7                 0.2.2      2026-04-22 [1] RSPM (R 4.4.0)
##  sandwich           3.1-1      2024-09-15 [1] RSPM (R 4.4.0)
##  scales             1.4.0      2025-04-24 [1] RSPM (R 4.4.0)
##  seqinr             4.2-36     2023-12-08 [1] RSPM (R 4.4.0)
##  sessioninfo        1.2.3      2025-02-05 [1] RSPM (R 4.4.0)
##  shape              1.4.6.1    2024-02-23 [1] RSPM (R 4.4.0)
##  shapefiles       * 0.7.2      2022-08-25 [1] RSPM (R 4.4.0)
##  shiny              1.10.0     2024-12-14 [2] RSPM (R 4.4.0)
##  sna                2.8        2024-09-08 [1] RSPM (R 4.4.0)
##  SNPassoc           2.1-2      2024-10-28 [1] RSPM (R 4.4.0)
##  SNPRelate          1.40.0     2024-10-29 [1] Bioconductor 3.20 (R 4.4.2)
```

```
##  snpStats            1.56.0     2024-10-29 [1] Bioconductor 3.20 (R 4.4.2)
##  sp                * 2.2-0      2025-02-01 [1] RSPM (R 4.4.0)
##  SparseM             1.84-2     2024-07-17 [1] RSPM (R 4.4.0)
##  StAMPP              1.6.3      2021-08-08 [1] RSPM (R 4.4.0)
##  statnet.common      4.12.0     2025-05-30 [1] RSPM (R 4.4.0)
##  strataG           * 2.5.5      2025-07-25 [1] Github (ericarcher/strataG@bf71904)
##  stringi             1.8.7      2025-03-27 [2] RSPM (R 4.4.0)
##  stringr           * 1.6.0      2025-11-04 [1] RSPM (R 4.4.0)
##  survival            3.7-0      2024-06-05 [3] CRAN (R 4.4.2)
##  svglite             2.2.1      2025-05-12 [1] RSPM (R 4.4.0)
##  systemfonts         1.3.2      2026-03-05 [1] RSPM (R 4.4.0)
##  terra               1.8-54     2025-06-01 [1] RSPM (R 4.4.0)
##  textshaping         1.0.1      2025-05-01 [1] RSPM (R 4.4.0)
##  TH.data             1.1-3      2025-01-17 [1] RSPM (R 4.4.0)
##  tibble            * 3.3.1      2026-01-11 [1] RSPM (R 4.4.0)
##  tidyr             * 1.3.2      2025-12-19 [1] RSPM (R 4.4.0)
##  tidyselect          1.2.1      2024-03-11 [1] RSPM (R 4.4.0)
##  tidyverse         * 2.0.0      2023-02-22 [1] RSPM (R 4.4.0)
##  timechange          0.4.0      2026-01-29 [1] RSPM (R 4.4.0)
##  timeDate            4041.110   2024-09-22 [1] RSPM (R 4.4.0)
##  tree                1.0-45     2025-08-18 [1] RSPM (R 4.4.0)
##  tzdb                0.5.0      2025-03-15 [1] RSPM (R 4.4.0)
##  urlchecker          1.0.1      2021-11-30 [1] RSPM (R 4.4.0)
##  usethis             3.1.0      2024-11-26 [1] RSPM (R 4.4.0)
##  vctrs               0.7.3      2026-04-11 [1] RSPM (R 4.4.0)
##  vegan             * 2.7-1      2025-06-05 [1] RSPM (R 4.4.0)
##  viridisLite         0.4.3      2026-02-04 [1] RSPM (R 4.4.0)
##  vroom               1.7.1      2026-03-31 [1] RSPM (R 4.4.0)
##  withr               3.0.2      2024-10-28 [2] RSPM (R 4.4.0)
##  xfun                0.52       2025-04-02 [2] RSPM (R 4.4.0)
##  xml2                1.5.2      2026-01-17 [1] RSPM (R 4.4.0)
##  xtable              1.8-4      2019-04-21 [2] RSPM (R 4.4.0)
##  yaml                2.3.10     2024-07-26 [2] RSPM (R 4.4.0)
##  zlibbioc            1.52.0     2024-10-29 [1] Bioconductor 3.20 (R 4.4.2)
##  zoo                 1.8-15     2025-12-15 [1] RSPM (R 4.4.0)
##
##  [1] /home/kubeflow/R/x86_64-pc-linux-gnu-library/4.4
##  [2] /usr/local/lib/R/site-library
##  [3] /usr/local/lib/R/library
##  * -- Packages attached to the search path.
##
## ------------------------------------------------------------------------------------------------------
```
